# Evaluating agreement between bodies of evidence from randomized controlled trials and cohort studies in medical research: a meta-epidemiological study

**DOI:** 10.1186/s12916-022-02369-2

**Published:** 2022-05-11

**Authors:** Nils Bröckelmann, Sara Balduzzi, Louisa Harms, Jessica Beyerbach, Maria Petropoulou, Charlotte Kubiak, Martin Wolkewitz, Joerg J. Meerpohl, Lukas Schwingshackl

**Affiliations:** 1 Institute for Evidence in Medicine, Medical Center - University of Freiburg, Faculty of Medicine, University of Freiburg, Breisacher Straße 86, 79110 Freiburg, Germany; 2 Institute of Medical Biometry and Statistics, Faculty of Medicine and Medical Center - University of Freiburg, Freiburg, Germany; 3 Cochrane Germany, Cochrane Germany Foundation, Freiburg, Germany

**Keywords:** Meta-epidemiological study, Agreement of effect estimates, Randomized controlled trials, Cohort studies

## Abstract

**Background:**

Randomized controlled trials (RCTs) and cohort studies are the most common study design types used to assess the treatment effects of medical interventions. To evaluate the agreement of effect estimates between bodies of evidence (BoE) from randomized controlled trials (RCTs) and cohort studies and to identify factors associated with disagreement.

**Methods:**

Systematic reviews were published in the 13 medical journals with the highest impact factor identified through a MEDLINE search. BoE-pairs from RCTs and cohort studies with the same medical research question were included. We rated the similarity of PI/ECO (Population, Intervention/Exposure, Comparison, Outcome) between BoE from RCTs and cohort studies. The agreement of effect estimates across BoE was analyzed by pooling ratio of ratios (RoR) for binary outcomes and difference of mean differences for continuous outcomes. We performed subgroup analyses to explore factors associated with disagreements.

**Results:**

One hundred twenty-nine BoE pairs from 64 systematic reviews were included. PI/ECO-similarity degree was moderate: two BoE pairs were rated as “more or less identical”; 90 were rated as “similar but not identical” and 37 as only “broadly similar”. For binary outcomes, the pooled RoR was 1.04 (95% CI 0.97–1.11) with considerable statistical heterogeneity. For continuous outcomes, differences were small. In subgroup analyses, degree of PI/ECO-similarity, type of intervention, and type of outcome, the pooled RoR indicated that on average, differences between both BoE were small. Subgroup analysis by degree of PI/ECO-similarity revealed high statistical heterogeneity and wide prediction intervals across PI/ECO-dissimilar BoE pairs.

**Conclusions:**

On average, the pooled effect estimates between RCTs and cohort studies did not differ. Statistical heterogeneity and wide prediction intervals were mainly driven by PI/ECO-dissimilarities (i.e., clinical heterogeneity) and cohort studies. The potential influence of risk of bias and certainty of the evidence on differences of effect estimates between RCTs and cohort studies needs to be explored in upcoming meta-epidemiological studies.

**Supplementary Information:**

The online version contains supplementary material available at 10.1186/s12916-022-02369-2.

## Background

Randomized controlled trials (RCTs) and cohort studies are the most common study design types used to assess the treatment effects of medical interventions [1, 2]. RCTs are considered the gold standard in medical research to assess benefits and harms of treatments [1–3]. Randomization allows causal inference [4]. However, RCTs may not be available for certain research questions due to ethical reasons [5] or they may suffer from low external validity [6–9], too short follow-up duration to assess late adverse events [5], or low adherence [10]. In contrast to RCTs, large cohort studies may often have higher external validity [6], e.g., when including diverse populations [8, 9]. Cohort studies can complement information from RCTs or might even serve as a replacement [11] and enlarge the available body of evidence (BoE: all studies available for a given research question, i.e., all RCTs/cohort studies investigating the impact of oral contraception on breast cancer), or they may be useful to identify relevant subgroups for subsequent RCTs [12]. However, there is an ongoing debate about the trustworthiness of results from cohort studies mainly fuelled by their susceptibility to risk of bias by confounding [8, 13]. For example, systematic reviews from the Cochrane Collaboration impose high thresholds on the inclusion of cohort studies [5]. Several studies have investigated whether the susceptibility to bias in different types of observational studies indeed leads to disagreement of effect estimates [14–17]; the largest study so far, a meta-methodological study comparing health care outcomes from RCTs to observational studies (including case-control and cohort studies) concluded that results were mainly concordant [18]. The authors suggested that factors other than the study design only should be investigated in the case of disagreement of results. However, the study lacked an empirical investigation of factors such as PI/ECO (population, intervention/exposure, comparator, outcome)-differences (for example, differences between the interventions tested in RCTs and cohort studies) that potentially account for disagreement of study results and little is known about this topic so far. Therefore, in the present meta-epidemiological study, we do not only evaluate the agreement of effect estimates between BoE from RCTs and cohort studies from the general medical field. Additionally, we investigate whether factors such as PI/ECO-differences between BoE are associated with disagreement. This also allows us, to explore and to better understand potential reasons for statistical heterogeneity. Factors associated with disagreement would require special attention in future health-care evidence syntheses integrating both BoE.

## Methods

This meta-epidemiological study was planned, written, and reported in adherence to guidelines for reporting meta-epidemiological research [19]. The detailed inclusion criteria are described in Table 1.

**Table 1:** Detailed description of inclusion and exclusion criteria

	Inclusion criteria	Exclusion criteria
Methods	Systematic review of interventions/exposures including RCTs and cohort studies; equivalent search for RCTs and cohort studies; performing quantitative meta-analysis for at least one BoE.	Umbrella reviews, narrative reviews, systematic reviews of diagnostic test accuracy, Individual patient data meta-analysis; no quantitative meta-analysis
BoE-pairs	BoE-pair with a BoE from RCTs and a BoE from cohort studies evaluating the same medical research question (e.g. association of Exenatide with pancreatitis; effect of Vitamin D on hypertension; comparing total- with unicompartimental knee arthroplasty for range of movement of the knee)	Single small study (*n* < 1.000 participants) for one BoE (RCT or cohort studies); BoE-pair with one BoE using a continuous outcome and the other BoE using a binary outcome (e.g. risk of hypertension vs. mean difference of systolic blood pressure)
Population	All populations (e.g. primary prevention, secondary prevention, general population, adults, children)	-
Intervention/Exposure	All types of medical interventions and exposures (e.g. drugs, invasive procedures, nutrients, vaccines)	-
Comparator	All types of comparators (e.g. placebo, drugs, invasive procedures, nutrients, vaccines)	-
Outcomes	Patient-relevant outcomes (e.g. mortality, cancer outcomes, cardiovascular outcomes, obstetrical outcomes) and of intermediate disease markers (e.g. LDL-cholesterol)	-
Study design	Randomized controlled trials (e.g. parallel, cluster, factorial, cross-over); cohort studies (e.g. prospective cohort, retrospective cohort, observational cohort analysis of RCT)	Quasi-RCTs, non-randomized controlled trials, case-control studies, cross-sectional studies, ecological studies

*BoE* Bodies of evidence, *LDL* Low-density lipoprotein, *PI/ECO* Population, intervention/ exposure, comparator, outcome, *RCT* Randomized controlled trial

### Literature search

The search was conducted in MEDLINE (via PubMed.gov) on June 05, 2020, for the period between January 01, 2010, to December 31, 2019, in the 13 medical journals with the highest impact factor (according to the Journal Citation Report [JCR] 2018; category: general and internal medicine). This cut-off was chosen to cover a 10-year period in line with a recent meta-epidemiological study in nutrition research [20]. Initially, we planned to include the 10 highest impact factor journals, but three journals (*New England Journal of Medicine*, *Nature Reviews Disease Primers*, and *Journal of cachexia, sarcopenia, and muscle*) did not publish any systematic review with an eligible BoE-pair (see inclusion criteria in Table 1). We therefore included the subsequent three journals according to the JCR 2018 (*Cochrane Database of Systematic Reviews*, *Mayo Clinic Proceedings*, *Canadian Medical Association Journal*). The search strategy is given in Additional file 1 (Appendix S1). The title and abstract screening was conducted by one reviewer (NB), and potentially relevant full texts were screened by two reviewers independently (NB, LS). Any discrepancy was resolved by a third reviewer (JJM). Supplementary hand searches identified three additional systematic reviews [21–23]. For each included BoE from a systematic review, we included a maximum of three patient-relevant outcomes (e.g., mortality, cardiovascular disease (CVD)), and a maximum of three intermediate disease markers (e.g., blood lipids). If more than three outcomes were available for a given systematic review, we included the primary outcomes, and thereafter, we used a top-down approach (mentioned first).

### Evaluating similarity between BoE from RCTs and cohort studies

We evaluated the similarity of PI/ECO between BoE from RCTs and cohort studies. In accordance with a previous meta-epidemiological study [20], the acronym PI/ECO instead of PICO was used, to better represent exposures in cohort studies (e.g., serum vitamin D status) and to distinguish them from interventions in RCTs (e.g., vitamin D supplementation). For each BoE-pair, the similarity of each PI/ECO-domain was rated as “more or less identical,” “similar but not identical,” or “broadly similar.” Overall, the similarity of each BoE-pair was then determined according to the domain with the lowest degree of similarity. For example, when the PI/ECO-rating for the domain “population” was rated as “broadly similar” the overall similarity of this BoE-pair was also rated as “broadly similar.” The PI/ECO-similarity rating was conducted by two reviewers independently (NB, JB) using pre-specified criteria (Additional file 1: Table S1). Categorization of interventions and outcomes was conducted by two reviewers (NB, LH). Discrepancies of PI/ECO-similarity rating or categorizations were resolved through discussion with experts.

### Data extraction

Data extraction was performed by two reviewers independently (NB, LH). The following data were extracted for each BoE: effect estimates, type of effect measure, 95% confidence interval (CI), number of studies, number of participants, number of events, and certainty of the evidence. Further, we extracted information on study characteristics of primary studies for each BoE: description of the study population, intervention/exposure, comparator, design of the primary study, intervention duration, and follow-up and risk of bias/study quality.

If RCTs were pooled with other types of studies (e.g., quasi-experimental RCTs), we performed a meta-analysis excluding these other study types. The rationale for this approach was the suggestion in the new Cochrane handbook to classify quasi-experimental RCTs as non-randomized studies of interventions (NRSI) [5]. This was the case for three BoE from RCTs [24–26]. Accordingly, meta-analyses of cohort studies were recalculated if they included other study types (e.g., case-control studies); this was the case for 35 BoE from cohort studies [25, 27–42]. If RCTs and cohort studies were pooled without subgroup analysis by study type, we performed separate meta-analyses; this was the case for nine BoE-pairs [37, 40, 43–45]. Upon request, authors from one systematic review [45] provided data to perform separate meta-analyses. In two BoE-pairs from one systematic review evaluating infection outcomes of influenza vaccines [46] RCTs with different populations (community-dwelling and institutionalized) were combined in a single meta-analysis; we pooled respective cohort studies that were initially not combined. For ten BoE pairs [38, 42, 47, 48], we pooled different types of cohort studies (e.g., clinical cohorts, population-based cohorts) that were not pooled in the corresponding systematic review. If there was a meta-analysis for the BoE from one study type (e.g., RCTs) and a corresponding BoE from the other study type (e.g., cohort studies) was not pooled but relevant data were available, we pooled the respective primary studies: cohort studies for nine BoE pairs [49–55] and primary RCTs for one BoE pair [56].

### Statistical analysis

If the summary effect measure for binary or continuous outcomes was not the same for BoE from RCTs and BoE from cohort studies, we used the appropriate conversion formulas in order to have the two estimates expressed in the same measure: risk ratio (RR), odds ratio (OR), or hazard ratio (HR) for binary outcomes and mean difference (MD) for continuous outcomes.

If effect measures (RR, OR, HR) for binary outcomes were not the same within a BoE pair, they were converted to an identical effect measure (RR) using an assumed control risk (ACR); $\mathrm{RR}=\frac{\mathrm{OR}}{1-\mathrm{ACR}\ \mathrm{x}\ \left(1-\mathrm{OR}\right)}$ [13, 57]. If either a RR, OR, or HR was used for both BoE, we did not convert summary effect estimates. We converted effect measures for binary outcomes for 16 BoE pairs [22, 23, 44, 52–54, 56, 58–60] and for continuous outcomes for one BoE pair [61]. Detailed descriptions about the conversions can be found in Additional file 1 (Table S2 [62–66]). We standardized the direction of effect of the outcomes so that summary effect estimates (HR/OR/RR) <1 are always expressing a beneficial effect. We revised the direction of effect for three outcomes from the systematic reviews by Hüpfl et al. [67] (survival to all-cause mortality) and Alipanah et al. [24] (treatment success/completion to low treatment success, low treatment completion) (see Table 2). To quantify differences of effect estimates, we computed a ratio of ratios (RoR) [68] for each BoE pair with a binary outcome. For continuous outcomes, we computed a difference of mean differences (DMD). For the assessment of binary and continuous outcomes cohort studies served as the reference group. We pooled the RoRs across BoE-pairs using a random-effects model [69] to assess whether in total effect estimates of BoE from RCTs are larger or smaller in relation to those of BoE from cohort studies. The RoR does not indicate larger or smaller treatment effects in one of the BoE, but only differences between the two BoEs. The direction of difference depends on the direction of effect of the underlying BoEs. For example, a risk ratio from RCTs of 0.8 and a risk ratio from cohort studies of 1 would yield a RoR of 0.8, whereas a risk of 1.00 in RCTs compared with a risk ratio of 1.25 in cohort studies would also yield a RoR of 0.8. We pooled DMDs for the same continuous outcomes using a random-effects model [69]. We evaluated the statistical heterogeneity of effect estimates across all BoE-pairs with binary outcomes and across BoE pairs using the same continuous outcomes with the *I*^2^ and *τ*^2^ statistics [69, 70]. To estimate *τ*^2^, we used Paule and Mandel method [71, 72]. We computed 95% prediction intervals (PIs) to estimate the extent of differences between results of BoE from RCTs and BoE from cohort studies likely to occur in future comparisons. Meta-analyses were performed with the R package meta [73] using random-effects models [69].

**Table 2:** Effect estimates and overall PI/ECO-similarity degree for each included body of evidence-pair

Systematic review	Body of evidence-pair		RCTs		Cohort studies		PI/ECO-similarity degree^**a**^
	Intervention	Outcome	Number of studies	Summary measure; effect estimates (95% CI)	Number of studies	Summary measure; effect estimates (95% CI)	
Abou-Setta 2011 [74]	Nerve block	Delirium	4	OR: 0.33 (0.16, 0.66)	2	OR: 0.24 (0.08, 0.72)	2
Abou-Setta 2011 [74]	Spinal anesthesia	All-cause mortality	2	OR: 1.73 (0.53, 5.68)	5	OR: 0.87 (0.45, 1.67)	2
Aburto 2013 [75]	Low sodium	All-cause mortality	4	RR: 0.7 (0.44, 1.14)	7	RR: 0.94 (0.83, 1.06)	2
Aburto 2013 [75]	Low sodium	Cardiovascular disease	2	RR: 0.84 (0.57, 1.23)	9	RR: 0.89 (0.75, 1.08)	2
Ahmad 2015 [27]	Intra-aortic balloon pump	All-cause mortality	12	OR: 0.96 (0.74, 1.24)	14	OR: 1.02 (0.57, 1.82)	1
Alexander 2017 [76]	DHA and EPA	Coronary heart disease	18	RR: 0.94 (0.85, 1.05)	17	RR: 0.82 (0.74, 0.92)	2
Alexander 2017 [76]	DHA and EPA	Coronary heart disease mortality	14	RR: 1 (0.89, 1.11)	14	RR: 0.77 (0.66, 0.9)	2
Alexander 2017 [76]	DHA and EPA	Coronary heart disease incidence	9	RR: 0.92 (0.78, 1.09)	4	RR: 0.81 (0.55, 1.19)	2
Alipanah 2018 [24]	Self-administered therapy	Low treatment success	4	RR: 1.05 (0.96, 1.15)	16	RR: 1.23 (1.12, 1.37	3
Alipanah 2018 [24]	Self-administered therapy	Low treatment completion	5	RR: 1.27 (0.9, 1.79)	14	RR: 0.91 (0.74, 1.11)	3
Alipanah 2018 [24]	Self-administered therapy	All-cause mortality	4	RR: 0.73 (0.45, 1.19)	23	RR: 1.35 (1, 1.84)	3
Anglemyer 2013 [77]	Antiretroviral therapy	HIV infection	1	RR: 0.11 (0.04, 0.32)	9	RR: 0.58 (0.35, 0.96)	3
Azad 2017 [21]	Nonnutritive sweeteners	Body Mass Index	3	MD: -0.37 (-1.1, 0.36)	1	MD: 0.77 (0.47, 1.07)	2
Barnard 2015 [28]	Surgical abortion by mid-level providers	Failure or incomplete abortion	2	RR: 2.97 (0.21, 41.82)	2	RR: 2.47 (1.45, 4.22)	2
Barnard 2015 [28]	Surgical abortion by mid-level providers	Complications	2	RR: 0.99 (0.17, 5.7)	2	RR: 1.3 (0.57, 2.96)	2
Barnard 2015 [28]	Surgical abortion by mid-level providers	Abortion failure and complications	2	RR: 3.07 (0.16, 59.08)	3	RR: 1.33 (0.78, 2.27)	2
Bellemain-Appaix 2012 [48]	Clopidogrel	All-cause mortality	7	OR: 0.8 (0.57, 1.11)	8	OR: 0.79 (0.52, 1.2)	2
Bellemain-Appaix 2012 [48]	Clopidogrel	Major bleeding	7	OR: 1.18 (0.93, 1.5)	8	OR: 1.16 (0.83, 1.61)	2
Bellemain-Appaix 2012 [48]	Clopidogrel	Coronary heart disease	7	OR: 0.77 (0.66, 0.89)	8	OR: 0.76 (0.6, 0.95)	2
Bellemain-Appaix 2014 [47]	P2Y12 inhibitor	All-cause mortality	3	OR: 0.92 (0.43, 1.98)	4	OR: 0.69 (0.38, 1.25)	2
Bellemain-Appaix 2014 [47]	P2Y12 inhibitor	Major bleeding	3	OR: 1.45 (0.97, 2.15)	4	OR: 1.12 (0.87, 1.45)	2
Bellemain-Appaix 2014 [47]	P2Y12 inhibitor	Main composite ischemic endpoint	3	OR: 0.85 (0.67, 1.07)	4	OR: 0.79 (0.54, 1.15)	2
Bloomfield 2016 [22]	Mediterranean diet	Breast cancer	1	RR: 0.53 (0.28, 1.03)	13	RR: 0.96 (0.9, 1.03)	2
Bolland 2015 [49]	Calcium	All fractures	22	RR: 0.9 (0.83, 0.96)	5	RR: 1.02 (0.93, 1.12)	2
Bolland 2015 [49]	Calcium	Vertebral fracture	12	RR: 0.86 (0.74, 1)	1	RR: 1.4 (1.1, 1.9)	2
Bolland 2015 [49]	Calcium	Hip fracture	13	RR: 0.95 (0.76, 1.18)	6	RR: 1.09 (0.91, 1.3)	2
Brenner 2014 [29]	Sigmoidoscopy	Colorectal cancer mortality	4	RR: 0.72 (0.65, 0.8)	1	RR: 0.59 (0.45, 0.76)	1
Brenner 2014 [29]	Sigmoidoscopy	Colorectal cancer incidence	4	RR: 0.82 (0.75, 0.89)	2	RR: 0.5 (0.37, 0.69)	2
Chowdhury 2012 [78]	Omega-3	Cerebrovascular disease	2	RR: 0.98 (0.89, 1.08)	10	RR: 0.9 (0.8, 1.01)	2
Chowdhury 2014a [79]	α-linolenic acid	Coronary heart disease	4	RR: 0.97 (0.69, 1.36)	7	RR: 0.99 (0.86, 1.14)	3
Chowdhury 2014a [79]	Omega-3	Coronary heart disease	17	RR: 0.94 (0.86, 1.03)	16	RR: 0.87 (0.78, 0.97)	3
Chowdhury 2014a [79]	Omega-6	Coronary heart disease	8	RR: 0.86 (0.69, 1.07)	8	RR: 0.98 (0.9, 1.06)	3
Chowdhury 2014b [80]	Vitamin D	All-cause mortality	22	RR: 0.98 (0.94, 1.02)	68	RR: 0.69 (0.65, 0.75)	3
Chung 2011 [58]	Vitamin D	Colorectal cancer	1	RR: 1.02 (0.6, 1.74)	9	RR: 0.94 (0.91, 0.97)	3
Chung 2011 [58]	Vitamin D	Breast cancer	1	RR: 0.99 (0.25, 4)	4	RR: 0.99 (0.97, 1.01)	3
Chung 2016 [56]	Calcium	Cardiovascular mortality	2	RR: 1.05 (0.82, 1.33)	6	RR: 0.99 (0.97, 1.01)	2
Ding 2017 [81]	Dairy	Systolic blood pressure	8	MD: -0.21 (-0.98, 0.57)	27	MD: -0.11 (-0.2, -0.02)	2
Fenton 2018 [30]	Radiation therapy	Erectile dysfunction	1	RR: 0.91 (0.77, 1.08)	7	RR: 1.3 (1.19, 1.43)	2
Fenton 2018 [30]	Radical Prostatectomy	Urinary incontinence	3	RR: 2.27 (1.82, 2.84)	5	RR: 2.92 (1.8, 4.71)	2
Fenton 2018 [30]	Radical Prostatectomy	Erectile dysfunction	3	RR: 1.6 (1.23, 2.07)	6	RR: 1.49 (1.33, 1.66)	2
Filippini 2017 [43]	Disease-modifying drugs	Conversion to clinically definite multiple sclerosis	7	HR: 0.52 (0.46, 0.6)	2	HR: 0.48 (0.3, 0.78)	2
Fluri 2010 [31]	Extracranial-intracranial arterial bypass	All-cause mortality	2	OR: 0.81 (0.62, 1.05)	11	OR: 1 (0.62, 1.63)	2
Fluri 2010 [31]	Extracranial-intracranial arterial bypass	Stroke	2	OR: 0.99 (0.79, 1.23)	15	OR: 0.8 (0.54, 1.18)	2
Fluri 2010 [31]	Extracranial-intracranial arterial bypass	Stroke mortality or dependency	1	OR: 0.94 (0.74, 1.21)	8	OR: 0.8 (0.5, 1.29)	2
Gargiulo 2016 [32]	Transcatheter aortic valve	Early all-cause mortality	5	OR: 0.8 (0.51, 1.25)	29	OR: 1.08 (0.84, 1.39)	2
Gargiulo 2016 [32]	Transcatheter aortic valve	Mid-term all-cause mortality	5	OR: 0.9 (0.64, 1.26)	18	OR: 1 (0.81, 1.24)	2
Gargiulo 2016 [32]	Transcatheter aortic valve	Long-term all-cause mortality	4	OR: 1.03 (0.65, 1.62)	6	OR: 1.7 (1.23, 2.35)	2
Hartling 2013 [50]	Treating gestational diabetes mellitus	High birth weight	5	RR: 0.5 (0.35, 0.71)	5	RR: 0.69 (0.31, 1.54)	2
Hartling 2013 [50]	Treating gestational diabetes mellitus	Large-for-gestational age neonate	3	RR: 0.56 (0.45, 0.69)	4	RR: 0.43 (0.27, 0.7)	2
Hartling 2013 [50]	Treating gestational diabetes mellitus	Shoulder dystocia	3	RR: 0.42 (0.23, 0.77)	4	RR: 0.38 (0.19, 0.78)	2
Henderson 2019 [51]	Treating asymptomatic bacteriuria	Pyelonephritis	12	RR: 0.24 (0.14, 0.4)	2	RR: 0.29 (0.15, 0.57)	3
Higgins 2016 [25]	Bacillus Calmette-Guérin	All-cause mortality	3	RR: 0.67 (0.4, 1.14)	8	RR: 0.46 (0.3, 0.69)	3
Higgins 2016 [25]	Measles containing vaccines	All-cause mortality	4	RR: 0.74 (0.51, 1.07)	13	RR: 0.53 (0.4, 0.7)	3
Hopley 2010 [33]	Total hip arthroplasty	Reoperation	4	RR: 1.09 (0.4, 2.99)	6	RR: 0.45 (0.18, 1.09)	2
Hopley 2010 [33]	Total hip arthroplasty	Dislocation	4	RR: 2.47 (0.69, 8.76)	5	RR: 0.8 (0.27, 2.39)	2
Hopley 2010 [33]	Total hip arthroplasty	Deep infection	4	RR: 1.71 (0.66, 4.45)	4	RR: 0.91 (0.25, 3.28)	2
Hüpfl 2010 [67]	Chest-compression-only cardiopulmonary resuscitation	All-cause mortality	3	RR: 0.82 (0.68, 0.99)	7	RR: 1.04 (0.9, 1.2)	3
Jamal 2013 [82]	Non-calcium-based phosphate binders	All-cause mortality	8	RR: 0.78 (0.61, 0.98)	3	RR: 0.89 (0.78, 1)	2
Jefferson 2010 [46]	Parenteral influenza vaccine	Influenza-like illness	4	RR: 0.59 (0.47, 0.73)	30	RR: 0.76 (0.66, 0.87)	3
Jefferson 2010 [46]	Parenteral influenza vaccine	Influenza	3	RR: 0.42 (0.27, 0.66)	10	RR: 0.5 (0.26, 0.97)	2
Jefferson 2012 [34]	Inactivated influenza vaccines	Influenza	5	RR: 0.41 (0.29, 0.59)	1	RR: 0.2 (0.1, 0.39)	2
Jefferson 2012 [34]	Inactivated influenza vaccines	Influenza-like illness	5	RR: 0.64 (0.54, 0.76)	2	RR: 0.29 (0.07, 1.15)	2
Jin 2012 [83]	Total flavonoids	Colorectal neoplasms	1	RR: 1.09 (0.93, 1.28)	3	RR: 1 (0.8, 1.25)	3
Johnston 2019 [23]	Low red meat	All-cause mortality	1	RR: 0.94 (0.89, 0.99)	24	RR: 0.87 (0.82, 0.92)	2
Johnston 2019 [23]	Low red meat	Cardiovascular mortality	1	RR: 1 (0.84, 1.19)	25	RR: 0.86 (0.79, 0.94)	2
Johnston 2019 [23]	Low red meat	Cardiovascular disease	1	RR: 0.97 (0.91, 1.04)	12	RR: 0.87 (0.75, 1.01)	2
Kansagara 2013 [52]	Transfusion	All-cause mortality	6	RR: 0.94 (0.61, 1.42)	11	RR: 2.49 (1.4, 4.43)	3
Keag 2018 [84]	Caesarean section	Urinary incontinence	1	OR: 0.78 (0.56, 1.08)	8	OR: 0.56 (0.47, 0.66)	3
Keag 2018 [84]	Caesarean section	Fecal incontinence	1	OR: 3.07 (0.9, 10.49)	5	OR: 1.04 (0.73, 1.48)	3
Kredo 2014 [85]	Starting and maintaining antiretroviral therapy	All-cause mortality	1	RR: 0.96 (0.82, 1.12)	2	RR: 1.23 (1.14, 1.33)	3
Kredo 2014 [85]	Starting and maintaining antiretroviral therapy	Attrition	1	RR: 0.73 (0.55, 0.97)	2	RR: 0.3 (0.05, 1.94)	3
Kredo 2014 [85]	Maintaining antiretroviral therapy	All-cause mortality	2	RR: 0.89 (0.59, 1.32)	1	RR: 0.19 (0.05, 0.78)	3
Li 2014 [54]	Exenatide	Acute pancreatitis	5	RR: 0.86 (0.22, 3.37)	2	RR: 0.92 (0.69, 1.22)	2
Li 2016 [53]	DDP-4 inhibitors	Heart failure	34	RR: 0.9 (0.61, 1.35)	4	RR: 1.1 (1.04, 1.16)	2
Li 2016 [53]	DDP-4 inhibitors	Hospital admission for heart failure	5	OR: 1.13 (1, 1.27)	6	OR: 0.85 (0.74, 0.97)	2
Matthews 2018 [86]	Tamoxifen	Heart failure	1	RR: 0.52 (0.33, 0.71)	2	RR: 0.84 (0.65, 1.07)	3
Menne 2019 [87]	SGLT-2 inhibitors	Acute kidney injury	41	OR: 0.75 (0.66, 0.84)	5	OR: 0.4 (0.33, 0.48)	2
Mesgarpour 2017 [88]	Erythropoiesis stimulating agents	Venous thromboembolism	12	RR: 1.12 (0.9, 1.4)	5	RR: 1.87 (0.59, 5.92)	2
Mesgarpour 2017 [88]	Erythropoiesis stimulating agents	All-cause mortality	17	RR: 0.81 (0.71, 0.93)	7	RR: 1.07 (0.65, 1.77)	2
Moberley 2013 [89]	Pneumococcal polysaccharide vaccines	Invasive pneumococcal disease	10	OR: 0.26 (0.14, 0.45)	2	OR: 0.57 (0.36, 0.89)	2
Molnar 2015 [35]	Neoral (Cyclosporin)	Acute rejection of kidney transplant	2	OR: 1.23 (0.64, 2.36)	2	OR: 0.47 (0.27, 0.83)	2
Navarese 2013 [90]	Early intervention for NSTE-ACS	All-cause mortality	7	OR: 0.83 (0.64, 1.09)	4	OR: 0.8 (0.63, 1.02)	2
Navarese 2013 [90]	Early intervention for NSTE-ACS	Myocardial infarction	7	OR: 1.15 (0.65, 2.01)	3	OR: 0.86 (0.69, 1.08)	2
Navarese 2013 [90]	Early intervention for NSTE-ACS	Major bleeding	7	OR: 0.76 (0.56, 1.04)	3	OR: 1.12 (0.69, 1.82)	2
Nelson 2010 [36]	Caesarean section	Anal incontinence, feces	1	OR: 1 (0.49, 2.05)	11	OR: 0.91 (0.72, 1.16)	3
Nelson 2010 [36]	Caesarean section	Anal incontinence, flatus	1	OR: 0.83 (0.51, 1.36)	4	OR: 1.02 (0.87, 1.2)	3
Nieuwenhuijse 2014 [37]	Ceramic-on-ceramic bearings for total hip arthroplasty	Harris Hip Score	7	MD: -0.23 (-1.09, 0.63)	3	MD: -0.5 (-2.09, 1.09)	2
Nieuwenhuijse 2014 [37]	High-flexion total knee arthroplasty	Flexion	20	MD: 1.68 (0.28, 3.08)	26	MD: 3.78 (1.64, 5.92)	2
Nieuwenhuijse 2014 [37]	Gender-specific total knee arthroplasty	Flexion-extension range	6	MD: 1.41 (-0.17, 2.99)	2	MD: 3.15 (-0.03, 6.34)	2
Nikooie 2019 [55]	Second generation antipsychotics	Sedation	6	RR: 1.26 (0.92, 1.72)	3	RR: 1.84 (0.4, 8.54)	2
Nikooie 2019 [55]	Second generation antipsychotics	Neurologic outcomes	6	RR: 0.45 (0.2, 1.01)	5	RR: 0.76 (0.59, 0.99)	2
Ochen 2019 [91]	Surgery for achilles tendon rupture	Re-rupture	10	RR: 0.4 (0.24, 0.69)	18	RR: 0.42 (0.28, 0.64)	2
Ochen 2019 [91]	Surgery for achilles tendon rupture	Complications	9	RR: 3.26 (1.26, 8.41)	15	RR: 2.93 (2.28, 3.75)	2
Pittas 2010 [60]	Vitamin D	Hypertension	1	RR: 1.01 (0.97, 1.05)	3	RR: 0.57 (0.41, 0.79)	3
Raman 2013 [38]	Carotid endarterectomy	Ipsilateral stroke	3	RR: 0.72 (0.58, 0.9)	2	RR: 0.47 (0.05, 4.46)	2
Raman 2013 [38]	Carotid endarterectomy	Stroke	3	RR: 0.68 (0.56, 0.82)	3	RR: 0.73 (0.43, 1.22)	2
Raman 2013 [38]	Carotid artery stenting	Periprocedural stroke	2	RR: 1.75 (0.87, 3.52)	5	RR: 1.91 (1.72, 2.11)	2
Schweizer 2013 [39]	Nasal deconolization	Surgical site infection	5	RR: 0.63 (0.36, 1.13)	6	RR: 0.4 (0.28, 0.57)	2
Schweizer 2013 [39]	Glycopeptide prophylaxis	Surgical site infection	8	RR: 1.13 (0.9, 1.42)	7	RR: 0.34 (0.11, 1.1)	2
Silvain 2012 [40]	Enoxaparin	All-cause mortality	6	RR: 0.88 (0.7, 1.1)	7	RR: 0.49 (0.39, 0.62)	2
Silvain 2012 [40]	Enoxaparin	Major bleeding	9	RR: 0.88 (0.62, 1.24)	7	RR: 0.72 (0.56, 0.93)	2
Silvain 2012 [40]	Enoxaparin	All-cause mortality or myocardial infarction	13	RR: 0.86 (0.74, 0.99)	7	RR: 0.44 (0.35, 0.55)	2
Suthar 2012 [26]	Antiretroviral therapy	Tuberculosis infection	2	HR: 0.5 (0.34, 0.75)	9	HR: 0.32 (0.25, 0.41)	3
Te Morenga 2013 [61]	Sugar	Weight gain	10	MD: 0.75 (0.3, 1.19)	4	MD: 0.31 (-0.07, 0.68)	2
Te Morenga 2013 [61]	Sugar	Body Mass Index	3	MD: -0.06 (-0.15, 0.04)	4	MD: 0.02 (0.00, 0,05)	2
Thomas 2010 [92]	nfluenza vaccin	Influenza-like illness	3	RR: 0.71 (0.55, 0.9)	1	RR: 0.31 (0.26, 0.36)	3
Tickell-Painter 2017 [93]	Mefloquine	Discontinuation due to adverse effects	3	RR: 2.86 (1.53, 5.31)	9	RR: 2.73 (1.83, 4.08)	2
Tickell-Painter 2017 [93]	Mefloquine	Serious adverse events or effects	3	RR: 0.7 (0.14, 3.53)	2	RR: 3.08 (0.39, 24.11)	3
Tickell-Painter 2017 [93]	Mefloquine	Nausea	2	RR: 1.35 (1.05, 1.73)	3	RR: 1.85 (1.42, 2.43)	3
Tricco 2018 [45]	Live-attenuated zoster vaccines	Suspected Herpes Zoster	5	RR: 0.61 (0.48, 0.93)	3	RR: 0.48 (0.27, 0.84)	2
Vinceti 2018 [59]	Selenium	Cancer	5	RR: 0.99 (0.86, 1.14)	7	RR: 0.75 (0.59, 0.94)	3
Vinceti 2018 [59]	Selenium	Cancer mortality	2	RR: 0.81 (0.49, 1.32)	7	RR: 0.77 (0.6, 0.97)	3
Vinceti 2018 [59]	Selenium	Colorectal cancer	3	RR: 0.74 (0.41, 1.33)	6	RR: 0.82 (0.72, 0.94)	3
Wilson 2011 [41]	Traditional birth attendants	Perinatal mortality	5	RR: 0.76 (0.64, 0.88)	1	RR: 0.82 (0.38, 1.78)	3
Wilson 2011 [41]	Traditional birth attendants	Neonatal mortality	6	RR: 0.79 (0.69, 0.88)	2	RR: 0.8 (0.47, 1.37)	3
Wilson 2019 [42]	Unicompartimental knee arthroplasty	Venous thromboembolism	2	RR: 0.24 (0.04, 1.37)	8	RR: 0.41 (0.29, 0.57)	2
Wilson 2019 [42]	Unicompartimental knee arthroplasty	Flexion-extension range	3	MD: -4.58 (-10.75, 1.59)	11	MD: -8.43 (-10.15, -6.71)	2
Wilson 2019 [42]	Unicompartimental knee arthroplasty	Operation duration	3	MD: -1.72 (-11.89, 8.45)	8	MD: -23.8 (-40.43, -7.17)	2
Yank 2011 [44]	Recombinant factor VII	All-cause mortality	2	RR: 1.4 (0.49, 4.02)	2	RR: 0.91 (0.39, 2.12)	2
Yank 2011 [44]	Recombinant factor VII	Thromboembolism	2	RR: 2.06 (0.48, 8.84)	2	RR: 1.81 (0.67, 4.87)	2
Zhang 2016 [94]	Everolimus-eluting bioresorbable vascular scaffold	Stent thrombosis	5	OR: 2.05 (0.95, 4.43)	3	OR: 2.32 (1.06, 5.07)	2
Zhang 2016 [94]	Everolimus-eluting bioresorbable vascular scaffold	All-cause mortality	5	OR: 0.96 (0.46, 2)	4	OR: 0.57 (0.23, 1.44)	2
Zhang 2016 [94]	Everolimus-eluting bioresorbable vascular scaffold	Coronary heart disease mortality	3	OR: 1.4 (0.45, 4.33)	4	OR: 0.81 (0.38, 1.7)	2
Zhang 2017 [95]	Percutaneous coronary intervention	All-cause mortality	5	HR: 1 (0.79, 1.26)	17	HR: 1.08 (0.92, 1.26)	2
Zhang 2017 [95]	Percutaneous coronary intervention	Cardiovascular mortality	4	HR: 1 (0.72, 1.39)	5	HR: 1.08 (0.51, 2.29)	2
Zhang 2017 [95]	Percutaneous coronary intervention	Myocardial infarction	5	HR: 1.39 (0.85, 2.27)	5	HR: 2.01 (1.64, 2.45)	2
Ziff 2015 [96]	Digoxin	All-cause mortality	7	RR: 0.99 (0.93, 1.05)	8	RR: 1.61 (1.31, 1.97)	3
Ziff 2015 [96]	Digoxin	Cardiovascular mortality	5	RR: 1.01 (0.94, 1.08)	3	RR: 2.53 (1.12, 5.71)	3
Ziff 2015 [96]	Digoxin	Hospital admission	2	RR: 0.94 (0.9, 0.99)	4	RR: 0.91 (0.87, 0.95)	2

*DDP-4* Dipeptidyl peptidase 4, *DHA* Docosahexaenoic acid, *EPA* Eicosapentaenoic acid, *HR* Hazard ratio, *NSTE-ACS* Non-ST elevation acute coronary syndrome, *OR* Odds raio, *PI/ECO* Population, intervention/ exposure, comparison, outcome, *RR* Risk ratio, *SGLT-2* Sodium glucose transporter 2;

^a^PI/ECO (population, intervention/ exposure, comparator, outcome)-similarity degree: 1 = more or less identical; 2 = similar but not identical; 3 = broadly similar

### Subgroup and sensitivity analyses

We performed pre-specified and post hoc subgroup analyses to explore factors potentially related to the disagreement of effect estimates. The study protocol specified subgroup analysis by degree of PI/ECO-similarity and intervention type (drug, invasive procedure, nutrient, vaccine). Post hoc subgroup analyses were performed by the type of binary effect estimate (RR, OR, HR), type of intervention stratified by degree of PI/ECO-similarity, and type of outcome (e.g., CVD outcomes, cancer outcomes). We performed a post hoc multivariable meta-regression among “similar but not identical” BoE pairs with binary outcomes. For each PI/ECO-domain, the average effect on the pooled RoR of the category “similar but not identical” was evaluated as compared to the reference category “more or less identical.” We performed two post hoc sensitivity analyses: First, by including only the BoE pair from each systematic review with the highest number of RCTs (if the number of RCTs was equal, we primarily included the BoE with the highest number of participants, followed by the highest number of events, followed by the highest number of cohort studies) and second, by direction of cohort study summary effect estimate (HR, OR, RR <1 vs. HR, OR, RR ≥1).

### Patient involvement

No patients were involved in setting the research question or the outcome measures, nor were they involved in developing plans for the design or implementation of the study. No patients were asked for advice on interpretation or writing up of results. There are no plans to disseminate the results of the research to study participants or the relevant patient community.

## Results

The literature search identified 1362 records of which 234 full texts were assessed for inclusion and 64 systematic reviews were included in this study (Additional file 1: Fig. S1 and Table S3). Overall, we included 129 BoE pairs [21–56, 58–61, 67, 74–96] (Table 2). Three journals contributed a major part of systematic reviews (*n* = 51; 80%): the *BMJ* (*n*=22), *Annals of Internal Medicine* (*n* = 15), and the *Cochrane Database of Systematic* Reviews (*n* = 14). The number of studies in BoE from RCTs ranged from 1 to 41 (median: 4) and from 1 to 68 (median: 5) in BoE from cohort studies. The range of participants was 99 to 437,600 (median: 3541) in BoE from RCTs and 162 to 1,934,183 (median: 12,850) in BoE from cohort studies. We performed re-analyses for 70 BoE pairs from 38 systematic reviews [22–25, 27–56, 58–61].

Interventions in BoE pairs (*n* = 129) consisted of invasive procedures (*n* = 44), drugs (*n* = 40), nutrition (*n* = 32), vaccines (*n* = 9), birth assistance (*n* = 2), blood transfusions (*n* = 1), and cardiopulmonary resuscitation (*n* = 1). The outcomes of the 129 BoE pairs were categorised as follows: all-cause mortality (*n* = 28), CVD outcomes (*n* = 27), drug safety outcomes including adherence outcomes (*n* = 20), infection outcomes (*n* = 14), orthopedic outcomes (*n* = 13), obstetrical outcomes (*n* = 10), oncological outcomes (*n* = 9), metabolic outcomes (*n* = 3), urological outcomes (*n* = 3), and neurological outcomes (*n* = 2).

The most frequently used tools for risk of bias assessment were the Cochrane risk of bias tool [97] for 94 (73%) BoE from RCTs and the Newcastle Ottawa scale [98] for 61 (47%) BoE from cohort studies. Certainty of the evidence ratings using GRADE [99] or Agency for Healthcare Research and Quality criteria [100] were available for 38 BoE from RCTs and 31 BoE from cohort studies. Study characteristics for each BoE including effect estimates, detailed descriptions of PI/ECO, the certainty of the evidence ratings, and study quality/risk of bias ratings of primary studies are depicted in Additional file 1 (Tables S4-S7); Additional file 1 (Table S8) shows an overview of the instruments that were used for risk of bias assessment.

### Similarity degree

Two (1.5%) BoE pairs were rated as “more or less identical”; 90 (69.8%) were rated as “similar but not identical” and 37 (28.7%) as “broadly similar”. The rating “broadly similar” was due to differences of study populations (*n* = 16), interventions and comparators (*n* = 20), and both population and outcome (*n* = 1) (Table 3, Additional file 1: Table S9).

**Table 3:** Ratings of PI/ECO-similarity degree for the included body of evidence-pairs by each PI/ECO-element

Similarity rating	Population	Intervention/Exposure	Comparator	Outcome	Overall
More or less identical	18/129 14%	41/129 32%	61/129 47%	120/129 93%	2/129 2%
Similar but not identical	94/129 73%	73/129 56%	52/129 40%	8/129 6%	90/129 70%
Broadly similar	17/129 13%	15/129 12%	16/129 13%	1/129 1%	37/129 28%

*PI/ECO* Population, intervention/ exposure, comparator, outcome

### Statistical heterogeneity of included individual comparisons

Median *I*^2^ across meta-analyses of RCTs was 8% and 46% across meta-analyses of cohort studies. For binary outcomes, median *I*^2^ was 4% for meta-analyses of RCTs and 44% for meta-analyses of cohort studies. For continuous outcomes, *I*^2^ was 9% across meta-analyses of RCTs and 69% across meta-analyses of cohort studies. Median *I*^2^ across meta-analyses with binary outcomes stratified by PI/ECO-similarity degree indicated higher statistical heterogeneity for “broadly similar” BoE: *I*^2^ was 23% for meta-analyses from RCTs and *I*^2^ was 62% for meta-analyses from cohort studies, whereas for “more or less identical” BoE, *I*^2^ was 0% for meta-analyses of RCTs and *I*^2^ was 34% for meta-analyses of cohort studies (Additional file 1: Table S10).

### Meta-epidemiological analysis

**Fig. 1:**
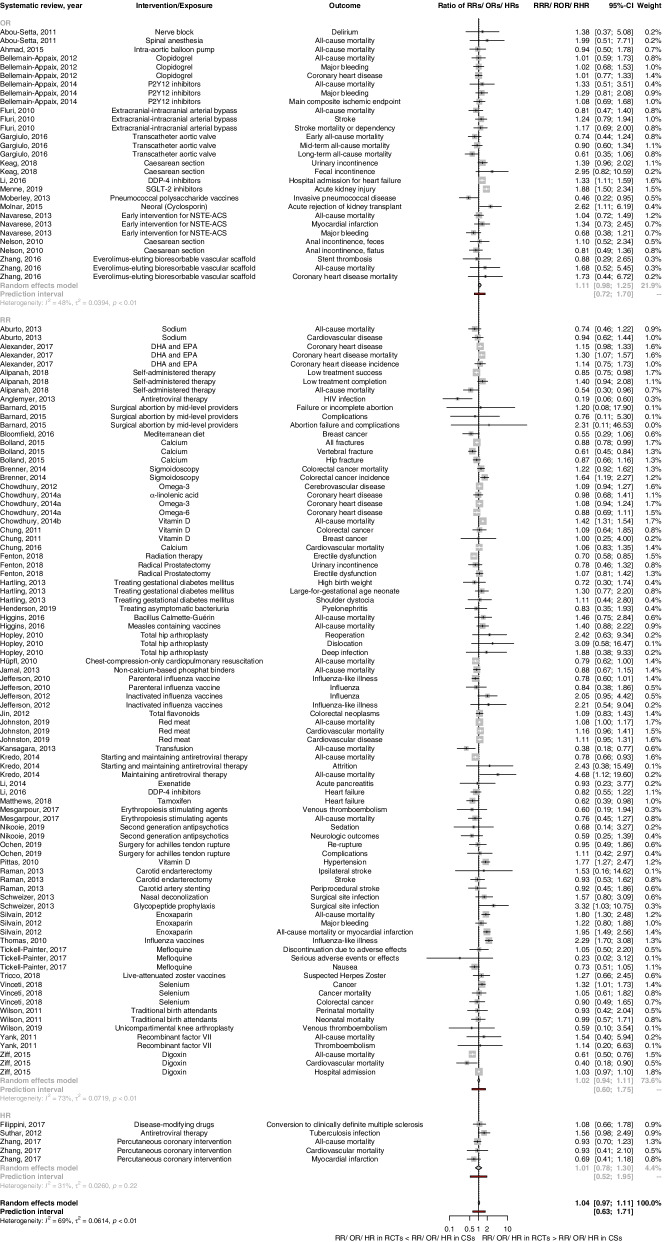
Forest plot for binary outcomes, pooled ratio of ratios (RoR) for bodies of evidence from randomized controlled trials vs. cohort studies stratified by type of effect measure. CSs cohort studies, DDP-4 dipeptidyl peptidase 4, DHA docosahexaenoic acid, EPA eicosapentaenoic acid, HR hazard ratio, NSTE-ACS= non-ST elevation acute coronary syndrome, OR odds ratio, RCTs randomized controlled trials, RHR ratio of hazard ratios, ROR ratio of odds ratios, RR risk ratio, RRR ratio of risk ratios, SGLT-2 sodium glucose transporter 2

**Fig. 2:**
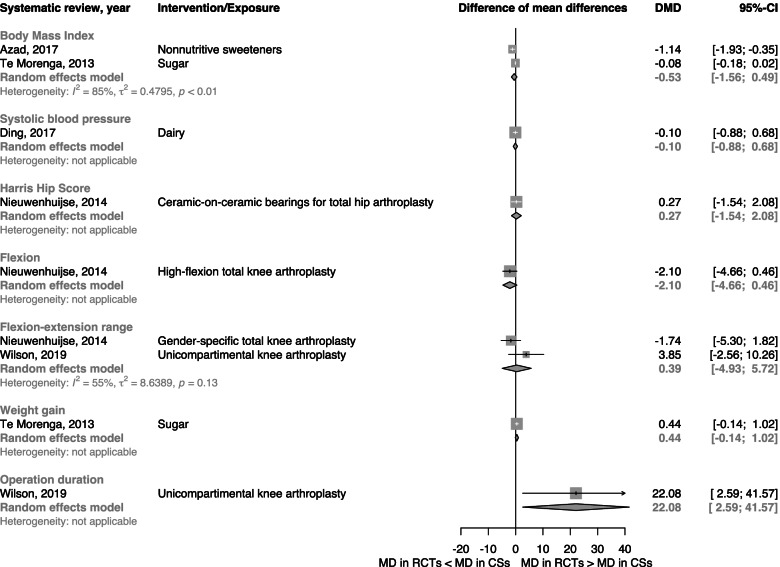
Forest plot for continuous outcomes, pooled difference of mean differences (DMD) for bodies of evidence from randomized controlled trials vs. cohort studies. CSs cohort studies, DMD difference of mean differences, MD mean difference, RCTs randomized controlled trials

Pooling RoRs across BoE pairs with binary outcomes resulted in a pooled RoR of 1.04 (95% CI 0.97 to 1.11; *n* = 120) with considerable statistical heterogeneity (*I*^2^ = 69%; *τ*^2^ = 0.061; 95% PI 0.63 to 1.71) (Fig. 1 and Table 4). Differences of MDs in continuous outcomes (*n* = 9) were mostly small, with the exception of operation duration for two types of knee prostheses where clear disagreement was shown [42] (Fig. 2).

**Table 4:** Overview of main results for binary outcomes (*n*=120)

Analysis	Number of BoE-pairs	Ratio of ratios; 95% CI	Heterogeneity (I^**2**^ (%);τ^**2**^)	95% prediction interval
Main	120	1.04 (0.97 to 1.11)	69; 0.061	0.63 to 1.71
**Stratified by type of binary effect measure**				
Risk ratios	85	1.02 (0.94 to 1.11)	73; 0.072	0.60 to 1.75
Odds ratios	30	1.11 (0.98 to 1.25)	48; 0.039	0.72 to 1.70
Hazard ratios	5	1.01 (0.78 to 1.30)	31; 0.026	0.52 to 1.95
**Stratified by degree of overall PI/ECO-similarity**				
More or less identical	2	1.17 (0.90 to 1.51)	0; 0.00	-
Similar but not identical	81	1.06 (0.99 to 1.14)	54; 0.034	0.73 to 1.54
Broadly similar	37	0.99 (0.85 to 1.16)	82; 0.149	0.45 to 2.21
**Stratified by type of intervention**^**a**^				
Drugs	40	1.04 (0.89 to 1.21)	76; 0.139	0.48 to 2.24
Invasive procedures	39	1.00 (0.91 to 1.10)	25; 0.011	0.79 to 1.26
Nutrition	28	1.07 (0.98 to 1.16)	71; 0.023	0.77 to 1.48
**Stratified by outcome-category**^**a**^				
All-cause mortality	28	0.94 (0.82 to 1.09)	80; 0.075	0.53 to 1.69
Cardiovascular disease outcomes	26	1.12 (1.02 to 1.23)	43; 0.022	0.81 to 1.55
Drug safety outcomes	20	1.06 (0.89 to 1.26)	67; 0.068	0.60 to 1.90

*BoE* Body of evidence, *CI* Confidence interval, *PI/ECO* Population, intervention/ exposure, comparator, outcome

^a^Only results of the largest subgroups are shown; detailed results are reported in Additional file 1 (Figs. S2a-S7)

### Subgroup analyses

For BoE pairs using RRs as summary effect estimate the pooled RoR was 1.02 (95% CI 0.94 to 1.11; *I*^2^= 73%; *τ*^2^= 0.072; 95% PI 0.60 to 1.75; *n*=85) and RoR 1.11 (95% CI 0.98 to 1.25; *I*^2^=48%; *τ*^2^=0.039; 95% PI 0.72 to 1.70; *n*=30), RoR 1.01 (95% CI 0.78 to 1.30; *I*^2^= 31%; *τ*^2^= 0.026; 95% PI 0.52 to 1.95; *n*=5) for ORs and HRs, respectively (Fig. 1 and Table 4).

**Fig. 3:**
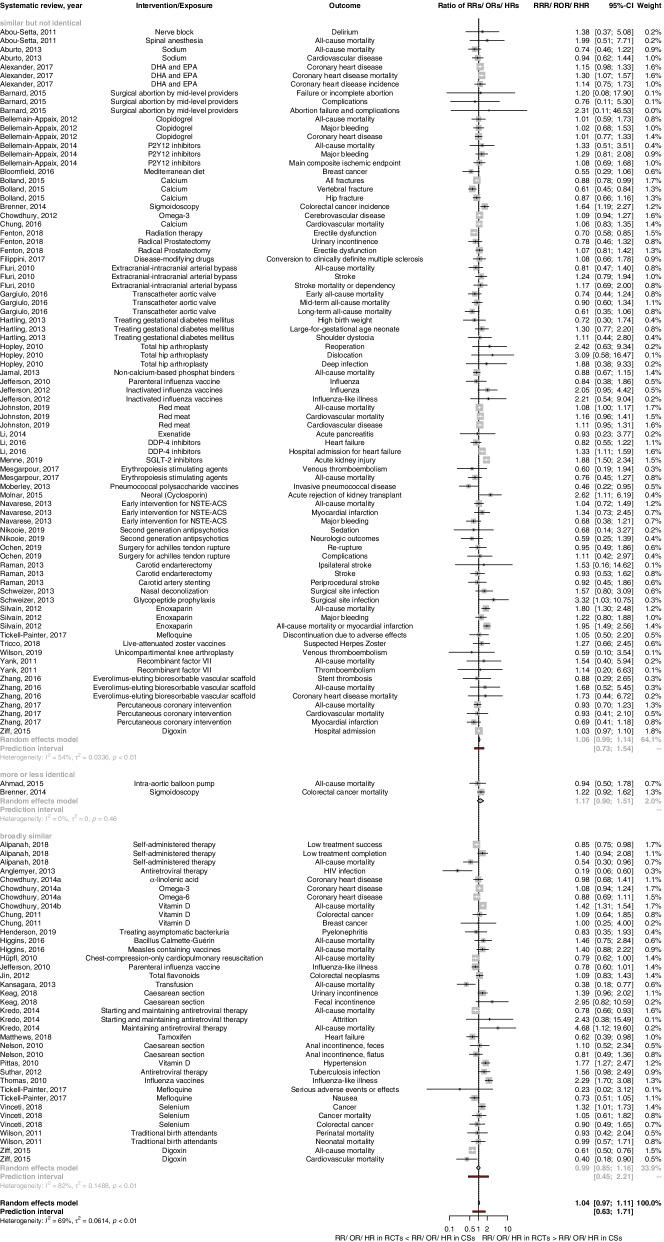
Forest plot for binary outcomes, pooled ratio of ratios (RoR) for bodies of evidence from randomized controlled trials vs. cohort studies stratified by overall PI/ECO*-similarity degree. *PI/ECO population, intervention/exposure, comparator, outcome, CSs cohort studies, DDP-4 dipeptidyl peptidase 4, DHA docosahexaenoic acid, EPA eicosapentaenoic acid, HR hazard ratio, NSTE-ACS non-ST elevation acute coronary syndrome, OR odds ratio, RCTs randomized controlled trials, RHR ratio of hazard ratios, ROR ratio of odds ratios, RR risk ratio; RRR ratio of risk ratios, SGLT-2 sodium glucose transporter 2

Analysis by overall PI/ECO-similarity degree of BoE-pairs showed a pooled RoR of 1.17 (95% CI 0.90 to 1.51; *I*^2^=0%; *τ*^2^=0.00; 95%; *n*=2) across “more or less identical,” 1.06 (95% CI 0.99 to 1.14; *I*^2^=54%; *τ*^2^=0.034; 95% PI 0.73 to 1.54; *n*=81) across “similar but not identical,” and 0.99 (95% CI 0.85 to 1.16; *I*^2^=82%; *τ*^2^=0.149; 95% PI 0.45 to 2.21; *n*=37) across “broadly similar” BoE-pairs (Fig. 3 and Table 4). Results of analyses by similarity of each PI/ECO-domain are depicted in Additional file 1 (Fig. S2a-d); in BoE-pairs with “broadly similar” intervention, the pooled RoR indicated the largest disagreement and statistical heterogeneity were highest (RoR: 1.14, 95% CI 0.87 to 1.49; *I*^2^= 86%; *τ*^2^= 0.194; 95% PI 0.42 to 3.08; *n*=15) (Additional file 1: Fig. S2b). Results of multivariable meta-regression by comparing for each PI/ECO-domain the “similar but not identical” to the reference category “more or less identical” among 81 BoE-pairs rated as “similar but not identical” with binary outcomes are as follows: On average, the pooled RoR was changed by the factor 1.14 for populations, 0.89 for interventions, 1.12 for comparators, and 1.02 for outcomes. The results of the meta-regression were not statistically significant (Table 5).

**Table 5:** Multivariable meta-regression for each PI/ECO-domain across body of evidence-pairs with binary outcomes within the category “similar but not identical”

PI/ECO	Estimate	95 % CI	*P* − *value*
Intercept	0.98	(0.74, 1.29)	0.8610
Population^a^	1.14	(0.88, 1.49)	0.3177
Intervention/Exposure^a^	0.89	(0.72, 1.11)	0.3060
Comparator^a^	1.12	(0.91, 1.37)	0.2749
Outcome^a^	1.02	(0.61, 1.72)	0.9361
Heterogeneity: *τ*^2^ = 0.00, *Q* = 34.35, *p* − *value* = 1			

*PI/ECO* Population, intervention/ exposure, comparator, outcome

^a^Results for the category “similar but not identical” with the reference category “more or less identical”

Our analyses stratified by type of intervention showed the following: The pooled RoR was 1.04 (95% CI 0.89 to 1.21; *I*^2^= 76%; *τ*^2^= 0.139; 95% PI 0.48 to 2.24; *n*=40) for drugs, 1.00 (95% CI 0.91 to 1.10; *I*^2^= 25%; *τ*^2^= 0.011; 95% PI 0.79 to 1.26; *n*=39) for invasive procedures, 1.07 (95% CI 0.98 to 1.16; *I*^2^= 71%; *τ*^2^= 0.023; 95% PI 0.77 to 1.48; *n*=28) for nutrition-interventions, 1.24 (95% CI 0.87 to 1.75; *I*^2^= 80%; *τ*^2^= 0.177; 95% PI 0.42 to 3.63; *n*=9) for vaccines, 0.97 (95% CI 0.62 to 1.52; *I*^2^= 0%; *τ*^2^= 0; *n*=2) for birth assistance, 0.38 (95% CI 0.18 to 0.77; *n*=1) for blood transfusion, and 0.79 (95% CI 0.62 to 1.00; *n*=1) for cardiopulmonary resuscitation (Table 4, Additional file 1: Fig. S3). Exploratory analyses with stratification by PI/ECO-similarity degree within subgroups of interventions (Additional file 1: Fig. S3a-e) showed disagreement between both BoE for drugs with divergence between BoE-pairs rated as “broadly similar” (RoR: 0.79, 95% CI 0.56 to 1.11; *I*^2^= 69%; *τ*^2^=0.290; 95% PI 0.23 to 2.71; *n*=14) and BoE-pairs rated as “similar but not identical” (RoR: 1.20, 95% CI 1.05 to 1.37; *I*^2^=67%; *τ*^2^=0.050; 95% PI 0.74 to 1.94; *n*=26) (Additional file 1: Fig. S3b). For “broadly similar” BoE pairs from nutrition research, differences in effect estimates between both BoE were observed (RoR: 1.17, 95% CI 1.03 to 1.33; *n*=11) (Additional file 1: Fig. S3c). Exploratory analysis excluding BoE-pairs evaluating effects of vitamin D or calcium (*n*=8) resulted in estimates that were more in agreement (RoR: 1.09, 95% CI 1.04 to 1.14; *I*^2^=0%; *τ*^2^=0.00; 95% PI 1.04 to 1.15; *n*=20) and statistical heterogeneity disappeared (Additional file 1: Fig. S4). Analysis of BoE pairs evaluating vaccines indicated a higher extend of disagreement for “broadly similar” BoE-pairs (RoR: 1.37, 95% CI 0.86 to 2.17; *I*^2^=90%; *τ*^2^=0.177; 95% PI 0.17 to 10.88; *n*=4) compared to “similar but not identical” BoE-pairs (RoR: 1.09, 95% CI 0.62 to 1.92; *I*^2^=58%; *τ*^2^=0.177; 95% PI 0.19 to 6.45; *n*=5) (Additional file 1: Fig. S3d).

Stratified analyses by outcome-category are shown in Additional file 1 (Fig. S5) and Table 4. The pooled RoR was 0.94 (95% CI 0.82 to 1.09; *I*^2^=80%; *τ*^2^=0.075; 95% PI 0.53 to 1.69; *n*=28) for BoE pairs reporting all-cause mortality, 1.12 (95% CI 1.02 to 1.23; *I*^2^=43%; *τ*^2^=0.022; 95% PI 0.81 to 1.55; *n*=26) for CVD outcomes, and 1.06 (95% CI 0.89 to 1.26; *I*^2^=67%; *τ*^2^=0.068; 95% PI 0.60 to 1.90; *n*=20) for drug safety outcomes.

The results of the sensitivity analysis where only one outcome (with the largest number of RCTs) was chosen from each systematic review confirmed findings from the main analysis (RoR: 1.08, 95% CI 0.97 to 1.20; *I*^2^=76%; *τ*^2^=0.097; 95% PI 0.57 to 2.03; *n*=60) (Additional file 1: Fig. S6). Sensitivity analysis by direction of effect yielded a pooled RoR of 1.18 (95% CI 1.10 to 1.27; *I*^2^=61%; *τ*^2^=0.046; 95% PI 0.77 to 1.82; *n*=79) and 0.81 (95% CI 0.76 to 0.87; *I*^2^=16%; *τ*^2^=0.005; 95% PI 0.69 to 0.95; *n*=41) for BoE pairs where the cohort study effect estimate was <1 and ≥1, respectively (Additional file 1: Fig. S7).

## Discussion

### Summary of findings

This large meta-epidemiological study identified and compared empirical data investigating the same medical research question to determine the extent to which estimates of BoE from RCTs and cohort studies are in agreement. Overall, 129 BoE pairs derived from 64 systematic reviews were enclosed for the analyses. Only two BoE pairs were rated as “more or less identical” according to PI/ECO-similarity. For binary outcomes, the pooled RoR showed that on average, the extent of deviations towards larger and smaller effect estimates in BoE from RCTs versus cohort studies was almost identical. Differences of effect estimates between the two BoE for continuous outcomes were mostly small. Subgroup analyses by intervention type, type of effect measure, and outcome category showed that on average, there was a little indication for overall differences between both BoE (with the exception of subgroups for ORs and CVD outcomes). Even though the pooled RoR showed that on average effect estimates did not differ, this does not preclude important differences in individual comparisons and/or studies.

Pooling RoRs from BoE-pairs with pharmacological interventions resulted in high statistical heterogeneity. The pooled RoR was similar to the main analysis in BoE pairs with a higher and lower degree of PI/ECO-similarity between both BoE. However, when pooling RoRs, statistical heterogeneity was highest across BoE pairs with the most dissimilar PI/ECO and PIs were substantially wider. Analysis of the pooled RoR by direction of effect in cohort studies indicated differences between both study types. Post hoc analyses revealed that statistical heterogeneity was higher across meta-analyses from “broadly similar” than “similar but not identical” BoE pairs, and higher across cohort studies compared to RCTs.

### Comparison with other studies

#### General medical field

The Cochrane review by Anglemyer et al. [18] evaluated the agreement of effect estimates between RCTs and observational studies in a sample of methodological reviews. Across nine reviews with specific estimates for RCTs versus cohort studies, they computed a pooled RoR of 1.04 (95% CI 0.89 to 1.21), which was nearly identical to our pooled RoR of 1.04 (95% CI 0.97 to 1.11). In the RCT versus cohort analysis, the overall difference of effect estimates was small for seven from nine studies; two studies [101, 102] showed discordance in different directions with a RoR of 0.71 and 3.58, respectively. Anglemyer et al. [18] concluded that on average, the difference of effect estimates between observational studies and RCTs is negligible and proposed that future work should explore other factors than the study design only that could explain occurring differences of effect estimates. In contrast to Anglemyer et al. [18], we performed more detailed data extraction, investigated PI/ECO-similarity degree, and calculated PIs. This allowed us to better understand potential differences. We evaluated statistical heterogeneity on different levels and showed that across the included meta-analyses as well as within the pooled RoR, median statistical heterogeneity and PI were highest across PI/ECO-dissimilar BoE-pairs, and higher across cohort studies compared to RCTs. Further, analysis by each PI/ECO-domain showed that differences of interventions were the main drivers towards disagreement; within the category “similar but not identical,” meta-regression showed that the average effects on the pooled RoR resulting from differences in populations, interventions, and comparators were comparably large, albeit not statistically significant.

#### Other research fields

Hong et al. [103] conducted a meta-epidemiological study comparing 74 pairs of summary effect estimates from RCTs and observational studies in the field of pharmacology. On average, differences were small albeit with considerable between-study variability, which is in line with our findings. Anglemyer et al. [18] showed differences between RCTs and all observational BoE for pharmacological studies (RoR: 1.17, 95% CI 0.95 to 1.43). In contrast, in our analysis, the pooled RoR for pharmacological BoE pairs was similar to the main analysis (RoR: 1.04, 95% CI 0.89 to 1.21). However, in stratified analyses, PI/ECO-similarity degree was an important driver for discordance across pharmacological BoE pairs: for “similar but not identical” BoE-pairs, the RoR was 1.20 and for “broadly similar” BoE-pairs, the RoR was 0.79, with considerable statistical heterogeneity (*I*^2^=67% and 69%, respectively). We found important differences of interventions in “broadly similar” BoE pairs; For example, early interventions at high CD4-cell counts with antiretroviral therapy in RCTs may prevent human immunodeficiency virus infection more likely compared to interventions at various disease stages in cohort studies [77]. Also, exposure to digoxin after myocardial infarction (MI) can increase mortality whereas in chronic heart failure (CHF) with sinus rhythm the effect on mortality is known to be more neutral [104, 105]. Hence, RCTs can show lower mortality when including populations with CHF and sinus rhythm than cohort studies that include MI survivors [96]. From BoE pairs rated as “similar but not identical,” many were from the cardiovascular field [40, 47, 48, 53, 96]. Both, BoE from RCTs and cohort studies often included mixed populations with acute and non-acute CVD [40, 47, 48]; this drives PI/ECO-dissimilarity and may increase statistical heterogeneity. A recent meta-epidemiological study has shown that differences in effect estimates between nutrition RCTs and cohort studies were mainly driven by dissimilarities in population, intervention or exposure, comparator, and outcome [20]. Franklin et al. [106] emulated ten selected pharmacological RCTs using observational data sets. For nine included RCT emulations, differences of effect estimates were within the range of random variation. Disagreement was largest in comparisons with active comparators in observational data and placebo in RCTs. The authors conclude that similar active comparators in RCTs, and observational studies increase the probability of agreement and stressed that different methods have a substantial impact on the finding of agreement.

### Potential implications

RCTs are considered the gold standard to evaluate causal inference for medical interventions [1–3]. Due to a variety of reasons such as low external validity [7, 9] and limited availability of RCTs [5], health care professionals and other decision-makers increasingly rely on results from observational studies. However, results from RCTs and observational studies can differ [15, 18, 107] and efforts to understand under which circumstances this occurs are ongoing [106]. Our study provides valuable insights into the field of general and internal medicine, but also into other important research fields such as public health. We showed that BoE from RCTs and cohort studies included in systematic reviews from high-impact factor medical journals often differ in terms of study populations (e.g., different disease status), interventions and comparators (e.g., different intervention-timing, different drugs of the same class), or outcomes (e.g., late-stage disease versus any disease). Our data highlight the importance of PI/ECO-differences—especially those of interventions—in explaining differences of effect estimates. As a perspective, evaluating differences in factors such as study size, follow-up time, or publication date may serve to further explore disagreement between the two study design types. However, other factors require equal attention. Appropriate adjustment for confounding is a necessary precondition to consider results from observational studies and residual confounding remains a major concern [108]. To deal with these uncertainties evaluating the risk of bias is of tremendous importance to assess the trustworthiness of findings. In our sample, the Cochrane risk of bias tool [97] for RCTs and the Newcastle Ottawa scale (NOS) [98] for cohort studies were mainly used, along with a variety of other instruments to rate the risk of bias/study quality. We assume that the increased use of the ROBINS-I tool [109] may facilitate integrating both BoE in evidence syntheses and facilitate analyses by the risk of bias and certainty of the evidence in methodological studies. The ROBINS-I tool is based on the target trial approach [110] and permits to better compare evidence from RCTs and observational studies. This will be useful to investigate the influence of bias on differences between findings from RCTs and cohort studies. In general, cohort studies may serve as a source for complementary or sequential information, or even replace findings from RCTs [11]. In evidence synthesis, cohort studies are sometimes included as a complementary source of evidence to increase the precision and/or generalizability of findings [12]. However, caution is warranted when pooling both BoE since, as shown in our study, PI/ECO-differences are common between both BoE, and cohort studies showed higher statistical heterogeneity.

### Strengths and limitations

Our study has several strengths: First, a large sample of BoE-pairs (*n*=129) derived from 64 systematic reviews with a high number of RCTs and cohort studies were included. BoE pairs investigated a broad range of medical topics from high-impact factor medical journals. Second, extensive data extraction, including a detailed description of the population, intervention, comparator, outcome, risk of bias ratings, and length of follow-up conducted by two reviewers independently allowed us to rigorously explore the clinical- and design features of the included BoE. Third, our analysis included an evaluation of agreement of effect estimates across the included BoE-pairs for binary and also continuous effect estimates. We stratified the analyses by type of binary effect measure, intervention-type, and outcome category. For the first time in the general medical field, we implemented an approach that allowed us to explore the influence of PI/ECO-differences on the disagreement of effect estimates.

Several limitations should be considered as well: First, meta-epidemiologic studies such as ours are based on an observational analysis and therefore show only non-causal associations [111, 112]. Factors such as publication date can act as meta-confounders. Further, we did not take into account the risk of bias/study quality and certainty of the evidence into the quantitative analysis, since the tools used by the systematic review authors were highly heterogeneous and often the corresponding information was not reported sufficiently in the systematic reviews. However, bias was assessed as follows in our sample: we showed that on average the effect estimates were in agreement (as shown by the pooled RoR) making systematic bias towards smaller or larger effect estimates unlikely. Potential bias may also exist in individual BoE pairs and influence the RoRs additionally to PI/ECO-differences. However, we showed that PI/ECO-dissimilarities were important drivers of statistical heterogeneity and wide PIs. Further, bias may affect individual cohort studies causing higher statistical heterogeneity in meta-analyses [13]. Accordingly, in our sample statistical heterogeneity in meta-analyses of cohort studies (median: *I*^2^= 46%) was higher than in meta-analyses of RCTs (median: *I*^2^= 8%). We did not explore whether disagreement was larger between RCTs compared to prospective and retrospective cohort studies, respectively. The corresponding information was reported in a suboptimal manner, and researchers may use inconsistent nomenclature [113, 114]. Second, we did not evaluate the methodological quality of the included systematic reviews, but given that we focused on high-impact journals, we assumed that published systematic reviews are of reasonably high methodological quality. Third, even though rating the degree of PI/ECO-similarity was performed by two reviewers using predefined criteria, this process is still partly subjective, and ratings may be too strict since only two BoE were judged as “more or less identical.” Further, PI/ECO-dissimilarities in BoE pairs were usually present in more than one PI/ECO-domain; this complicates drawing conclusions about the difference of effect estimates that results from a given PI/ECO-dissimilarity in one domain (e.g., from a difference of interventions). Fourth, performing several subgroup analyses might increase the likelihood of findings by chance. However, most of these analyses did not find any subgroup differences, thereby increasing our confidence in the findings of the main analysis. Further, with the exception of analysis by PI/ECO-similarity degree and intervention type, subgroup analyses were performed post hoc. However, analyses by type of effect estimate and outcome category were planned before the main analysis was conducted. Fifth, some degree of overlap between BoE cannot be ruled out since some primary studies contributed to more than one included BoE. This might have increased the precision of our findings. However, a sensitivity analysis of only one outcome per systematic review showed similar findings to the main analysis. Sixth, with regard to the search strategy, choosing another time frame may yield different results; however, we chose the dates to cover a 10-year period (January 01, 2010, to December 31, 2019). Further, the restriction on BoE pairs from the same systematic review may limit the representativeness of the sample. However, the main alternative, i.e., the inclusion of BoE from matched systematic reviews from RCTs and cohort studies, may have other drawbacks, such as impaired comparability of systematic review methodology.

## Conclusions

On average the pooled effect estimates between RCTs and cohort studies did not differ. Statistical heterogeneity and wide PIs were mainly driven by PI/ECO-dissimilarities (i.e., clinical heterogeneity) and cohort studies. Differences of interventions were the main drivers towards disagreement; however, when focusing on “similar but not identical” BoE-pairs (i.e., with at least moderate similarity), the similarity degree categories (“similar but not identical,” “more or less identical”) affected more the average effect in populations, interventions, or comparators compared to the outcome albeit not statistically significant. The quantitative analysis did not assess how the risk of bias and certainty of the evidence influenced disagreement in addition to PI/ECO-dissimilarities. Upcoming meta-epidemiological studies may further explore the impact of risk of bias, certainty of the evidence, and residual confounding on differences of effect estimates between RCTs and cohort studies.

## Supplementary Information

**Additional file 1: Appendix S1.** Search strategy for systematic reviews. **Figure S1.** Flow diagram, identification of systematic reviews. **Table S1.** Criteria for rating PI/ECO-similarity degree. **Table S2.** Transformations made to the original data extraction. **Table S3.** Reasons for exclusion of systematic reviews. **Table S4.** Characteristics of included BoE from RCTs. **Table S5.** Certainty of the evidence and risk of bias for BoE from RCTs. **Table S6.** Characteristics of BoE from cohort studies. **Table S7.** Risk of bias and certainty of the evidence for BoE from cohort studies. **Table S8.** Heat map: instruments used for the assessment of risk of bias for BoE from RCTs and cohort studies. **Table S9.** Ratings of PI/ECO-similarity degree for included BoE-pairs. **Table S10.** Effect estimates and statistical heterogeneity for meta-analyses of RCTs and cohort studies. **Figure S2a.** Forest plot, analysis by population similarity degree. **Figure S2b.** Forest plot, analysis by intervention/ exposure similarity degree. **Figure S2c.** Forest plot, analysis by comparator similarity degree. **Figure S2d.** Forest plot, analysis by outcome similarity degree. **Figure S3.** Forest plot, analysis by intervention-type. **Figure S3a.** Forest plot, analysis of invasive procedures, stratified by PI/ECO-similarity degree. **Figure S3b.** Forest plot, analysis of drugs as intervention, stratified by PI/ECO-similarity degree. **Figure S3c.** Forest plot, analysis of nutrition as intervention, stratified by PI/ECO-similarity degree. **Figure S3d.** Forest plot, analysis of vaccines as intervention, stratified by PI/ECO-similarity degree. **Figure S3e.** Forest plot, analysis of birth assistance as intervention, stratified by PI/ECO-similarity degree. **Figure S4.** Forest plot, analysis of nutrition as intervention: Vitamin D/ Calcium as intervention vs. other nutrition-interventions. **Figure S5.** Forest plot, analysis by outcome-category. **Figure S6.** Sensitivity analysis: one BoE-pair per systematic review. **Figure S7.** Sensitivity analysis by direction of cohort study summary effect estimate.

## Data Availability

Data are based on published meta-analyses.

## Abbreviations

ACR: Assumed control risk
BoE: Body of evidence
CHF: Chronic heart failure
CI: Confidence interval
CSs: Cohort studies
CVD: Cardiovascular disease
DDP-4: Dipeptidyl peptidase 4
DHA: Docosahexaenoic acid
DMD: Difference of mean differences
EPA: Eicosapentaenoic acid
HR: Hazard ratio
LDL: Low-density lipoprotein
MD: Mean difference
MI: Myocardial infarction
NRSI: Non-randomized studies of interventions
NSTE-ACS: Non-ST elevation acute coronary syndrome
OR: Odds ratio
PI: Prediction interval
PI/ECO: Population, intervention/exposure, comparator, outcome
RCT: Randomized controlled trial
RHR: Ratio of hazard ratios
RR: Risk ratio
RoR: Ratio of ratios
ROR: Ratio of odds ratios
RRR: Ratio of risk ratios
SGLT-2: Sodium-glucose transporter 2
