# Supplementary material for: Evaluating agreement between bodies of evidence from randomized controlled trials and cohort studies in medical research: a meta-epidemiological study
Source: BMC Med. 2022 May 11;20:174. doi: 10.1186/s12916-022-02369-2 (PMC9092682; doi:10.1186/s12916-022-02369-2)
Supplement: Supplementary file 1 — Additional file 1: Appendix S1. Search strategy for systematic reviews. Figure S1. Flow diagram, identification of systematic reviews. Table S1. Criteria for rating PI/ECO-similarity degree. Table S2. Transformations made to the original data extraction. Table S3. Reasons for exclusion of systematic reviews. Table S4. Characteristics of included BoE from RCTs. Table S5. Certainty of the evidence and risk of bias for BoE from RCTs. Table S6. Characteristics of BoE from cohort studies. Table S7. Risk of bias and certainty of the evidence for BoE from cohort studies. Table S8. Heat map: instruments used for the assessment of risk of bias for BoE from RCTs and cohort studies. Table S9. Ratings of PI/ECO-similarity degree for included BoE-pairs. Table S10. Effect estimates and statistical heterogeneity for meta-analyses of RCTs and cohort studies. Figure S2a. Forest plot, analysis by population similarity degree. Figure S2b. Forest plot, analysis by intervention/ exposure similarity degree. Figure S2c. Forest plot, analysis by comparator similarity degree. Figure S2d. Forest plot, analysis by outcome similarity degree. Figure S3. Forest plot, analysis by intervention-type. Figure S3a. Forest plot, analysis of invasive procedures, stratified by PI/ECO-similarity degree. Figure S3b. Forest plot, analysis of drugs as intervention, stratified by PI/ECO-similarity degree. Figure S3c. Forest plot, analysis of nutrition as intervention, stratified by PI/ECO-similarity degree. Figure S3d. Forest plot, analysis of vaccines as intervention, stratified by PI/ECO-similarity degree. Figure S3e. Forest plot, analysis of birth assistance as intervention, stratified by PI/ECO-similarity degree. Figure S4. Forest plot, analysis of nutrition as intervention: Vitamin D/ Calcium as intervention vs. other nutrition-interventions. Figure S5. Forest plot, analysis by outcome-category. Figure S6. Sensitivity analysis: one BoE-pair per systematic review. Figure S7. Sensitivity analysis by direct [file 12916_2022_2369_MOESM1_ESM.pdf]

Additional File 1

Evaluating agreement between bodies of evidence from randomized controlled trials and cohort studies in medical research: a meta-epidemiological study

Nils Bröckelmann[1], Sara Balduzzi[2], Louisa Harms[1], Jessica Beyerbach[1], Maria Petropoulou[2], Charlotte Kubiak[1], Martin Wolkewitz[2], Joerg J Meerpohl[1,3], Lukas Schwingshackl[1]

[1] Institute for Evidence in Medicine, Medical Center - University of Freiburg, Faculty of Medicine, University of Freiburg, Freiburg, Germany.

[2] Institute of Medical Biometry and Statistics, Faculty of Medicine and Medical Center - University of Freiburg, Freiburg, Germany.

[3] Cochrane Germany, Cochrane Germany Foundation, Freiburg, Germany.

Table of contents

Appendix S1. Search strategy for systematic reviews in MEDLINE via PubMed (search date: 04.05.2020)........ 2

Figure S1. Flow diagram showing the process for identifying eligible systematic reviews 3

Table S1. Criteria for rating PI/ECO*-similarity degree between bodies of evidence from randomized controlled trials and cohort studies // *PI/ECO= population, intervention/ exposure, comparator, outcome 4

Table S2. Overview of transformations made to the original data extraction 6

Table S3. Reasons for exclusion of systematic reviews and corresponding articles in alphabetic order 8

Table S4: Characteristics of included bodies of evidence from randomized controlled trials 14

Table S5. Certainty of the evidence and risk of bias for included bodies of evidence from randomized controlled trials 49

Table S6. Characteristics of included bodies of evidence from cohort studies 64

Table S7. Risk of bias and certainty of the evidence for included bodies of evidence from cohort studies 93

Table S8. Overview of the instruments used for the assessment of risk of bias for bodies of evidence of RCTs and cohort studies, heat map 107

Table S9. Ratings of PI/ECO*-similarity degree for included body of evidence-pairs // *PI/ECO= population, intervention/ exposure, comparator, outcome 117

Table S10. Effect estimates and statistical heterogeneity for meta-analyses of RCTs and cohort studies 123

Figures S2a to S7: Forest Plots 131

Appendix S1. Search strategy for systematic reviews in MEDLINE via PubMed (search date: 04.05.2020)	
ID	Search
#1	"lancet london england"[Journal] OR "JAMA"[Journal] OR "bmj clinical research ed"[Journal] OR "jama internal medicine"[Journal] OR "Annals of internal medicine"[Journal] OR "PLoS medicine"[Journal] OR "BMC medicine"[Journal] OR "The Cochrane database of systematic reviews"[Journal] OR "Mayo Clinic proceedings"[Journal] OR "Canadian Medical Association journal"[Journal] OR "Nat Rev Dis Primers"[Journal] OR "J Cachexia Sarcopenia Muscle"[Journal] OR "N Engl J Med"[Journal]
#2	"systematic review"[Title/Abstract]) OR "systematic literature review"[Title/Abstract] OR "systematic scoping review"[Title/Abstract] OR "systematic meta-review"[Title/Abstract] OR "systematic search"[Title/Abstract] OR "systematic review"[Publication Type] OR "meta analys*"[Title/Abstract] OR "meta analys*"[Publication Type] OR "cochrane database syst rev"[Journal]
#3	"random*"[Title/Abstract] OR "placebo"[Title/Abstract] OR "clinical trials as topic"[MeSH Terms:noexp] OR "trial"[Title]
#4	"epidemiolog*"[Title/Abstract] OR "cohort stud*"[Title/Abstract] OR "observation*"[Title/Abstract] OR "non rct*"[Title/Abstract] OR "non random*"[Title/Abstract]
#5	#1 AND #2 AND #3 AND #4
#6	#1 AND #2 AND #3 AND #4 Filters: from 2010/1/1 - 2019/12/31

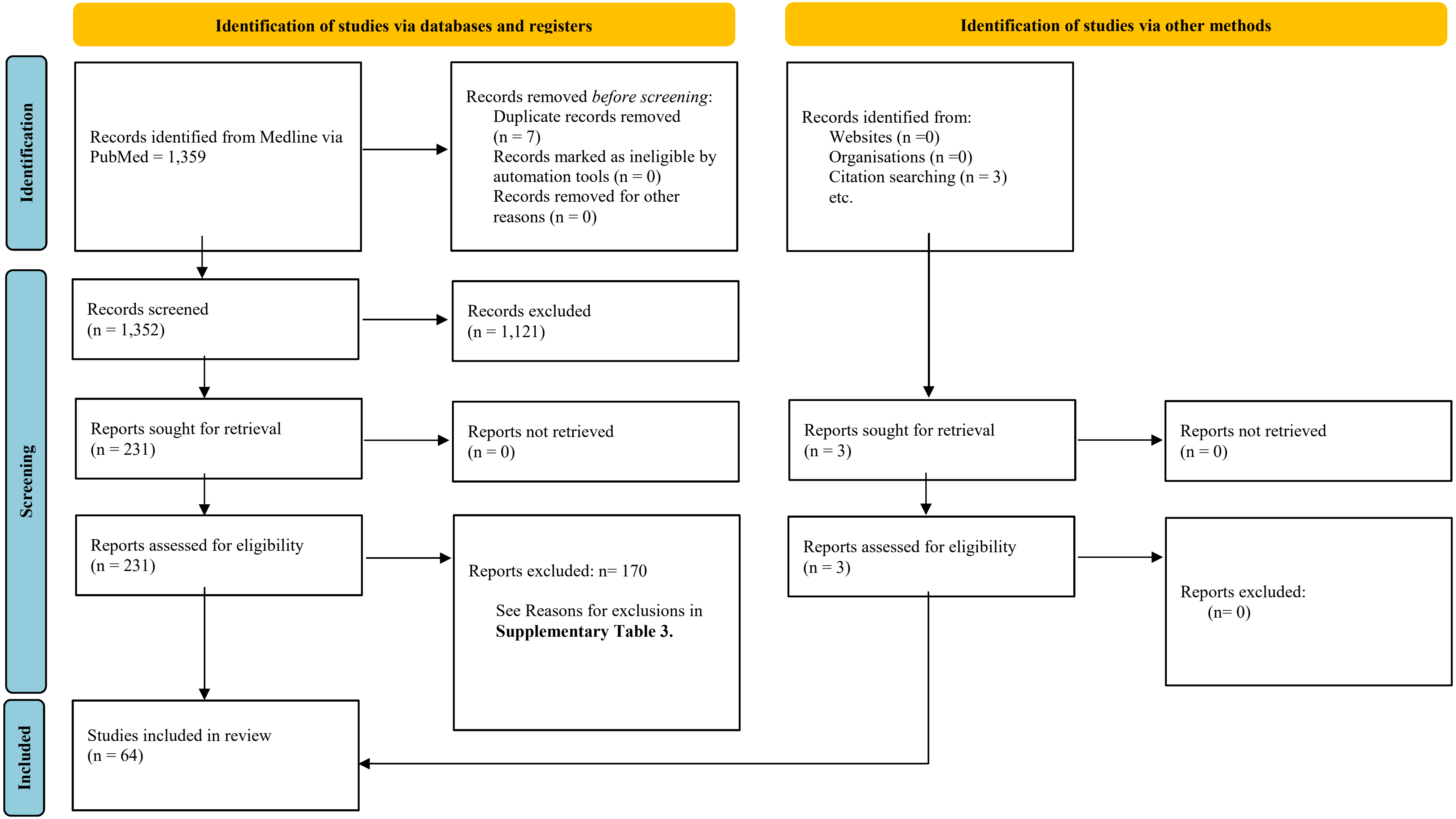

Figure S1. Flow diagram showing the process for identifying eligible systematic reviews

Table S1. Criteria for rating PI/ECO*-similarity degree between bodies of evidence from randomized controlled trials and cohort studies // *PI/ECO= population, intervention/ exposure, comparator, outcome

Rating	Population	Intervention/Exposure	Comparator	Outcome
**1 = “more or less identical”**	Same health status and type of population	Same drug, invasive procedure, nutrition-intervention or vaccine	-Same drug or invasive procedure<br>-Nutrition: Placebo vs. Nil or low intake; low intake vs. low intake	Same outcome
	e.g.<br>-Both BoE with either healthy population, general population or diseased population<br>-Same age category (both adults, both postmenopausal women)	e.g.<br>- Both Enoxaparin<br>- Both PCI<br>- Both high dairy-intake	e.g.<br>-Both no Enoxaparin<br>-Both UKA<br>-Placebo vs. No intervention or low intake	e.g.<br>Mortality in both BoE
**2 = “similar but not identical”**	Populations with mixed health status in RCTs and/ or cohort studies	-Different drugs of the same class/ Any drug of the same class vs. specific drug of the same class<br>-Similar invasive procedure/ same invasive procedure with different co-interventions<br>-Similar vaccines or identical vaccine with different route of administration<br>-Supplementary or free food vs. Intake<br>-Similar but not identical time frame of intervention	-Different drugs of the same class<br>-Similar invasive procedures, drug, vaccine or diet<br>-General dietary advice vs. High intake	-Similar outcome<br>-Both with mixed similar outcomes
	e.g.<br>-Merged healthy and diseased population in one BoEvs. healthy population in the other BoE<br>-Both BoE with merged healthy and diseased population<br>-Population with cardiovascular risk factors (without manifest disease) vs. healthy population	e.g.<br>-Both different SGAs<br>-Both similar regional anaesthetic nerve blocks<br>-Various pneumococcal vaccines (2,3,12,14,17 and 23-valent) versus 23-valent only<br>-One or two doses of measles containing vaccines versus unclear number of doses<br>-Free non-caloric beverages vs. Low intake of SSBs<br>-Early intervention with different time frame (first 14 vs. First 24 hours)	e.g.<br>-Both different DDP-4 inhibitors<br>-“Best medical treatment” with Aspirin and additionally with various other drugs<br>-No vaccination or delayed vaccination versus no vaccination<br>-Transfemoral vs. transapical TAVI<br>-General dietary advice vs. High red meat intake	e.g.<br>-Late stage only or all CRC vs. All CRC<br>-Both with mixed sedation outcomes (e.g. Sleepiness, sedation)

**3 = "broadly similar"**	-Different health status of populations in RCTs and cohort studies<br>-Other substantial differences (e.g. Age-category, type of population)	-Same drug for different indication<br>-Enhanced treatment vs. Any treatment<br>-Supplement vs. Status<br>-Different time frame/ early treatment vs. Any treatment	-Active intervention (drug, invasive procedure, nutrient) vs. No intervention or placebo<br>-Different time frame	-Broadly similar
	e.g.<br>-Healthy population in one BoE vs. population with cardiovascular disease in the other BoE<br>-Children/ adolescents vs. Adults<br>-Travellers vs. Pregnant women	e.g.<br>-Digoxin for HF vs. digoxin post-myocardial infarction without HF<br>-Dispatcher-assisted bystander CPR vs. Unassisted bystander CPR<br>-Enhanced training of birth attendants vs. any support by birth attendant<br>-Selenium supplements vs. High selenium status<br>-Early ART vs. Any ART	e.g.<br>-Restrictive transfusion vs. No transfusion<br>-Placebo vs. Low selenium status<br>-No vaccination of health care workers vs. low share of vaccinated health care workers per facility<br>-Pregnant women with untreated bacteriuria vs. pregnant without screening for bacteriuria<br>-Delayed ART vs. No ART	e.g.<br>-Colorectal adenoma vs. Cancer

ART= antiretroviral therapy; BoE= bodies of evidence; CPR= cardiopulmonary resuscitation; CRC= colorectal cancer; DDP-4= dipeptidyl peptidase 4; HF= heart failure; PCI= percutaneous coronary intervention; PI/ECO= population, intervention/ exposure, comparator, outcome; RCT= randomized controlled trial; SGA= second-generation antipsychotic; SSBs= sugar-sweetened beverages; TAVI= transcatheter aortic valve replacement; UKA= unicompartimental knee arthroplasty.

Table S2. Overview of transformations made to the original data extraction

Systematic review	Outcome	Type of BoE (RCTs/ cohort studies)	n (studies)	Original		What we used		Rationale
				**HR**	**95% CI**	**RR**	**95% CI**	
Bloomfield 2016 (22)	Breast cancer	RCT	1	0.43	0.21, 0.88	0.53	0.28, 1.03	Number of patients and events in intervention and control group, 4.8 years median follow-up; Table 2. in Toledo 2015 (62)
Chung 2016 (56)	Cardiovascular mortality	cohort studies	6	0.99	0.97, 1.01	0.99	0.97, 1.01	Data to calculate a RR for the HR were not available in the systematic review
Johnston 2019 (23)	Mortality	RCT	1	0.99	0.95, 1.03	0.94	0.89, 0.99	Number of patients and events in intervention and control group follow-up 9/30/2010; Supplementary table 2 in Thomson 2014 (63)
Johnston 2019 (23)	Cardiovascular mortality	RCT	1	0.98	0.91, 1.06	1.00	0.84, 1.19	Number of patients and events in intervention and control group for coronary heart disease death and fatal stroke, mean 8.1 years follow-up; Table 4 in Howard 2006 (64)
Johnston 2019 (23)	Cardiovascular disease	RCT	1	0.99	0.94, 1.05	0.97	0.91, 1.04	Number of patients and events in intervention and control group, mean 8.1 years follow-up; Table 4 in Howard 2006 (64)
Pittas 2010 (60)	Hypertension	RCT	1	1.01	0.96, 1.06	1.01	0.97, 1.05	Number of patients and events in intervention and control group, mean follow-up 7 years; Figure 2 in Margolis 2008 (65)
				**OR**	**95% CI**	**RR**	**95% CI**	
Chung 2011(58)	Colorectal cancer	cohort studies	9	0.94	0.91, 0.97	0.94	0.91, 0.97	ACR= 0.0201; calculated as control group risk of the corresponding RCT Trivedi 2003 (66)
Chung 2011 (58)	Breast cancer	cohort studies	4	0.99	0.97, 1.01	0.99	0.97, 1.01	ACR= 0.0124; calculated as control group risk of the corresponding RCT Trivedi 2003 (66)
Kansagara 2013 (52)	Mortality	cohort studies	11	*	*	2.49	1.40, 4.43	Matching cohort studies (n=11) reporting 30-day mortality for blood transfusions (Supplemental Table 8) were included; ORs (n=5) were converted to RRs using the ACR= 0.0817 calculated as median control group risk from the included RCTs in Figure 1; data to convert HRs for (n=6) cohort studies were not available in the systematic review (52)
Li 2014 (54)	Acute pancreatitis	RCT	5	0.86	0.22, 3.39	0.86	0.22, 3.37	ACR= 0.0020; calculated as the median control group risk of the included RCTs in Table 2 (54)
		cohort studies	2	*	*	0.92	0.69, 1.22	Matching cohort studies (n=2) from Table 4 were included; The OR (n=1) was converted to a RR using the ACR= 0.0020 calculated as the median control group risk of the included RCTs in Table 2; data to convert the

								HR (n=1) were not available in the systematic review (54)
Li 2016 (53)	Heart failure	cohort studies	4	*	*	1.10	1.04, 1.16	Matching cohort studies (n=4) from Table 5/6 were included; ORs (n=3) were converted to RRs using the ACR= 0.0016 calculated as the median control group risk of the included RCTs in Appendix Figure E; data to convert one HR (n=1) were not available in the systematic review (53)
Vinceti 2018 (59)	Any cancer	cohort studies	7	0.72	0.55, 0.93	0.75	0.59, 0.94	ACR= 0.1509; calculated as the median control group risk of the corresponding RCTs in Analysis 1.1. Comparison 1 (59)
Vinceti 2018 (59)	Cancer mortality	cohort studies	7	0.76	0.59, 0.97	0.77	0.60, 0.97	ACR= 0.0597; calculated as the median control group risk of the corresponding RCTs Analysis 1.2. Comparison 1 (59)
Vinceti 2018 (59)	Colorectal cancer	cohort studies	6	0.82	0.72, 0.94	0.82	0.72, 0.94	ACR= 0.0077; calculated as the median control group risk of the corresponding RCTs Analysis 1.5. Comparison 1 (59)
				**RD**	**95% CI**	**RR**	**95% CI**	
Yank 2011 (44)	Mortality	RCTs	2	0.01	-0.05, 0.06	1.40	0.49, 4.02	Common meta-analysis for RCTs and cohort studies using a Risk difference was separated and effect measures converted to RRs using data in Figure 2 & 3 (44)
		cohort studies	2			0.91	0.39, 2.12	
Yank 2011 (44)	Thromboembolic events	RCTs	2	0.05	0.01, 0.10	2.06	0.48, 8.84	
		cohort studies	2			1.81	0.67, 4.87	
				**SMD**	**95% CI**	**MD**	**95% CI**	
Te Morenga 2013 (61)	BMI	RCTs	3	0.09	-0.14, 0.32	-0.06	-0.15, 0.04	Meta-analysis of RCTs (n=5) shown in Figure 6 using SMD; we included only RCTs reporting the outcome BMI (n=3)

*studies were not pooled in the original publication; ACR= assumed control risk; HR= hazard ratio; MD= mean difference; OR= odds ratio; RCT= randomized controlled trial; RR= risk ratio; SMD= standardized mean difference.

Table S3. Reasons for exclusion of systematic reviews and corresponding articles in alphabetic order

Methods of systematic reviews: Excluded RCTs or cohort studies, searches not equivalent for RCTs and cohort studies, no meta-analysis, evaluation of diagnostic accuracy, individual patient data analysis; n=56 articles
– Brodie D, Slutsky AS, Combes A. Extracorporeal Life Support for Adults With Respiratory Failure and Related Indications: A Review. Jama. 2019;322(6):557-68.<br>– Baker PR, Francis DP, Soares J, Weightman AL, Foster C. Community wide interventions for increasing physical activity. Cochrane Database Syst Rev. 2011(4):Cd008366.<br>– Baker PR, Francis DP, Soares J, Weightman AL, Foster C. Community wide interventions for increasing physical activity. Cochrane Database Syst Rev. 2015;1:Cd008366.<br>– Bannuru RR, Dvorak T, Obadan N, Yu WW, Patel K, Chung M, et al. Comparative evaluation of radiation treatments for clinically localized prostate cancer: an updated systematic review. Ann Intern Med. 2011;155(3):171-8.<br>– Borst SE, Shuster JJ, Zou B, Ye F, Jia H, Wokhlu A, et al. Cardiovascular risks and elevation of serum DHT vary by route of testosterone administration: a systematic review and meta-analysis. BMC Med. 2014;12:211.<br>– Bourke JP, Bueser T, Quinlivan R. Interventions for preventing and treating cardiac complications in Duchenne and Becker muscular dystrophy and X-linked dilated cardiomyopathy. Cochrane Database Syst Rev. 2018;10:Cd009068.<br>– Carter JL, Coletti RJ, Harris RP. Quantifying and monitoring overdiagnosis in cancer screening: a systematic review of methods. Bmj. 2015;350:g7773.<br>– Chingcuanco F, Segal JB, Kim SC, Alexander GC. Bioequivalence of Biosimilar Tumor Necrosis Factor-alpha Inhibitors Compared With Their Reference Biologics: A Systematic Review. Ann Intern Med. 2016;165(8):565-74.<br>– Chou R, Cantor AG, Zakher B, Bougatsos C. Screening for HIV in pregnant women: systematic review to update the 2005 U.S. Preventive Services Task Force recommendation. Ann Intern Med. 2012;157(10):719-28.<br>– Chou R, Selph S, Dana T, Bougatsos C, Zakher B, Blazina I, et al. Screening for HIV: systematic review to update the 2005 U.S. Preventive Services Task Force recommendation. Ann Intern Med. 2012;157(10):706-18.<br>– Chou R, Dana T, Bougatsos C, Blazina I, Starmer AJ, Reitel K, et al. Pressure ulcer risk assessment and prevention: a systematic comparative effectiveness review. Ann Intern Med. 2013;159(1):28-38.<br>– Chou R, Korthuis PT, McCarty D, Coffin PO, Griffin JC, Davis-O'Reilly C, et al. Management of Suspected Opioid Overdose With Naloxone in Out-of-Hospital Settings: A Systematic Review. Ann Intern Med. 2017;167(12):867-75.<br>– Chowdhury R, Ramond A, O'Keeffe LM, Shahzad S, Kunutsor SK, Muka T, et al. Environmental toxic metal contaminants and risk of cardiovascular disease: systematic review and meta-analysis. Bmj. 2018;362:k3310.<br>– Colebatch AN, Marks JL, Edwards CJ. Safety of non-steroidal anti-inflammatory drugs, including aspirin and paracetamol (acetaminophen) in people receiving methotrexate for inflammatory arthritis (rheumatoid arthritis, ankylosing spondylitis, psoriatic arthritis, other spondyloarthritis). Cochrane Database Syst Rev. 2011(11):Cd008872.<br>– Type and timing of menopausal hormone therapy and breast cancer risk: individual participant meta-analysis of the worldwide epidemiological evidence. Lancet. 2019;394(10204):1159-68.<br>– Dahabreh IJ, Chung M, Balk EM, Yu WW, Mathew P, Lau J, et al. Active surveillance in men with localized prostate cancer: a systematic review. Ann Intern Med. 2012;156(8):582-90.<br>– Demicheli V, Jefferson T, Di Pietrantonj C, Ferroni E, Thorning S, Thomas RE, et al. Vaccines for preventing influenza in the elderly. Cochrane Database Syst Rev. 2018;2:Cd004876.<br>– Demicheli V, Jefferson T, Ferroni E, Rivetti A, Di Pietrantonj C. Vaccines for preventing influenza in healthy adults. Cochrane Database Syst Rev. 2018;2:Cd001269.<br>– Des Guetz G, Uzzan B, Morere JF, Perret G, Nicolas P. Duration of adjuvant chemotherapy for patients with non-metastatic colorectal cancer. Cochrane Database Syst Rev. 2010(1):Cd007046.<br>– Drekonja DM, Butler M, MacDonald R, Bliss D, Filice GA, Rector TS, et al. Comparative effectiveness of Clostridium difficile treatments: a systematic review. Ann Intern Med. 2011;155(12):839-47.<br>– Frank JW, Lovejoy TI, Becker WC, Morasco BJ, Koenig CJ, Hoffecker L, et al. Patient Outcomes in Dose Reduction or Discontinuation of Long-Term Opioid Therapy: A Systematic Review. Ann Intern Med. 2017;167(3):181-91.<br>– Goldzweig CL, Orshansky G, Paige NM, Towfigh AA, Haggstrom DA, Miake-Lye I, et al. Electronic patient portals: evidence on health outcomes, satisfaction, efficiency, and attitudes: a systematic review. Ann Intern Med. 2013;159(10):677-87.<br>– Harder T, Wichmann O, Klug SJ, van der Sande MAB, Wiese-Posselt M. Efficacy, effectiveness and safety of vaccination against human papillomavirus in males: a systematic review. BMC Med. 2018;16(1):110.<br>– Hollingsworth JM, Rogers MA, Krein SL, Hickner A, Kuhn L, Cheng A, et al. Determining the noninfectious complications of indwelling urethral catheters: a systematic review and meta-analysis. Ann Intern Med. 2013;159(6):401-10.<br>– Humphrey LL, Deffebach M, Pappas M, Baumann C, Artis K, Mitchell JP, et al. Screening for lung cancer with low-dose computed tomography: a systematic review to update the US Preventive services task force recommendation. Ann Intern Med. 2013;159(6):411-20.<br>– Jefferson T, Rivetti A, Di Pietrantonj C, Demicheli V. Vaccines for preventing influenza in healthy children. Cochrane Database Syst Rev. 2018;2:Cd004879.<br>– Jiang B, Chaichana K, Veeravagu A, Chang SD, Black KL, Patil CG. Biopsy versus resection for the management of low-grade gliomas. Cochrane Database Syst Rev. 2017;4:Cd009319.<br>– Johnson SA, Stevens SM, Woller SC, Lake E, Donadini M, Cheng J, et al. Risk of deep vein thrombosis following a single negative whole-leg compression ultrasound: a systematic review and meta-analysis. Jama. 2010;303(5):438-45.<br>– Jullien S, Ryan H, Modi M, Bhatia R. Six months therapy for tuberculous meningitis. Cochrane Database Syst Rev. 2016;9(9):Cd012091.<br>– Kardamanidis K, Martiniuk A, Ivers RQ, Stevenson MR, Thistlethwaite K. Motorcycle rider training for the prevention of road traffic crashes. Cochrane Database Syst Rev. 2010(10):Cd005240.

- Khan F, Rahman A, Carrier M, Kearon C, Weitz JI, Schulman S, et al. Long term risk of symptomatic recurrent venous thromboembolism after discontinuation of anticoagulant treatment for first unprovoked venous thromboembolism event: systematic review and meta-analysis. Bmj. 2019;366:l4363.
- Lee JS, Giesler DL, Gellad WF, Fine MJ. Antibiotic Therapy for Adults Hospitalized With Community-Acquired Pneumonia: A Systematic Review. Jama. 2016;315(6):593-602.
- Leffers N, Daemen T, Helfrich W, Boezen HM, Cohlen BJ, Melief K, et al. Antigen-specific active immunotherapy for ovarian cancer. Cochrane Database Syst Rev. 2010(1):Cd007287.
- Leffers N, Daemen T, Helfrich W, Boezen HM, Cohlen BJ, Melief CJ, et al. Antigen-specific active immunotherapy for ovarian cancer. Cochrane Database Syst Rev. 2014(9):Cd007287.
- Lip GY, Shantsila E. Anticoagulation versus placebo for heart failure in sinus rhythm. Cochrane Database Syst Rev. 2014(3):Cd003336.
- Lopez LM, Bernholc A, Chen M, Grey TW, Otterness C, Westhoff C, et al. Hormonal contraceptives for contraception in overweight or obese women. Cochrane Database Syst Rev. 2016(8):Cd008452.
- Lopez LM, Grey TW, Tolley EE, Chen M. Brief educational strategies for improving contraception use in young people. Cochrane Database Syst Rev. 2016;3:Cd012025.
- Lopez LM, Ramesh S, Chen M, Edelman A, Otterness C, Trussell J, et al. Progestin-only contraceptives: effects on weight. Cochrane Database Syst Rev. 2016(8):Cd008815.
- Maguire MJ, Weston J, Singh J, Marson AG. Antidepressants for people with epilepsy and depression. Cochrane Database Syst Rev. 2014(12):Cd010682.
- Michelena HI, Abel MD, Suri RM, Freeman WK, Click RL, Sundt TM, et al. Intraoperative echocardiography in valvular heart disease: an evidence-based appraisal. Mayo Clin Proc. 2010;85(7):646-55.
- Mohammed Vashist N, Samaan M, Mosli MH, Parker CE, MacDonald JK, Nelson SA, et al. Endoscopic scoring indices for evaluation of disease activity in ulcerative colitis. Cochrane Database Syst Rev. 2018;1:Cd011450.
- Mosli MH, Parker CE, Nelson SA, Baker KA, MacDonald JK, Zou GY, et al. Histologic scoring indices for evaluation of disease activity in ulcerative colitis. Cochrane Database Syst Rev. 2017;5:Cd011256.
- Mussa FF, Horton JD, Moridzadeh R, Nicholson J, Trimarchi S, Eagle KA. Acute Aortic Dissection and Intramural Hematoma: A Systematic Review. Jama. 2016;316(7):754-63.
- Nelson HD, Pappas M, Cantor A, Haney E, Holmes R. Risk Assessment, Genetic Counseling, and Genetic Testing for BRCA-Related Cancer in Women: Updated Evidence Report and Systematic Review for the US Preventive Services Task Force. Jama. 2019;322(7):666-85.
- Novak G, Parker CE, Pai RK, MacDonald JK, Feagan BG, Sandborn WJ, et al. Histologic scoring indices for evaluation of disease activity in Crohn's disease. Cochrane Database Syst Rev. 2017;7:Cd012351.
- Paijens ST, Leffers N, Daemen T, Helfrich W, Boezen HM, Cohlen BJ, et al. Antigen-specific active immunotherapy for ovarian cancer. Cochrane Database Syst Rev. 2018;9:Cd007287.
- Pepper DJ, Jaswal D, Sun J, Welsh J, Natanson C, Eichacker PQ. Evidence Underpinning the Centers for Medicare & Medicaid Services' Severe Sepsis and Septic Shock Management Bundle (SEP-1): A Systematic Review. Ann Intern Med. 2018;168(8):558-68.
- Selph SS, Bougatsos C, Dana T, Grusing S, Chou R. Screening for HIV Infection in Pregnant Women: Updated Evidence Report and Systematic Review for the US Preventive Services Task Force. Jama. 2019;321(23):2349-60.
- Simmonds MC, Brown JV, Heirs MK, Higgins JP, Mannion RJ, Rodgers MA, et al. Safety and effectiveness of recombinant human bone morphogenetic protein-2 for spinal fusion: a meta-analysis of individual-participant data. Ann Intern Med. 2013;158(12):877-89.
- Singh JA, Hossain A, Kotb A, Wells G. Risk of serious infections with immunosuppressive drugs and glucocorticoids for lupus nephritis: a systematic review and network meta-analysis. BMC Med. 2016;14(1):137.
- Sturt AS, Dokubo EK, Sint TT. Antiretroviral therapy (ART) for treating HIV infection in ART-eligible pregnant women. Cochrane Database Syst Rev. 2010(3):Cd008440.
- Thorlund JB, Juhl CB, Roos EM, Lohmander LS. Arthroscopic surgery for degenerative knee: systematic review and meta-analysis of benefits and harms. Bmj. 2015;350:h2747.
- Valenti R, Pantoni L, Markus HS. Treatment of vascular risk factors in patients with a diagnosis of Alzheimer's disease: a systematic review. BMC Med. 2014;12:160.
- Wallis CJ, Mahar AL, Choo R, Herschorn S, Kodama RT, Shah PS, et al. Second malignancies after radiotherapy for prostate cancer: systematic review and meta-analysis. Bmj. 2016;352:i851.
- Yang L, Sahlqvist S, McMinn A, Griffin SJ, Ogilvie D. Interventions to promote cycling: systematic review. Bmj. 2010;341:c5293.
- Yotsu RR, Richardson M, Ishii N. Drugs for treating Buruli ulcer (Mycobacterium ulcerans disease). Cochrane Database Syst Rev. 2018;8:Cd012118.

Umbrella review; n=16 articles

- Adam SS, McDuffie JR, Ortel TL, Williams JW. Comparative effectiveness of warfarin and new oral anticoagulants for the management of atrial fibrillation and venous thromboembolism: a systematic review. Ann Intern Med. 2012;157(11):796-807.
- Bhutta ZA, Yakoob MY, Lawn JE, Rizvi A, Friberg IK, Weissman E, et al. Stillbirths: what difference can we make and at what cost? Lancet. 2011;377(9776):1523-38.
- Chou R, Dana T, Bougatsos C, Blazina I, Khangura J, Zakher B. Screening for hepatitis B virus infection in adolescents and adults: a systematic review to update the U.S. Preventive Services Task Force recommendation. Ann Intern Med. 2014;161(1):31-45.
- Coker TR, Chan LS, Newberry SJ, Limbos MA, Suttorp MJ, Shekelle PG, et al. Diagnosis, microbial epidemiology, and antibiotic treatment of acute otitis media in children: a systematic review. Jama. 2010;304(19):2161-9.
- Demicheli V, Jefferson T, Al-Ansary LA, Ferroni E, Rivetti A, Di Pietrantonj C. Vaccines for preventing influenza in healthy adults. Cochrane Database Syst Rev. 2014(3):Cd001269.

– Fu R, Selph S, McDonagh M, Peterson K, Tiwari A, Chou R, et al. Effectiveness and harms of recombinant human bone morphogenetic protein-2 in spine fusion: a systematic review and meta-analysis. Ann Intern Med. 2013;158(12):890-902.<br>– Gartlehner G, Hansen RA, Morgan LC, Thaler K, Lux L, Van Noord M, et al. Comparative benefits and harms of second-generation antidepressants for treating major depressive disorder: an updated meta-analysis. Ann Intern Med. 2011;155(11):772-85.<br>– Ierodiakonou D, Garcia-Larsen V, Logan A, Groome A, Cunha S, Chivinge J, et al. Timing of Allergenic Food Introduction to the Infant Diet and Risk of Allergic or Autoimmune Disease: A Systematic Review and Meta-analysis. Jama. 2016;316(11):1181-92.<br>– Jonas DE, Amick HR, Feltner C, Weber RP, Arvanitis M, Stine A, et al. Screening for Obstructive Sleep Apnea in Adults: Evidence Report and Systematic Review for the US Preventive Services Task Force. Jama. 2017;317(4):415-33.<br>– Jonas DE, Kahwati LC, Yun JDY, Middleton JC, Coker-Schwimmer M, Asher GN. Screening for Atrial Fibrillation With Electrocardiography: Evidence Report and Systematic Review for the US Preventive Services Task Force. Jama. 2018;320(5):485-98.<br>– LeBlanc ES, Patnode CD, Webber EM, Redmond N, Rushkin M, O'Connor EA. Behavioral and Pharmacotherapy Weight Loss Interventions to Prevent Obesity-Related Morbidity and Mortality in Adults: Updated Evidence Report and Systematic Review for the US Preventive Services Task Force. Jama. 2018;320(11):1172-91.<br>– Melnikow J, Henderson JT, Burda BU, Senger CA, Durbin S, Weyrich MS. Screening for Cervical Cancer With High-Risk Human Papillomavirus Testing: Updated Evidence Report and Systematic Review for the US Preventive Services Task Force. Jama. 2018;320(7):687-705.<br>– Nelson HD, Smith ME, Griffin JC, Fu R. Use of medications to reduce risk for primary breast cancer: a systematic review for the U.S. Preventive Services Task Force. Ann Intern Med. 2013;158(8):604-14.<br>– Nelson HD, Fu R, Cantor A, Pappas M, Daeges M, Humphrey L. Effectiveness of Breast Cancer Screening: Systematic Review and Meta-analysis to Update the 2009 U.S. Preventive Services Task Force Recommendation. Ann Intern Med. 2016;164(4):244-55.<br>– Shekelle PG, Newberry SJ, FitzGerald JD, Motala A, O'Hanlon CE, Tariq A, et al. Management of Gout: A Systematic Review in Support of an American College of Physicians Clinical Practice Guideline. Ann Intern Med. 2017;166(1):37-51.<br>– Viswanathan M, Treiman KA, Kish-Doto J, Middleton JC, Coker-Schwimmer EJ, Nicholson WK. Folic Acid Supplementation for the Prevention of Neural Tube Defects: An Updated Evidence Report and Systematic Review for the US Preventive Services Task Force. Jama. 2017;317(2):190-203.
**Updated version of Systematic review included; n=3 articles**
– Dennert G, Zwahlen M, Brinkman M, Vinceti M, Zeegers MP, Horneber M. Selenium for preventing cancer. Cochrane Database Syst Rev. 2011(5):Cd005195.<br>– Zhang XL, Zhu QQ, Kang LN, Li XL, Xu B. Mid- and Long-Term Outcome Comparisons of Everolimus-Eluting Bioresorbable Scaffolds Versus Everolimus-Eluting Metallic Stents: A Systematic Review and Meta-analysis. Ann Intern Med. 2017;167(9):642-54.<br>– Anglemyer A, Rutherford GW, Baggaley RC, Egger M, Siegfried N. Antiretroviral therapy for prevention of HIV transmission in HIV-discordant couples. Cochrane Database Syst Rev. 2011(8):Cd009153.
**No eligible BoE-pair; n=78 articles**
– Aburto NJ, Hanson S, Gutierrez H, Hooper L, Elliott P, Cappuccio FP. Effect of increased potassium intake on cardiovascular risk factors and disease: systematic review and meta-analyses. Bmj. 2013;346:f1378.<br>– Aibana O, Huang CC, Aboud S, Arnedo-Pena A, Becerra MC, Bellido-Blasco JB, et al. Vitamin D status and risk of incident tuberculosis disease: A nested case-control study, systematic review, and individual-participant data meta-analysis. PLoS Med. 2019;16(9):e1002907.<br>– Anglemyer A, Horvath T, Rutherford G. The accessibility of firearms and risk for suicide and homicide victimization among household members: a systematic review and meta-analysis. Ann Intern Med. 2014;160(2):101-10.<br>– Baalbergen A, Veenstra Y, Stalpers LL, Ansink AC. Primary surgery versus primary radiation therapy with or without chemotherapy for early adenocarcinoma of the uterine cervix. Cochrane Database Syst Rev. 2010(1):Cd006248.<br>– Baalbergen A, Veenstra Y, Stalpers L. Primary surgery versus primary radiotherapy with or without chemotherapy for early adenocarcinoma of the uterine cervix. Cochrane Database Syst Rev. 2013(1):Cd006248.<br>– Bach PB, Mirkin JN, Oliver TK, Azzoli CG, Berry DA, Brawley OW, et al. Benefits and harms of CT screening for lung cancer: a systematic review. Jama. 2012;307(22):2418-29.<br>– Bao W, Rong Y, Rong S, Liu L. Dietary iron intake, body iron stores, and the risk of type 2 diabetes: a systematic review and meta-analysis. BMC Med. 2012;10:119.<br>– Booth RA, Ansari MT, Loit E, Tricco AC, Weeks L, Doucette S, et al. Assessment of thiopurine S-methyltransferase activity in patients prescribed thiopurines: a systematic review. Ann Intern Med. 2011;154(12):814-23, w-295-8.<br>– Carrier M, Le Gal G, Wells PS, Rodger MA. Systematic review: case-fatality rates of recurrent venous thromboembolism and major bleeding events among patients treated for venous thromboembolism. Ann Intern Med. 2010;152(9):578-89.<br>– Chang LW, Harris J, Humphreys E. Optimal monitoring strategies for guiding when to switch first-line antiretroviral therapy regimens for treatment failure in adults and adolescents living with HIV in low-resource settings. Cochrane Database Syst Rev. 2010(4):Cd008494.<br>– Chou R, Cottrell EB, Wasson N, Rahman B, Guise JM. Screening for hepatitis C virus infection in adults: a systematic review for the U.S. Preventive Services Task Force. Ann Intern Med. 2013;158(2):101-8.<br>– Chou R, Hartung D, Rahman B, Wasson N, Cottrell EB, Fu R. Comparative effectiveness of antiviral treatment for hepatitis C virus infection in adults: a systematic review. Ann Intern Med. 2013;158(2):114-23.<br>– Chou R, Evans C, Hoverman A, Sun C, Dana T, Bougatsos C, et al. Preexposure Prophylaxis for the Prevention of HIV Infection: Evidence Report and Systematic Review for the US Preventive Services Task Force. Jama. 2019;321(22):2214-30.<br>– Costelloe C, Metcalfe C, Lovering A, Mant D, Hay AD. Effect of antibiotic prescribing in primary care on antimicrobial resistance in individual patients: systematic review and meta-analysis. Bmj. 2010;340:c2096.<br>– Dangour AD, Watson L, Cumming O, Boisson S, Che Y, Velleman Y, et al. Interventions to improve water quality and supply,

sanitation and hygiene practices, and their effects on the nutritional status of children. Cochrane Database Syst Rev. 2013(8):Cd009382.
- Davey P, Marwick CA, Scott CL, Charani E, McNeil K, Brown E, et al. Interventions to improve antibiotic prescribing practices for hospital inpatients. Cochrane Database Syst Rev. 2017;2:Cd003543.
- Edmond KM, Kortsalioudaki C, Scott S, Schrag SJ, Zaidi AK, Cousens S, et al. Group B streptococcal disease in infants aged younger than 3 months: systematic review and meta-analysis. Lancet. 2012;379(9815):547-56.
- Fayad AI, Buamscha DG, Ciapponi A. Intensity of continuous renal replacement therapy for acute kidney injury. Cochrane Database Syst Rev. 2016;10:Cd010613.
- Goudet SM, Bogin BA, Madise NJ, Griffiths PL. Nutritional interventions for preventing stunting in children (birth to 59 months) living in urban slums in low- and middle-income countries (LMIC). Cochrane Database Syst Rev. 2019;6(6):Cd011695.
- Gowing L, Ali R, White JM. Opioid antagonists with minimal sedation for opioid withdrawal. Cochrane Database Syst Rev. 2017;5:Cd002021.
- Gurusamy KS, Nagendran M, Guerrini GP, Toon CD, Zinnuroglu M, Davidson BR. Intraperitoneal local anaesthetic instillation versus no intraperitoneal local anaesthetic instillation for laparoscopic cholecystectomy. Cochrane Database Syst Rev. 2014(3):Cd007337.
- Haider BA, Olofin I, Wang M, Spiegelman D, Ezzati M, Fawzi WW. Anaemia, prenatal iron use, and risk of adverse pregnancy outcomes: systematic review and meta-analysis. Bmj. 2013;346:f3443.
- Hartling L, Abou-Setta AM, Dursun S, Mousavi SS, Pasichnyk D, Newton AS. Antipsychotics in adults with schizophrenia: comparative effectiveness of first-generation versus second-generation medications: a systematic review and meta-analysis. Ann Intern Med. 2012;157(7):498-511.
- Henderson JT, Whitlock EP, O'Connor E, Senger CA, Thompson JH, Rowland MG. Low-dose aspirin for prevention of morbidity and mortality from preeclampsia: a systematic evidence review for the U.S. Preventive Services Task Force. Ann Intern Med. 2014;160(10):695-703.
- Henson CC, Burden S, Davidson SE, Lal S. Nutritional interventions for reducing gastrointestinal toxicity in adults undergoing radical pelvic radiotherapy. Cochrane Database Syst Rev. 2013(11):Cd009896.
- Imdad A, Nicholson MR, Tanner-Smith EE, Zackular JP, Gomez-Duarte OG, Beaulieu DB, et al. Fecal transplantation for treatment of inflammatory bowel disease. Cochrane Database Syst Rev. 2018;11:Cd012774.
- Jefferson T, Di Pietrantonj C, Rivetti A, Bawazeer GA, Al-Ansary LA, Ferroni E. Vaccines for preventing influenza in healthy adults. Cochrane Database Syst Rev. 2010(7):Cd001269.
- Jonas DE, Amick HR, Feltner C, Bobashev G, Thomas K, Wines R, et al. Pharmacotherapy for adults with alcohol use disorders in outpatient settings: a systematic review and meta-analysis. Jama. 2014;311(18):1889-900.
- Jonas DE, Reddy S, Middleton JC, Barclay C, Green J, Baker C, et al. Screening for Cardiovascular Disease Risk With Resting or Exercise Electrocardiography: Evidence Report and Systematic Review for the US Preventive Services Task Force. Jama. 2018;319(22):2315-28.
- Kahn SR, Morrison DR, Cohen JM, Emed J, Tagalakis V, Roussin A, et al. Interventions for implementation of thromboprophylaxis in hospitalized medical and surgical patients at risk for venous thromboembolism. Cochrane Database Syst Rev. 2013(7):Cd008201.
- Khanna R, Nelson SA, Feagan BG, D'Haens G, Sandborn WJ, Zou GY, et al. Endoscopic scoring indices for evaluation of disease activity in Crohn's disease. Cochrane Database Syst Rev. 2016(8):Cd010642.
- Kredo T, Ford N, Adeniyi FB, Garner P. Decentralising HIV treatment in lower- and middle-income countries. Cochrane Database Syst Rev. 2013(6):Cd009987.
- Kroon FP, van der Burg LR, Ramiro S, Landewe RB, Buchbinder R, Falzon L, et al. Non-steroidal anti-inflammatory drugs (NSAIDs) for axial spondyloarthritis (ankylosing spondylitis and non-radiographic axial spondyloarthritis). Cochrane Database Syst Rev. 2015(7):Cd010952.
- Kunisaki KM, Greer N, Khalil W, Koffel E, Koeller E, MacDonald R, et al. Provider Types and Outcomes in Obstructive Sleep Apnea Case Finding and Treatment: A Systematic Review. Ann Intern Med. 2018;168(3):195-202.
- Kyrgiou M, Athanasiou A, Kalliala IEJ, Paraskevaidi M, Mitra A, Martin-Hirsch PP, et al. Obstetric outcomes after conservative treatment for cervical intraepithelial lesions and early invasive disease. Cochrane Database Syst Rev. 2017;11(11):Cd012847.
- Langstrom N, Enebrink P, Lauren EM, Lindblom J, Werko S, Hanson RK. Preventing sexual abusers of children from reoffending: systematic review of medical and psychological interventions. Bmj. 2013;347:f4630.
- Lansbury L, Rodrigo C, Leonardi-Bee J, Nguyen-Van-Tam J, Lim WS. Corticosteroids as adjunctive therapy in the treatment of influenza. Cochrane Database Syst Rev. 2019;2:Cd010406.
- Lin JS, Bowles EJA, Williams SB, Morrison CC. Screening for Thyroid Cancer: Updated Evidence Report and Systematic Review for the US Preventive Services Task Force. Jama. 2017;317(18):1888-903.
- Majorin F, Torondel B, Ka Seen Chan G, Clasen T. Interventions to improve disposal of child faeces for preventing diarrhoea and soil-transmitted helminth infection. Cochrane Database Syst Rev. 2019;9:Cd011055.
- Marschall J, Carpenter CR, Fowler S, Trautner BW. Antibiotic prophylaxis for urinary tract infections after removal of urinary catheter: meta-analysis. Bmj. 2013;346:f3147.
- McQuade CN, Mizus M, Wald JW, Goldberg L, Jessup M, Umscheid CA. Brain-Type Natriuretic Peptide and Amino-Terminal Pro-Brain-Type Natriuretic Peptide Discharge Thresholds for Acute Decompensated Heart Failure: A Systematic Review. Ann Intern Med. 2017;166(3):180-90.
- Mhimbira FA, Cuevas LE, Dacombe R, Mkopi A, Sinclair D. Interventions to increase tuberculosis case detection at primary healthcare or community-level services. Cochrane Database Syst Rev. 2017;11:Cd011432.
- Nabi G, Cleves A, Shelley M. Surgical management of localised renal cell carcinoma. Cochrane Database Syst Rev. 2010(3):Cd006579.
- Negrini S, Minozzi S, Bettany-Saltikov J, Chockalingam N, Grivas TB, Kotwicki T, et al. Braces for idiopathic scoliosis in adolescents. Cochrane Database Syst Rev. 2015(6):Cd006850.

- O'Connor E, Senger CA, Henninger ML, Coppola E, Gaynes BN. Interventions to Prevent Perinatal Depression: Evidence Report and Systematic Review for the US Preventive Services Task Force. Jama. 2019;321(6):588-601.
- Paul S, Saxena A, Terrin N, Viveiros K, Balk EM, Wong JB. Hepatitis B Virus Reactivation and Prophylaxis During Solid Tumor Chemotherapy: A Systematic Review and Meta-analysis. Ann Intern Med. 2016;164(1):30-40.
- Plourde G, Pancholy SB, Nolan J, Jolly S, Rao SV, Amhed I, et al. Radiation exposure in relation to the arterial access site used for diagnostic coronary angiography and percutaneous coronary intervention: a systematic review and meta-analysis. Lancet. 2015;386(10009):2192-203.
- Poirot E, Skarbinski J, Sinclair D, Kachur SP, Slutsker L, Hwang J. Mass drug administration for malaria. Cochrane Database Syst Rev. 2013(12):Cd008846.
- Reynolds A, Mann J, Cummings J, Winter N, Mete E, Te Morenga L. Carbohydrate quality and human health: a series of systematic reviews and meta-analyses. Lancet. 2019;393(10170):434-45.
- Rojas-Reyes MX, Granados Rugeles C, Charry-Anzola LP. Oxygen therapy for lower respiratory tract infections in children between 3 months and 15 years of age. Cochrane Database Syst Rev. 2014(12):Cd005975.
- Santos JAR, Christoforou A, Trieu K, McKenzie BL, Downs S, Billot L, et al. Iodine fortification of foods and condiments, other than salt, for preventing iodine deficiency disorders. Cochrane Database Syst Rev. 2019;2:Cd010734.
- Saulle R, Vecchi S, Gowing L. Supervised dosing with a long-acting opioid medication in the management of opioid dependence. Cochrane Database Syst Rev. 2017;4:Cd011983.
- Schoot RA, Kremer LC, van de Wetering MD, van Ommen CH. Systemic treatments for the prevention of venous thrombo-embolic events in paediatric cancer patients with tunnelled central venous catheters. Cochrane Database Syst Rev. 2013(9):Cd009160.
- Schoot RA, van Dalen EC, van Ommen CH, van de Wetering MD. Antibiotic and other lock treatments for tunnelled central venous catheter-related infections in children with cancer. Cochrane Database Syst Rev. 2013(6):Cd008975.
- Singh M, Holmes DR, Dehmer GJ, Lennon RJ, Wharton TP, Kutcher MA, et al. Percutaneous coronary intervention at centers with and without on-site surgery: a meta-analysis. Jama. 2011;306(22):2487-94.
- Smith ME, Totten A, Hickam DH, Fu R, Wasson N, Rahman B, et al. Pressure ulcer treatment strategies: a systematic comparative effectiveness review. Ann Intern Med. 2013;159(1):39-50.
- Sobieraj DM, Baker WL, Nguyen E, Weeda ER, Coleman CI, White CM, et al. Association of Inhaled Corticosteroids and Long-Acting Muscarinic Antagonists With Asthma Control in Patients With Uncontrolled, Persistent Asthma: A Systematic Review and Meta-analysis. Jama. 2018;319(14):1473-84.
- Sobieraj DM, Weeda ER, Nguyen E, Coleman CI, White CM, Lazarus SC, et al. Association of Inhaled Corticosteroids and Long-Acting β-Agonists as Controller and Quick Relief Therapy With Exacerbations and Symptom Control in Persistent Asthma: A Systematic Review and Meta-analysis. Jama. 2018;319(14):1485-96.
- Strunz EC, Addiss DG, Stocks ME, Ogden S, Utzinger J, Freeman MC. Water, sanitation, hygiene, and soil-transmitted helminth infection: a systematic review and meta-analysis. PLoS Med. 2014;11(3):e1001620.
- Suthar AB, Ford N, Bachanas PJ, Wong VJ, Rajan JS, Saltzman AK, et al. Towards universal voluntary HIV testing and counselling: a systematic review and meta-analysis of community-based approaches. PLoS Med. 2013;10(8):e1001496.
- Swiger KJ, Manalac RJ, Blumenthal RS, Blaha MJ, Martin SS. Statins and cognition: a systematic review and meta-analysis of short- and long-term cognitive effects. Mayo Clin Proc. 2013;88(11):1213-21.
- Thaler K, Delivuk M, Chapman A, Gaynes BN, Kaminski A, Gartlehner G. Second-generation antidepressants for seasonal affective disorder. Cochrane Database Syst Rev. 2011(12):Cd008591.
- Thangaratinam S, Tan A, Knox E, Kilby MD, Franklyn J, Coomarasamy A. Association between thyroid autoantibodies and miscarriage and preterm birth: meta-analysis of evidence. Bmj. 2011;342:d2616.
- Thomas RE, Jefferson T, Lasserson TJ. Influenza vaccination for healthcare workers who care for people aged 60 or older living in long-term care institutions. Cochrane Database Syst Rev. 2013(7):Cd005187.
- Thomas RE, Jefferson T, Lasserson TJ. Influenza vaccination for healthcare workers who care for people aged 60 or older living in long-term care institutions. Cochrane Database Syst Rev. 2016(6):Cd005187.
- Verbeek JH, Ijaz S, Mischke C, Ruotsalainen JH, Makela E, Neuvonen K, et al. Personal protective equipment for preventing highly infectious diseases due to exposure to contaminated body fluids in healthcare staff. Cochrane Database Syst Rev. 2016;4:Cd011621.
- Verbeek JH, Martimo KP, Karppinen J, Kuijer PP, Viikari-Juntura E, Takala EP. Manual material handling advice and assistive devices for preventing and treating back pain in workers. Cochrane Database Syst Rev. 2011(6):Cd005958.
- Veroniki AA, Cogo E, Rios P, Straus SE, Finkelstein Y, Kealey R, et al. Comparative safety of anti-epileptic drugs during pregnancy: a systematic review and network meta-analysis of congenital malformations and prenatal outcomes. BMC Med. 2017;15(1):95.
- Villar JC, Perez JG, Cortes OL, Riarte A, Pepper M, Marin-Neto JA, et al. Trypanocidal drugs for chronic asymptomatic Trypanosoma cruzi infection. Cochrane Database Syst Rev. 2014(5):Cd003463.
- Wagner AD, Thomssen C, Haerting J, Unverzagt S. Vascular-endothelial-growth-factor (VEGF) targeting therapies for endocrine refractory or resistant metastatic breast cancer. Cochrane Database Syst Rev. 2012(7):Cd008941.
- Waldron C, Nunn J, Mac Giolla Phadraig C, Comiskey C, Guerin S, van Harten MT, et al. Oral hygiene interventions for people with intellectual disabilities. Cochrane Database Syst Rev. 2019;5:Cd012628.
- West CP, Dyrbye LN, Erwin PJ, Shanafelt TD. Interventions to prevent and reduce physician burnout: a systematic review and meta-analysis. Lancet. 2016;388(10057):2272-81.
- West S, Nolan SJ, Cotton J, Gandhi S, Weston J, Sudan A, et al. Surgery for epilepsy. Cochrane Database Syst Rev. 2015(7):Cd010541.
- Wilcox ME, Chong CA, Stanbrook MB, Tricco AC, Wong C, Straus SE. Does this patient have an exudative pleural effusion? The Rational Clinical Examination systematic review. Jama. 2014;311(23):2422-31.
- Wong MC, Glenny AM, Tsang BW, Lo EC, Worthington HV, Marinho VC. Topical fluoride as a cause of dental fluorosis in children. Cochrane Database Syst Rev. 2010(1):Cd007693.

– Yokoyama Y, Nishimura K, Barnard ND, Takegami M, Watanabe M, Sekikawa A, et al. Vegetarian diets and blood pressure: a meta-analysis. JAMA Intern Med. 2014;174(4):577-87.<br>– Young MK, Cripps AW, Nimmo GR, van Driel ML. Post-exposure passive immunisation for preventing rubella and congenital rubella syndrome. Cochrane Database Syst Rev. 2015(9):Cd010586.<br>– Young MK, Nimmo GR, Cripps AW, Jones MA. Post-exposure passive immunisation for preventing measles. Cochrane Database Syst Rev. 2014(4):Cd010056.
**Incomparability between outcomes of BoE: Continuous versus binary, incomplete outcome data (missing effect estimates or confidence intervals) in a BoE-pair without meta-analysis for both BoE; n=17 articles**
– Ferri M, Allara E, Bo A, Gasparrini A, Faggiano F. Media campaigns for the prevention of illicit drug use in young people. Cochrane Database Syst Rev. 2013(6):Cd009287.<br>– Hartmann-Boyce J, McRobbie H, Bullen C, Begh R, Stead LF, Hajek P. Electronic cigarettes for smoking cessation. Cochrane Database Syst Rev. 2016;9(9):Cd010216.<br>– Hooper L, Abdelhamid A, Bunn D, Brown T, Summerbell CD, Skeaff CM. Effects of total fat intake on body weight. Cochrane Database Syst Rev. 2015(8):Cd011834.<br>– Hooper L, Abdelhamid A, Moore HJ, Douthwaite W, Skeaff CM, Summerbell CD. Effect of reducing total fat intake on body weight: systematic review and meta-analysis of randomised controlled trials and cohort studies. Bmj. 2012;345:e7666.<br>– Hutton B, Joseph L, Fergusson D, Mazer CD, Shapiro S, Tinmouth A. Risks of harms using antifibrinolytics in cardiac surgery: systematic review and network meta-analysis of randomised and observational studies. Bmj. 2012;345:e5798.<br>– Maher AR, Maglione M, Bagley S, Suttorp M, Hu JH, Ewing B, et al. Efficacy and comparative effectiveness of atypical antipsychotic medications for off-label uses in adults: a systematic review and meta-analysis. Jama. 2011;306(12):1359-69.<br>– McKnight RF, Adida M, Budge K, Stockton S, Goodwin GM, Geddes JR. Lithium toxicity profile: a systematic review and meta-analysis. Lancet. 2012;379(9817):721-8.<br>– McRobbie H, Bullen C, Hartmann-Boyce J, Hajek P. Electronic cigarettes for smoking cessation and reduction. Cochrane Database Syst Rev. 2014(12):Cd010216.<br>– Mhaskar R, Kumar A, Miladinovic B, Djulbegovic B. Bisphosphonates in multiple myeloma: an updated network meta-analysis. Cochrane Database Syst Rev. 2017;12:Cd003188.<br>– Mhaskar R, Redzepovic J, Wheatley K, Clark OA, Miladinovic B, Glasmacher A, et al. Bisphosphonates in multiple myeloma. Cochrane Database Syst Rev. 2010(3):Cd003188.<br>– Mhaskar R, Redzepovic J, Wheatley K, Clark OA, Miladinovic B, Glasmacher A, et al. Bisphosphonates in multiple myeloma: a network meta-analysis. Cochrane Database Syst Rev. 2012(5):Cd003188.<br>– Paige NM, Miake-Lye IM, Booth MS, Beroes JM, Mardian AS, Dougherty P, et al. Association of Spinal Manipulative Therapy With Clinical Benefit and Harm for Acute Low Back Pain: Systematic Review and Meta-analysis. Jama. 2017;317(14):1451-60.<br>– Pega F, Liu SY, Walter S, Pabayo R, Saith R, Lhachimi SK. Unconditional cash transfers for reducing poverty and vulnerabilities: effect on use of health services and health outcomes in low- and middle-income countries. Cochrane Database Syst Rev. 2017;11:Cd011135.<br>– Shepherd E, Salam RA, Manhas D, Synnes A, Middleton P, Makrides M, et al. Antenatal magnesium sulphate and adverse neonatal outcomes: A systematic review and meta-analysis. PLoS Med. 2019;16(12):e1002988.<br>– Toews I, Lohner S, Kullenberg de Gaudry D, Sommer H, Meerpohl JJ. Association between intake of non-sugar sweeteners and health outcomes: systematic review and meta-analyses of randomised and non-randomised controlled trials and observational studies. Bmj. 2019;364:k4718.<br>– Tricco AC, Ashoor HM, Antony J, Beyene J, Veroniki AA, Isaranuwatchai W, et al. Safety, effectiveness, and cost effectiveness of long acting versus intermediate acting insulin for patients with type 1 diabetes: systematic review and network meta-analysis. Bmj. 2014;349:g5459.<br>– Weiss J, Freeman M, Low A, Fu R, Kerfoot A, Paynter R, et al. Benefits and Harms of Intensive Blood Pressure Treatment in Adults Aged 60 Years or Older: A Systematic Review and Meta-analysis. Ann Intern Med. 2017;166(6):419-29.

BoE= body of evidence; RCT= randomized controlled trial.

Table S4: Characteristics of included bodies of evidence from randomized controlled trials

Reference/ year	Intervention	Outcome	Number of studies	Sampl e size	Case s	Description of population	Age; mean/ range (years)	Description of intervention	Description of comparator	Description of outcome	Study design	Follow-up
Abou-Setta 2011 (74)	Nerve block	Delirium	4	461	44	Mostly elderly population (>65 years) / with: hip fracture / community dwelling or not specified / without: dementia, cognitive deficits	>16	Regional anaesthesia, either epidural, fascia iliaca compartment block or 3-in-1 nerve block / continuous or single administration of anesthetics / mostly with: Bupivacaine, partly with: Bupivacaine and Morphine / doses: epidural Bupivacaine 0.125% and Morphine, 4ml of 50ug per ml/h; 3-in-1 nerve block with Bupivacaine 0.5%, 30ml, single adiminstration; fascia iliaca compartment block with 0.25% Bupivacaine 0.3ml/kg (interval not reported) or 0.25mg of 0.3ml/kg 24 hours before and after surgery	Placebo (2x) / IV analgesia with Morphine 0.1mg/kg, single administration (1x) / IV NSAID (1x)	Delirium	Parallel	NR
Abou-Setta 2011 (74)	Spinal anesthesia	All-cause mortality	2	99	15	Mostly elderly female population / with: hip fracture (2x), stable CAD (1x)	>65	Spinal anesthesia with Bupivacaine 0.5% or Mepivacaine 4% (dose not reported)/ spinal anestheisa with Bupivacaine 0.5% 1.6ml incremental dosage or Bupivacaine 0.5% 2.5ml, single administration	General anesthesia; with: Fentanyl 1-2ug/kg bolus followed by 25-50ug maintenance dose on demand or not reported	Mortality at 30 days	Parallel	NR
Aburto 2013 (75)	Low sodium	All-cause mortality	4	3,595	69	Male and female population / mostly healthy, with: normal blood pressure (3x), no use of antihypertensive medication / partly with: moderate overweight, hypertension (1x)	38.5-58.6 (mean)	Dietary and behavioural counselling to reduce sodium intake / with or without: weight loss intervention	No dietary counselling / recommendations for generally healthy diet	All-cause mortality	Parallel, factorial	2-11.5 years

Aburto 2013 (75)	Low sodium	Cardiovascular disease	2	720	93	Male and female population / health status: hypertensive, taking one or two drugs for hypertension (1x) / otherwise healthy males with hypertension (1x)	57.1-65.8 (mean)	Dietary counselling to reduce sodium intake	Unspecific retention procedures (presentations unrelated to the subject of the trial) / no treatment	Cardiovascular disease	Parallel, factorial	24-30 months
Ahmad 2015 (27)	Intra-aortic balloon pump	All-cause mortality	12	2,123	361	Population with acute CVD (MI, complicated MI, cardiogenic shock, cardiac failure)	NR	Intra-aortic balloon pump before or after PCI / mostly co-interventions for all patients: PCI (8/12), thrombolysis (2/12) / no reperfusion therapy (2/12)	No intra-aortic balloon pump, intensive care	Mortality at 30 days	Parallel	NR
Alexander 2017 (76)	DHA and EPA	Coronary heart disease	18	47,494	NR	Male and female population / primary, mixed or secondary prevention of CVD / mostly in highly developed countries	>18	Ethyl esters, fish oil, fatty fish or EPA and DHA enriched margarine / EPA+DHA: 0.38-5.04 g/day	Mostly oils not containing EPA and DHA (oil, com oil, oleic acid margarine, oil mixture without marine n-3 fatty acids) / partly: no supplements, dietary advice, aluminium hydroxide (1x), gelatin (1x)	Any coronary heart disease event	Parallel, factorial	0.5-7 years
Alexander 2017 (76)	DHA and EPA	Coronary heart disease mortality	14	39,537	NR	Male and female population / primary, mixed or secondary prevention of CVD / mostly in highly developed countries	>18	Ethyl esters, fish oil, fatty fish or EPA and DHA enriched margarine / EPA+DHA: 0.38-5.04 g/day	Mostly oils not containing EPA and DHA (oil, com oil, oleic acid margarine, sunflower oil, oil mixture without marine n-3 fatty acids) / partly other controls (no supplements, dietary advice, aluminium hydroxide, gelatine)	Fatal coronary heart disease events	Parallel, factorial	0.5-7 years
Alexander 2017 (76)	DHA and EPA	Coronary heart disease incidence	9	33,441	NR	Male and female population / primary, mixed or secondary prevention of CVD / mostly in highly developed countries	>18	Ethyl esters, fish oil, fatty fish / EPA+DHA: 0.6-3.46 g/day	Oils not containing EPA and DHA (oil, com oil, oleic acid margarine, sunflower oil, oil mixture without marine n-3 fatty acids) / other controls (no supplements, dietary advice, aluminium hydroxide, gelatine)	Non-fatal coronary heart disease events	Parallel, factorial	1-7 years

Alipanah 2018 (24)	Self-administered therapy	Low treatment success	4	1,603	1.151	Adult population (>15 years) / with: mostly pulmonary, smear positive tuberculosis / excluding or including: multidrug-resistant tuberculosis and patients with history of antituberculosis treatment / setting: Thailand, Pakistan, South Africa	≥15	Self-administered therapy	Directly observed therapy / either observed by health staff in hospital or by community or family member	Low treatment success	Parallel	NR
Alipanah 2018 (24)	Self-administered therapy	Low treatment completion	5	1,982	406	Adult population (>15 years) / with: mostly pulmonary, smear positive tuberculosis / excluding or including multidrug-resistant tuberculosis and patients with history of antituberculosis treatment / partly population without: HIV / setting: India, Thailand, Pakistan, South Africa	≥15	Self-administered therapy	Directly observed therapy / either observed by health staff in hospital or by community or family member	Low treatment completion	Parallel	NR
Alipanah 2018 (24)	Self-administered therapy	All-cause mortality	4	1,603	67	Adult population (>15 years) / with: mostly pulmonary, smear positive tuberculosis / excluding or including multidrug-resistant tuberculosis and patients with history of antituberculosis treatment /setting: Thailand, Pakistan, South Africa	≥15	Self-administered therapy	Directly observed therapy / either observed by health staff in hospital or by community or family member	Mortality	Parallel	NR
Anglemyer 2013 (77)	Antiretroviral therapy	HIV infection	1	3,500	39	Serodiscordant couples (HIV status), mostly heterosexual (≈97%) / CD4 count 350-550 cells/µL / setting: nine countries (Botswana, Brazil, India, Malawi, Kenya, South Africa, Thailand, United States of	≥18	Immediate ART (350-550 CD4 cells/µL)	Delayed ART / initiation of treatment when CD4-cells <250 cells/µL or AIDS defining disease	Incident HIV infection of uninfected partner in serodiscordant couples (virologically linked and unlinked)	Parallel	1,585 (immediate treatment) to 1,567 (delayed treatment) person-years

						America, Zimbabwe)						
Azad 2017 (21)	Nonnutritive sweeteners	Body Mass Index	3	242	NA	Male and female population / with: mild essential hypertension (2x) or overweight (1x) but otherwise healthy (3x) (without: cancer, CVD) / mean BMI: 23-34/ setting: Brazil, China, Iran	32-52 (mean)	Stevioside capsules daily 3.8-15mg/kg (1x) or 1500mg (1x) / one 250ml diet beverage daily 5 days/week / co-intervention for all patients (1x): bi-weekly dietary and behavioural counseling to increase physical activity (based at weight-loss clinic)	Placebo, water	BMI change at latest follow-up	Parallel	0.5-2 years (duration)
Barnard 2015 (28)	Surgical abortion by mid-level providers	Failure or incomplete abortion	2	2,789	24	Pregnant women / gestational age: <12 weeks / urban and peri-urban areas in South-Africa and Vietnam (two studies at different time and setting with same methodology by same author)	>18	Surgical abortion by mid-level providers with government-accredited training in abortion	Surgical abortion by doctors	Failure or incomplete abortion	Parallel	10-14 days follow-up
Barnard 2015 (28)	Surgical abortion by mid-level providers	Complications	2	2,789	4	Pregnant women / gestational age: <12 weeks / urban and peri-urban areas in South-Africa and Vietnam (two studies at different time and setting with same methodology by same author)	>18	Surgical abortion by mid-level providers with government-accredited training in abortion	Surgical abortion by doctors	Immediate complications (excessive bleeding after abortion, cervical injury, confirmed/suspected perforation, adverse drug reaction); Delayed complications (retained POC needing re-evacuation, haematometra, post-abortion pelvic infection, excessive post-abortion bleeding, abortion-related death)	Parallel	10-14 days follow-up

Barnard 2015 (28)	Surgical abortion by mid-level providers	Abortion failure and complications	2	2,789	28	Pregnant women / gestational age: <12 weeks / urban and peri-urban areas in South-Africa and Vietnam (two studies at different time and setting with same methodology by same author)	>18	Surgical abortion by mid-level providers with government-accredited training in abortion	Surgical abortion by doctors	Immediate complications (excessive bleeding after abortion, cervical injury, confirmed/suspected perforation, adverse drug reaction); Delayed complications (retained POC needing re-evacuation, haematometra, post-abortion pelvic infection, excessive post-abortion bleeding, abortion-related death)	Parallel	10-14 days follow-up
Bellemain-Appaix 2012 (48)	Clopidogrel	All-cause mortality	7	8,608	151	Adults with CAD/CHD scheduled for catheterisation, PCI, or both / mostly with ACS, partly with definite STEMI, partly with elective PCI	NR	Clopidogrel 300-900mg pretreatment before PCI / mostly several hours before procedure (minimum 2 hours to median 10 days before procedure (range)) / followed by maintanance dose (3x)	Delayed treatment with Clopidogrel (immediately before, during or after the procedure) with a loading dose of 300-900mg	All-cause mortality	Parallel	7 days or hospital discharge to 1 year
Bellemain-Appaix 2012 (48)	Clopidogrel	Major bleeding	7	8,608	286	Adults with CAD/CHD scheduled for catheterisation, PCI, or both / mostly with ACS, partly with definite STEMI, partly with elective PCI	NR	Clopidogrel 300-900mg pretreatment before PCI / mostly several hours before procedure (minimum 2 hours to median 10 days before procedure (range)) / followed by maintanance dose (3x)	Delayed treatment with Clopidogrel (immediately before, during or after the procedure) with a loading dose of 300-900mg	Major bleeding (TIMI major/minor or substantially disabling bleeding, intraocular bleeding leading to the loss of vision, or bleeding necessitating transfusion of 2 or more units of blood (1x))	Parallel	7 days or hospital discharge to 1 year

Bellemain-Appaix 2012 (48)	Clopidogrel	Coronary heart disease	7	8,608	955	Adults with CAD/CHD scheduled for catheterisation, PCI, or both / mostly with ACS, partly with definite STEMI, partly with elective PCI	NR	Clopidogrel 300-900mg pretreatment before PCI / mostly several hours before procedure (minimum 2 hours to median 10 days before procedure (range)) / followed by maintanance dose (3x)	Delayed treatment with Clopidogrel (immediately before, during or after the procedure) with a loading dose of 300-900mg	Major coronary events (CV death, death, MI, periprocedural MI, stroke, urgent target vessel revascularization)	Parallel	7 days or hospital discharge to 1 year
Bellemain-Appaix 2014 (47)	P2Y12 inhibitors	All-cause mortality	3	7,246	39	Adults undergoing PCI / 66.5-100% (range) with: non-ST elevation ACS; non-acute patients only (1x)	61.5-64.2 (mean)	Clopidogrel with subsequent PCI, 300mg loading dose followed by maintenance dose, mean 2 hours to 10 days before procedure / randomization after coronary angiogramm (1x) / Prasugrel 30mg median 4,4 hours before PCI and immediately after PCI (1x)	Placebo instead of pretreatment / delayed treatment with Clopidogrel or Prasugrel	All-cause death; at 7, 28 or 30 days respectively	Parallel	1-12 months (follow-up)
Bellemain-Appaix 2014 (47)	P2Y12 inhibitors	Major bleeding	3	7,547	154	Adults undergoing PCI / 66.5-100% (range) with: non-ST elevation ACS; non-acute patients only (1x)	61.5-64.2 (mean)	Clopidogrel with subsequent PCI, 300mg loading dose followed by maintenance dose, mean 2 hours to 10 days before procedure / randomization after coronary angiogramm (1x) / Prasugrel 30mg median 4,4 hours before PCI and immediately after PCI (1x)	Placebo instead of pretreatment / delayed treatment with Clopidogrel or Prasugrel	Major bleeding; at 7, 28 or 30 days respectively; Bleeding: Thrombolysis in Myocardial Infarction major bleeding criteria (2x) or substantially disabling bleeding, intraocular bleeding leading to loss of vision, or bleeding necessitating transfusion of ≥2 units of blood (1x)	Parallel	1-12 months (follow-up)
Bellemain-Appaix 2014 (47)	P2Y12 inhibitors	Main composite ischemic endpoint	3	7,246	645	Adults undergoing PCI / 66.5-100% (range) with: non-ST elevation ACS; non-acute patients only (1x)	61.5-64.2 (mean)	Clopidogrel with subsequent PCI, 300mg loading dose followed by maintenance dose, mean 2 hours to 10 days before procedure / randomization after coronary angiogramm (1x) / Prasugrel 30mg median 4,4 hours before PCI and immediately after PCI (1x)	Placebo instead of pretreatment / delayed treatment with Clopidogrel or Prasugrel	Main composite ischemic endpoint; at 7, 28 or 30 days respectively	Parallel	1-12 months (follow-up)

Bloomfield 2016 (22)	Mediterranean diet	Breast cancer	1	4,152	NR	Female population / without prevalent CVD or other severe conditions but with metabolic or CVD risk factors, without breast cancer / setting: Spain	67.4-68.1 (mean)	Mediterranean diet / free extra-virgin olive oil or nuts included	Low-fat diet	Breast cancer incidence	Parallel	4.8 years
Bolland 2015 (49)	Calcium	All fractures	22	68,505	6,725	Mostly general female population, community dwelling / institutionalised population (2x)	56-86 (mean)	Supplements / calcium (8x) only or calcium and vitamin D (13x), calcium and UV-light (1x) / doses: calcium 600 to 1600 mg/day, vitamin D 400 to 1000 IU/day or 300 000 IU IM stat (2x)	Placebo, "control"	All fractures	Parallel, factorial, cluster	1-7 years (duration)
Bolland 2015 (49)	Calcium	Vertebral fracture	12	48,967	666	Mostly general female population, community dwelling / institutionalised population (1x)	58-80 (mean)	Supplements / calcium (8x) or calcium and vitamin D (4x) / doses: calcium 750 to 1600 mg/day, vitamin D 400 to 800 IU/d or 300 000 IU IM stat (1x)	Placebo, "control"	Vertebral fractures	Parallel, factorial	1.5-7 years (duration)
Bolland 2015 (49)	Calcium	Hip fracture	13	56,648	981	Mostly general female population, community dwelling / institutionalised population (2x)	58-85 (mean)	Supplements / calcium (4x) or calcium and vitamin D (9x) / doses: calcium 400 to 1200 mg/day, vitamin D 400 to 800 IU/d or 300 000 IU IM stat (1x)	Placebo, "control"	Hip fracture	Parallel, factorial	1-7 years (duration)
Brenner 2014 (29)	Sigmoidoscopy	Colorectal cancer mortality	4	437,600	NR	Male and female population / generally healthy (without: pre-existing colorectal cancer, adenomas (2x), other cancer (1x), increased risk for colorectal cancer, other severe or terminal disease (3x)) / setting: highly developed countries	55-74 (range)	Flexible sigmoidoscopy / flexible sigmoidoscopy with or without faecal occult blood test (1x) / sigmoidoscopy repeated after 3-5 years (1x)	No sigmoidoscopy	Mortality from colorectal cancer	Parallel	7-11.9 (median)
Brenner 2014 (29)	Sigmoidoscopy	Colorectal cancer incidence	4	437,600	NR	Male and female population / generally healthy (without: pre-existing colorectal cancer, adenomas (2x), other cancer (1x), increased risk for colorectal cancer, other severe or terminal	55-74 (range)	Flexible sigmoidoscopy / flexible sigmoidoscopy with or without faecal occult blood test (1x) / sigmoidoscopy repeated after 3-5 years (1x)	No sigmoidoscopy	Incidence of colorectal cancer	Parallel	7-11.9 years (median)

						disease (3x)) / setting: highly developed countries						
Chowdhury 2012 (78)	Omega-3	Cerebrovascular disease	2	31,181	978	Male and female population / without CVD (2x) or with: risk factors for CVD, metabolic disease (2x)	40-75 (range)	EPA 1.8 g/day capsule / EPA+DHA 1 g/day capsule / co-intervention for all patients (1x): Pravastatin 10mg/day or Simvastatin 5mg/day	Placebo containing olive oil	Cerebrovascular disease	Parallel	4.6-6.23 years follow-up (mean)
Chowdhury 2014a (79)	α-linolenic acid	Coronary heart disease	4	18,866	419	Male (2x) or male and female (2x) population / with CVD (3x) or healthy (1x) / setting: highly developed countries	40-80 (range)	α-linolenic acid / supplements or dietary oils / 2-5.5 g/day	General dietary advice / sunflower oil / EPA+DHA / margarine	Cardiovascular disease (non-fatal myocardial infarction, fatal myocardial infarction, fatal coronary heart event, sudden cardiac death, heart failure, fatal coronary heart disease)	Parallel	1-3.4 years
Chowdhury 2014a (79)	Omega-3	Coronary heart disease	17	76,580	4,974	Male and female population / with: CVD or high risk for CVD / setting: highly developed countries	18-80 (range)	Supplements (capsules) of polyunsaturated fatty acids / partly margarine (1x) / 0.3-6 g/day	Placebo / unspecified or other fatty food (sunflower oil, olive oil, corn oil, non-marine fatty-acids, margarine or ALA) / Pravastatin or Simvastatin (1x)	Fatal and non-fatal coronary events (non-fatal myocardial infarction, fatal myocardial infarction, revascularisation, fatal coronary heart disease, non-fatal coronary heart disease, acute coronary syndrome)	Parallel, factorial	0.1- 6.2 years
Chowdhury 2014a (79)	Omega-6	Coronary heart disease	8	14,476	974	Male (6x) or mixed population (2x) / with: CVD or high risk for CVD / setting: highly developed countries	30-88 (range)	Diet rich of linolenic acid / mixed polyunsaturated fatty acids principally consisting of linolenic acid (6x), linolenic acid–specific supplementation (2x) / linolenic acid intake of total daily fat 8-72% or 40,5-85g/day polyunsaurated fatty acids	Mostly unspecific dietary advice / partly low linolenic acid diet, low linoleic acid diet with high proportion of other fats	Fatal and non-fatal coronary events (revascularisation, non fatal MI, fatal MI, fatal coronary heart event, sudden cardiac death)	Parallel, cross-over cluster RCT, factorial	1-8 years

Chowdhury 2014b (80)	Vitamin D	All-cause mortality	22	30,716	5,114	Population with or without pre-existing chronic disease (CVD, metabolic disease, cancer) / community dwelling or institutionalised / setting: mostly Europe, partly North America, Asia-pacific	56-85 (mean)	Mostly oral vitamin D2 or D3 supplementation / partly injections / dose: vitamin D3: 10-6,000 IU/day, vitamin D2: 208-4,500 IU/day	Mostly placebo / partly no treatment	All-cause mortality	Parallel, cluster, factorial	0.38 to 6.8 years (mean follow-up)
Chung 2011 (58)	Vitamin D	Colorectal cancer	1	2,686	NR	Elderly general population / men (76%) and women / community dwelling	75 (mean)/ 65-85 (range)	Vitamin D3 oral supplements / 100,000 IU/4 months	Placebo	Colorectal cancer	Parallel	5 years (follow-up & duration)
Chung 2011 (58)	Vitamin D	Breast cancer	1	2,686	NR	Elderly general population, women (for outcome breast cancer) / community dwelling	75 (mean)/ 65-85 (range)	Vitamin D3 oral supplements / 100,000 IU/4 months	Placebo	Breast cancer	Parallel	5 years (follow-up & duration)
Chung 2016 (56)	Calcium	Cardiovascular mortality	2	4,103	246	Male and female elderly population / general population or population with fracture / setting: England, Scotland and Australia	77 (mean)/ >70	Calcium supplements / 1,000mg elemental calcium or 1,200 mg calcium carbonate daily for 24 months to 5 years	Placebo	Cardiovascular disease deaths, ischemic heart disease deaths	Parallel, factorial	3-9.5 years
Ding 2017 (81)	Dairy	Systolic blood pressure	8	735	NR	Male and female general population / partly: healthy or with CVD, metabolic disease, overweight	50-57 (mean for 2x)/ 20-85 (range)	Diet rich of dairy products / 3-5 servings/day (low fat milk, low-fat yogurt, unspecified dairy products daily) / free low-fat dairy products (home delivery or coupons)	Low-dairy diet / non-dairy dietary intervention / diet as usual	Systolic blood pressure mmHg, end of trial	Parallel, cross-over	1-12 months study duration
Fenton 2018 (30)	Radiation therapy	Erectile dysfunction	1	884	336	Male population with localised prostate cancer	49-69 (range)	Radiation therapy	Conservative management, including regular PSA screening, subsequent radical or palliative treatments if required	Erectile dysfunction (perceived as troublesome by patients)	Parallel	6 years

Fenton 2018 (30)	Radical Prostatectomy	Urinary incontinence	3	1,796	296	Male population with localised prostate cancer	40-75 (range)	Radical prostatectomy	Conservative management / surveillance including regular PSA screening, subsequent radical or palliative treatments if required (1x) / palliative treatment including hormon therapy, transurethral resection of the prostate and other options (1x) / observation, offered transurethral resection of the prostate in case of local progress (1x)	Urinary incontinence (defined by regular use of pads or as perceived troublesome by patients)	Parallel	2-8 years
Fenton 2018 (30)	Radical Prostatectomy	Erectile dysfunction	3	1,777	955	Male population with localised prostate cancer	40-75 (range)	Radical prostatectomy	Conservative management / surveillance including regular PSA screening, subsequent radical or palliative treatments if required (1x) / palliative treatment including hormon therapy, transurethral resection of the prostate and other options (1x) / observation, offered transurethral resection of the prostate in case of local progress (1x)	Erectile dysfunction (perceived as troublesome by patients)	Parallel	2-8 years
Filippini 2017 (43)	Disease-modifying drugs	Conversion to clinically definite multiple sclerosis	7	3,386	NR	Female (>64%) and male population / with: first attack suggestive of multiple sclerosis (and mostly with at least two silent lesions in MRI) / setting: mostly highly developed countries	28-33 (mean)/ 18-55 (range)	Different disease modifying drugs (Interferon beta-1b SC or IM (3x), Cladribine P.O. (1x), Glatiramer acetate SC (1x), Teriflunomide P.O. (1x)) / mostly treatment with corticosteroids if relapse (+/- Acetaminophen) / partly co-intervention with Acetaminophen after injection / all patients: open-label treatment with study drug if conversion to definite multiple sclerosis	Placebo (SC, IM, P.O.)	Clinically definite multiple sclerosis (defined by clinical assessment, EDSS-score, Poser criteria, confirmed by a central commitee (1x))	Parallel	18 months to 3 years

Fluri 2010 (31)	Extracranial-intracranial arterial bypass	All-cause mortality	2	1,691	281	Population with occlusive carotid artery disease / with haemodynamic compromise only (1x) or all patients with occlusion of the carotid artery irrespective of their cerebral haemodynamics (1x)	NR	Extracranial-intracranial arterial bypass plus medical treatment	Best medical treatment/ Aspirin, 325mg 4x/day or NR (1x)	All-cause death	Parallel	25-55.8 months
Fluri 2010 (31)	Extracranial-intracranial arterial bypass	Stroke	2	1,691	442	Population with occlusive carotid artery disease / with haemodynamic compromise only (1x) or all patients with occlusion of the carotid artery irrespective of their cerebral haemodynamics (1x)	NR	Extracranial-intracranial arterial bypass plus medical treatment	Best medical treatment/ Aspirin, 325mg 4x/day or NR (1x)	Any stroke	Parallel	25-55.8 months
Fluri 2010 (31)	Extracranial-intracranial arterial bypass	Stroke mortality or dependency	1	1,377	338	Population with occlusion of the carotid artery irrespective of their cerebral haemodynamics	NR	Extracranial-intracranial arterial bypass plus medical treatment	Aspirin, 325mg 4x/day	Death or dependency	Parallel	55.8 months
Gargiulo 2016 (32)	Transcatheter aortic valve	Early all-cause mortality	5	3,822	153	Male and female population with severe CVD / including patients at high surgical risk (3x) or only low to intermediate risk patients (2x) / partly with additional diseases (metabolic disease, severe kidney disease, pulmonary disease)	79-84.5 (mean)	Transcatheter aortic valve replacement / transfemoral access (primary choice), transapically only (1x) / first generation valve	Surgical aortic valve replacement	Early all-cause mortality	Parallel	<2-5 years
Gargiulo 2016 (32)	Transcatheter aortic valve	Mid-term all-cause mortality	5	3,822	563	Male and female population, mostly with severe CVD / including patients at high surgical risk (3x) or only low to intermediate risk patients (2x) / partly with additional diseases (metabolic disease, kidney disease,	79-84.5 (mean)	Transcatheter aortic valve replacement / transfemoral access (primary choice), transapically only (1x) / first generation valve	Surgical aortic valve replacement	Midterm all-cause mortality	Parallel	<2-5 years

						pulmonary disease)						
Gargiulo 2016 (32)	Transcatheter aortic valve	Long-term all-cause mortality	4	3,755	1.044	Male and female population, mostly with severe CVD / including patients at high surgical risk (2x) or only low to intermediate risk patients (2x) / partly with additional diseases (metabolic disease, kidney disease, pulmonary disease)	79-84.5 (mean)	Transcatheter aortic valve replacement / mostly by femoral access / first generation valve	Surgical aortic valve replacement	Longterm all-cause mortality	Parallel	2-5 years
Hartling 2013 (50)	Treating gestational diabetes mellitus	High birth weight	5	2,643	341	Pregnant women / mostly with mild or borderline GDM, partly with manifest GDM / blood glucose (unclear if fasting) 4.68-10.0mmol/L / ethnicity: asian, black, hispanic, white, caucasian / without: severe glucose impairment, CVD, rheumatoid disease, renal disease, active chronic diseases, prenancy with risk factors (e.g. multiple gestation)	26.3-31.1 (mean)	Dietary counselling to maintain an euglycemic diet (3 meals and 2–3 snacks; 40–55% carbohydrates), dietary therapy, e.g. calorie restriction / blood glucose monitoring and insulin administration if needed (5x)	Standard obstetric care	Birth weight >4000g	Parallel	NR

Hartling 2013 (50)	Treating gestational diabetes mellitus	Large-for-gestational age neonate	3	2,261	313	Pregnant women / borderline or mild GDM / fasting blood glucose 4.68-4.8mmol/L, BMI: 23.0-30.2 / ethnicity: asian, black, hispanic, causasian, white / without: severe GDM, complicated gestation (e.g. fetal anomaly, likely preterm delivery), or other conditions posing a risk to the pregnancy, multiple gestation (1x)	28.9-31.1 (mean)	Dietary counseling, formal diet / blood glucose monitoring and insulin if needed (3x)	Standard obstetric care	Large-for-gestational age neonate	Parallel	NR
Hartling 2013 (50)	Treating gestational diabetes mellitus	Shoulder dystocia	3	2,044	51	Pregnant women / borderline or mild GDM / fasting blood glucose 4.8mmol/L (or NR), BMI: 26.0-30.2 / ethnicity: asian, black, hispanic, white; without: severe GDM, complicated gestation (e.g. fetal anomaly, likely preterm delivery), or other conditions posing a risk to the pregnancy; without multiple gestation (1x)	26.3-30.9 (mean)	Dietary counseling, dietary therapy / blood glucose monitoring and insulin if needed (3x)	Standard obstetric care	Shoulder dystocia	Parallel	NR
Henderson 2019 (51)	Treating asymptomatic bacteriuria	Pyelonephritis	12	2,068	269	Pregnant women with asymptomatic bacteriuria / 16 to 32 weeks of gestation (range) or NR (4x) / exclusion criteria sparsely reported; (without: hypertension, renal disease, recent urinary tract infection; low risk pregnancies only (1x)) / setting: mostly highly developed countries (11x)	25.1-29 (mean)/ 10-40 (range)	Treating asymptomatic bacteriuria (≥10^5 colony forming units/ml of the same organism in 1-3 tests) / antibiotics: mostly sulfonamides (sulfamethizole, sulfadimethoxine, others), mostly with high doses used (most studies conducted between 1960-70 (10x))	No treatment for asymptomatic bacteriuria	Pyelonephritis	Parallel	NR

Higgins 2016 (25)	Bacillus Calmette-Guérin	All-cause mortality	3	3,057	199	Infants / low birth weight (2x) / without: DTP and measles vaccination, malformations or tuberculosis (1x) / setting: native americans in Canada; Guinea-Bissau; participants of factorial RCT with additional randomisation of Vitamin A and placebo (1x)	Infants; after birth or not specified	BCG vaccination / SC or not specified	No BCG vaccination (1x), delayed BCG vaccination (2x)	Mortality	Parallel, factorial	<1 year to 5 years
Higgins 2016 (25)	Measles containing vaccines	All-cause mortality	4	17,190	169	Infants or infants and children (1x) / sparse information about health status / with or without prior DTP vaccination; BCG vaccination status not specified / setting: Guinea-Bissau, Nigeria; participants of factorial RCT with additional randomisation of Vitamin A and placebo (1x)	4.5 months to 2 years (range)	Measles containing vaccine / one or two doses / at 4.5, 6 or 9 months / vaccine: monovalent, strains: Edmonston, Schwarz, Enders B	Delayed measles containing vaccine (1x) / polio vaccine (2x) / pertussis/tetanus vaccines (1x)	Mortality	Parallel, factorial	<1.5 years to <5 years/ for analysis: 9 months (reported in systematic review)
Hopley 2010 (33)	Total hip arthroplasty	Reoperation	4	421	27	Mostly female population / with: displaced femoral neck fracture / ambulatory and oriented patients (2x) or patients with considerable cognitive impairment (2x) (mini-mental-status-test 10/ SPMSQ-score 9)	74-82 (mean)	Total hip arthroplasty, cemented (3x) / cemented or uncemented (1x)	Bipolar cemented hemiarthroplasty (3x) / uni- or bipolar and cemented or uncemented (1x)	Reoperation	Parallel	1-3.33 years
Hopley 2010 (33)	Total hip arthroplasty	Dislocation	4	421	10	Mostly female population / with: displaced femoral neck fracture / ambulatory and oriented patients (2x) or patients with considerable cognitive impairment (2x) (mini-mental-status-test 10/ SPMSQ-score 9)	74-82 (mean)	Total hip arthroplasty, cemented (3x) / cemented or uncemented (1x)	Bipolar cemented hemiarthroplasty (3x) / uni- or bipolar and cemented or uncemented (1x)	Dislocation	Parallel	1-3.33 years

Hopley 2010 (33)	Total hip arthroplasty	Deep infection	4	421	17	Mostly female population / with: displaced femoral neck fracture / ambulatory and oriented patients (2x) or patients with considerable cognitive impairment (2x) (mini-mental-status-test 10/ SPMSQ-score 9)	74-82 (mean)	Total hip arthroplasty, cemented (3x) / cemented or uncemented (1x)	Bipolar cemented hemiarthroplasty (3x) / uni- or bipolar and cemented or uncemented (1x)	Deep infections	Parallel	1-3.33 years
Hüpfl 2010 (67)	Chest-compression-only cardiopulmonary resuscitation	All-cause mortality	3	3,031	389	Population with out-of hospital cardiac arrest	NR	Instructions for chest-compression-only bystander cardiopulmonary resuscitation	Instructions for standard bystander cardiopulmonary resuscitation (including rescue ventilation)	All-cause mortality	Parallel	Hospital discharge to 30 days (range)
Jamal 2013 (82)	Non-calcium-based phosphat binders	All-cause mortality	8	4,340	936	Male and female population / with: advanced chronic kidney disease, mostly requiring haemodialysis (7x) / without: gastrointestinal disease (4x), cancer (2x), diabetes or uncontrolled diabetes (4x), CVD (4x)	47-60 (mean)	Non-calcium-based phosphat binders (Sevelamer or Lanthanum) / Atorvastatin for intervention and control group (1x)	Calcium-based phosphat binders (calcium acetate or calcium carbonat)	All-cause mortality	Parallel	5 to >44 months
Jefferson 2010 (46)	Parenteral influenza vaccine	Influenza-like illness	4	6,894	346	Elderly population / community-dwelling generally healthy (2x) or healthy elderly in a retirement community (1x) patients in a psychiatric hospital (1x) / setting: Europe and USA	>52/ NR (1x)	Parenteral influenza vaccines / monovalent or trivalent, live attenuated or inactivated / matching the circulating strains during outbreak / pneumococcal vaccines for intervention and control group (1x)	Placebo / influenza B vaccine, placebo or no vaccine (1x)	Influenza like illness, clinically defined (e.g. sudden onset of fever, cough, prostration, weakness, myalgia, widespread aches, headache or myalgia)	Parallel	3-6 months

Jefferson 2010 (46)	Parenteral influenza vaccine	Influenza	3	2,217	89	Elderly population / health status: generally healthy community-dwellers (1x), institutionalised population in a psychiatric hospital (1x) or nursing home residents (1x) / without: severe illness or immunocompromised (1x) / setting: Russia, USA, Netherlands	73 (median)/ >60/ 41-95 (range)/ NR (1x)	Parenteral influenza vaccine / monovalent or trivalent, live attenuated or inactivated / vaccine strains matching the circulating strains during outbreak, partly not during outbreak (1x)	Placebo	Laboratory confirmed influenza	Parallel	1-6 months
Jefferson 2012 (34)	Inactivated influenza vaccines	Influenza	5	1,628	252	Male and female children and adolescents / known to be healthy (2x) or health status NR	<3-18 (range)	Inactivated influenza vaccines / 1-2 doses, bi- or trivalent / intranasally, IM / (RCTs conducted 1990-2003)	Placebo	Culture confirmed influenza (3x), clinical illness assessed by examination (1x), confirmed by subsequent illness of household member, illness in the epidemic period (1x)	Parallel, cluster-RCT (randomization by family)	4-6 months/ NR (4x)
Jefferson 2012 (34)	Inactivated influenza vaccines	Influenza-like illness	5	19,388	4,996	Healthy male and female children and adolescents / partly without: previous hypersensitivity reactions	<3-18 (range)	Inactivated influenza vaccines / 1-2 doses, bi- or trivalent / intranasally, IM, SC / (RCTs conducted 1990-2001)	Placebo / no treatment (1x)	Influenza-like illness (clinically defined)	Parallel, cluster-RCT (clusters: family, school, class)	<5 months
Jin 2012 (83)	Total flavonoids	Colorectal neoplasms	1	929	358	Male and female population with colorectal adenoma / without: other gastrointestinal diseases, severe overweight / setting: USA	>35	Diet with low fat and high amount of fibre, fruit and vegetables	Control diet (as usual)	Colorectal adenoma recurrence	Parallel	NR
Johnston 2019 (23)	Low red meat	All-cause mortality	1	48,835	NR	Female postmenopausal population / with: overweight or obesity (>70%), hypertension (30-40%) / without: cancer, type-1 diabetes, infaust disease	50-79 (range)	Behavioral support to achieve diet with low fat and high amount of vegetables, fruit and grain	General dietary advice by educational materials	All-cause mortality	Parallel	<17.05 years follow-up

Johnston 2019 (23)	Low red meat	Cardiovascular mortality	1	48,835	NR	Female postmenopausal population / with: overweight or obesity (>70%), hypertension (30-40%) / without: cancer, type-1 diabetes, infaust disease	50-79 (range)	Behavioral support to achieve diet with low fat and high amount of vegetables, fruit and grain	General dietary advice by educational materials	Cardiovascular mortality	Parallel	< 13.8 years follow-up
Johnston 2019 (23)	Low red meat	Cardiovascular disease	1	48,835	NR	Female postmenopausal population / with: overweight or obesity (>70%), hypertension (30-40%) / without: cancer, type-1 diabetes, infaust disease	50-79 (range)	Behavioral support to achieve diet with low fat and high amount of vegetables, fruit and grain	General dietary advice by educational materials	Cardiovascular disease	Parallel	< 13.8 years follow-up
Kansagara 2013 (52)	Transfusion	All-cause mortality	6	1,757	NR	Male and female population / with: anemia, mostly with cardiovascular disease (4x) / population in non-operative setting with CHD (3x) or undergoing orthopaedic (2x) or major vascular surgery (1x) / setting: USA and Canada	64-83.3 (mean)	Liberal transfusion protocols with transfusions starting at haemoglobin values ≤ 10g/dL	More restrictive transfusion protocols with transfusions starting at haemoglobin values ≤7-9 g/dL	Mortality at 30 days	Parallel	>30 days
Keag 2018 (84)	Caesarean section	Urinary incontinence	1	2,088	NR	Pregnant women, general population / singleton living fetus in breech presentation, 50% primipara (with or without prior vaginal delivery) / without further contraindication to vaginal delivery (e.g. fetal weight >4 kg, feto-pelvic dysproportion, placenta previa) / setting: highly developed countries	≈30% > 30	Caesarean section (planned)	Trial of vaginal delivery, assisted by experienced clinician (followed by caesarean section if unsuccesful)	Urinary incontinence	Parallel	2 years

Keag 2018 (84)	Caesarean section	Fecal incontinence	1	2,088	NR	Pregnant women, general population / singleton living fetus in breech presentation, 50% primipara (with or without prior vaginal delivery) / without further contraindication to vaginal delivery (e.g. fetal weight >4 kg, feto-pelvic dysproportion, placenta previa) / setting: highly developed countries	≈30% > 30	Caesarean section (planned)	Trial of vaginal delivery, assisted by experienced clinician (followed by caesarean section if unsuccesful)	Fecal incontinence	Parallel	2 years
Kredo 2014 (85)	Starting and maintaining antiretroviral therapy	All-cause mortality	1	2,770	523	Male and female adult population (study cohort I of Fairall 2012) / either eligible for ART (HIV infection with CD4 ≤200 cells/μL) or likely to become eligible for ART (with CD4 201–350 cells/μL), 32-52% WHO stage I (asymptomatic) / setting: rural and periurban South Africa	35-36 (mean)/ >16	ART task-shifting from doctors to nurses / ART initiation and maintenance by nurses and referral to doctors in complicated cases (e.g. very low CD4, pregnant, previous ART) / including various ART regimens	ART initiation and re-prescription by doctors with concomitant care by nurse	Death at 12 months	Cluster-RCT	1-1.5 years duration (range)
Kredo 2014 (85)	Starting and maintaining antiretroviral therapy	Attrition	1	2,770	180	Male and female adult population (study cohort I of Fairall 2012) / either eligible for ART (HIV infection with CD4 ≤200 cells/μL) or likely to become eligible for ART (with CD4 201–350 cells/μL), 32-52% WHO stage I (asymptomatic) / setting: rural and periurban South Africa	35-36 (mean)/ >16	ART task-shifting from doctors to nurses / ART initiation and maintenance by nurses and referral to doctors in complicated cases (e.g. very low CD4, pregnant, previous ART) / including various ART regimens	ART initiation and re-prescription by doctors with concomitant care by nurse	Lost to follow-up at 12 months	Cluster-RCT	1-1.5 years duration (range)

Kredo 2014 (85)	Maintaining antiretroviral therapy	All-cause mortality	2	4,332	94	Male and female adult population / (study cohort II of Fairall 2012) receiving ART for 6 months, 78-79% viral load <400 copies/ml (1x) or population with advanced HIV (CD4 < 350 cells/µL) recently started ART and 35% with AIDS defining event, median CD4 164-165 cells/µL (1x) / setting: rural and periurban South Africa	32.2-38 (mean)/ >16	ART task-shifting from doctors to nurses / maintenance of ART by trained nurses / various ART regimens	ART maintenance from doctors with or without prior experience in the field / concomitant care by nurse (1x)	Death at 12 months	Parallel, cluster-RCT	1-1.5 years duration (range); median 120 weeks duration
Li 2014 (54)	Exenatide	Acute pancreatitis	5	3,998	7	Male and female population with diabetes / mean BMI 30.5-32.5, mean HbA1c 7.5-8.5%, mean diabetes duration 4.9-9.6 years / included only population already treated with Metformin with or without Sulfonylureas / mostly (4x): population with inadequate glycaemic control only	52.3-58.9 (mean)	Exenatide / dosing: 2mg/week (2x); 5µg twice/day for 4 weeks followed by 10µg twice/day for the remaining study duration (3x)	Other antidiabetic drug (Pioglitazone, Insulin glargine, Glimepiride)	Pancreatitis	Parallel	26-234 weeks (range)
Li 2016 (53)	DDP-4 inhibitors	Heart failure	34	26,368	75	Male and female population with diabetes / mean BMI 24.0-32.8, mean baseline HbA1c 7.1-9.9%, mean/median diabetes duration 1.7-17.5 years / mostly without prevalent CVD / mostly population already receiving antidiabetic treatment with different drugs: Metformin, Pioglitazone, Various oral antidiabetics with or without Insulin, Glyburide, Voglibose	49.7-72.6 (mean)	DPP-4 inhibitors, one per study (Sitagliptin, Vildagliptin, Linagliptin, Saxagliptin or Alogliptin) / mostly as add-on therapy, partly as monotherapy	Active drug (including Sulfonylureas and SGLT-2 inhibitors) or placebo	Heart failure	Parallel, factorial	12-206 weeks

Li 2016 (53)	DDP-4 inhibitors	Hospital admission for heart failure	5	37,028	1,174	Male and female population with diabetes / mostly with CVD, risk factors for CVD or with concomitant kidney disease / mean BMI 29.5-31.1, mean HbA1c level 7.8-8.1%, mean duration of diabetes 9.2-11.6 years / population receiving already standard diabetic therapy	60.9-66.6 (mean)	DPP-4 inhibitors, one per study (Sitagliptin, Vildagliptin, Linagliptin, Saxagliptin or Alogliptin) / as add-on to standard therapy	Placebo; placebo or glimepiride (1x)	Hospital admission for heart failure	Parallel	52-156 weeks
Matthews 2018 (86)	Tamoxifen	Heart failure	1	9,766	NR	Female post-menopausal population / with histologically confirmed breast adenocarcinoma and completed local treatment with curative intension / with or without involvement of lymph nodes, oestrogen-receptor-positive or progesterone-receptor-positive disease / population without: evidence of metastatic disease, severe CHD, other cancers	64 (median)	Tamoxifen 20mg/day for 2-3 years followed by Exemestane 25mg/day until a total of 5 years of treatment	Exemestane 25mg/day for 5 years	Heart failure	Parallel	5.1 years (median)
Menne 2019 (87)	SGLT-2 inhibitors	Acute kidney injury	41	68,159	1,089	Population with diabetes (mostly type II) / mostly receiving background therapy with other anti-diabetic drugs (Insulin, Metformin, DDP-4 Inhibitors, Sulfonylureas, other unspecified oral antidiabetics, standard care) / partly population with: drug-naive diabetes, type-I diabetes, CVD, including/excluding definite or advanced renal insufficiency (e.g. eGFR <30, <50 or 60)	NR	SGLT-2 Inhibitors, one per study (Bexagliflozin, Canagliflozin, Dapagliflozin, Empagliflozin, Ertugliflozin)	Placebo or active drug / drugs: mostly DDP-4 Inhibitors or Sulfonylureas; partly: Semaglutide (1x), Metformin plus insulin (1x)	Any acute kidney injury (serious and non-serious); acute renal failure, renal impairment, renal failure (acute or chronic change), increase in creatinine or decline of eGFR	Parallel, factorial	12 weeks-296 weeks study duration

Mesgarpour 2017 (88)	Erythropoiesis stimulating agents	Venous thromboembolism	12	4,874	373	Male and female adult population / critically-ill at ICU and mostly with anemia (10x) / various admission reasons (e.g. multidisciplinary ICU, medical or surgical ICU for trauma, post-operative, burns, patients with PCI for STEMI)	60.9 (mean)/ ≥18/ 15-70 (range)	Erythropoiesis stimulating agents / drugs and dosing: repeated administration of SC rHuEPO 300 IU/kg; SC injection of rHuEPO 40,000 IU at study days 1, 7, 14; SC injection of Epoetin alfa 40,000 IU at days 1, 8, 15; IV injection of Erythropoietin-beta 500 IU/kg to a maximum of 50,000 IU; two doses SC rHuEPO 40,000 IU and Iron saccharate; IV epoetin alfa 40,000 IU and IV Iron; SC Epoetin alfa 10,000-40,000 IU for 12 weeks or until Hb >12g/dl; Epoetin alfa 40,000 IU weekly up to 3 doses; IV EPO 40,000 IU single bolus; IV Epo 500 IU/kg 1-3 doses; IV rHuEPO 150-300 IU/kg daily for up to 30 days; IV Epoetin alfa 60,000 IU single bolus	Placebo, IV iron saccharate (1x), standard care (1x), sodium chloride (1x) or not reported (4x)	Venous thromboembolism	Parallel, factorial	5 days to 12 months
Mesgarpour 2017 (88)	Erythropoiesis stimulating agents	All-cause mortality	17	4,546	NR	Male and female adult population / critically-ill and mostly at ICU with anemia (16x) / various admission reasons (trauma including traumatic brain injury, critical state after major cardiac or cardiothoracic or abdominal surgery, anemia and sepsis, severely burned) / partly population in long-term acute care hospitals (1x)	15-70 (range)	Recombinant Erythropoietin-ß or Epoetin alfa for haematopoetic indications / intravenous or subcutaneous administration / doses: 300 to 600 IU/kg (2000 to 50,000 IU), 1 to 12 doses / co-interventions (for all patients): IV Iron (2x), IV folic acid 1mg/day + IV Iron saccharate (1x)	Placebo (saline (3x) or not specified), usual care (1x)	Mortality; assessment at different time points after intervention ranging from in-hospital mortality to 6 months; mostly longer than 10 days	Parallel, factorial	5 days to 6 months

Moberley 2013 (89)	Pneumococcal polysaccharide vaccines	Invasive pneumococcal disease	10	35,893	78	Male and female adult population / institutionalised or community dwelling / with: risk factors for pneumococcal disease (age or other risk factors) / mostly immunocompetent / risk factors: advanced age, pulmonary disease (e.g. COPD, bronchogenic carcinoma), institutionalised, environmental factors (population in low-income countries) / partly without: cancer, organ dysfunctions, immobility	>40/ 42-106 (range)	Different types of pneumococcal vaccines: 2- or 3-valent PPV, 12-valent PPV, 14-valent PPV, 17-valent PPV, 23-valent PPV / partly: administered with influenza as co-intervention for all patients (1x)	Mostly placebo or no vaccine / partly: influenza vaccine for all patients (1x)	Invasive pneumoccal disease	Parallel, cluster-RCT	2-2.9 years (mean)/ 0.5-3 years (range)
Molnar 2015 (35)	Neoral (Cyclosporin)	Acute rejection of kidney transplant	2	273	43	Adult population with kidney transplant / differing status of transplant: older transplant with stable graft function (transplanted between 1 and 10 years prior to enrollment, no rejections in the past 6 months) or all incident transplants / setting: Iran and USA	38.1-39.3 (mean)/ NR (1x)	Neoral (Cyclosporin)	Iminoral, Consupren (generic Cyclosporin)	Acute rejection of kidney transplant	Parallel	1 year
Navarese 2013 (90)	Early intervention for NSTE-ACS	All-cause mortality	7	5,370	230	Male and female population / undergoing PCI, CABG or medical treatment for ACS / with or without: metabolic disease, positive cardiac biomarkers, ST-segment depression, three-vessel CAD, use of Glycoprotein IIb/IIIa inhibitors / setting: mainly European (6x)	62-70 (mean)	Early intervention for NSTE-ACS / time to intervention 0.5-14 hours	Delayed strategy / time to intervention 20.5-86 hours	Mortality	Parallel	1-6 months (follow-up)

Navarese 2013 (90)	Early intervention for NSTE-ACS	Myocardial infarction	7	5,340	408	Male and female population / undergoing PCI, CABG or medical treatment for ACS / with or without: metabolic disease, positive cardiac biomarkers, ST-segment depression, three-vessel CAD, use of Glycoprotein IIb/IIIa inhibitors / setting: mainly European (6x)	62-70 (mean)	Early intervention for NSTE-ACS / time to intervention 0.5-14 hours	Delayed strategy / time to intervention 20.5-86 hours	Myocardial infarction	Parallel	1-6 months (follow-up)
Navarese 2013 (90)	Early intervention for NSTE-ACS	Major bleeding	7	5,370	173	Male and female population; undergoing PCI, CABG or medical treatment for ACS / with or without: metabolic disease, positive cardiac biomarkers, ST-segment depression, three-vessel CAD, use of Glycoprotein IIb/IIIa inhibitors / setting mainly European (6x)	62-70 (mean)	Early intervention for NSTE-ACS / time to intervention 0.5-14 hours	Delayed strategy / time to intervention 20.5-86 hours	Major bleeding	Parallel	1-6 months (follow-up)
Nelson 2010 (36)	Caesarean section	Anal incontinence, feces	1	1,226	NR	Pregnant women, general population / singleton living fetus in breech presentation, 50% primipara (with or without prior vaginal delivery) / without further contraindication to vaginal delivery (e.g. fetal weight >4 kg, feto-pelvic dysproportion, placenta previa) / setting: highly developed countries	≈30% > 30	Caesarean section (planned)	Trial of vaginal labour	Anal incontinence, feces	Parallel	3 months to 2 years

Nelson 2010 (36)	Caesarean section	Anal incontinence, flatus	1	1,226	NR	Pregnant women, general population / singleton living fetus in breech presentation, 50% primipara (with or without prior vaginal delivery) / without further contraindication to vaginal delivery (e.g. fetal weight >4 kg, feto-pelvic dysproportion, placenta previa) / setting: highly developed countries	≈30% > 30	Caesarean section (planned)	Trial of vaginal labour	Anal incontinence, flatus	Parallel	3 months
Nieuwenhuijse 2014 (37)	Ceramic-on-ceramic bearings for total hip arthroplasty	Harris Hip Score	7	1,334	NR	Male and female population, mostly with osteoarthritis (range 72-77% or NR) (other indications for arthroplasty: avascular necrosis, fracture) / mostly active or young populations only (6x) or broader populations with advanced age and hip fracture	45.3-72.7 (mean)/ 12-76 (range)	Ceramic-on-ceramic bearings for total hip arthroplasty	Metal-on-polyethylene or ceramic-on-polyethylene bearings for hip arthroplasty	Harris-Hip score at short-, mid-, or long-term	Parallel	2-12.4 years (mean)
Nieuwenhuijse 2014 (37)	High-flexion total knee arthroplasty	Flexion	20	2,042	NR	Male and female population / mostly with osteoarthritis / all patients or only active patients	61.4-70.6 (mean)/ 43-86 (range)	High flexion prosthesis for total knee arthroplasty	Standard prosthesis for total knee arthroplasty	Flexion in degrees at short-, mid-, or long-term	Parallel	1-11 years (mean)
Nieuwenhuijse 2014 (37)	Gender-specific total knee arthroplasty	Flexion-extension range	6	866	NR	Female population with osteoarthritis / partly without: CVD (1x)	66-71.2 (mean)/ 47-87 (range)	Gender-specific prosthesis for total knee arthroplasty	Non-gender specific prosthesis for total knee arthroplasty	Flexion-extension range of motion, short-term	Parallel	6 weeks to 3.25 years (mean)
Nikooie 2019 (55)	Second generation antipsychotics	Sedation	6	872	119	Male and female population with delirium / inpatients with or without critical illness (ICU, mechanical ventilation) / partly without: dementia, substance-induced delirium	44-67 (mean)	Second generation antipsychotic / any drug or specific: Olanzapine 3.1-5.5 mg/day; Quetiapine 67.6 mg/day; Risperidone 1 mg/day; Ziprasidone 20mg/day (mean doses)	Haloperidol / mean dose: 0.8-11 mg/day	Sedation outcomes: Sleepiness, excessive/severe sedation, somnolence, hypersomnia, oversedation	Parallel	NR

Nikooie 2019 (55)	Second generation antipsychotics	Neurologic outcomes	6	869	19	Male and female population with delirium / inpatients with or without critical illness (ICU, mechanical ventilation) / partly without: dementia, substance-induced delirium	44-67 (mean)	Second generation antipsychotic /any drug or specific: Olanzapine 3.1-5.5 mg/day; Quetiapine 67.6 mg/day; Risperidone 1 mg/day; Ziprasidone 20-113 mg/day (mean doses)	Haloperidol / mean/median dose: 0.8-15 mg/d	Extrapyramidal symptoms, dystonia, akathisia, tremors, rigidity, tics	Parallel	NR
Ochen 2019 (91)	Surgery for achilles tendon rupture	Re-rupture	10	944	70	Mostly male (≈83%) young adult and middle-aged population with acute achilles tendon rupture / setting: highly developed countries	37.2-41.8 (mean)/ 21-77 (range)	Surgical Achilles tendon repair / open surgery; minimal-invasive procedure (1x)	Nonoperative treatment / cast, brace or orthosis for 7-10 weeks, with or without early weight bearing; partly with early functional rehabilitation	Re-rupture	Parallel	12-30 months (mean)
Ochen 2019 (91)	Surgery for achilles tendon rupture	Complications	9	894	128	Mostly male (≈80%) young adult and middle-aged population with acute achilles tendon rupture / setting: highly developed countries	37.2-41.8 (mean)/ 21-77 (range)	Surgical Achilles tendon repair / open surgery; minimal-invasive procedure (1x)	Nonoperative treatment / cast, brace or orthosis for 7-10 weeks, with or without early weight bearing; partly with early functional rehabilitation	Complications (Infections, Thrombosis and others)	Parallel	12-30 months (mean)
Pittas 2010 (60)	Vitamin D	Hypertension	1	17,122	NR	Female population / postmenopausal without hypertension / setting: USA	50-79 (range)	Supplements / vitamin D3 400 IU/day and calcium carbonate 1000 mg/day	Placebo	Incident hypertension (self-reported by participants)	Parallel	7 years (duration)
Raman 2013 (38)	Carotid endarterectomy	Ipsilateral stroke	3	5,223	308	Male and female population with asymptomatic carotid artery stenosis / without: severe disease (3x) (likely leading to death or death and disability in next 5 years/ precluding long-term follow-up), contralateral cerebrovascular symptoms (2x), lesions too complicated for surgery (2x), history of CHD (1x) / with or without: hypertension, metabolic disease, smokers and non-smokers	64.1-68 (mean)/ 40-91 (range)	Carotid endarterectomy / additional medical therapy according to physician or local standard (Aspirin 325mg/day to 650mg/2x daily)	Medical therapy according to physician or local standard (Aspirin 325mg/d to 650mg/2x daily) / with or without deferred carotid endarterectomy if symptoms occur / additional modification of cardiovascular risk factors (not meeting current standards): treatment of hypertension and diabetes, partly lipid lowering agents	Ipsilateral stroke (including any stroke within 30 days)	Parallel	4.4-9 years (median)

Raman 2013 (38)	Carotid endarterectomy	Stroke	3	5,223	508	Male and female population with asymptomatic carotid artery stenosis / without: severe disease (3x) (likely leading to death or death and disability in next 5 years/ precluding long-term follow-up), contralateral cerebrovascular symptoms (2x), lesions too complicated for surgery (2x), history of CHD (1x) / with or without: hypertension, metabolic disease, smokers and non-smokers	64.1-68 (mean)/ 40-91 (range)	Carotid endarterectomy / additional medical therapy according to physician or local standard (Aspirin 325mg/day to 650mg/2x daily)	Medical therapy according to physician or local standard (Aspirin 325mg/d to 650mg/2x daily) / with or without deferred carotid endarterectomy if symptoms occur / additional modification of cardiovascular risk factors (not meeting current standards): treatment of hypertension and diabetes, partly lipid lowering agents	Any stroke (including any death within 30 days) / defined as events of perioperative stroke or death or any nonperioperative territory stroke	Parallel	4.4-9 years (median)
Raman 2013 (38)	Carotid artery stenting	Periprocedural stroke	2	1,418	33	Male and female population with asymptomatic carotid stenosis / without: acute CHD/ arrythmia (1x) / with or without: hypertension, metabolic disease, smokers and non-smokers, previous severe stroke (1x), stroke within 48 hours (1x), at least one high risk factor (e.g. severe CHD, severe pulmonary disease)	69.3-72.5 (mean)	Carotid artery stenting (by experienced surgeon at tertiary care center) plus dual antiplatelet therapy (Aspirin plus Thienopyridines)	Carotid endarterectomy (by experienced surgeon at tertiary care center) plus medical therapy / medical therapy: single agent antiplatelet treatment (Aspirin or Thienopyridines) or combination of Aspirin and Extended-release Dipyridamole	Any periprocedural stroke	Parallel	3-4 years (range)
Schweizer 2013 (39)	Nasal deconolization	Surgical site infection	5	3,029	136	Male and female population / inpatients at orthopaedic or cardiac surgery ward / setting: USA, Canada and The Netherlands	NR	Nasal Mupirocin alone or with Chlorhexidine soap (1x) / dose: 2% or 2.15% Mupirocin / start: 5-7 days preoperatively (2x) or not reported / treatment regardless of carrier status (MRSA/ MSSA) or for MRSA/ MSSA carriers only (2x)	Placebo	Wound infections, mostly assessed by Center for Disease Control criteria	Parallel	NR

Schweizer 2013 (39)	Glycopeptide prophylaxis	Surgical site infection	8	6,379	282	Male and female population / mostly cardiac surgery patients, orthopaedic surgery (1x)	NR	Prophylactic treatment with Vancomycin or Teicoplanin / alone or with Rifampicin, Gentamicin or Ticarcillin/ Clavulanate	Prophylactic treatment with Cephalosporins (Cefuroxime, Cefazolin, Ceftriaxone)	Wound infections, mostly assessed by Center for Disease Control criteria	Parallel	30 days to 6 months, mostly not specified
Silvain 2012 (40)	Enoxaparin	All-cause mortality	6	14,749	299	Mostly male population with CAD/ CHD / mostly with MI (4x) and undergoing PCI or elective PCI only (2x) / with: postfibrinolytic PCI only (2x) / without: MI complicated by cardiogenic shock (2x) / "real world" MI population (1x) / with or without: metabolic disease	NR	Enoxaparin / SC or IV / dosing: 0.5-1mg/kg, single bolus or repeated administration regimens (every 12 hours) / with or without: additional bolus during PCI / additional unfractionated Heparin during PCI (1x) / mostly additional anticoagulation (e.g. Aspirin, Clopdigrel) for all patients	Unfractionated Heparin / IV / 50-100 IU/kg bolus with or without Glycoprotein IIb/IIIa inhibitors / with or without continued administration after bolus (12 IU/kg/h or titration to activated clotting time 200-300 seconds)	All-cause mortality	Parallel	In-hospital to 12 months
Silvain 2012 (40)	Enoxaparin	Major bleeding	9	15,946	372	Mostly male population with CAD/ CHD / with: elective PCI only (5x) or urgent PCI for MI (4x) / with: postfibrinolytic PCI (2x) / "real world" MI population (1x) / without: MI complicated by cardiogenic shock (3x), increased risk for bleeding (1x), previous anticoagulation (5x) / with or without: metabolic disease	NR	Enoxaparin / SC or IV / dosing: 0.5-1mg/kg; single bolus or repeated administration regimens (every 12 hours) / with or without: additional bolus during PCI / additional unfractionated Heparin during PCI (1x) / mostly additional anticoagulation (e.g. Aspirin, Clopdigrel) for all patients	Unfractionated Heparin / IV / 50-100 IU/kg or 10,000 IU bolus with or without Glycoprotein IIb/IIIa inhibitors (if these were adminstered, the Heparin dose was usually reduced) / with or without continued administration after bolus (12 IU/kg/h or titration to activated clotting time 200-300 seconds)	Major bleeding (STEEPLE, TIMI or individual study definition)	Parallel	In-hospital to 12 months
Silvain 2012 (40)	Enoxaparin	All-cause mortality or myocardial infarction	13	15,733	1,520	Mostly male population with CAD/ CHD / elective (9x) or urgent PCI (4x) / with: postfibrinolytic PCI (1x) / "real world" MI population (1x) / without: MI complicated by cardiogenic shock (2x), prior thrombolysis or anticoagulation (6x) / with or without:	NR	Enoxaparin / SC or IV / dosing: 0.5-1mg/kg; single bolus or repeated administration regimens (every 12 hours) / with or without: additional bolus during PCI / additional Abciximab (1x) / mostly additional anticoagulation (e.g. Aspirin, Clopdigrel) for all patients	Unfractionated Heparin / IV / 50-100 IU/kg or 10,000 IU bolus with or without Glycoprotein IIb/IIIa inhibitors (if these were adminstered, the Heparin dose was usually reduced) / with or without continued administration after bolus (12 IU/kg/h or titration to activated clotting time 200-300	Death or Myocardial infarction	Parallel	In-hospital to 1 month

						metabolic disease			seconds)			
Suthar 2012 (26)	Antiretroviral therapy	Tuberculosis infection	2	2,536	104	Male and female adult population with HIV, CD4 count: 200-550 cells/µL (range), 280-442 cells/µL (median) / BMI 21.0-21.3 (median) / without: preexisting advanced HIV infection (previous or current AIDS defining disease or HIV infection WHO stage 4) and previous exposure to ART (2x) / setting: developing countries	≥18	Immediate ART with ≥3 antiretroviral drugs / ART: ≥3 of any antiretroviral drugs (1x) or two nucleoside reverse transcriptase inhibitors plus either a non-nucleoside reverse transcriptase inhibitor or a protease inhibitor (1x)	Delayed ART initiated at decline of CD4 count to 200-250 cells/µL (1x) or when clinical AIDS developed (1x)	Tuberculosis infection (AIDS clinical trials group definition, American Thoracic society case definition)	Parallel, randomization of couples (1x)	20.4-21 months median follow-up
Te Morenga 2013 (61)	Sugar	Weight gain	10	509	NR	Male and female population / healthy (5x) or with: overweight or obesity (4x) post-obese (1x) / additionally (for non-healthy): metabolic disease (1x), disease of the gallbladder (1x) or CVD risk factors (1x)	20-55 (range)	High-sugar diet with sugar-rich (glucose, fructose, sucrose) food, drinks or both / sugar in foods: 80 to >100g/day or 28% of total energy / sugar sweetened beverages: 1L or 1135 g/day / partly: additional fibre depletion (1x), low fat (1x)	Diet with high amount of complex carbohydrates (e.g. starch), artificial sweeteners or low to moderate sugars (10-40g/day) / additionally: partly fibre depletion (1x), low fat (1x)	Weight gain	Parallel, cross-over	2 weeks to 6 months
Te Morenga 2013 (61)	Sugar	Body Mass Index	3	1,627	NR	Male and female schoolchildren (2x) (elementary school) and adolescents (1x) / general population / without: smokers, major medical illness, eating disorder (BMI <25th age percentile) (1x)	7-18 (range)	General dietary advice, nutrition education without specific advice regarding reduction of sugars or no intervention	Reduced sugar intake / intervention descriptions: counselling to reduce sugar-sweetened beverages, home deliveries of non-caloric beverages (4 servings/day) (1x); nutrition education to reduce fat and sugar intake and increase intake of complex carbohydrates (1x); behavioural classroom intervention to reduce intake of sugar sweetened beverages (10x 1-hour sessions) (1x)	Change in BMI	Parallel, cluster-RCT	25 weeks-8 months (duration)

Thomas 2010 (92)	Influenza vaccines	Influenza-like illness	3	7,031	688	Population (included for intervention): mostly female (≈70%) health care workers / setting: nursing homes or geriatric long-stay hospitals in UK or France with elderly population with varying health status / 0-80% of residents with influenza vaccination per facility	77-86 (mean)	Actively promoting influenza vaccination for health care workers / methods: offering vaccination or actively promoting vaccination (informational material, specially trained nurses, interviews)	General information about influenza vaccination or not offering vaccination to health care workers	Influenza like illness in residents, clinical definition (fever or an acute deterioration in physical or mental ability plus typical symptoms of a viral respiratory infection)	Cluster-RCT	5 months or during influenza season (follow-up)
Tickell-Painter 2017 (93)	Mefloquine	Discontinuation due to adverse effects	3	1,438	52	Mostly healthy adult short-term travellers from high income countries / without: alcoholism, psychiatric or severe neurologic disorders, drug hypersensitivity, pregnancy or lacation, severe blood disorders, organ dysfunctions, previous malaria, recent stay in endemic area / included children (1x)	33-35.3 (mean)/ 3-70 (range)	Mefloquine / dose: 250mg/week (less for children according to weight) / duration: 1-3 weeks before to 4 weeks after travel	Atovaquone-proguanil / dose: 250mg/day (for children according to weight) / duration: 1-17 days before to 1 week after travelling	Discontinuation of study drug due to adverse effects	Parallel	7-60 days follow-up/ Mean duration of exposure to malaria: 2.5 weeks or 19 days (mean), 1-3 weeks (range)
Tickell-Painter 2017 (93)	Mefloquine	Serious adverse events or effects	3	747	3	Generally healthy male population from Ivory Coast and Thailand / pregnant women from Thailand (1x)	16-60 (range)	Mefloquine / dosing: 250mg/week for 4 weeks followed by maintenance dose of 125mg/week / additional loading dose of 500mg (1x)	Placebo	Serious adverse events, including childhood deaths (1x)	Parallel	20-24 weeks, NR (1x)
Tickell-Painter 2017 (93)	Mefloquine	Nausea	2	244	119	Diverse adult population / pregnant Thai women in malaria-endemic area (presumed semi-immune) and non-pregnant healthy Dutch volunteers (not exposed to malaria)	Pregnant women; adults	Mefloquine / dosing: 250 mg/week for 4 weeks, then 125 mg/week until delivery, with or without 500 mg loading dose (1x) or 250 mg tablet weekly with loading dose (one tablet daily for 3 days in week 1)(1x)	Placebo	Nausea	Parallel	30 days to 20 weeks (range)

Tricco 2018 (45)	Live-attenuated zoster vaccines	Suspected Herpes Zoster	5	62,529	1.597	Male and female population / mostly immunocompetent population (e.g. no use of steroids), partly: immunocompromised (1x) / with: previous chickenpox / mostly without: previous herpes-zoster	66.2 (mean)-69.0 (median)	Live-attenuated zoster vaccines (Zostavax, Oka/Merck); Zostavax + Pneumovax 23 (1x) / one dose	Placebo; Placebo plus Pneumovax 23 vaccine and delayed zoster vaccination (1/5)	Suspected herpes zoster	Parallel	2-65 months (range); follow-up of 2 months in one study applying delayed vaccination
Vinceti 2018 (59)	Selenium	Cancer	5	21,860	2.332	Population with diverse health status / without: prostate cancer, haemorrhagic stroke, hypertension or with: high risk for prostate cancer, completely resected stage I non-small-cell lung cancer, BRCA1+ mutation, history of skin cancer	62-66 (mean)	Selenium supplements (selenium yeast, sodium selenite or selenomethionine) / P.O. / dose: 200 -400µg/day	Placebo	Any cancer, lung cancer, prostate cancer	Parallel	3-13 years (follow-up)
Vinceti 2018 (59)	Selenium	Cancer mortality	2	18,698	359	Population with diverse health status / without: prostate cancer, haemorrhagic stroke, hypertension or with: history of skin cancer	62-63 (mean)	Selenium supplements (selenium yeast or selenomethionine) / P.O. / dose: 200µg/day	Placebo	Mortality from any cancer	Parallel	8-13 years (follow-up)
Vinceti 2018 (59)	Selenium	Colorectal cancer	3	20,259	159	Population with diverse health status / without: prostate cancer, haemorrhagic stroke, hypertension or with: history of skin cancer, completely resected stage I non-small-cell lung cancer	62-66 (mean)	Selenium supplements (selenium yeast or selenomethionine) / P.O. / dose: 200µg/day	Placebo	Colorectal cancer	Parallel	8-13 years (follow-up)

Wilson 2011 (41)	Traditional birth attendants	Perinatal mortality	5	110,068	6,207	Women giving birth in rural settings of low-income countries (Congo, Guatemala, India, Pakistan and Zambia)	25.3-26.7 (mean)/ NR (3x)	Enhanced training for traditional birth attendants (antenatal, intra-partum and postpartum care) or newborn-care training only / co-interventions: for all participants or intervention arm only (2x): resource support (e.g. clean birth kits)	Less intensive training for traditional birth attendants or no intervention (2x)	Perinatal mortality	Cluster-RCT	NR
Wilson 2011 (41)	Traditional birth attendants	Neonatal mortality	6	133,629	3,900	Women giving birth in rural settings of low-income countries (Bangladesh, Congo, Guatemala, India, Pakistan and Zambia)	25.3-26.7 (mean)/ 20-29 (range)/ NR (3x)	Enhanced training for traditional birth attendants (antenatal, intra-partum and postpartum care) or newborn-care training only / co-interventions: for all participants or intervention arm only (2x): resource support (e.g. clean birth kits)	Less intensive training for traditional birth attendants or no intervention (2x)	Neonatal mortality	Cluster-RCT	NR
Wilson 2019 (42)	Unicompartimental knee arthroplasty	Venous thromboembolism	2	614	7	Male and female population with anteromedial osteoarthritis / health status: good (medically fit, ASA-score 1/2, without prior surgery of the knee) or health status NR / BMI ≈31 or NR / functioning anterior cruciate ligament (1x)	64.7-69.7 (mean)/ 47-89 (range)	Unicompartimental knee arthroplasty	Total knee arthroplasty	Venous thromboembolism	Parallel	1-5 years follow-up
Wilson 2019 (42)	Unicompartimental knee arthroplasty	Flexion-extension range	3	270	NR	Male and female population with anteromedial osteoarthritis / diverse healths status: healthy (2x) (no major deformity, BMI<30 or without: serious liver-, heart-, renal disease, chronic pain, complex osteoarthritis) or health status NR (1x) / mean BMI 30 or NR / with (3x): intact anterior cruciate ligament	60.5-69.7 (mean)/ 47-89 (range)	Unicompartimental knee arthroplasty	Total knee arthroplasty	Range of movement	Parallel	4-5 years follow-up

Wilson 2019 (42)	Unicompartimental knee arthroplasty	Operation duration	3	660	NR	Male and female adult population with anteromedial or medial osteoarthritis or simultaneous bilateral anteriomedial osteoarthritis / health status diverse: healthy (2x) (medically fit, ASA 1/2, no major deformity, BMI<30, without serious liver-, heart-, renal disease, chronic pain, complex osteoarthritis) or health status NR / mean BMI 27.5-31 / primary arthroplasty only (2x)	59.72-65.2 (mean)	Unicompartimental knee arthroplasty	Total knee arthroplasty	Operation duration	Parallel	1-4 years follow-up
Yank 2011 (44)	Recombinant factor VII	All-cause mortality	2	191	15	Male and female population undergoing cardiac surgery / with or without post-operative bleeding / without: recent thrombotic disease, history of stroke, required urgent re-operation	62-69.5 (mean/ median)	Recombinant factor VIIa / dose: 40-90 µg/kg / administered after cardiopulmonary bypass surgery / either prophylactic or treatment of postoperative bleeding	Placebo	In-hospital mortality	Parallel	NR
Yank 2011 (44)	Recombinant factor VII	Thromboembolism	2	191	12	Male and female population undergoing cardiac surgery / with or without post-operative bleeding / without: recent thrombotic disease, history of stroke, required urgent re-operation	62-69.5 (mean/ median)	Recombinant factor VIIa / dose: 40-90µg/kg / administered after cardiopulmonary bypass surgery / either prophylactic or treatment of postoperative bleeding	Placebo	Thromboembolic events	Parallel	NR

Zhang 2016 (94)	Everolimus-eluting bioresorbable vascular scaffold	Stent thrombosis	5	3,541	36	Male (>70%) and female population with CHD / type of CHD: angina pectoris (excluding acute MI) (4x), MI (1x) / with or without: metabolic disease, hypertension, smokers and non-smokers / setting: China, Europe, Japan, USA	57.2-67.3 (mean)	Coronary stenting with Everolimus-eluting bioresorbable vascular scaffold	Coronary stenting with Everolimus-eluting metallic stents	Stent thrombosis (definite or probable) mostly defined as: definite: angiographic or pathological confirmation, probable thrombosis: typical clinical presentation without angiographic correlate and without other explanation	Parallel	6-12 months follow-up (range)
Zhang 2016 (94)	Everolimus-eluting bioresorbable vascular scaffold	All-cause mortality	5	3,522	30	Male (>70%) and female population with CHD / type of CHD: angina pectoris (excluding acute MI) (4x), mixed population with angina pectoris or MI (1x) / with or without: metabolic disease, hypertension, smokers and non-smokers / setting: China, Europe, Japan, USA	57.2-67.3 (mean)	Coronary stenting with Everolimus-eluting bioresorbable vascular scaffold	Coronary stenting with Everolimus-eluting metallic stents	All-cause death	Parallel	9-12 months follow-up (range)
Zhang 2016 (94)	Everolimus-eluting bioresorbable vascular scaffold	Coronary heart disease mortality	3	2,623	13	Male (>70%) and female population with CHD / type of CHD: angina pectoris (excluding acute MI) (2x), mixed population with angina or MI (1x) / with or without: metabolic disease, hypertension, smokers and non-smokers / setting: China, Europe, USA	57.2-65 (mean)	Coronary stenting with Everolimus-eluting bioresorbable vascular scaffold	Coronary stenting with Everolimus-eluting metallic stents	Cardiac death	Parallel	9-12 months follow-up (range)

Zhang 2017 (95)	Percutaneous coronary intervention	All-cause mortality	5	4,499	353	Male (73-77.5%) and female population with CHD / without: acute MI (4x) / with or without: metabolic disease and hypertension, prior MI (6.7-33%), current smokers: 20.8-27.7%	61.3-66.2 (mean)	Percutaneous coronary intervention / stent types: bare metal stent or early-generation drug eluting stent (3x); second-generation drug eluting stent (2x)	Coronary artery bypass graft surgery	All-cause mortality	Parallel	3-10 years (range)
Zhang 2017 (95)	Percutaneous coronary intervention	Cardiovascular mortality	4	4,394	185	Male (76-77.5%) and female population with CHD / without: acute MI (3x) / with or without: metabolic disease and hypertension, prior MI (6.7-25.4%); current smokers: 20.8-27.7%	62.7-66.2 (mean)	Percutaneous coronary intervention / stent types: bare metal stent or early-generation drug eluting stent (2x); second-generation drug eluting stent (2x)	Coronary artery bypass graft surgery	Cardiovascular mortality	Parallel	3-5 years (range)
Zhang 2017 (95)	Percutaneous coronary intervention	Myocardial infarction	5	4,499	254	Male (73-77.5%) and female population with CHD / without: acute MI (4x) / with or without: metabolic disease and hypertension, prior MI (6.7-33%), current smokers: 20.8-27.7%	61.3-66.2 (mean)	Percutaneous coronary intervention / stent types: bare metal stent or early-generation drug eluting stent (3x); second-generation drug eluting stent (2x)	Coronary artery bypass graft surgery	Myocardial infarction	Parallel	3-10 years (range)
Ziff 2015 (96)	Digoxin	All-cause mortality	7	8,406	NR	Population with chronic heart failure of different degrees (NYHA II-IV) / including different etiologies for heart failure / with: reduced or preserved left ventricular ejection fraction / mostly without: atrial fibrillation	57-67 (mean)	Digoxin	Placebo	All-cause mortality	Parallel	3-37 monts follow-up
Ziff 2015 (96)	Digoxin	Cardiovascular mortality	5	8,068	NR	Population with chronic heart failure of different degrees (NYHA II-IV) / including different etiologies for heart failure / with: reduced or preserved left ventricular ejection fraction / mostly without: atrial fibrillation	60-67 (mean)	Digoxin	Placebo	Cardiovascular mortality	Parallel	3-37 months (follow-up)

Ziff 2015 (96)	Digoxin	Hospital admission	2	7,778	NR	Population with different degrees of chronic heart failure (NYHA II-IV) / with reduced or preserved left ventricular ejection fraction / all in sinus rythm	63-67 (mean)	Digoxin	Placebo	All-cause hospital admission	Parallel	37 months (follow-up)

ACS= acute coronary syndrome; AIDS= acquired immune deficiency syndrome; ART= antiretroviral therapy; ASA= american society of anesthesiologists; BCG= bacillus calmette-guérin; BMI= body mass index; CABG= coronary artery bypass graft; CAD= coronary artery disease; CD4= cluster of differentiation 4; CHD= coronary heart disease; COPD= chronic obstructive pulmonary disease; CVD= cardiovascular disease; DDP-4= dipeptidyl peptidase 4; DTP= diphteria, tetanus, pertussis; eGFR= estimated glomerular filtration rate; GDM= gestational diabetes mellitus; HbA1c= hemoglobin A1c; HIV= human immunodeficiency virus; HSV-2= herpes simplex virus type 2; ICU= intensive care unit; MI= myocardial infarction; MRI= magnetic resonance imaging; MRSA= methicillin resistant staphylococcus aureus; MSSA= methicillin sensitive staphylococcus aureus; NA= not applicable; NOS= newcastle-ottawa scale; NR= not reported; NSAID= non-steroidal anti-inflammatory drug; NSTE-ACS= non-ST elevation acute coronary syndrome; NYHA= new york heart association (stage); PCI= percutaneous coronary intervention; PPV= pneumococcal polysaccharide vaccine; SGLT-2= sodium glucose transporter 2; SPMSQ= short portable mental status questionnaire; STAT= instantly; STEMI= ST elevation myocardial infarction; µL= microliter WHO= world health organization // units: g= gram; IU= international units; kcal= kilocalorie; kg= kilogram; L= liter; mg= milligram; mL= milliliter; mmol= millimol; µg= microgram; µL= microliter // application routes: IM= intramuscular; IV= intravenous; P.O.= per os; SC= subcutaneous.

Table S5. Certainty of the evidence and risk of bias for included bodies of evidence from randomized controlled trials

Reference/ year	Intervention	Outcome	Certainty/ strength of the evidence	Tool	risk of bias tool	Reported as number of low risk of bias studies per domain
Abou-Setta 2011 (74)	Nerve block	Delirium	Moderate (RCTs and cohort studies)	AHRQ/ GRADE	Cochrane	Random sequence generation (3/4), allocation concealment (3/4), Blinding (2/4), incomplete outcome data (2/4) selective reporting (4/4); other bias (2/4)
Abou-Setta 2011 (74)	Spinal anesthesia	All-cause mortality	Low (RCTs and cohort studies)	AHRQ/ GRADE	Cochrane	Random sequence generation (0/2), allocation concealment (0/2), blinding (1/2), incomplete outcome data (1/2) selective reporting (2/2); other bias (0/2)
Aburto 2013 (75)	Low sodium	All-cause mortality	Moderate	GRADE	Cochrane	Random sequence generation (1/4), allocation concealment (3/4), blinding (3/4), incomplete outcome data (3/4), selective reporting (4/4), assessment of compliance? (4/4), groups balanced at baseline? (4/4), intention to treat analysis? (4/4), free from follow up bias? (1/4)
Aburto 2013 (75)	Low sodium	Cardiovascular disease	Moderate	GRADE	Cochrane	Random sequence generation (1/2), allocation concealment (1/2), blinding (0/2), incomplete outcome data (1/2), selective reporting (1/2), assessment of compliance? (2/2), groups balanced at baseline? (2/2), intention to treat analysis? (2/2), free from follow up bias? (0/2)
Ahmad 2015 (27)	Intra-aortic balloon pump	All-cause mortality	NR	NA	Cochrane	Random sequence generation (descriptively rated), allocation concealment (0/12), blinding of participants and personnel (0/12), blinding of outcome assessments (descriptively rated), incomplete outcome data (descriptively rated), selective reporting (descriptively rated)
Alexander 2017 (76)	DHA and EPA	Coronary heart disease	NR	NA	Cochrane	Random sequence generation (10/18), allocation concealment (12/18), blinding of personnel and participants (14/18), blinding of outcome assessment (16/18), incomplete outcome data (17/18), selective reporting (18/18), other bias (15/18)
Alexander 2017 (76)	DHA and EPA	Coronary heart disease mortality	NR	NA	Cochrane	Random sequence generation (9/14), allocation concealment (9/14), blinding of personnel and participants (10/14), blinding of outcome assessment (13/14), incomplete outcome data (14/14) selective reporting (14/14), other bias (12/14)
Alexander 2017 (76)	DHA and EPA	Coronary heart disease incidence	NR	NA	Cochrane	Random sequence generation (7/9), allocation concealment (6/9), blinding of personnel and participants (6/9), blinding of outcome assessment (8/9), incomplete outcome data (8/9), selective reporting (9/9), other bias (8/9)

Alipanah 2018 (24)	Self-administered therapy	Low treatment success	NR	NA	Cochrane	Random sequence generation (4/4), allocation concealment (4/4), blinding of personnel and participants (0/4), blinding of outcome (0/4), incomplete outcome data (1/4), selective reporting (4/4)
Alipanah 2018 (24)	Self-administered therapy	Low treatment completion	NR	NA	Cochrane	Random sequence generation (4/5), allocation concealment (3/5), blinding of personnel and participants (0/5), blinding of outcome (0/5), incomplete outcome data (2/5), selective reporting (4/5)
Alipanah 2018 (24)	Self-administered therapy	All-cause mortality	NR	NA	Cochrane	Random sequence generation (4/4), allocation concealment (3/4), blinding of personnel and participants (0/4), blinding of outcome (0/4), incomplete outcome data (1/4), selective reporting (4/4)
Anglemyer 2013 (77)	Antiretroviral therapy	HIV infection	High (virologically linked-HIV infection)	GRADE	Cochrane	Random sequence generation (1/1), allocation concealment (0/1), blinding (performance bias and detection bias) (0/1), incomplete outcome data (1/1), selective reporting (1/1), other bias (1/1)
Azad 2017 (21)	Nonnutritive sweeteners	Body Mass Index	NR	NA	Cochrane	Random sequence generation (3/3), allocation concealment (3/3), blinding (2/3), incomplete outcome data (2/3), selective reporting (2/3), other bias (3/3)/ overall: low risk of bias (1/3)
Barnard 2015 (28)	Surgical abortion by mid-level providers	Failure or incomplete abortion	Low	GRADE	Cochrane	Random sequence generation (2/2), allocation concealment (2/2), blinding of participants and personnel (2/2), blinding of outcome assessment (0/2), incomplete outcome data, all outcomes (2/2), selective reporting (0/2), other bias (0/2)
Barnard 2015 (28)	Surgical abortion by mid-level providers	Complications	Low	GRADE	Cochrane	Random sequence generation (2/2), allocation concealment (2/2), blinding of participants and personnel (2/2), blinding of outcome assessment (0/2), incomplete outcome data, all outcomes (2/2), selective reporting (0/2), other bias (0/2)
Barnard 2015 (28)	Surgical abortion by mid-level providers	Abortion failure and complications	Low	GRADE	Cochrane	Random sequence generation (2/2), allocation concealment (2/2), blinding of participants and personnel (2/2), blinding of outcome assessment (0/2), incomplete outcome data, all outcomes (2/2), selective reporting (0/2), other bias (0/2)
Bellemain-Appaix 2012 (48)	Clopidogrel	All-cause mortality	NR	NA	JADAD	Randomization (6/6), double-blinding (4/6), withdrawals/ dropouts description (6/6); one RCT (Davlouros 2009) rated with NOS without a reason given

Bellemain-Appaix 2012 (48)	Clopidogrel	Major bleeding	NR	NA	JADAD	Randomization (6/6), double-blinding (4/6), withdrawals/ dropouts description (6/6); one RCT (Davlouros 2009) rated with NOS without a reason given
Bellemain-Appaix 2012 (48)	Clopidogrel	Coronary heart disease	NR	NA	JADAD	Randomization (6/6), double-blinding (4/6), withdrawals/ dropouts description (6/6); one RCT (Davlouros 2009) rated with NOS without a reason given
Bellemain-Appaix 2014 (47)	P2Y12 inhibitors	All-cause mortality	NR	NA	Cochrane	Random sequence generation (3/3), allocation concealment (3/3), blinding of participants and personnel (3/3), blinding of outcome assessment (3/3), incomplete outcome data(3/3), selective reporting (death, MACE, bleeding outcomes) (3/3), selective reporting (stent thrombosis) (1/3)
Bellemain-Appaix 2014 (47)	P2Y12 inhibitors	Major bleeding	NR	NA	Cochrane	Random sequence generation (3/3), allocation concealment (3/3), blinding of participants and personnel(3/3), blinding of outcome assessment (3/3), incomplete outcome data (3/3), selective reporting (death, MACE, bleeding outcomes) (3/3), selective reporting (stent thrombosis) (1/3)
Bellemain-Appaix 2014 (47)	P2Y12 inhibitors	Main composite ischemic endpoint	NR	NA	Cochrane	Random sequence generation (3/3), allocation concealment (3/3), blinding of participants and personnel (3/3), blinding of outcome assessment (3/3), incomplete outcome data (3/3), selective reporting (death, MACE, bleeding outcomes) (3/3), selective reporting (stent thrombosis) (1/3)
Bloomfield 2016 (22)	Mediterranean diet	Breast cancer	Low (RCTs and cohort studies)	AHRQ 2010	Cochrane	Random sequence generation (1/1), allocation concealment (1/1), blinding (1/1), incomplete outcome data (1/1), selective outcome reporting (1/1)/ overall: low risk of bias (1/1)
Bolland 2015 (49)	Calcium	All fractures	NR	NA	Cochrane	Yes/ no answers, number in brackets is number of trials with "yes"; random sequence generation described (9/22), allocation concealment (9/22), number of double-blind trials (14/22), blinding of outcome assessment (17/22), incomplete outcome data (3/22), differential drop-out (yes or not specified) (3/22), other bias (7/22)/ overall: low risk of bias (4/22)
Bolland 2015 (49)	Calcium	Vertebral fracture	NR	NA	Cochrane	Yes/ no answers, number in brackets is number of trials with "yes"; random sequence generation described (4/12), allocation concealment (3/12), number of double-blind trials (9/12), blinding of outcome assessment (11/12), incomplete outcome data (4/12), differential drop-out (yes or not specified) (1/12), other bias (2/12)/ overall: low risk of bias (4/12)
Bolland 2015 (49)	Calcium	Hip fracture	NR	NA	Cochrane	Yes/ no answers, number in brackets is number of trials with "yes"; random sequence generation described (7/13), allocation concealment (6/13), number of double-blind trials (8/13), blinding of outcome assessment (9/13), incomplete outcome data (2/13), differential drop-out (yes or not specified) (2/13), other bias (5/13)/ overall: low risk of bias (3/12)

Brenner 2014 (29)	Sigmoidoscopy	Colorectal cancer mortality	NR	NA	Individual tool	8.5/9 (Mean number of quality criteria met)
Brenner 2014 (29)	Sigmoidoscopy	Colorectal cancer incidence	NR	NA	Individual tool	8.5/9 (Mean number of quality criteria met)
Chowdhury 2012 (78)	Omega-3	Cerebrovascular disease	NR	NA	Individual tool based onMOOSE, QUATSO, and STROBE guidelines	Modified quality score (for RCTs and cohort studies) (0-6, 6 is best): average 5.5
Chowdhury 2014a (79)	α-linolenic acid	Coronary heart disease	NR	NA	Cochrane	Random sequence generation (4/4), allocation concealment (4/4), blinding of participants and personnel (3/4), blinding of outcome assessments (3/4), incomplete outcome data (4/4), selective reporting (3/4), other bias (3/4)
Chowdhury 2014a (79)	Omega-3	Coronary heart disease	NR	NA	Cochrane	Random sequence generation (17/17), allocation concealment (16/17), blinding of participants and personnel (14/17), blinding of outcome assessments (14/17), incomplete outcome data (17/17), selective reporting (17/17), other bias (13/17)
Chowdhury 2014a (79)	Omega-6	Coronary heart disease	NR	NA	Cochrane	Random sequence generation (8/8), allocation concealment (8/8), blinding of participants and personnel (4/8), blinding of outcome assessments (5/8), incomplete outcome data (8/8), selective reporting (6/8), other bias (4/8)
Chowdhury 2014b (80)	Vitamin D	All-cause mortality	NR	NA	Cochrane	Random sequence generation (22/22), allocation concealment (20/22), blinding of personnel and participants (18/22), blinding of outcome assessment (8/22), incomplete outcome data (12/22), selective reporting (22/22), other bias (10/22)
Chung 2011 (58)	Vitamin D	Colorectal cancer	NR	NA	AHRQ/ CONSORT	Fair quality (1/1)
Chung 2011 (58)	Vitamin D	Breast cancer	NR	NA	AHRQ/ CONSORT	Fair quality (1/1)

Chung 2016 (56)	Calcium	Cardiovascular mortality	NR	NA	AHRQ/ CONSORT	Appropriate randomization technique (2/2), allocation concealment (2/2), dropout rate <20% (2/2), blinded outcome assessment (1/2), intention to-treat analysis (2/2), appropriate statistical analysis (2/2), assessment for confounding (2/2), clear reporting with no discrepancies (2/2)
Ding 2017 (81)	Dairy	Systolic blood pressure	NR	NA	NA	NR
Fenton 2018 (30)	Radiation therapy	Erectile dysfunction	Moderate (RCTs and cohort studies)	AHRQ/ GRADE	AHRQ/ USPSTF	Good quality (1/1)
Fenton 2018 (30)	Radical Prostatectomy	Urinary incontinence	Moderate (RCTs and cohort studies)	AHRQ/ GRADE	AHRQ/ USPSTF	Good quality (3/3)
Fenton 2018 (30)	Radical Prostatectomy	Erectile dysfunction	Moderate (RCTs and cohort studies)	AHRQ/ GRADE	AHRQ/ USPSTF	Good quality (3/3)
Filippini 2017 (43)	Disease-modifying drugs	Conversion to clinically definite multiple sclerosis	NR	NA	Cochrane	Random sequence generation (7/7), allocation concealment (4/7), other major baseline imbalance (7/7), blinding of personnel and participants (0/7), blinding of outcome assessment (3/7), incomplete outcome data (3/7), selective reporting (6/7)
Fluri 2010 (31)	Extracranial-intracranial arterial bypass	All-cause mortality	NR	NA	Individual tool	Adequate allocation concealment (2/2)
Fluri 2010 (31)	Extracranial-intracranial arterial bypass	Stroke	NR	NA	Individual tool	Adequate allocation concealment (2/2)
Fluri 2010 (31)	Extracranial-intracranial arterial bypass	Stroke mortality or dependency	NR	NA	Individual tool	Adequate allocation concealment (1/1)

Gargiulo 2016 (32)	Transcatheter aortic valve	Early all-cause mortality	NR	NA	Cochrane	Random sequence generation (5/5), allocation concealment (5/5), blinding of personnel and participants (0/5), blinding of outcome assessment (5/5), incomplete outcome data (5/5), selective reporting (5/5), other bias (4/5)
Gargiulo 2016 (32)	Transcatheter aortic valve	Mid-term all-cause mortality	NR	NA	Cochrane	Random sequence generation (5/5), allocation concealment (5/5), blinding of personnel and participants (0/5), blinding of outcome assessment (5/5), incomplete outcome data (5/5), selective reporting (5/5), other bias (4/5)
Gargiulo 2016 (32)	Transcatheter aortic valve	Long-term all-cause mortality	NR	NA	Cochrane	Random sequence generation (4/4), allocation concealment (4/4), blinding of personnel and participants (0/4), blinding of outcome assessment (4/4), incomplete outcome data (4/4), selective reporting (4/4), other bias (4/4)
Hartling 2013 (50)	Treating gestational diabetes mellitus	High birth weight	Moderate	AHRQ/ GRADE	Cochrane	Random sequence generation (2/5), allocation concealment (1/5), blinding of personnel and participants (2/5), blinding of outcome assessment (4/5), incomplete outcome data (3/5), selective reporting (5/5), other bias (5/5)/ overall: good (1/5), fair (3/5), poor (1/5)
Hartling 2013 (50)	Treating gestational diabetes mellitus	Large-for-gestational age neonate	NR	NA	Cochrane	Random sequence generation (2/3), allocation concealment (1/3), blinding of personnel and participants (2/3), blinding of outcome assessment (3/3), incomplete outcome data (3/3), selective reporting (3/3), other bias (3/3)/ overall: good (1/3), fair (2/3)
Hartling 2013 (50)	Treating gestational diabetes mellitus	Shoulder dystocia	Moderate	AHRQ/ GRADE	Cochrane	Random sequence generation (1/3), allocation concealment (1/3), blinding of personnel and participants (2/3), blinding of outcome assessment (3/3), incomplete outcome data (2/3), selective reporting (3/3), other bias (3/3)/ overall: good (1/3), fair (2/3)
Henderson 2019 (51)	Treating asymptomatic bacteriuria	Pyelonephritis	Moderate	AHRQ/ GRADE	USPSTF criteria	Fair quality (12/12)
Higgins 2016 (25)	Bacillus Calmette-Guérin	All-cause mortality	NR	NA	Cochrane	Risk of bias due to confounding (2/3), risk of performance bias (2/3), detection bias (3/3), attrition bias(3/3), bias in selection (0/3)/ overall risk: moderate (1/3), low (2/3)
Higgins 2016 (25)	Measles containing vaccines	All-cause mortality	NR	NA	Cochrane	Confounding (1/4), performance bias (1/4), detection bias (3/4), attrition bias (0/4), selection bias (0/4)/ overall: moderate risk (3/4)

Hopley 2010 (33)	Total hip arthroplasty	Reoperation	NR	NA	Special criteria for study topic according to Parker et al.	Average number of quality criteria from rating scale met: 7.3
Hopley 2010 (33)	Total hip arthroplasty	Dislocation	NR	NA	Special criteria for study topic according to Parker et al.	Average number of quality criteria from rating scale met: 7.3
Hopley 2010 (33)	Total hip arthroplasty	Deep infection	NR	NA	Special criteria for study topic according to Parker et al.	Average number of quality criteria from rating scale met: 7.3
Hüpfl 2010 (67)	Chest-compression-only cardiopulmonary resuscitation	All-cause mortality	NR	NA	NR	High quality (3/3)
Jamal 2013 (82)	Non-calcium-based phosphat binders	All-cause mortality	NR	NA	Cochrane	Number of studies with low risk of bias in all domains (4/8), high risk of bias (2/8), unclear risk of bias (2/8)/ results of domains not reported
Jefferson 2010 (46)	Parenteral influenza vaccine	Influenza-like illness	NR	NA	Cochrane (incomplete)	Allocation concealment adequate (2/4)
Jefferson 2010 (46)	Parenteral influenza vaccine	Influenza	NR	NA	Cochrane (incomplete)	Allocation concealment adequate (1/3)
Jefferson 2012 (34)	Inactivated influenza vaccines	Influenza	NR	NA	Cochrane	Random sequence generation (2/5), allocation concealment (2/5), blinding of participants and personnel (3/5), detection bias (3/5), incomplete outcome data (3/5)/ summary (overall assessment) (2/5)
Jefferson 2012 (34)	Inactivated influenza vaccines	Influenza-like illness	NR	NA	Cochrane	Random sequence generation (2/5), allocation concealment (1/5), blinding (performance bias and detection bias), all outcomes (3/5), incomplete outcome data, all outcomes (3/5)/ summary (overall assessment) (1/5)

Jin 2012 (83)	Total flavonoids	Colorectal neoplasms	NR	NA	Cochrane	Random sequence generation (0/1), allocation concealment (0/1), blinding of personnel and participants (0/1), blinding of outcome assessment (0/1), incomplete outcome data (0/1), selective reporting (0/1), other bias (1/1)
Johnston 2019 (23)	Low red meat	All-cause mortality	Low	GRADE	Cochrane	Randomization sequence adequately generated (1/1), allocation adequately concealed (1/1), blinding of participants (0/1), blinding of data collectors (1/1), blinding of outcome assessors or adjudicators (1/1), loss to follow-up (missing outcome data) (1/1), reporting bias (1/1), other bias (1/1)
Johnston 2019 (23)	Low red meat	Cardiovascular mortality	Very low	GRADE	Cochrane	Randomization sequence adequately generated (1/1), allocation adequately concealed (1/1), blinding of participants (0/1), blinding of data collectors (1/1), blinding of outcome assessors or adjudicators (1/1), loss to follow-up (missing outcome data) (0/1), reporting bias (1/1), other bias (1/1)
Johnston 2019 (23)	Low red meat	Cardiovascular disease	Low	GRADE	Cochrane	Randomization sequence adequately generated (1/1), allocation adequately concealed (1/1), blinding of participants (0/1), blinding of data collectors (0/1), blinding of outcome assessors or adjudicators (1/1), loss to follow-up (missing outcome data) (1/1), reporting bias (1/1), other bias (1/1)
Kansagara 2013 (52)	Transfusion	All-cause mortality	Low	GRADE	Cochrane	Random sequence generation (3/6), allocation concealment (4/6), blinding of personnel and participants and outcome assessors (not convertable from SR), incomplete outcome data (6/6), selective reporting (6/6), other bias (not convertable from SR)/ overall low risk of bias: (6/6)
Keag 2018 (84)	Caesarean section	Urinary incontinence	NR	NA	SIGN	Study quality: ++ (0,+ or ++)
Keag 2018 (84)	Caesarean section	Fecal incontinence	NR	NA	SIGN	Study quality: ++ (0,+ or ++)
Kredo 2014 (85)	Starting and maintaining antiretroviral therapy	All-cause mortality	High	GRADE	Cochrane, modified	Baseline CD4 count (1/1), other baseline variables (0/1), co-interventions (1/1), random sequence generation (1/1), allocation concealment (1/1), contamination protection (0/1)
Kredo 2014 (85)	Starting and maintaining antiretroviral therapy	Attrition	Moderate	GRADE	Cochrane, modified	Baseline CD4 count (1/1), other baseline variables (0/1), co-interventions (1/1), random sequence generation (1/1), allocation concealment (1/1), contamination protection (0/1)

Kredo 2014 (85)	Maintaining antiretroviral therapy	All-cause mortality	Moderate	GRADE	Cochrane, modified	Baseline CD4 count (2/2), other baseline variables (1/2), co-interventions (2/2), random sequence generation (2/2), allocation concealment (2/2), contamination protection (1/2)
Li 2014 (54)	Exenatide	Acute pancreatitis	NR	NA	Cochrane, modified	Random sequence generation (4/5), allocation concealment (4/5), blinding of participants and personnel (1/5), blinded assessment of pancreatitis events (1/5), adjudication of pancreatitis events (0/5)
Li 2016 (53)	DDP-4 inhibitors	Heart failure	Low	GRADE	Cochrane, modified	Randomization sequence generation (16/34), allocation concealment (11/34), blinding of participants and personnel (34/34), blinded assessment HF or HF-hospital admission events (34/34), HF or HF-hospital admission outcome adjudicated (6/34), blinded HF or HF-hospital admission outcome adjudication (3/34), industry funded (34/34)
Li 2016 (53)	DDP-4 inhibitors	Hospital admission for heart failure	Moderate	GRADE	Cochrane, modified	Randomization sequence generation (2/5), allocation concealment (2/5), blinding of participants and personnel (4/5), blinded assessment HF or HF-hospital admission events (4/5), HF or HF-hospital admission outcome adjudicated (yes: (5/5)), blinded HF or HF-hospital admission outcome adjudication (yes: (3/5)), industry funded (yes: (5/5)
Matthews 2018 (86)	Tamoxifen	Heart failure	NR	NA	Cochrane	Random sequence generation (1/1), allocation concealment (1/1), blinding (0/1), incomplete outcome data (0/1), selective reporting (0/1), other sources of bias (1/1)
Menne 2019 (87)	SGLT-2 inhibitors	Acute kidney injury	NR	NA	Cochrane	Random sequence generation (32/41), allocation concealment (32/41), blinding of participants and personnel (36/41), detection bias (blinding of outcome assessment) (29/41), incomplete outcome data serious adverse events (34/41), incomplete outcome data non-serious adverse events (14/41), selective reporting (37/41)
Mesgarpour 2017 (88)	Erythropoiesis stimulating agents	Venous thromboembolism	Very low (RCTs and cohort studies)	GRADE	Cochrane	Random sequence generation (9/12), allocation concealment (6/12), blinding of personnel and participants (10/12), blinding of outcome assessment (6/12), incomplete outcome data (7/12), selective reporting (10/12), other bias (3/12)
Mesgarpour 2017 (88)	Erythropoiesis stimulating agents	All-cause mortality	Low (RCTs and cohort studies)	GRADE	Cochrane	Random sequence generation (10/17), allocation concealment (6/17), blinding of personnel and participants (12/17), blinding of outcome assessment (9/17), incomplete outcome data (12/17), selective reporting (10/17), other bias (2/17)
Moberley 2013 (89)	Pneumococcal polysaccharide vaccines	Invasive pneumococcal disease	NR	NA	Cochrane	Random sequence generation (4/10), allocation concealment (5/10), blinding of participants and personnel (6/10), detection bias (blinding of outcome assessment) (6/10), incomplete outcome data (1/10), selective reporting (1/10)

Molnar 2015 (35)	Neoral (Cyclosporin)	Acute rejection of kidney transplant	NR	NA	Cochrane	Random sequence generation (1/2), allocation concealment (1/2), blinding of participants and personnel (1/2), blinding of outcome assessment (0/2), incomplete outcome data (clinical outcomes) (2/2), selective reporting (2/2), other bias (0/2)
Navarese 2013 (90)	Early intervention for NSTE-ACS	All-cause mortality	NR	NA	Cochrane	Number of trials with answer "Yes": adequate sequence generation? (5/7), allocation concealment? (5/7), patient blinding? (0/7), physician blinding? (0/7), adjudication of outcomes blinding? (6/7), incomplete data outcome addressed? (7/7), selective outcome reporting? (0/7), free of other Bias? (7/7)
Navarese 2013 (90)	Early intervention for NSTE-ACS	Myocardial infarction	NR	NA	Cochrane	Number of trials with answer "Yes": adequate sequence generation? (5/7), allocation concealment? (5/7), patient blinding? (0/7), physician blinding? (0/7), adjudication of outcomes blinding? (6/7), incomplete data outcome addressed? (7/7), selective outcome reporting? (0/7), free of other bias? (7/7)
Navarese 2013 (90)	Early intervention for NSTE-ACS	Major bleeding	NR	NA	Cochrane	Number of trials with answer "Yes": adequate sequence generation? (5/7), allocation concealment? (5/7), patient blinding? (0/7), physician blinding? (0/7), adjudication of outcomes blinding? (6/7), incomplete data outcome addressed? (7/7), selective outcome reporting? (0/7), free of other bias? (7/7)
Nelson 2010 (36)	Caesarean section	Anal incontinence, feces	NR	NA	NA	NR
Nelson 2010 (36)	Caesarean section	Anal incontinence, flatus	NR	NA	NA	NR
Nieuwenhuijse 2014 (37)	Ceramic-on-ceramic bearings for total hip arthroplasty	Harris Hip Score	NR	NA	Modified rating tool based on Cochrane and CONSORT criteria; partly with verbal ratings, overall: low to high quality	High quality: (1/7)
Nieuwenhuijse 2014 (37)	High-flexion total knee arthroplasty	Flexion	NR	NA	Modified rating tool based on Cochrane and CONSORT criteria; partly with verbal ratings, overall: low to high quality	High quality: (7/20)
Nieuwenhuijse 2014 (37)	Gender-specific total knee arthroplasty	Flexion-extension range	NR	NA	Modified rating tool based on Cochrane and CONSORT criteria; partly with	High quality: (1/6)

					verbal ratings, overall: low to high quality	
Nikooie 2019 (55)	Second generation antipsychotics	Sedation	Moderate	AHRQ	Cochrane	Random sequence generation (5/6), allocation concealment (2/6), blinding of personnel, participants and blinding of outcome assessment (3/6), incomplete outcome data (3/6), selective reporting (3/6), other bias (1/6)/ overall: low risk of bias (3/6)
Nikooie 2019 (55)	Second generation antipsychotics	Neurologic outcomes	NR	NA	Cochrane	Random sequence generation (5/6), allocation concealment (3/6), blinding of personnel, participants and blinding of outcome assessment (4/6), incomplete outcome data (4/6), selective reporting (4/6), other bias (2/6)/ overall: low risk of bias (4/6)
Ochen 2019 (91)	Surgery for achilles tendon rupture	Re-rupture	NR	NA	MINORS	MINORS score: 20.3 (mean)/ 16-23 (range)
Ochen 2019 (91)	Surgery for achilles tendon rupture	Complications	NR	NA	MINORS	MINORS score: 20.4 (mean)/ 16-23 (range)
Pittas 2010 (60)	Vitamin D	Hypertension	NR	NA	AHRQ/ CONSORT	Good quality (1/1)
Raman 2013 (38)	Carotid endarterectomy	Ipsilateral stroke	Moderate	AHRQ/ GRADE	AHRQ	Appropriate randomization (3/3), allocation concealment (3/3), dropout rate <20% (3/3), blinded patient (0/3), blinded outcome assessment (2/3), intention to treat analysis (3/3), appropriate statistical analysis (3/3), if multicenter, was this accounted for in analysis? (0/3)/ overall: low risk of bias (3/3), quality A (3/3)
Raman 2013 (38)	Carotid endarterectomy	Stroke	Moderate	AHRQ/ GRADE	AHRQ	Appropriate randomization (3/3), allocation concealment (3/3), dropout rate <20% (3/3), blinded patient (0/3), blinded outcome assessment (2/3), intention to treat analysis (3/3), appropriate statistical analysis (3/3), if multicenter, was this accounted for in analysis? (0/3)/ overall: low risk of bias (3/3), quality A (3/3)
Raman 2013 (38)	Carotid artery stenting	Periprocedural stroke	Insufficient	AHRQ/ GRADE	AHRQ	Appropriate randomization (2/2), allocation concealment (1/2), dropout rate <20% (2/2), blinded patient (0/2), blinded outcome assessment (2/2), intention to treat analysis (2/2), appropriate statistical analysis (2/2), if multicenter, was this accounted for in analysis? (0/2)/ overall: low risk of bias (1/2), good quality (1/2), fair quality (1/2)

Schweizer 2013 (39)	Nasal deconolization	Surgical site infection	NR	NA	Cochrane	Random sequence generation (5/5), allocation concealment (4/5), blinding of personnel and participants (5/5), blinding of outcome assessment (5/5), incomplete outcome data (5/5), selective reporting (4/5), other bias (5/5)
Schweizer 2013 (39)	Glycopeptide prophylaxis	Surgical site infection	NR	NA	Cochrane	Random sequence generation (4/8), allocation concealment (2/8), blinding of personnel and participants (4/8), blinding of outcome assessment (5/8), incomplete outcome data (7/8), selective reporting (8/8), other bias (7/8)
Silvain 2012 (40)	Enoxaparin	All-cause mortality	NR	NA	Cochrane/ NOS	Low risk of bias (2/3); retrospective randomized analyses: NOS (9/9); rating for domains not presented
Silvain 2012 (40)	Enoxaparin	Major bleeding	NR	NA	Cochrane/ NOS	Low risk of bias (5/6); retrospective randomized analyses: NOS (9/9); rating for domains not presented
Silvain 2012 (40)	Enoxaparin	All-cause mortality or myocardial infarction	NR	NA	Cochrane/ NOS	Low risk of bias (7/11); retrospective randomized analyses: NOS (9/9); rating for domains not presented
Suthar 2012 (26)	Antiretroviral therapy	Tuberculosis infection	NR	NA	Cochrane/ modified NOS	Random sequence generation (2/2), allocation concealment (2/2), blinding of personnel and participants and blinding of outcome assessment (0/2), incomplete outcome data (2/2), selective reporting (2/2), other bias (2/2)/ RCTs were also assessed by modified NOS (93% of maximum score)
Te Morenga 2013 (61)	Sugar	Weight gain	Moderate	GRADE	Cochrane	Random sequence generation (3/10), allocation concealment (1/10), blinding of personnel and participants (5/10), blinding of outcome assessment (1/10), incomplete outcome data (4/10), selective reporting (5/10), other bias (9/10), sugar industry funding (4/10)
Te Morenga 2013 (61)	Sugar	Body Mass Index	Moderate (BMI and BMI-z score)	GRADE	Cochrane	Random sequence generation (3/3), allocation concealment (1/3), blinding of personnel and participants (0/3), blinding of outcome assessment (1/3), incomplete outcome data (3/3), selective reporting (3/3), other bias (3/3), sugar industry funding (2/3)
Thomas 2010 (92)	Influenza vaccines	Influenza-like illness	NR	NA	Cochrane	Random sequence generation (2/3), allocation concealment (1/3), blinding (0/3), incomplete outcome data (1/3), selective reporting (3/3), other bias (1/3)

Tickell-Painter 2017 (93)	Mefloquine	Discontinuation due to adverse effects	High (RCTs and cohort studies)	GRADE	Cochrane	Random sequence generation (3/3), allocation concealment (2/3), blinding of participants and personnel (2/3), blinding of outcome assessment (2/3), incomplete outcome data (0/3), selective reporting (1/3), other bias (1/3)
Tickell-Painter 2017 (93)	Mefloquine	Serious adverse events or effects	NR	NA	Cochrane	Random sequence generation (0/3), allocation concealment (0/3), blinding of participants and personnel (3/3), blinding of outcome assessment (1/3), incomplete outcome data (2/3), selective reporting (0/3), other (1/3)
Tickell-Painter 2017 (93)	Mefloquine	Nausea	NR	NA	Cochrane	Random sequence generation (0/2), allocation concealment (0/2), blinding of participants and personnel (2/2), blinding of outcome assessment (1/2), incomplete outcome data (2/2), selective reporting (0/2), other (1/2)
Tricco 2018 (45)	Live-attenuated zoster vaccines	Suspected Herpes Zoster	NR	NR	Cochrane	Random sequence generation (2/5), allocation concealment (1/5), blinding of personnel and participants (5/5), blinding of outcome assessment (4/5), incomplete outcome data (5/5), selective reporting (4/5), other bias (0/5)
Vinceti 2018 (59)	Selenium	Cancer	High (only for 3/5 studies with low risk of bias)	GRADE	Cochrane	Random sequence generation (4/5), allocation concealment (4/5), blinding (3/5), selective reporting (5/5)
Vinceti 2018 (59)	Selenium	Cancer mortality	High (only for 1/2 studies with low risk of bias)	GRADE	Cochrane	Random sequence generation (2/2), allocation concealment (2/2), blinding (1/2), selective reporting (2/2)
Vinceti 2018 (59)	Selenium	Colorectal cancer	High (only for 2/3 studies with low risk of bias)	GRADE	Cochrane	Random sequence generation (3/3), allocation concealment (3/3), blinding (2/3), selective reporting (3/3)
Wilson 2011 (41)	Traditional birth attendants	Perinatal mortality	NR	NA	CONSORT	Adequate randomisation (5/5), baseline comparability (5/5), sample size calculation (5/5), accounted for clustering (4/5), masking (0/5), loss of clusters to follow-up (5/5), intention to treat analysis (5/5)
Wilson 2011 (41)	Traditional birth attendants	Neonatal mortality	NR	NA	CONSORT	Adequate randomisation (6/6), baseline comparability (6/6), sample size calculation (6/6), accounted for clustering (5/6), masking (0/6), loss of clusters to follow-up (6/6), intention to treat analysis (6/6)

Wilson 2019 (42)	Unicompartimental knee arthroplasty	Venous thromboembolism	NR	NA	Cochrane	Sequence generation (1/2), allocation concealment (2/2), blinding (2/2), incomplete outcome data (1/2), selective outcome report (1/2), free of other bias (2/2)
Wilson 2019 (42)	Unicompartimental knee arthroplasty	Flexion-extension range	NR	NA	Cochrane	Sequence generation (0/3), allocation concealment (2/3), blinding (3/3), incomplete outcome data (3/3), selective outcome report (0/3), free of other bias (2/3)
Wilson 2019 (42)	Unicompartimental knee arthroplasty	Operation duration	NR	NA	Cochrane	Sequence generation (1/3), allocation concealment (1/3), blinding (3/3), incomplete outcome data (2/3), selective outcome report (1/3), free of other bias (2/3)
Yank 2011 (44)	Recombinant factor VII	All-cause mortality	Low (RCTs and cohort studies)	AHRQ/ GRADE	AHRQ/ based on various tools (JADAD, CONSORT and others)	Good (1/2), fair (1/2)
Yank 2011 (44)	Recombinant factor VII	Thromboembolism	Moderate (RCTs and cohort studies)	AHRQ/ GRADE	AHRQ/ based on various tools (JADAD, CONSORT and others)	Good (1/2), fair (1/2)
Zhang 2016 (94)	Everolimus-eluting bioresorbable vascular scaffold	Stent thrombosis	NR	NA	Cochrane	Random sequence generation (5/5), allocation concealment (4/5), blinding of personnel and participants (4/5), blinding of outcome assessment (5/5), incomplete outcome data (5/5), selective reporting (5/5), other bias (5/5)
Zhang 2016 (94)	Everolimus-eluting bioresorbable vascular scaffold	All-cause mortality	NR	NA	Cochrane	Random sequence generation (5/5), allocation concealment (5/5), blinding of personnel and participants (3/5), blinding of outcome assessment (5/5), incomplete outcome data (5/5), selective reporting (5/5), other bias (5/5)
Zhang 2016 (94)	Everolimus-eluting bioresorbable vascular scaffold	Coronary heart disease mortality	NR	NA	Cochrane	Random sequence generation (3/3), allocation concealment (3/3), blinding of personnel and participants (1/3), blinding of outcome assessment (3/3), incomplete outcome data (3/3), selective reporting (3/3), other bias (3/3)
Zhang 2017 (95)	Percutaneous coronary intervention	All-cause mortality	NR	NA	Cochrane	Random sequence generation (4/5), allocation concealment (4/5), blinding of personnel and participants (0/6), blinding of outcome assessment (5/5), incomplete outcome data (4/5), selective reporting (5/5), other bias (4/5)

Zhang 2017 (95)	Percutaneous coronary intervention	Cardiovascular mortality	NR	NA	Cochrane	Random sequence generation (4/4), allocation concealment (4/4), blinding of personnel and participants (0/4), blinding of outcome assessment (4/4), incomplete outcome data (3/4), selective reporting (4/4), other bias (3/4)
Zhang 2017 (95)	Percutaneous coronary intervention	Myocardial infarction	NR	NA	Cochrane	Random sequence generation (4/5), allocation concealment (4/5), blinding of personnel and participants (0/5), blinding of outcome assessment (5/5), incomplete outcome data (4/5), selective reporting (5/5), other bias (4/5)
Ziff 2015 (96)	Digoxin	All-cause mortality	NR	NA	Cochrane	Random sequence generation (3/7), allocation concealment (4/7), blinding of personnel (7/7), blinding of outcome (6/7), incomplete outcome data (6/7), selective reporting (3/7), other threats to validity (1/7)
Ziff 2015 (96)	Digoxin	Cardiovascular mortality	NR	NA	Cochrane	Random sequence generation (3/5), allocation concealment (4/5), blinding of personnel (5/5), blinding of outcome (5/5), incomplete outcome data (5/5), selective reporting (3/5), other threats to validity (1/5)
Ziff 2015 (96)	Digoxin	Hospital admission	NR	NA	Cochrane	Random sequence generation (2/2), allocation concealment (2/2), blinding of personnel (2/2), blinding of outcome (2/2), incomplete outcome data (2/2), selective reporting (2/2), other threats to validity (1/2)

AHRQ= agency for healthcare research and quality; CONSORT= consolidated standards of reporting trials; DDP-4= dipeptidyl peptidase 4; DHA= docosahexaenoic acid; EPA= eicosapentaenoic acid; GRADE= grades of recommendation, assessment, development, and evaluation; MINORS= methodological index for non-randomized studies; MOOSE= meta-analyses of observational studies in epidemiology; NA= not applicable; NOS= newcastle-ottawa scale; NR= not reported; NSTE-ACS= non-ST elevation acute coronary syndrome; QUATSO= quality assessment tool for systematic reviews of observational studies; RoB= risk of bias; SGLT-2= sodium glucose transporter 2; SIGN= scottish intercollegiate guidelines network; STROBE= strengthening the reporting of observational studies in epidemiology; UPSTF= united states preventive services task force.

Table S6. Characteristics of included bodies of evidence from cohort studies

Reference/ year	Intervention	Outcome	n (studies)	Sample size	Cases	Description of population	Age; mean/ range (years)	Description of exposure	Description of comparator	Description of outcome	Study design (cohort, nested case-control, case-cohort)	Follow-up (years)
Abou-Setta 2011 (74)	Nerve block	Delirium	2	634	66	Elderly population with hip fracture / health status: community dwelling or institutionalised / including patients with dementia (1x) or not specified (1x)	>50	Femoral nerve block with Bupivacaine 0.25% 30ml, maintenance: 0.1% Bupivacaine and IV analgesia with NSAID (1-2g Paracetamol)or Metamizol administered as patient-controlled analgesia (1x)/ 3-in-1 nerve block with Bupivacaine (Bolus of 100mg, maintenance with 50mg), administration every 8 hours	IV analgesia with Paracetamol or Metamizol 1-2g (1x)/ Preoperative: Morphine, Postoperative: Morphine tablets, Acetaminophen or Ibuprofen (1x)	Delirium	Retrospective cohort, retrospective cohort with historic controls	NR
Abou-Setta 2011 (74)	Spinal anesthesia	All-cause mortality	5	2,960	195	Elderly population with hip fracture / health status: mostly not specified, partly previously community dwelling without severe dementia (1x)	>65 to >75	Spinal anaesthesia, mostly with Bupivacaine / drugs and doses reported: Bupivacaine 0.5%, dosage: 2.5-5mg, continuous administration or single administration; Bupivacaine dosage: 8-15 mg, Mepivacaine 4 %, dosage: 2ml (80 mg)	General anaesthesia / drugs and doses reported: Fentanyl 3-5mg/kg; Sulfentanil; Thiopental	Mortality at 30 days	Prospective cohort, retrospective cohort	NR
Aburto 2013 (75)	Low sodium	All-cause mortality	7	21,515	NR	Male and female general population / with and without CVD and metabolic disease	25–74 (range)	Low sodium intake or excretion / partly: risks for outcomes per 1.9 g/day intake change (1x)	High sodium intake or status/ excretion / measured by: 24-hour dietary recall, overnight urinary sodium excretion, 24-hour sodium and potassium urinary excretion estimated from fasting morning urine, 24-hour urinary sodium concentration	All-cause mortality	Prospective cohort, cohort, case-cohort analysis	4.4-22 years

Aburto 2013 (75)	Low sodium	Cardiovascular disease	9	46,483	NR	Male and female general population / with and without CVD and metabolic disease	>15/ 25-79 (mean)	Low sodium intake or excretion / partly: risks for outcomes per 1.9 g/day intake change (1x)	High sodium intake or status/ excretion / measured or estimated by various methods (24-hour urinary sodium excretion, overnight urinary sodium excretion, 24-hour dietary recall, 24-hour urinary sodium concentration or excretion, food frequency questionnaire)	Cardiovascular disease (stroke, MI, CHD, coronary revascularisation, death from cardiovascular disease)	Prospective cohort, cohort, case-cohort analysis	<5-22 years
Ahmad 2015 (27)	Intra-aortic balloon pump	All-cause mortality	14	15,485	7.048	Population with acute CVD: cardiogenic shock (13/14), mostly caused by MI	NR	Intra-aortic balloon pump / mostly co-interventions for all patients: PCI (PCI) (5/14), thrombolysis (7/14), thrombolysis or PCI (1/14) / none (1/14)	No intra-aortic balloon pump, intensive care	Mortality at 30 days	Prospective cohort, cohort, retrospective cohort, observational analysis of RCT	NR
Alexander 2017 (76)	DHA and EPA	Coronary heart disease	17	687,166	NR	Male and female population / mostly healthy (without CVD at baseline), partly population with CVD / setting: highly developed countries	>18	High intake of EPA and DHA / assessed by food frequency questionnaire or cross-check dietary history method / high intake: >0.25 to >0.34 g/day (EPA only); 1.72g/day (mean) to 5.18g/day (median); 0.24 % of total kcal (1x); 0.51% of total fat (1x)	Low EPA and DHA intake / low intake: 0g/day to 1.48g/day (median); 0.05% of total fat (1x); median 0.03% of total kcal (1x)	Any CHD event (sudden cardiac death, coronary events, total MI, fatal MI, non-fatal MI, any CHD event, coronary death)	Prospective cohort	4.8-40 years
Alexander 2017 (76)	DHA and EPA	Coronary heart disease mortality	14	615,427	NR	Male and female population / mostly healthy (without CVD at baseline), partly population with CVD / setting: highly developed countries	>18	High intake of EPA and DHA / assessed by food frequency questionnaire or cross-check dietary history method / high intake: 0.22 to 2.64 g/day (mean/ median intake); >0.15 to >0.25 g/day; 0.51% of total fat (1x)/ 0.24% median of total kcal (1x)	Low EPA and DHA / low intake: <0.04 to <0.25g/day; 0-0.58 g/day (mean/median); 0.05% of total fat (1x); median 0.03 % of total kcal (1x)	Fatal CHD (fatal MI, sudden cardiac death, coronary death)	Prospective cohort	4.8-40 years

Alexander 2017 (76)	DHA and EPA	Coronary heart disease incidence	4	193,330	NR	Male and female population without CVD at baseline / setting: highly developed countries	>18	High intake of EPA and DHA / assessed by food frequency questionnaire / high intake: >0.19 g/day to >0.25 g/d; median 2.1 g/day (1x); median 0.24% of total kcal intake (1x)	Low EPA and DHA / low intake: <0.06g to <0.25g/day; median 0.03% of total kcal (1x); 0.3 median g/day (1x)	Non-fatal CHD (non-fatal MI; also: coronary events)	Prospective cohort	11-16 years
Alipanah 2018 (24)	Self-administered therapy	Low treatment success	16	19,211	13,846	Child and adult population / with: pulmonary or extrapulmonary tuberculosis, smear positive and negative / with or without: HIV-coinfection, previous treatment for tuberculosis / partly with: multidrug resistant tuberculosis / setting: countries with different levels of development (Brazil, China, Haiti, India, Japan, Nigeria, Poland, South Africa, Spain, Taiwan, Thailand)	≥15/ or not specified (including children)	Self-administered therapy / partly: partially directly-observed therapy (1x)	Directly observed therapy / providers: health staff in hospital or in community; community or family member	Low treatment success	Prospective cohort, retrospective cohort, cohort with historic controls	NR
Alipanah 2018 (24)	Self-administered therapy	Low treatment completion	14	11,679	3,469	Child and adult population / with: pulmonary or extrapulmonary tuberculosis, smear positive and negative / with or without: HIV-coinfection, previous treatment for tuberculosis / partly with: multidrug resistant tuberculosis or metabolic disease / setting: countries with different levels of development (Brazil, Haiti, India, Japan, Poland, Thailand, U.K., USA)	≥15/ or not specified (including children)	Self-administered therapy	Directly observed therapy / providers: health staff in hospital or in community; community or family member	Low treatment completion	Prospective cohort, retrospective cohort	NR

Alipanah 2018 (24)	Self-administered therapy	All-cause mortality	23	100,208	1,603	Child and adult population / with: pulmonary or extrapulmonary tuberculosis, smear positive and negative / with or without: HIV-coinfection, previous treatment for tuberculosis / partly with: multidrug resistant tuberculosis or metabolic disease / setting: countries with different levels of development (Brazil, China, Haiti, India, Japan, Nigeria, Poland, South Africa, Spain, Taiwan, Thailand, USA)	≥15/ or not specified (including children)	Self-administered therapy	Directly observed therapy / providers: health staff in hospital or in community; community or family member	Mortality	Prospective cohort, retrospective cohort; cohort with historic controls	NR
Anglemyer 2013 (77)	Antiretroviral therapy	HIV infection	9	97,898	>2,084	Serodiscordant couples (sexual partners) / mostly heterosexual, partly homosexual (2x) / either male or female partner HIV infected / populations with: drug abuse (2x), HSV-2 co-infection (1x) / setting: countries with or without a high state of development (Brazil, Botswana, China, Italy, Kenya, Rwanda, South Africa, Spain, Tanzania, Uganda, Zambia)	NR	ART for infected partners / various stages of disease at baseline (e.g. 500 cells/µL and >80% without AIDS (1x) or 250 cells/µL or WHO stage IV illness / mostly with three or more drugs, partly with: Zidovudine only (1x)	No ART	HIV infection (virologically linked and unlinked)	Prospective cohort, cohort, retrospective cohort	53.6 to 101,295.1 person-years of exposure/ <1.3 to 6 years
Azad 2017 (21)	Nonnutritive sweeteners	Body Mass Index	1	3,371	NA	Male and female general population (randomly chosen households) / mean BMI 27 / setting: USA	44 (mean)	Highest intake quantile (22 or more artificially sweetened beverages per week)	No artificially sweetened beverages	BMI change	Prospective cohort	8 years

Barnard 2015 (28)	Surgical abortion by mid-level providers	Failure or incomplete abortion	2	12,850	62	Pregnant women / mostly in 1st trimester of pregnancy / without uterine or cervical abnormalities (1x) / setting: USA	>16/ reproductive age	Abortion by experienced or specially trained mid-level providers (nurses, midwives, physician assistants) / procedures: manually, aspiration, vacuum curettage	Abortion by doctors (experienced or not specified) / vacuum curettage or not specified	Incomplete abortion (requiring a repeat abortion/ failed abortion)	Prospective cohort	2-4 weeks
Barnard 2015 (28)	Surgical abortion by mid-level providers	Complications	2	12,850	116	Pregnant women / mostly in 1st trimester of pregnancy / without uterine or cervical abnormalities (1x) / setting: USA	>16/ reproductive age	Abortion by experienced or specially trained mid-level providers (nurses, midwives, physician assistants) / procedures: manually, aspiration, vacuum curettage	Abortion by doctors (experienced or not specified) / vacuum curettage or not specified	Early complications (not specified/ according to National Abortion Federation guidelines); delayed complications up to 2-4 weeks	Prospective cohort	2-4 weeks
Barnard 2015 (28)	Surgical abortion by mid-level providers	Abortion failure and complications	3	15,308	249	Pregnant women / in 1st trimester of pregnancy / < 12 weeks (1x) to < 14 weeks gestation (2x) / population without: uterine or cervical abnormalities (1x) or pelvic inflammatory disease, increased bleeding risk (1x) / setting: USA	>16/ reproductive age	Abortion by experienced or specially trained mid-level providers (nurses, midwives, physician assistants), physician assistants without specified level of experience (1x) / procedures: manually, aspiration, vacuum curettage	Abortion by doctors (experienced or not specified (2x)) / vacuum curettage (1x), early uterine evacuation or suction curettage (1x), procedure not specified (1x)	Early complications during the procedure or before leaving the facility (not specified/ according to National Abortion Federation guidelines); delayed complications up to 2-4 weeks; Ectopic/ extrauterine pregnancy, perforation, cervical lacteration, infection, haemorrhage, other complications	Prospective cohort	2-4 weeks
Bellemain-Appaix 2012 (48)	Clopidogrel	All-cause mortality	8	29,206	799	Adult population with CAD/CHD scheduled for catheterisation, PCI, or both	NR	Clopidogrel 300 to ≥600mg loading dose several days to directly before procedure or 75mg maintenance dose ≥ 5 days (2x) / subsequent maintenance dose or loading dose administered some hours before procedure (2x) or not specified (1x)	Clopidogrel 300-600mg peri-interventionally or lower loading dose	All-cause mortality	Prospective cohort, retrospective cohort, observational analysis of RCT	In hospital to 1 year (range)

Bellemain-Appaix 2012 (48)	Clopidogrel	Major bleeding	8	29,232	611	Adult population with CAD/CHD scheduled for catheterisation, PCI, or both	NR	Clopidogrel 300 to ≥600mg loading dose several days to directly before procedure or 75mg maintenance dose ≥ 5 days (2x) / subsequent maintenance dose or loading dose administered some hours before procedure (2x) or not specified (1x)	Clopidogrel 300-600mg peri-interventionally or lower loading dose	Major bleeding; definitions given (TIMI minor/ major, vascular complication, transfusion, ≥ 4g/dl decrease in Hb, intracranial, ≥5g/dl Hb loss, requiring transfusion or surgery, clinically significant bleeding)	Prospective cohort, retrospective cohort, observational analysis of RCT	In hospital to 1 year (range)
Bellemain-Appaix 2012 (48)	Clopidogrel	Coronary heart disease	8	29,206	3,350	Adult population with CAD/CHD scheduled for catheterisation, PCI, or both	NR	Clopidogrel 300 to ≥600mg loading dose several days to directly before procedure or 75mg maintenance dose ≥ 5 days (2x) / subsequent maintenance dose or loading dose administered some hours before procedure (2x) or not specified (1x)	Clopidogrel 300-600mg peri-interventionally or lower loading dose	Major coronary event (Death, Myocardial infarction, urgent target vessel revascularization, recurrent acute coronary syndrome)	Prospective cohort, retrospective cohort, observational analysis of RCT	In hospital to 1 year (range)
Bellemain-Appaix 2014 (47)	P2Y12 inhibitors	All-cause mortality	4	11,175	186	Adults undergoing PCI / 57.4-100% (range) with non-ST elevation ACS	59.4-67.3 (mean)	Clopidogrel pretreatment for PCI, ≥300mg Clopidogrel >12 hours or shortly before PCI or maintenance dose starting days before PCI	Delayed treatment with ≥300mg Clopidogrel / shortly before, during or shortly after PCI	All-cause death; in hospital (2x) or at 30 days (2x)	Retrospective cohort, observational analysis of RCT	In hospital to 1 year (range)
Bellemain-Appaix 2014 (47)	P2Y12 inhibitors	Major bleeding	4	11,188	341	Adults undergoing PCI / 57.4-100% (range) with non-ST elevation ACS	59.4-67.3 (mean)	Clopidogrel pretreatment for PCI, ≥300mg Clopidogrel >12 hours or shortly before PCI or maintenance dose starting days before PCI	Delayed treatment with ≥300mg Clopidogrel / shortly before, during or shortly after PCI	Major bleeding; in hospital (2x) or at 30 days (2x); definitions: Thrombolysis in Myocardial Infarction major bleeding criteria (1x); ≥4 g/dL drop in Hb (1x); IC, transfusion, hemodynamic compromise (1x); clinically significant (1x)	Retrospective cohort, observational analysis of RCT	In hospital to 1 year (range)

Bellemain-Appaix 2014 (47)	P2Y12 inhibitors	Main composite ischemic endpoint	4	11,188	848	Adults undergoing PCI / 57.4-100% (range) with non-ST elevation ACS	59.4-67.3 (mean)	Clopidogrel pretreatment for PCI, ≥300mg Clopidogrel >12 hours or shortly before PCI or maintenance dose starting days before PCI	Delayed treatment with ≥300mg Clopidogrel / shortly before, during or shortly after PCI	Main composite ischemic endpoint; in hospital (2x) or at 30 days (2x)	Retrospective cohort, observational analysis of RCT	In hospital to 1 year (range)
Bloomfield 2016 (22)	Mediterranean diet	Breast cancer	13	805,893	NR	Female population / general population or healthy (no history of cancer) / setting: highly developed countries	38.5-62 (mean/median)	High adherence to mediterranean diet or healthy diet / assessment with: variants or classic mediterranean diet score, other	Low adherence to mediterranean diet, healthy diet	Breast cancer incidence	Prospective cohort (including cohort-subcohort controlled study, reanalysis from prospective cohort)	7-25 years
Bolland 2015 (49)	Calcium	All fractures	5	116,199	11,194	Mostly female population (women (3x), mixed (1x), men (1x)) / general population or basically healthy (3x): community dwelling, without risk factors for osteoporosis, able to walk	50-79 (range)/ 56-81 (mean)	Mostly calcium supplement use with or without dietary calcium intake / partly additional vitamin D supplement use / dietary calcium intake only (1x)	No use of calcium supplements / never used calcium supplements (1x)	All fractures	Prospective cohort	3-8.4 years (mean)
Bolland 2015 (49)	Calcium	Vertebral fracture	1	9,704	389	Female general population / without: women unable to walk	72 (mean)	Current use of calcium supplements	Never used calcium supplements	Vertebral fracture	Prospective cohort	6.6 years
Bolland 2015 (49)	Calcium	Hip fracture	6	270,446	2,828	Mostly female population (women (3x), mixed (2x), male (1x), NR(1x)) / mostly general population	50-79 (range)/ 54-73 (mean)	Mostly calcium supplement use / partly calcium and vitamin D (1x)	No use of calcium supplements / never used calcium supplements (1x)	Hip fracture	Prospective cohort	6.6-18 years
Brenner 2014 (29)	Sigmoidoscopy	Colorectal cancer mortality	1	88,902	NR	Male and female generally healthy population (health professionals, nurses) / without: baseline history of cancer, at risk for colorectal cancer / setting: highly developed countries	62.5-68.3 (mean)	Sigmoidoscopy	No sigmoidoscopy	Mortality from colorectal cancer	Prospective cohort	25 years

Brenner 2014 (29)	Sigmoidoscopy	Colorectal cancer incidence	2	2,966	NR	Male and female general population / at average risk for colorectal cancer (1x) or without further exclusion criteria (1x) / setting: highly developed countries;	55-85 (range)	Sigmoidoscopy	No sigmoidoscopy	Colorectal cancer; late stages of colorectal cancer (2b to 4) only (1x)	Prospective cohort, nested case-control	<10 years
Chowdhury 2012 (78)	Omega-3	Cerebrovascular disease	10	301,023	4,197	Male and female population / general population, healthy at baseline / mostly in highly developed countries	16-84 (range)	Omega-3 fatty acid intake / assessed with food frequency questionnaire or dietary recall / average intake (range) 0.11 g/day - 2.60 g/day	Low Omega-3 fatty acid intake	Cerebrovascular disease	Prospective cohort	4-28 years (mean)
Chowdhury 2014a (79)	α-linolenic acid	Coronary heart disease	7	157,258	7,431	Male (5x), female (1x) or mixed (1x) population / mostly healthy, partly with CVD (1x) / setting: highly developed countries	30-84 (range)	α-linolenic acid intake / assessed with food frequency questionnaire, diet history questionnaire, 7-day weighed food record, 4-day food record	Low intake of α-linolenic acid	Cardiovascular disease (non-fatal myocardial infarction, fatal coronary heart disease, sudden cardiac death)	Prospective cohort	5-20 years
Chowdhury 2014a (79)	Omega-3	Coronary heart disease	16	422,786	9,089	General or healthy male and female population / partly with pre-existing CHD (1x)	18-84 (range)	Dietary long chain omega-3 fatty acid intake / assessed with food frequency questionnaire, 7-day food diary, Diet history questionnaire, 4-day food record, 7-day weighed food record	Low intake	Coronary disease (myocardial infarction, coronary heart disease, sudden cardiac death, angina pectoris)	Prospective cohort	5-23 years
Chowdhury 2014a (79)	Omega-6	Coronary heart disease	8	206,376	8,155	General or healthy male and female population / partly with risk for CHD (1x)	20-75 (range)	Dietary long chain omega-6 fatty acid intake / assessed with food frequency questionnaire, 7-day food diary, diet history questionnaire, 4-day food record, 7-day weighed food record	Low intake	Coronary disease (myocardial infarction, coronary heart disease, sudden cardiac death)	Prospective cohort	5-20 years

Chowdhury 2014b (80)	Vitamin D	All-cause mortality	68	840,908	64,636	Male and female population with or without pre-existing chronic disease (CVD, metabolic disease, cancer) / community dwelling or institutionalised / setting: mostly Europe and North America, partly Asia-Pacific Region and South America (1x)	29-84 (range)	High circulating 25-hydroxyvitamin D (Serum or Plasma) / average level 10-30 ng/ml	Low circulating 25-hydroxyvitamin D (Serum and Plasma)	All-cause mortality	Prospective cohort, retrospective cohort	0.3-29 years
Chung 2011 (58)	Vitamin D	Colorectal cancer	9	2,249	1,127	Male and female general population / partly smokers (2x) / setting: USA, Europe and Japan	57- 69.2 (mean)/ 40-79 (range)	10nmol/L increase in 25-hydroxyvitamin D blood concentration	NA	Colorectal cancer (colorectal cancer, Invasive CRC (1x), Colon cancer (1x))	Nested-case control	7 months to 17 years (range)
Chung 2011 (58)	Vitamin D	Breast cancer	4	4,726	2,363	Female general population / pre- and postmenopausal / setting: USA and Sweden (1x)	57-70 (mean)/ 30-85 (range)	10nmol/L increase in 25-hydroxyvitamin D blood concentration	NA	Breast cancer	Nested-case control	<1 month to 15 years (range)
Chung 2016 (56)	Calcium	Cardiovascular mortality	6	160,954	NR	Male and female population / general population or without CVD at baseline	54-61 (mean)/ 25-78 (range)	Risk per 100mg/day increase of total calcium intake / mean calcium intake 400–2,400mg/day	NA	Cardiovascular (4x) or ischemic heart disease (2x) mortality	Prospective cohort	8–19 years
Ding 2017 (81)	Dairy	Systolic blood pressure	27	165,464	NR	Male and female general population / partly with CVD	20-84 (range)	Dairy intake / mean 1.09-4.8 servings/day; 456g intake per day / change of systolic blood pressure per serving increase	Dairy intake / mean 0-0.93 servings/day; 84g intake per day; information not complete for all studies	Systolic blood pressure assessment: mostly clinical (16/27), self reported (2/27) or NR (9/27)	Prospective cohort, cohort	0-17 years follow-up
Fenton 2018 (30)	Radiation therapy	Erectile dysfunction	7	2,950	1,625	Male population with localised prostate cancer / partly with all stages (1x)	61.2-76 (mean/median)/ 37-88 (range)	Radiation therapy	Conservative management / no active treatment, active surveillance, not further specified	Erectile dysfunction; defined as erection not sufficient for sexual intercourse or partly presence of at least one troublesome symptom (1x)	Prospective cohort, retrospective cohort	1-10 years

Fenton 2018 (30)	Radical Prostatectomy	Urinary incontinence	5	5,067	911	Male population with localised prostate cancer	61.2-69 (mean/ median)/ 37-88 (range)	Radical prostatectomy	Conservative management / no active treatment, active surveillance, not further specified	Urinary incontinence; perceived as troublesome by patients, need for pads or frequent symptoms	Prospective cohort	1-3.8 years (mean/median)
Fenton 2018 (30)	Radical Prostatectomy	Erectile dysfunction	6	5,558	3,525	Male population with localised prostate cancer	61.2-69 (mean/ median)/ 37-88 (range)	Radical prostatectomy	Conservative management / no active treatment, active surveillance, not further specified	Erectile dysfunction defined as erection not sufficient for sexual intercourse; partly presence of at least one troublesome symptom (1x)	Prospective cohort, cohort	1-4.3 years (mean/median)
Filippini 2017 (43)	Disease-modifying drugs	Conversion to clinically definite multiple sclerosis	2	3,592	NR	Female (>70%) and male population / with: signs and symptoms compatible with early multiple sclerosis (and almost all with silent lesions in MRI) / mostly in highly developed countries	32.5 (mean)/ 31.6 (median)	Different disease modifying drugs (e.g. Interferon beta-1a, Interferon beta-1b, glatiramer actetate, others not specified), SC, IM or P.O. / early or delayed treatment (1/2)	No use of disease modifying drug	Clinically definite multiple sclerosis, assessment by EDSS-score or Poser-criteria	Prospective cohort	2 years/ 5,378.70 person-years
Fluri 2010 (31)	Extracranial-intracranial arterial bypass	All-cause mortality	11	690	85	Population with occlusive carotid artery disease / most participants included irrespective of cerebral haemodynamics	NR	Extracranial-intracranial arterial bypass plus medical treatment	Best medical treatment / insufficient information, if reported various regimens: Aspirin with or without Dipyridamole, Clopidogrel, Warfarin	All-cause death	Prospective cohort, retrospective cohort	NR
Fluri 2010 (31)	Extracranial-intracranial arterial bypass	Stroke	15	796	132	Population with occlusive carotid artery disease / most participants included irrespective of cerebral haemodynamics	NR	Extracranial-intracranial arterial bypass plus medical treatment	Best medical treatment / insufficient information, if reported various regimens: Aspirin with or without Dipyridamole, Clopidogrel, Warfarin	Any stroke	Prospective cohort, retrospective cohort	NR

Fluri 2010 (31)	Extracranial-intracranial arterial bypass	Stroke mortality or dependency	8	346	131	Population with occlusive carotid artery disease / most participants included irrespective of cerebral haemodynamics	NR	Extracranial-intracranial arterial bypass plus medical treatment	Best medical treatment / insufficient information, if reported various regimens: Aspirin with or without Dipyridamole, Clopidogrel, Warfarin	Death or dependency	Retrospective cohort	NR
Gargiulo 2016 (32)	Transcatheter aortic valve	Early all-cause mortality	29	12,464	592	Male and female population with severe CVD / mostly including patients at high surgical risk (22x) or only patients at low or intermediate risk (7x) / partly population with: metabolic disease, kidney disease, pulmonary disease	71.1-82.7 (mean)/ 60-94 (range)	Transcatheter aortic valve replacement / mostly by femoral access / first generation valve, partly Sapien 3 valve (1x)	Surgical aortic valve replacement	Early all-cause mortality	Prospective cohort, retrospective cohort with or without historic controls, retrospective database cohort	2-5 years (6x/ NR)
Gargiulo 2016 (32)	Transcatheter aortic valve	Mid-term all-cause mortality	18	7,400	998	Male and female population with severe CVD / mostly including patients at high surgical risk (11x) or only patients at low or intermediate risk (7x) / partly population with: metabolic disease, kidney disease, pulmonary disease	71.1-82.7 (mean)/ 60-94 (range)	Transcatheter aortic valve replacement / mostly by femoral access / first generation valve, partly Sapien 3 valve (1x)	Surgical aortic valve replacement	Midterm all-cause mortality	Prospective cohort, retrospective cohort with or without historic controls, retrospective database cohort	2-5 years (3x/ NR)
Gargiulo 2016 (32)	Transcatheter aortic valve	Long-term all-cause mortality	6	1,750	316	Male and female population with severe CVD / all including patients at high surgical risk / partly population with: metabolic disease, kidney disease, pulmonary disease	78.2-82.3 (mean)	Transcatheter aortic valve replacement / mostly by femoral access / first generation valve	Surgical aortic valve replacement	Long-term all-cause mortality	Retrospective cohort	2-5 years
Hartling 2013 (50)	Treating gestational diabetes mellitus	High birth weight	5	3,168	331	Pregnant women with borderline, mild or definite GDM / mean fasting glucose reported for one study (5.4 mmol/l), BMI 23.11-30.3 / population without complicated pregancies (e.g. without multiple gestation, fetal anomaly) or not specified / ethnicity: asian, black, hispanic, white	27.6- 34.4 (mean)	Dietary counseling, formal diet (3x) e.g. with caloric restriction / blood glucose monitoring, insulin if required (5x)	Standard care	Birth weight > 4000g	Retrospective cohort	NR

Hartling 2013 (50)	Treating gestational diabetes mellitus	Large-for-gestational age neonate	4	2,294	388	Pregnant women / mostly mild or borderline GDM, partly definite GDM (1x) / mean fasting glucose reported for one study (5.4 mmol/l), BMI: 23.12-30.3 / complicated pregnancies (e.g. multiple gestation) excluded or not specified / ethnicity: asian, black, hispanic, white	27.6-31.5 (mean)	Dietary counseling, formal diet (2x) e.g. with caloric restriction / blood glucose monitoring, insulin if required (4x)	Standard care	Large-for-gestational age neonate	Retrospective cohort	NR
Hartling 2013 (50)	Treating gestational diabetes mellitus	Shoulder dystocia	4	3,054	48	Pregnant women with mild GDM or all types of GDM / mean fasting glucose reported for one study (5.4 mmol/l), BMI: 23.11-30.3 / complicated pregnancies (e.g. multiple gestations) excluded or not specified / ethnicity: asian, black, hispanic, white	27.6-34.4 (mean)	Dietary counseling, formal diet (3x) e.g. with caloric restriction / blood glucose monitoring, insulin if required (4x)	Standard care	Shoulder dystocia	Retrospective cohort	NR
Henderson 2019 (51)	Treating asymptomatic bacteriuria	Pyelonephritis	2	5,289	74	Pregnant women / with and without asymptomatic bacteriuria / < 25 weeks to < 33 weeks of gestation / no exclusion criteria reported / setting: Turkey and Spain	27.7 (mean)/ NR (1x)	Treating screen detected asymptomatic bacteriuria / different antibiotics	No screening (and treatment) for asymptomatic bacteriuria	Pyelonephritis	Cohort with historic controls	NR
Higgins 2016 (25)	Bacillus Calmette-Guérin	All-cause mortality	8	12,225	>826	Infants and children / mostly no exclusion criteria specified / with DTP vaccination, with or without measles vaccination / low birth weight children (2x) / setting: Guinea-Bissau, India, Malawi, Papua-New Guinea, Senegal	29 days to 23 months/ NR	BCG vaccination	No BCG vaccination	All-cause mortality	Prospective cohort, retrospective cohort, retrospective database cohort, cohort nested in RCT	<0.5-5 years

Higgins 2016 (25)	Measles containing vaccines	All-cause mortality	13	82,208	>1,290	Infants and children / mostly no exclusion criteria specified / with and without prior DTP or BCG vaccination / infants with low birth weight (1x) / setting: Bangladesh, Guinea-Bissau, Haiti, India, Malawi, Papua New-Guinea, Senegal	<4 months to <35 months/ NR	Measles containing vaccine / number of doses unclear / timing (range) 0-13 months or not reported / monovalent (2x), Schwarz or Edmonston-Zagreb (2x) or not reported (11x)	No measles containing vaccines	All-cause mortality	Prospective cohort, retrospective cohort, cohort with historic controls	2-5 years
Hopley 2010 (33)	Total hip arthroplasty	Reoperation	6	787	54	Mostly female population with displaced femoral neck fracture / prior ambulatory patients only (3x), mostly no specific information on health status (mean ASA 3 (1x), without severe cognitive impairment (1x)) / note: the study conducted in women with mean age 59 years was conducted in India in a setting with reduced life expectancy	59-84 (mean)	Total hip arthroplasty, cemented (5x) / cemented or uncemented (1x)	Uni- or bipolar, cemented or uncemented hemiarthroplasty	Reoperation	Retrospective cohort	1-71 months (range of mean follow-up)
Hopley 2010 (33)	Total hip arthroplasty	Dislocation	5	721	22	Mostly female population with displaced femoral neck fracture / prior ambulatory patients only (2x), mostly no specific information on health status (mean ASA 3 (1x), without severe cognitive impairment (1x)) / note: the study conducted in women with mean age 59 years was conducted in India in a setting with reduced life expectancy	59-84 (mean)	Total hip arthroplasty, cemented (4x) / cemented or uncemented (1x)	Uni- or bipolar, cemented or uncemented hemiarthroplasty	Dislocation	Retrospective cohort	1-70 months (range of mean follow-up)
Hopley 2010 (33)	Total hip arthroplasty	Deep infection	4	806	10	Mostly female population with displaced femoral neck fracture / mostly no specific information on health status (mean ASA 3 (1x), without severe cognitive impairment (1x), prior ambulatory patients only (1x))	72-84 (mean)	Total hip arthroplasty, cemented	Uni- or bipolar, cemented or uncemented hemiarthroplasty	Deep infections	Retrospective cohort	1-70 months (range of mean follow-up)

Hüpfl 2010 (67)	Standard cardiopulmonary resuscitation	Survival	7	13,883	1,086	Population with out-of hospital cardiac arrest	NR	Bystander standard cardiopulmonary resuscitation (including rescue ventilation), without instructions or assisstance	Bystander chest-compression-only cardiopulmonary resuscitation, without instructions or assisstance	Survival to hospital discharge or closest available endpoint: 30-day survival, 1-week survival, awake after 14 days	Prospective cohort, retrospective cohort, cohort	Survival to hospital discharge to 1 year (range)
Jamal 2013 (82)	Non-calcium-based phosphat binders	All-cause mortality	3	2,813	791	Male and female population with advanced kidney disease requiring haemodialysis	61.5-68 (mean)	Non-calcium-based phosphat binder (Sevelamer)	Calcium-based phosphat binders (calcium acetate or calcium carbonat)	All-cause mortality	Prospective cohort, retrospective cohort	2-3.5 years
Jefferson 2010 (46)	Parenteral influenza vaccine	Influenza-like illness	30	22,001	2,268	Elderly population / generally healthy community-dwelling or institutionalised (nursing home residents, health status mostly not further specified) / partly: chronically ill nursing home residents requiring intermediate and skilled nursing care, suffering from dementia, bed ridden	71-85 (mean)/ 30 to 108 (range)	Parenteral influenza vaccines / mostly trivalent / matching or not matching the circulating strains (during or not during outbreak) / partly: amantadine prophylaxis for nursing home residents (either all, ill residents or healthy residents)	No vaccination	Influenza like illness mostly clinically defined (e.g. fever, chills, congestion, cough, coryza, sore throat, general malaise, myalgia) / partly laboratory, epidemiological and clincal criteria	Prospective cohort, cohort, retrospective cohort	< 1-5 months
Jefferson 2010 (46)	Parenteral influenza vaccine	Influenza	10	20,190	185	Elderly population / community-dwelling, general elderly population or institutionalised (in nursing homes with or without known diseases)	80-85 (mean)/ 36-105 (range)	Parenteral influenza vaccines / mostly trivalent / strains mostly matching the circulating strains in epidemic or non-epidemic years	No vaccination	Mostly laboratory confirmed influenza (4-fold increase in antibody titre/ not specified); Influenza as defined by International Classification for primary care (1x, R80: proven influenza without pneumonia)	Prospective cohort, retrospective database cohort, retrospective cohort	1-10 months

Jefferson 2012 (34)	Inactivated influenza vaccines	Influenza	1	1,302	35	Male and female infants and children / general population / setting: Japan	0-15 (range)	Inactivated influenza vaccine / one or two doses / sliding scale of doses according to age / unclear if tri-or bivalent / (cohort study from 2003)	No vaccination	Influenza defined as: Influenza-like illness plus rapid test diagnosis, or serum antibody increase or viral isolation	Prospective cohort	<8 months
Jefferson 2012 (34)	Inactivated influenza vaccines	Influenza-like illness	2	4,215	1,366	Male and female infants and children / general population / setting: Japan	0-15 (range)	Inactivated influenza vaccines / one or two doses / trivalent or NR / dosing according to age / (cohort studies from 2003/ 2006)	No vaccination	Influenza-like illness; clinical definiton: sudden onset, temperature over 38 °C, sore throat and fatigue/ acute febrile illness occurring during the highest epidemic period	Prospective cohort	<8 months
Jin 2012 (83)	Total flavonoids	Colorectal neoplasms	3	72,320	1,022	General population / pre- and postmenopausal women, men from the the health professionals follow-up study (1x), woman from the Nurses´ Health study (1x) / setting: USA	>45/ (NR 2x)	High flavonoid intake	Low flavonoid intake	Colorectal cancer	Prospective cohort, sub-cohort controlled cohort	NR
Johnston 2019 (23)	Low red meat	All-cause mortality	24	545,071	NR	Male and female population, generally healthy	33-74.1 (mean)	Lower adherence to diet high in red or processed meat (western diet, diet with pasta and meat, unhealthy diet, others) or vegetarian diet	Higher adherence to diet rich in red and processed meat or non-vegetarian diet	All-cause mortality	Prospective cohort	4-26 years
Johnston 2019 (23)	Low red meat	Cardiovascular mortality	25	858,554	NR	Male and female population, generally healthy	33-67 (mean)	Lower adherence to diet high in red or processed meat (western diet, diet with pasta and meat, unhealthy diet, others) or vegetarian diet	Higher adherence to diet rich in red and processed meat or non-vegetarian diet	Cardiovascular mortality	Prospective cohort	4-26 years
Johnston 2019 (23)	Low red meat	Cardiovascular disease	12	113,737	NR	Male and female population, generally healthy	38.2-67 (mean)	Lower adherence to diet high in red or processed meat (mostly western diet, southern diet, others) or vegetarian diet	Higher adherence to diet rich in red and processed meat or non-vegetarian diet	Cardiovascular disease	Prospective cohort	4-17 years

Kansagara 2013 (52)	Transfusion	All-cause mortality	11	172,988	NR	Male and female population with anemia / setting: non-operative, patients with acute CVD, mostly MI / partly: population undergoing vascular surgery or orthopedic surgery (1x)	59-77.8 (mean)	Received transfusions; nadir of haematocrit 26.5-29.2% (mean/ median) / haemoglobin 8.7-10g/dL	Received no transfusions	Mortality at 30 days; mortality or myocardial infarction at 30 days (2x)	Observational analysis of RCT, retrospective cohort, retrospective database cohort	>30 days-4 years
Keag 2018 (84)	Caesarean section	Urinary incontinence	8	65,842	NR	Female general population / primiparae only (3/8) / all types of birth presentations, without: multiple gestation (4x), risk pregnancy (1x), previous pelvic organ prolapse or urinary incontinence surgery (1x) / setting: mostly highly developed countries	>18/ <80 (at assessment)	Caesarean section	Vaginal birth	Urinary incontinence	Prospective cohort, retrospective cohort	1-21 years/ until age of 65 (1x)
Keag 2018 (84)	Caesarean section	Fecal incontinence	5	43,260	NR	Female general population / primiparae only (3/5) / all types of birth presentations / setting: in highly developed countries	>18/ <80 (at assessment)	Caesarean section	Vaginal birth	Fecal incontinence	Prospective cohort, retrospective cohort	1-20 years
Kredo 2014 (85)	Starting and maintaining antiretroviral therapy	All-cause mortality	2	39,160	3,771	Female and male population / median baseline CD4 117-119 cells/µL (1x), with advanced HIV-infection (CD4 count <200 cells/µL, WHO clinical stage 4) (1x) / setting: Ethiopia and rural South Africa	Adults/ NR	ART task-shifting from doctors to nurses and decentralisation to peripheral health offers / initiation and maintenance of ART by nurses and health officers and referral to doctors in case of treatment failure or severe manifestation; additional support with mobile physician team, adherence counselors and patient support groups (1x) / various ART regimens	Initiation and maintenance of ART by doctors at hospital	Death at 12 months	Retrospective cohort	1-2 years maximum follow-up (range)

Kredo 2014 (85)	Starting and maintaining antiretroviral therapy	Attrition	2	39,156	11,038	Female and male population / median baseline CD4 117-119 cells/µL (1x), with advanced HIV-infection (CD4 count <200 cells/µL, WHO clinical stage 4)(1x) / setting: Ethiopa and rural South Africa	Adults/ NR	ART task-shifting from doctors to nurses and decentralisation to peripheral health offers / initiation and maintenance of ART by nurses and health officers and referral to doctors in case of treatment failure or severe manifestation; additional support with mobile physician team, adherence counselors and patient support groups (1x) / various ART regimens	Initiation and maintenance of ART by doctors at hospital	Lost to follow-up 12 months	Retrospective cohort	1-2 years maximum follow-up (range)
Kredo 2014 (85)	Maintaining antiretroviral therapy	All-cause mortality	1	2,772	34	Male and female population / on ART without complications, eligible for down-referral (ART for >11 months, no opportunistic infections, stable weight, virologically suppressed <400 copies/mL, CD4 cell > 200 cells/µL) / median CD4 at down referral 389 cells/µL, on ART for median 30 months / setting: peri-urban South Africa	35.3 (mean)	ART task-shifting from doctors to nurses and decentralisation to peripheral health offers / maintenance by nurses (patients down-referred by doctors) and referral to doctors in case of complications (e.g. toxicity, detectable viral load) / various ART regimens / co-intervention (all patients) adherence counselling	Initiation and maintenance of ART by doctors at advanced hospital (were not offered down-referral or refused) / co-intervention (all patients) adherence counselling	Death at 12 months	Retrospective cohort	1 year follow-up
Li 2014 (54)	Exenatide	Acute pancreatitis	2	307,176	<1,466	Male and female population with diabetes / no pancreatitis or pancreatic cancer at baseline / mean diabetes duration 3.1 years (1x) / 59.7-81.5% receiving Metformin at baseline (1x) / setting: USA	52.7-63.1 (mean)	Exenatide	No Exenatide (1x) / known exposure to other antidiabetic drug (Sulfonylurea, Biguanide, Thiazolidinedione) (1x)	Acute pancreatitis (1x)/ Admission for acute pancreatitis (1x)	Retrospective database cohort	>1 year
Li 2016 (53)	DDP-4 inhibitors	Heart failure	4	21,435	998	Male and female population with diabetes / with or without CVD at baseline / median BMI 32.6 (1x), median HbA1c 7.3% (1x), median diabetes duration 4.9 years (1x) / setting: Europe	55 (mean) to 65.8 (median)	DDP-4 inhibitors (any) with or without Metformin (2x) or Sitagliptin with or without Metformin (2x)	Sulfonylureas with or without Metformin (3x) or no use of Sitagliptin (1x)	Heart failure	Prospective cohort, retrospective cohort	1-4 years (mean/ median)

Li 2016 (53)	DDP-4 inhibitors	Hospital admission for heart failure	6	1,618,295	4,341	Male and female population with diabetes / with or without CVD at baseline / HbA1c 8.0% (1x) or not reported (5x), mean diabetes duration 2.3-2.5 years	58.3-67 (mean)	DDP-4 inhibitors (any)	Sulfonylureas, Pioglitazone or other oral antidiabetic drugs, not specified (1x)	Hospital admission for heart failure	Retrospective cohort, nested-case control	0.5-2.6 years (mean/ median)
Matthews 2018 (86)	Tamoxifen	Heart failure	2	29,562	NR	Female postmenopausal population with breast cancer (primary breast cancer survivors of all stages or stage I-II breast cancer, estrogen- or progesterone-receptor-positive breast cancer) / without CVD	45-69 (range)	Tamoxifen use	No use of Tamoxifen (e.g. treatment with aromatase-inhibitors)	Heart failure	Retrospective database cohort	72886 person-years/ <10 years
Menne 2019 (87)	SGLT-2 inhibitors	Acute kidney injury	5	83,934	777	Population with diabetes (e.g. members of health plan, registered as antidiabetic drug users) with standard care background therapy / partly: population with advanced kidney disease only (eGFR <60 mL/min) (1x)	NR	SGLT-2 Inhibitors / drugs: any of Canagliflozin, Empagliflozin, Dapagliflozin or Dapagliflozin only (1x)	No SGLT-2 Inhibitor use, treatment with other antidiabetic drugs (e.g. DDP 4-inhibitors, GLP-1 receptor agonists)	Any acute kidney injury / varying definitions (different ICD codes, hospitalisation for acute kidney injury, hospitalisation for kidney disease)	Retrospective cohort, retrospective database cohort	24-62 weeks (follow-up)
Mesgarpour 2017 (88)	Erythropoiesis stimulating agents	Venous thromboembolism	5	13,963	180	Male and female population / medical or surgical ICU with or without anemia (3/5 populations with definite anemia, defined for 1x: Hb<12g/dL) / reasons for admission: trauma, burns / only patients receiving enoxaparin or unfractionated heparin (2x)	>16	Erythropoiesis stimulating agents / drugs and dosing: any (2x); SC rHuEPO 40,000 IU/week; Darbepoetin alfa; SC rHuEPO 100 IU/kg or Darbepoetin 0.45 µg/kg weekly	No erythropoiesis stimulating agents	Venous thromboembolism	Retrospective cohort, retrospective database cohort	30 days/ NR (4x)

Mesgarpour 2017 (88)	Erythropoiesis stimulating agents	All-cause mortality	7	>924,791	NR	Male and female population / critically-ill and at ICU / with definite anemic population (2x) / other specified admission reasons: trauma, severe burns, severe traumatic brain injury	>16	Erythropoiesis stimulating agents for haematopoetic indications / drugs: Darbepoetin alfa, Epoetin alfa, rHuEPO or non-rHuEPO; prescription of any erythropoesis stimulating agent (1x)	No erythropoiesis stimulating agents	Mortality; at 30 days or not specified	Retrospective cohort, retrospective cohort with historic controls	30 days or not reported
Moberley 2013 (89)	Pneumococcal polysaccharide vaccines	Invasive pneumococcal disease	2	58,606	NR	Male and female elderly general population / members of health insurance or community dwelling / setting: in high-income countries	>65	Pneumococcal vaccine: 23-valent PPV	No vaccination	Invasive pneumococcal disease	Prospective cohort, retrospective cohort	<3,3 years
Molnar 2015 (35)	Neoral (Cyclosporin)	Acute rejection of kidney transplant	2	219	72	Adult population with kidney transplant / either incident transplants (without graft failure within 14 days post transplantation) or all living recipients / setting: Macedonia and USA	38.6-51.2 (mean)	Neoral (Cyclosporin)	Equoral, Gengraf (generic Cyclosporin)	Acute rejection of kidney transplant	Retrospective cohort with historic controls	0.5 years
Navarese 2013 (90)	Early intervention for NSTE-ACS	All-cause mortality	4	77,499	2,995	Male and female population with non-ST elevation ACS / undergoing PCI, CABG or medical treatment / with or without: positive biomarkers, metabolic disease, ST-segment depression, medication with glycoprotein IIb/IIIa inhibitors	62.5-68 (mean)	Early intervention for NSTE-ACS / time to intervention ≤24 or <24 hours (3x); 23.4 hours (mean, 1x)	Delayed intervention / time to intervention >24 hours (2x); >48 hours (1x); 46.3 hours (mean, 1x)	Mortality	Observational analysis of RCT, prospective cohort	Hospital discharge to 12 months
Navarese 2013 (90)	Early intervention for NSTE-ACS	Myocardial infarction	3	70,253	3,182	Male and female population with non-ST elevation ACS / undergoing PCI, CABG or medical treatment / with or without: positive biomarkers, metabolic disease, ST-segment depression, medication with glycoprotein IIb/IIIa inhibitors	62.5-68 (mean)	Early intervention for NSTE-ACS / time to intervention ≤24 hours (2x); 23.4 hours (mean, 1x)	Delayed intervention / time to intervention >24 hours (2x); 46.3 hours (mean, 1x)	Myocardial infarction	Observational analysis of RCT, prospective cohort	Hospital discharge to 12 months

Navarese 2013 (90)	Early intervention for NSTE-ACS	Major bleeding	3	21,147	916	Male and female population with non-ST elevation ACS / undergoing PCI, CABG or medical treatment / with or without: positive biomarkers, metabolic disease, ST-segment depression, medication with glycoprotein IIb/IIIa inhibitors	62.5-67.5 (mean)	Early intervention for NSTE-ACS / time to intervention ≤24 hours or <24 hours	Delayed intervention / time to intervention >24 hours (2x); >48 hours (1x)	Major bleeding	Observational analysis of RCT, prospective cohort	30 days to 12 months
Nelson 2010 (36)	Caesarean section	Anal incontinence, feces	11	16,832	NR	Pregnant women / primipara only or all pregnancies / reported excluding women with prepartum incontinence (1x)	42.7 (mean for 1x)/ NR	Caesarean section	Vaginal delivery	Fecal incontinence	Prospective cohort, cohort study	2 weeks to 12 years (range)
Nelson 2010 (36)	Caesarean section	Anal incontinence, flatus	4	5,594	NR	Pregnant women / primipara only or all pregnancies / reported excluding women with prepartum incontinence (1x)	NR	Caesarean section	Vaginal delivery	Incontinence of flatus	Prospective cohort, cohort study	3 months -10 years
Nieuwenhuijse 2014 (37)	Ceramic-on-ceramic bearings for total hip arthroplasty	Harris Hip Score	3	403	NR	Male and female population / mostly with osteoarthritis / population including all patients (1x), only <70 years (1x) or with high activity level only (1x)	52.5-68.3 (mean)/ 37-79 (range)	Ceramic-on-ceramic bearings for total hip arthroplasty	Metal-on-polyethylene or ceramic-on-polyethylene bearings for total hip arthroplasty	Harris-Hip Score at short-,mid-, or long-term	Retrospective cohort	3.3-10.9 years (mean)/ 2-12 years (range)
Nieuwenhuijse 2014 (37)	High-flexion total knee arthroplasty	Flexion	26	3,079	NR	Male and female population / mostly with osteoarthritis / mostly all patients regardless of age or activity status	63.9-72.9 (mean)	High flexion prosthesis for total knee arthroplasty	Standard prosthesis for total knee arthroplasty	Flexion in degrees at short- or mid-term	Retrospective cohort, cohort with historic controls	0-5.7 years (mean follow-up)
Nieuwenhuijse 2014 (37)	Gender-specific total knee arthroplasty	Flexion-extension range	2	274	NR	Female (49.6-100%) and male population / mostly with osteoarthritis / without: cancer (1x) fracture (1x)	64.8-68.1 (mean)	Gender-specific prosthesis for total knee arthroplasty	Non-gender specific prosthesis for total knee arthroplasty	Flexion-extension range of motion, short-term	Retrospective cohort	0.5-1 year (mean)

Nikooie 2019 (55)	Second generation antipsychotics	Sedation	3	162	16	Male and female population with delirium / health status: all inpatients with delirium (1x), with cancer (1x), with physical diseases (1x)	64-71 (mean)	Second generation antipsychotics / drugs: Olanzapine 4.9-8.2mg/day; Quetiapine 26.7mg/day; Risperidone 0.6mg/day (mean doses)	Haloperidol / 0.9-4.9mg/day (mean); 1.5-10mg/day (range)	Excessively sedated, sedation	Prospective cohort, retrospective cohort	NR
Nikooie 2019 (55)	Second generation antipsychotics	Neurologic outcomes	5	2,679	138	Male and female population with delirium / health status: all inpatients with delirium (2x), with cancer (2x), with physicial diseases (1x)	62-74 (mean)	Second generation antipsychotics / drugs, doses (reported as mean or as maximum dose): Risperidone 0.6-1mg/day, max. 1.4mg/day; Olanzapine 3.5-8.2mg/day, max. 10.2mg/day; Aripiprazole 15.2 mg/day, max. 7.2mg/day; Quetiapine 26.7 mg/day, max 71.8mg/day	Haloperidol / 0.9-4.6mg/day (mean); 1.5-10mg/day (range)	Dystonia, Extrapyramidal symptoms	Prospective cohort, retrospective cohort	NR
Ochen 2019 (91)	Surgery for achilles tendon rupture	Re-rupture	18	14,847	397	Mostly male (~74%) young adult and middle-aged population with acute achilles tendon rupture / elderly population only (1x)	29.7-72 (mean)/ 11-85 (range)	Surgical Achilles tendon repair / open or minimalinvase surgery	Nonoperative treatment / cast, brace or orthosis for 6-13 weeks; mostly without early full-weightbearing; partly with early functional rehabilitation	Re-rupture	Prospective cohort, observational analysis of RCT, retrospective cohort, cohort with historic controls	12-95 months (mean)
Ochen 2019 (91)	Surgery for achilles tendon rupture	Complications	15	14,559	429	Mostly male (~74%) young adult and middle-aged population with acute achilles tendon rupture / elderly population only (1x)	34.8-72 (mean)/ 11-85 (range)	Surgical Achilles tendon repair / open or minimalinvase surgery	Nonoperative treatment / cast, brace or orthosis for 6-13 weeks; mostly without early full-weightbearing; partly with early functional rehabilitation	Complications (Infections, Thrombosis and others)	Prospective cohort, observational analysis of RCT, retrospective cohort, cohort with historic controls	12-95 months (mean)
Pittas 2010 (60)	Vitamin D	Hypertension	3	2,553	>407	Generally healthy male and female population (from the health professionals follow-up study (USA) and the nurses health study (USA))	43-65 (mean)/ 30-75 (range)	High vitamin D status (>75 or 81 nmol/L)	Low vitamin D status (<37-51 nmol/L)	Incident hypertension; validated self-report	Prospective cohort, nested case-control	7-8 years (mean)

Raman 2013 (38)	Carotid endarterectomy	Ipsilateral stroke	2	356	25	Male and female population with asymptomatic carotid artery stenosis / including patients with contralateral symptomatic lesions / with or without: metabolic disease, hypertension, smokers and non-smokers	60-69.3 (mean)	Carotid endarterectomy plus medical therapy (not further specified)	Medical therapy (not further specified)	Ipsilateral stroke (including any stroke within 30d)	Retrospective cohort	2-3.8 years (mean)
Raman 2013 (38)	Carotid endarterectomy	Stroke	3	490	52	Male and female population with asymptomatic carotid artery stenosis / including patients with contralateral symptomatic lesions / with or without: high-grade stenosis, metabolic disease, hypertension, smokers and non-smokers	68-70 (mean)	Carotid endarterectomy plus medical therapy (not further specified)	Medical therapy (not further specified)	Any stroke (including any death within 30 days)	Retrospective cohort	3-5 years (mean)
Raman 2013 (38)	Carotid artery stenting	Periprocedural stroke	5	372,257	3,527	Male and female population with asymptomatic carotid artery stenosis / broad population from administrative datasets or registries / with or without: CAD, hypertension, metabolic disease, smokers and non-smokers	69.8-72 (mean)	Carotid artery stenting	Carotid endarterectomy	Any periprocedural stroke	Retrospective cohort, retrospective database cohort	<30 days
Schweizer 2013 (39)	Nasal deconolization	Surgical site infection	6	20,171	137	Male and female population at orthopedic or cardiac surgery wards	NR	Nasal Mupirocin / dose: 2% or not specified (2x) / start: for 3-5 days preoperatively (4x) / treatment for all patients regardless of MRSA/ MSSA carrier status (4x) or for MRSA/ MSSA carriers only (2x)	No use of nasal Mupirocin	Wound infections, mostly assessed by Center for Disease Control criteria	Prospective cohort, retrospective cohort	NR
Schweizer 2013 (39)	Glycopeptide prophylaxis	Surgical site infection	7	27,971	481	Male and female population at orthopedic or cardiac surgery wards	NR	Prophylactic treatment with Vancomycin or Teicoplanin (1x) / alone or with Rifampicin, Cephalosporine or non-specified antimicrobial agent	Prophlyactic antibiotic treatment / mostly Cephalosporins or other Beta-lactam antibiotics; Vancomyin (1x)	Wound infections, mostly assessed by Center for Disease Control criteria	Prospective cohort, retrospective cohort, retrospective cohort with historical controls	60 days, mostly not specified

Silvain 2012 (40)	Enoxaparin	All-cause mortality	7	11,074	476	Mostly male population, with: acute CHD/ MI and undergoing urgent PCI / without: cardiogenic shock (2x), population with MI post-fibrinolysis (1x), previous thrombolysis or anticoagulation (2x) / with or without: metabolic disease	NR	Enoxaparin / IV or SC / dosing: single bolus (0.5-1mg/kg or 30 mg) or repeated administration (every 12h) / partly additional dose of unfractionated Heparin or Enoxaparin during PCI / additional anticoagulation for most patients of both groups (e.g. Aspirin, Clopidogrel)	Unfractionated Heparin / IV / dosing: 60-70 IU/kg or 5,000 IU bolus / with subsequent continued administration during PCI with 12 IU/kg/h or additional bolus with 50-70 IU/kg / treatment control: with or without titration to activated clotting time (200-300s) or not reported	All-cause mortality	Prospective cohort, obervational analysis of RCT, cohort	In-hospital to 15 months
Silvain 2012 (40)	Enoxaparin	Major bleeding	7	11,376	388	Mostly male population, with: acute CHD/ MI (6x) and undergoing urgent PCI/ partly: PCI after fibrinolysis (1x), elective PCI (1x), high risk MI (1x), without cardiogenic shock (1x), prior anticoagulation or thrombolysis (2x) / with or without: metabolic disease	NR	Enoxaparin / IV or SC / dosing: single bolus (0.5-1mg/kg or 30 mg) or repeated administration (every 12h) / partly additional dose of unfractionated Heparin or Enoxaparin during PCI / additional anticoagulation for most patients of both groups (e.g. Aspirin, Clopidogrel)	Unfractionated Heparin / IV / dosing: 60-70IU/kg or 5,000 IU bolus / subsequent continued administration or additional bolus during PCI (12 IU/kg/h or 50-70 UI/kg bolus) / treatment control: with or without titration to activated clotting time 200-300s or not reported	Major bleeding (TIMI or individual study definition)	Prospective cohort, obervational analysis of RCT, cohort	In-hospital to 15 months
Silvain 2012 (40)	Enoxaparin	All-cause mortality or myocardial infarction	7	8,707	543	Mostly male population, with CHD and undergoing PCI / mostly urgent PCI for MI (6x) / partly: elective PCI (1x), without: cardiogenic shock (1x), prior thrombolysis or anticoagulation (1x) / with or without: metabolic disease	NR	Enoxaparin / IV or SC / dosing: single bolus (0.5-1mg/kg) or repeated administration (every 12h) / partly additional dose of unfractionated Heparin or Enoxaparin during PCI / additional anticoagulation for most patients of both groups (e.g. Aspirin, Clopidogrel)	Unfractionated Heparin / IV / dosing: 60-70IU/kg or 5,000 IU bolus / subsequent continued administration or additional bolus during PCI (12 IU/kg/h or 50-70 UI/kg bolus) / treatment control: with or without titration to activated clotting time 200-300s or not reported	Death or Myocardial infarction	Prospective cohort, cohort study	In-hospital to 15 months
Suthar 2012 (26)	Antiretroviral therapy	Tuberculosis infection	9	21,348	866	Male and female population / with or without advanced HIV infection (7x), CD4 count <200 to >500 cells/µL / partly: without advanced HIV infection (1x), patients with signs of active tuberculosis infection (1x) / setting: developing countries	≥15	ART with ≥3 antiretroviral drugs / ART: not specified or consisting of two nucleoside reverse transcriptase inhibitors plus either a non-nucleoside reverse transcriptase inhibitor or a protease inhibitor	Population not receiving ART	Tuberculosis infection / various defintions, mostly as a combination of the following items: clinical signs and response to therapy, radiological, microbiological, histological, autopsy confirmed	Prospective cohort, retrospective cohort, observational analysis of RCT	4.75-22.8 months (median); 13-60 months (range)

Te Morenga 2013 (61)	Sugar	Weight gain	4	149,305	NR	Mostly female generally healthy population / younger (university graduates, nurses) and older adults / without: diabetes (3x), severe diseases (3x) (cancer, heart disease)	21-74 (range)	High intake of sugar sweetened beverages (including sugar sweetened soft drinks, fruit juice and others e.g. iced tea)	Low intake of sugar sweetened beverages	Weight gain	Prospective cohort	4-6 years (range)
Te Morenga 2013 (61)	Sugar	Body Mass Index	4	>4,094	NR	Male and female children and adolescents / general population / without underweight (BMI < 5th percentile) (1x)	2-14 (range)	Risk by one serving/day or 100g/day intake increase of sugar sweetened beverages (soft drinks, fruit juice) / intake assessment by food frequency questionaire, 3 day diet record (1x)	NA (see intervention)	Change in BMI	Prospective cohort, prospective cohort nested in RCT	1-10 years (range)
Thomas 2010 (92)	Influenza vaccines	Influenza-like illness	1	12,742	1,442	Elderly population / institutionalised in two types of long-term nursing care facilities (requiring standard care or advanced care) / age and gender not reported / 0 to >80% of residents with influenza vaccination / setting: Japan	NR	Number of health care workers vaccinated per facility ≥10	Number of health care workers vaccinated per facility <10	Influenza-like illness in residents (not further specified)	Prospective cohort	3 months
Tickell-Painter 2017 (93)	Mefloquine	Discontinuation due to adverse effects	9	7,785	934	Generally healthy adults / short-term travellers or longer stay (soldiers, volunteers) / further specifications: soldiers in active service (2x), travellers from Japan, Peace Corps volunteers (2x), healthy adult travellers (without severe underlying disease) (1x), medical students with stay abroad (1x), short-term travellers from USA (1x)	18-65 (range)	Mefloquine / dose 250mg/week or not specified / duration: minimum 1 week before to 4 weeks after travelling	Atovaquone-proguanil / dose and duration mostly not specified (8x) / partly: 250mg Atovaquone+100mg Proguanil daily for 2 days before to 1 week after travel (1x)	Discontinuation of study drug due to adverse effects	Prospective cohort, retrospective cohort, cohort	Reported as duration of exposure to malaria: 10-224 days (range), 2 weeks-6 months (mean)

Tickell-Painter 2017 (93)	Mefloquine	Serious adverse events or effects	2	1,167	7	Travellers / general population / without: known adverse reactions to study drug or adverse reaction in the first week, pregnancy	>18/ NR	Mefloquine / dose and duration: 250mg/week for 1 week before to 4 weeks post travel (1x); not specified (1x)	Non-users of antimalarials (travellers to countries with or without malaria or not specified)	Serious adverse events	Retrospective cohort	Reported as: duration of exposure to malaria: 1 to 9 weeks (1x) (range)/ monitoring until 20 weeks post-travel (1x)
Tickell-Painter 2017 (93)	Mefloquine	Nausea	3	1,901	301	Travellers / general population / without: known adverse reactions to study drug, serious adverse reaction to Mefloquine in the first week, pregnancy	>18/ NR	Mefloquine / dose: 250mg/week (2x) / duration: 1 week before to 4 weeks post-travel (1x)	Non-users of antimalarials (travellers to countries with or without malaria without use of study drug or not specified)	Nausea	Prospective cohort, retrospective cohort	Reported as: duration of exposure to malaria: 1 to 9 weeks (1x) (range)/ monitoring until 20 weeks post-travel (1x)
Tricco 2018 (45)	Live-attenuated zoster vaccines	Suspected Herpes Zoster	3	1,934,183	55,228	Male and female population / immunocompetent (1x) immunocompromised (1x) or mixed immunocompetent and immunocompromised (1x)	67.7-74.0 (mean)	Live zoster vaccine (Zostavax) / mostly single dose	No vaccination	Suspected herpes zoster	Retrospective cohort, cohort	36-102 months
Vinceti 2018 (59)	Selenium	Cancer	7	76,239	1,940	General population / health status: no history of cancer (2x) or community-dwelling (1x) or not specified	15-74 (range)	High selenium (serum and plasma status)	Low selenium (serum and plasma status)	Any cancer; stomach, rectal, lung, colon and bladder cancer	Cohort-subcohort controlled study, nested case-control	5-20 years
Vinceti 2018 (59)	Selenium	Cancer mortality	7	183,863	3,869	General population / health status: no history of cancer (2x), adult haemodialysis patients (1x) or not specified	5-90 (range)	High selenium (selenium intake, serum and plasma status)	Low selenium (selenium intake, serum and plasma status)	Mortality from any cancer; malignant disease-related death, cancer deaths, cancer mortality	Cohort, cohort-subcohort controlled study, nested case-control	4-14 years
Vinceti 2018 (59)	Selenium	Colorectal cancer	6	712,746	2,627	General population / mostly without history of cancer	25-70 (range)	High selenium (serum or toenail status, selenium supplement)	Low selenium (serum or toenail status, selenium supplement)	Colorectal cancer	Cohort study, cohort-subcohort controlled study, nested case-control	2-20 years

Wilson 2011 (41)	Traditional birth attendants	Perinatal mortality	1	1,028	51	Women giving birth in rural low-income setting in Brazil	20-34 (range for 69.2%)	Birth assissted by traditional birth attendant / experience: trained with five one-hour meetings, practical experience at mini-maternity unit	Birth not assissted by traditional birth attendant (assistance by neighbour or relative)	Perinatal mortality	Prospective cohort	NR
Wilson 2011 (41)	Traditional birth attendants	Neonatal mortality	2	4,032	63	Women giving birth in rural low-income setting in Brazil, Mozambique	26.6-27.3 (mean)/ 20-34 (range for 69.2%)	Birth assissted by traditional birth attendant or access to trained traditional birth attendant / experience: trained with five one-hour meetings, practical experience at mini-maternity unit (1x); three-week training program (1x)	Birth not assissted by traditional birth attendant (assistance by neighbour or relative/ no access to traditional birth attendant)	Neonatal mortality	Prospective cohort, retrospective cohort	NR
Wilson 2019 (42)	Unicompartimental knee arthroplasty	Venous thromboembolism	8	261,684	2,536	Male and female adults mostly with osteoarthritis / any health status but sparse information / mean BMI: 27.7-32.6 / mostly primary arthroplasty only / partly: without fractures and emergency procedures, intact anterior cruciate ligament only (1x)	63.96-79.7 (mean)/47-86 (range)	Unicompartimental knee arthroplasty	Total knee arthroplasty	Venous thromboembolism	Retrospective database cohort, retrospective cohort, cohort	30 days to 5 years
Wilson 2019 (42)	Unicompartimental knee arthroplasty	Flexion-extension range	11	3,891	NR	Male and female adults mostly with osteoarthritis / any health status but sparse information / mean BMI: 24.1-40.5 / all arthroplasties or primary arthroplasties only / partly: intact anterior cruciate ligament only, only obese population BMI > 35 (1x), elderly adults >70 only (1x)	59.7-80.1 (mean)/ 38-93 (range)	Unicompartimental knee arthroplasty	Total knee arthroplasty	Range of movement in degrees	Retrospective cohort, cohort	2-10 years

Wilson 2019 (42)	Unicompartimental knee arthroplasty	Operation duration	8	56,349	NR	Male and female adults / any health status but sparse information / mean BMI: 29.5-40.5 / mostly with primary knee arthroplasty only / elderly only (>70/>65) (2x)	59.7-80.1 (mean)	Unicompartimental knee arthroplasty	Total knee arthroplasty	Operation duration	Retrospective cohort, retrospective database cohort, cohort	30 days to 30 months
Yank 2011 (44)	Recombinant factor VII	All-cause mortality	2	182	19	Male and female population with refractory bleeding after cardiac surgery	56-73.2 (mean)	Recombinant factor VIIa / dose: 18-51.1 µg/kg (mean) / administered after surgery for refractory bleeding	No recombinant factor VIIa use	In-hospital mortality	Retrospective cohort	NR
Yank 2011 (44)	Recombinant factor VII	Thromboembolism	2	182	15	Male and female population with refractory bleeding after cardiac surgery	56-73.2 (mean)	Recombinant factor VIIa / dose: 18-51.1 µg/kg (mean) / administered after surgery for refractory bleeding	No recombinant factor VIIa use	Thromboembolic events	Retrospective cohort	NR
Zhang 2016 (94)	Everolimus-eluting bioresorbable vascular scaffold	Stent thrombosis	3	2,767	30	Male (>67%) and female population / mixed population with either MI or angina pectoris / with or without: metabolic disease, hypertension, smokers or non-smokers / setting: "worldwide"	54-62.2 (mean)	Coronary stenting with Everolimus-eluting bioresorbable vascular scaffold	Coronary stenting with Everolimus-eluting metallic stents	Stent thrombosis (definite or probable); not further specified	Retrospective cohort, cohort	6-12 months
Zhang 2016 (94)	Everolimus-eluting bioresorbable vascular scaffold	All-cause mortality	4	1,580	26	Male (>67%) and female population / mixed population with either MI or angina pectoris / with or without: metabolic disease, hypertension, smokers or non-smokers / setting: "worldwide"	54-64.2 (mean)	Coronary stenting with Everolimus-eluting bioresorbable vascular scaffold	Coronary stenting with Everolimus-eluting metallic stents	All-cause death	Retrospective cohort, cohort	6-12 months
Zhang 2016 (94)	Everolimus-eluting bioresorbable vascular scaffold	Coronary heart disease mortality	4	2,951	31	Male (>67%) and female population / mixed population with either MI or angina pectoris / with or without: metabolic disease, hypertension, smokers or non-smokers / setting: "worldwide"	54-64.2 (mean)	Coronary stenting with Everolimus-eluting bioresorbable vascular scaffold	Coronary stenting with Everolimus-eluting metallic stents	Cardiac death	Retrospective cohort, cohort	6-12 months

Zhang 2017 (95)	Percutaneous coronary intervention	All-cause mortality	17	16,467	NR	Male (65-82.5%) and female population with CHD / with or without: acute MI, metabolic disease and hypertension, prior MI 6.9-38.1%, current smokers: 17-53.6%	60.8-78 (mean)/ 75-88 (range)	Percutaneous coronary intervention / stent types: mostly bare metal stent or early-generation drug eluting stent; partly: second-generation drug eluting stent (1x)	Coronary artery bypass graft surgery	All-cause mortality	Prospective cohort, retrospective cohort	1-9.7 years (mean)
Zhang 2017 (95)	Percutaneous coronary intervention	Cardiovascular mortality	5	6,605	NR	Male (76.3-82.5%) and female population with CHD / without: acute MI (4x) / with or without: metabolic disease, hypertension, prior MI 16-38.1%, current smokers: 25-53.6%	62.2-69.4 (mean)	Percutaneous coronary intervention / stent types: mostly bare metal stent or early-generation drug eluting stent	Coronary artery bypass graft surgery	Cardiovascular mortality	Prospective cohort, retrospective cohort	1-7.1 years (median/ mean)
Zhang 2017 (95)	Percutaneous coronary intervention	Myocardial infarction	5	6,637	NR	Male (76.3-82.5%) and female population with CHD / without: acute MI (3x) / with or without: metabolic disease, hypertension, prior MI 20.1-38.1%, current smokers: 6.9-53.6%	60.8-69.4 (mean)	Percutaneous coronary intervention / stent types: mostly bare metal stent or early-generation drug eluting stent	Coronary artery bypass graft surgery	Myocardial infarction	Prospective cohort, retrospective cohort	3-7.1 years (median/ mean)
Ziff 2015 (96)	Digoxin	All-cause mortality	8	34,008	NR	Survivors of MI (6x), or population with ACS (1x) / with or without: heart failure, atrial fibrillation, metabolic disease	53-75 (mean)	Digoxin	No digoxin use	All-cause mortality	Prospective cohort, observational analysis of RCT, retrospective cohort	1-12 years
Ziff 2015 (96)	Digoxin	Cardiovascular mortality	3	11,399	NR	Survivors of MI with or without heart failure (2x) and atrial fibrillation / elderly without heart failure and in sinus rythm (1x)	53-75 (mean)	Digoxin	No digoxin use	Cardiovascular mortality	Prospective cohort, observational analysis of RCT	2-12 years (mean follow-up)
Ziff 2015 (96)	Digoxin	Hospital admission	4	6,584	NR	Mostly population with acute or chronic heart failure / population with or without: atrial fibrillation	63-76 (mean)	Digoxin	No digoxin use	All-cause hospital admission	Prospective cohort, observational analysis of RCT, retrospective cohort	1-8.2 years (mean follow-up)

ACS= acute coronary syndrome; AIDS= acquired immune deficiency syndrome; ART= antiretroviral therapy; ASA= american society of anesthesiologists; BCG= bacillus calmette-guérin; BMI= body mass index; CABG= coronary artery bypass graft; CAD= coronary artery disease; CD4= cluster of differentiation 4; CHD= coronary heart disease; COPD= chronic obstructive pulmonary disease; CVD= cardiovascular disease; DDP-4= dipeptidyl peptidase 4; DTP= diphteria, tetanus, pertussis; eGFR= estimated glomerular filtration rate; GDM= gestational diabetes mellitus; HbA1c= hemoglobin A1c; HIV= human immunodeficiency virus; HSV-2= herpes simplex virus type 2; ICU= intensive care unit; MI= myocardial infarction; MRI= magnetic resonance imaging; MRSA= methicillin resistant staphylococcus aureus; MSSA= methicillin sensitive staphylococcus aureus; NA= not applicable; NOS= newcastle-ottawa scale; NR= not reported; NSAID= non-steroidal anti-inflammatory drug; NSTE-ACS= non-ST elevation acute coronary syndrome; NYHA= new york heart association (stage); PCI= percutaneous coronary intervention; PPV= pneumococcal polysaccharide vaccine; SGLT-2= sodium glucose transporter 2; SPMSQ= short portable mental status questionnaire; STAT= instantly; STEMI= ST elevation myocardial infarction; µL= microliter WHO= world health organization // units: g= gram; IU= international units; kcal= kilocalorie; kg= kilogram; L= liter; mg= milligram; mL= milliliter; mmol= millimol; µg= microgram; µL= microliter // application routes: IM= intramuscular; IV= intravenous; P.O.= per os; SC= subcutaneous.

Table S7. Risk of bias and certainty of the evidence for included bodies of evidence from cohort studies

**Reference/ year**	**Intervention/ Exposure**	**Outcome**	**Certainty / strength of the evidence***	risk of bias **tool**	**Study quality/ low** risk of bias **(reported as number of low risk of bias studies per domain)**
Abou-Setta 2011 (74)	Nerve block	Delirium	Moderate (RCTs and cohort studies)	NOS	NOS: 8
Abou-Setta 2011 (74)	Spinal anesthesia	All-cause mortality	Low (RCTs and cohort studies)	NOS	NOS: 6.6
Aburto 2013 (75)	Low sodium	All-cause mortality	Very low	Cochrane tool, modified	Selection of participants (6/7), Blinding of participants and personnel (0/7), Blinding of outcome assessment (2/7), Incomplete outcome data (6/7), Selective reporting (7/7), Defining exposure (5/7), Other confounding (5/7)
Aburto 2013 (75)	Low sodium	Cardiovascular disease	Very low	Cochrane tool, modified	Selection of participants (6/9), Blinding of participants and personnel (1/9), Blinding of outcome assessment (4/9), Incomplete outcome data (8/9), Selective reporting (9/9), Defining exposure (6/9), Other confounding (6/9)
Ahmad 2015 (27)	Intra-aortic balloon pump	All-cause mortality	NR	Individual tool	Attempt to control for confounders (6/14)
Alexander 2017 (76)	DHA and EPA	Coronary heart disease	NR	NOS	NOS: 7.6
Alexander 2017 (76)	DHA and EPA	Coronary heart disease mortality	NR	NOS	NOS: 7.4
Alexander 2017 (76)	DHA and EPA	Coronary heart disease incidence	NR	NOS	NOS: 7.5

Alipanah 2018 (24)	Self-administered therapy	Low treatment success	NR	NOS	NOS: 6.8
Alipanah 2018 (24)	Self-administered therapy	Low treatment completion	NR	NOS	NOS: 6.9
Alipanah 2018 (24)	Self-administered therapy	All-cause mortality	NR	NOS	NOS: 6.9
Anglemyer 2013 (77)	Antiretroviral therapy	HIV infection	Moderate	NOS (and Cochrane)	NOS: 6.9 (cochrane: Random sequence generation (0/9); allocation concealment (0/9); blinding (performance bias and detection bias) (0/9); incomplete outcome data (3/9); selective reporting (6/9); other bias (7/9))
Azad 2017 (21)	Nonnutritive sweeteners	Body Mass Index	NR	NOS	NOS: 7
Barnard 2015 (28)	Surgical abortion by mid-level providers	Failure or incomplete abortion	Very low (before exclusion of one study)	Cochrane	Selection bias (0/2), allocation concealment (0/2), blinding of participants and personnel (2/2), blinding of outcome assessment (0/2), incomplete outcome data (0/2), selective reporting (0/2), other bias (0/2)
Barnard 2015 (28)	Surgical abortion by mid-level providers	Complications	Very low (before exclusion of one study)	Cochrane	Selection bias (0/2), allocation concealment (0/2), blinding of participants and personnel (2/2), blinding of outcome assessment (0/2), incomplete outcome data (1/2), selective reporting (1/2), other bias (1/2)
Barnard 2015 (28)	Surgical abortion by mid-level providers	Abortion failure and complications	Very low (before exclusion of one study)	Cochrane	Selection bias (0/3), allocation concealment (0/3), blinding of participants and personnel (3/3), blinding of outcome assessment (0/3), incomplete outcome data (2/3), selective reporting (1/3), other bias (2/3)
Bellemain-Appaix 2012 (48)	Clopidogrel	All-cause mortality	NR	NOS	NOS: 8.6
Bellemain-Appaix 2012 (48)	Clopidogrel	Major bleeding	NR	NOS	NOS: 8.6

Bellemain-Appaix 2012 (48)	Clopidogrel	Coronary heart disease	NR	NOS	NOS: 8.6
Bellemain-Appaix 2014 (47)	P2Y12 inhibitors	All-cause mortality	NR	NOS	NOS: 8.5
Bellemain-Appaix 2014 (47)	P2Y12 inhibitors	Major bleeding	NR	NOS	NOS: 8.5
Bellemain-Appaix 2014 (47)	P2Y12 inhibitors	Main composite ischemic endpoint	NR	NOS	NOS: 8.5
Bloomfield 2016 (22)	Mediterranean diet	Breast cancer	Low (RCTs and cohort studies)	Individual tool	Population (5/13), outcomes (10/13), measurement (1/13), confounding: (9/13)/ overall: low risk of bias: (3/13)
Bolland 2015 (49)	Calcium	All fractures	NR	NA	NR
Bolland 2015 (49)	Calcium	Vertebral fracture	NR	NA	NR
Bolland 2015 (49)	Calcium	Hip fracture	NR	NA	NR
Brenner 2014 (29)	Sigmoidoscopy	Colorectal cancer mortality	NR	Individual tool	9/9 (Mean number of quality criteria met)
Brenner 2014 (29)	Sigmoidoscopy	Colorectal cancer incidence	NR	Individual tool	6.5/9 (Mean number of quality criteria met)

Chowdhury 2012 (78)	Omega-3	Cerebrovascular disease	NR	Individual tool based on MOOSE, QUATSO, and STROBE guidelines	Modified quality score (for RCTs and cohort studies) (0-6, 6 is best): average: 5.9
Chowdhury 2014a (79)	α-linolenic acid	Coronary heart disease	NR	NOS	NOS: 7.7
Chowdhury 2014a (79)	Omega-3	Coronary heart disease	NR	NOS	NOS: 8.2
Chowdhury 2014a (79)	Omega-6	Coronary heart disease	NR	NOS	NOS: 7.9
Chowdhury 2014b (80)	Vitamin D	All-cause mortality	NR	NOS	NOS: 7.3
Chung 2011 (58)	Vitamin D	Colorectal cancer	NR	AHRQ/ STROBE	Study quality rated as good, fair, or poor: fair (8/9), poor (1/9)
Chung 2011 (58)	Vitamin D	Breast cancer	NR	AHRQ/ STROBE	Study methodological quality rated as good, fair, or poor: fair (3/4), poor (1/4)
Chung 2016 (56)	Calcium	Cardiovascular mortality	NR	AHRQ/ STROBE	Percentage of studies with low risk of bias: sampling scheme described (70%), exposure assessors blinded to outcome status (100%), outcome assessors blinded to exposure measurement (0%), food composition database or supplemental composition reported (70%), internal calibration for food frequency questionnaires(100%), justification of final adjusted model selection was reported (70%), clear definition of outcomes (100%), <20% loss to follow-up (100%), a primary outcome is specified (50%)
Ding 2017 (81)	Dairy	Systolic blood pressure	NR	NA	NR

Fenton 2018 (30)	Radiation therapy	Erectile dysfunction	Moderate (RCTs and cohort studies)	AHRQ/ USPSTF (supplemented by NOS)	Study quality rated as good, fair, or poor: good: (2/7), fair (5/7)
Fenton 2018 (30)	Radical Prostatectomy	Urinary incontinence	Moderate (RCTs and cohort studies)	AHRQ/ USPSTF (supplemented by NOS)	Study quality rated as good, fair, or poor: good (2/5), fair (3/5)
Fenton 2018 (30)	Radical Prostatectomy	Erectile dysfunction	Moderate (RCTs and cohort studies)	AHRQ/ USPSTF (supplemented by NOS)	Study quality rated as good, fair, or poor: good (2/6), fair (4/6)
Filippini 2017 (43)	Disease-modifying drugs	Conversion to clinically definite multiple sclerosis	NR	ROBINS-I	Confounding (0/2), selection of participants into the study (1/2), classification of interventions (1/2), deviations from intended interventions (0/2), missing data (0/2), measurement of outcomes (0/2), selection of the reported result (2/2)/ overall: low risk of bias (0/2)
Fluri 2010 (31)	Extracranial-intracranial arterial bypass	All-cause mortality	NR	Individual tool	Adequate allocation concealment (10/11)
Fluri 2010 (31)	Extracranial-intracranial arterial bypass	Stroke	NR	Individual tool	Adequate allocation concealment (13/15)
Fluri 2010 (31)	Extracranial-intracranial arterial bypass	Stroke mortality or dependency	NR	Individual tool	Adequate allocation concealment (7/8)
Gargiulo 2016 (32)	Transcatheter aortic valve	Early all-cause mortality	NR	NOS	NOS: 8.6; NR (1x)
Gargiulo 2016 (32)	Transcatheter aortic valve	Mid-term all-cause mortality	NR	NOS	NOS: 8.4
Gargiulo 2016 (32)	Transcatheter aortic valve	Long-term all-cause mortality	NR	NOS	NOS 8.6; NR (1x)

Hartling 2013 (50)	Treating gestational diabetes mellitus	High birth weight	Low	NOS	NOS: 8
Hartling 2013 (50)	Treating gestational diabetes mellitus	Large-for-gestational age neonate	NR	NOS	NOS: 8.3
Hartling 2013 (50)	Treating gestational diabetes mellitus	Shoulder dystocia	Low	NOS	NOS: 8
Henderson 2019 (51)	Treating asymptomatic bacteriuria	Pyelonephritis	NR	USPSTF	Study quality rated as good, fair, or poor: fair quality (2/2)
Higgins 2016 (25)	Bacillus Calmette-Guérin	All-cause mortality	NR	ROBINS-I	Selection of participants into the study (0/8), confounding (0/8), classification of vaccination status (0/8), deviations from intended interventions (0/8), measurement of outcomes (8/8), missing outcome data (0/8), selection of the reported result (0/8)/ overall: low risk of bias (0/8)
Higgins 2016 (25)	Measles containing vaccines	All-cause mortality	NR	ROBINS-I	Selection of participants into the study (1/13), confounding (0/13), classification of vaccination status (0/13), deviations from intended interventions (0/13), measurement of outcomes (13/13), missing outcome data (0/13), selection of the reported result (0/13)/ overall: low risk of bias (0/13)
Hopley 2010 (33)	Total hip arthroplasty	Reoperation	NR	Special criteria for study topic according to Parker et al.	Average number of quality criteria from rating scale met: 5
Hopley 2010 (33)	Total hip arthroplasty	Dislocation	NR	Special criteria for study topic according to Parker et al.	Average number of quality criteria from rating scale met: 5
Hopley 2010 (33)	Total hip arthroplasty	Deep infection	NR	Special tool for study topic	Average number of quality criteria from rating scale met: 4.8
Hüpfl 2010 (67)	Chest-compression-only cardiopulmonary resuscitation	All-cause mortality	NR	NA	NR

Jamal 2013 (82)	Non-calcium-based phosphat binders	All-cause mortality	NR	NA	NR
Jefferson 2010 (46)	Parenteral influenza vaccine	Influenza-like illness	NR	NOS, not convertable	Adequate allocation concealment (7/30)/ overall quality (assessed by Newcastle Ottawa scale, high quality defined as maximum 1 inadequate item): Quality A (8/26), rating appears in sensitivity analysis, only for cohort studies in nursing homes
Jefferson 2010 (46)	Parenteral influenza vaccine	Influenza	NR	NOS, not convertable	Adequate allocation concealment (3/10)
Jefferson 2012 (34)	Inactivated influenza vaccines	Influenza	NR	NOS	Selection of exposed cohort (0/1), selection of non-exposed cohort (0/1), comparability (0/1), assessment of outcome summary assessments (0/1)
Jefferson 2012 (34)	Inactivated influenza vaccines	Influenza-like illness	NR	NOS	Selection of exposed cohort (0/2), selection of non-exposed cohort (0/2), comparability (0/2), assessment of outcome (1/2), summary assessments (0/2)
Jin 2012 (83)	Total flavonoids	Colorectal neoplasms	NR	NOS	Modified NOS: average 13 from maximum 16 points
Johnston 2019 (23)	Low red meat	All-cause mortality	Very low	CLARITY, modified	Number of studies with low risk of bias: 1. Was selection of exposed and non-exposed cohorts drawn from the same population? (22/30), 2. Can we be confident in the assessment of exposure? (4/30), 3. Can we be confident that the outcome of interest was not present at start of study? (30/30), 4. Did the study match exposed and unexposed for all variables that are associated with the outcome of interest or did the statistical analysis adjust for these prognostic variables? (20/30), 5. Can we be confident in the assessment of the presence or absence of prognostic factors? (22/30), 6. Can we confident in the assessment of outcome? (28/30), 7. Was the follow-up of cohorts adequate? (13/30)
Johnston 2019 (23)	Low red meat	Cardiovascular mortality	Very low	CLARITY, modified	Number of studies with low risk of bias: 1. Was selection of exposed and non-exposed cohorts drawn from the same population? (21/28), 2. Can we be confident in the assessment of exposure? (5/28), 3. Can we be confident that the outcome of interest was not present at start of study? (24/28), 4. Did the study match exposed and unexposed for all variables that are associated with the outcome of interest or did the statistical analysis adjust for these prognostic variables? (3/28), 5. Can we be confident in the assessment of the presence or absence of prognostic factors? (24/28), 6. Can we confident in the assessment of outcome? (26/28), 7. Was the follow-up of cohorts adequate? (12/28)

Johnston 2019 (23)	Low red meat	Cardiovascular disease	Low	CLARITY, modified	Number of studies with low risk of bias: 1. Was selection of exposed and non-exposed cohorts drawn from the same population? (15/16), 2. Can we be confident in the assessment of exposure? (5/16), 3. Can we be confident that the outcome of interest was not present at start of study? (5/16), 4. Did the study match exposed and unexposed for all variables that are associated with the outcome of interest or did the statistical analysis adjust for these prognostic variables? (3/16), 5. Can we be confident in the assessment of the presence or absence of prognostic factors? (14/16), 6. Can we confident in the assessment of outcome? (14/16), 7. Was the follow-up of cohorts adequate? (4/16)
Kansagara 2013 (52)	Transfusion	All-cause mortality	NR	Individual tool (based on NOS, AHRQ criteria)	Nonbiased selection? (11/11), high overall loss to follow-up or differential loss to follow-up? "No" for: (7/11), outcomes prespecified and defined? (11/11), ascertainment techniques adequately described? (9/11), nonbiased and adequate ascertainment methods? (7/11), propensity matching? (8/11), account for bleeding (6/11), account for timing of transfusion (3/11), adequate duration of follow-up? (verbal descriptions in systematic review)
Keag 2018 (84)	Caesarean section	Urinary incontinence	NR	RoBANS/ SIGN	Selection of participants (8/8), confounding variables (7/8), measurement of exposure (2/8), blinding of outcome assessments (0/8), incomplete outcome data (3/8), selective outcome reporting (8/8)/ overall quality: 0 (2/8)/ + (6/8)/ ++ (0/8)
Keag 2018 (84)	Caesarean section	Fecal incontinence	NR	RoBANS/ SIGN	Selection of participants (5/5), confounding variables (3/5), measurement of exposure (1/5), blinding of outcome assessments (0/5), incomplete outcome data (3/5), selective outcome reporting (5/5)/ overall quality: 0 (1/5)/ + (4/5)/ ++ (0/5)
Kredo 2014 (85)	Starting and maintaining antiretroviral therapy	All-cause mortality	Low	NOS, modified	Baseline CD4 count (1/2), other baseline variables (0/2), co-interventions (0/2), data collection (0/2), patient selection bias (2/2)
Kredo 2014 (85)	Starting and maintaining antiretroviral therapy	Attrition	Very low	NOS, modified	Baseline CD4 count (1/2), other baseline variables (0/2), co-interventions (0/2), data collection (0/2), patient selection bias (2/2)
Kredo 2014 (85)	Maintaining antiretroviral therapy	All-cause mortality	Very low	NOS, modified	Baseline CD4 count (1/1), other baseline variables (1/1), co-interventions (1/1), data collection (0/1), patient selection bias (1/1)
Li 2014 (54)	Exenatide	Acute pancreatitis	NR	NOS, modified with verbal rating	NA
Li 2016 (53)	DDP-4 inhibitors	Heart failure	Very low	NOS, modified with verbal rating	NA

Li 2016 (53)	DDP-4 inhibitors	Hospital admission for heart failure	Very low	NOS, modified with verbal rating	NA
Matthews 2018 (86)	Tamoxifen	Heart failure	NR	Cochrane tool, modified	Exposure definition (0/2), outcome/case definition (2/2), control selection (not applicable), confounding (2/2), missing Data (1/2), censoring (2/2)
Menne 2019 (87)	SGLT-2 inhibitors	Acute kidney injury	NR	NA	NR
Mesgarpour 2017 (88)	Erythropoiesis stimulating agents	Venous thromboembolism	Very low (RCTs and cohort studies)	NOS (number of studies with low risk of bias)	Representativeness of the exposed cohort? (5/5), selection of the non-exposed cohort? (5/5), ascertainment of exposure? (3/5), demonstration that the outcome of interest was not present at start of study? (5/5), comparability of cohorts on the basis of the design or analysis? (1/5), assessment of outcome? (2/5), was the follow-up long enough for the outcomes to occur? (1/5), adequacy of follow-up of the cohorts? (5/5)
Mesgarpour 2017 (88)	Erythropoiesis stimulating agents	All-cause mortality	Low (RCTs and cohort studies)	NOS (number of studies with low risk of bias)	Representativeness of the exposed cohort? (7/7), selection of the non-exposed cohort? (7/7), ascertainment of exposure? (5/7), demonstration that the outcome of interest was not present at start of study? (5/7), comparability of cohorts on the basis of the design or analysis? (4/7), assessment of outcome? (4/7), was the follow-up long enough for the outcomes to occur? (3/7), adequacy of follow-up of the cohorts? (7/7)
Moberley 2013 (89)	Pneumococcal polysaccharide vaccines	Invasive pneumococcal disease	NR	Cochrane tool, modified	Random sequence generation (0/2), selection of participants (2/2), confounding (2/2), performance bias (0/2), detection bias (2/2), incomplete outcome data (0/2), selective reporting (2/2)
Molnar 2015 (35)	Neoral (Cyclosporin)	Acute rejection of kidney transplant	NR	Tool by Wells et al. 2013	Number of studies with answer "yes" in brackets: comparison of intervention between two groups (2/2), intervention groups formed by time (2/2), retrospective design (2/2), confounding considered in study design or analysis (2/2), study protocol (1/2), outcome of acute rejection: prespecified, measured and analyzed (2/2)
Navarese 2013 (90)	Early intervention for NSTE-ACS	All-cause mortality	NR	NOS	NOS: 8.3
Navarese 2013 (90)	Early intervention for NSTE-ACS	Myocardial infarction	NR	NOS	NOS: 8.7

Navarese 2013 (90)	Early intervention for NSTE-ACS	Major bleeding	NR	NOS	NOS: 8.3
Nelson 2010 (36)	Caesarean section	Anal incontinence, feces	NR	NA	No presentation of risk of bias assessment; the risk of incomplete outcome data is only presented for part of the studies
Nelson 2010 (36)	Caesarean section	Anal incontinence, flatus	NR	NA	No presentation of risk of bias assessment; the risk of incomplete outcome data is only presented for part of the studies
Nieuwenhuijse 2014 (37)	Ceramic-on-ceramic bearings for total hip arthroplasty	Harris Hip Score	NR	Modified tool based on STROBE (studies rated as low to high quality); partly with verbal ratings	High quality (0/3)
Nieuwenhuijse 2014 (37)	High-flexion total knee arthroplasty	Flexion	NR	Modified tool based on STROBE (studies rated as low to high quality); partly with verbal ratings	High quality (0/26)
Nieuwenhuijse 2014 (37)	Gender-specific total knee arthroplasty	Flexion-extension range	NR	Modified tool based on STROBE (studies rated as low to high quality); partly with verbal ratings	High quality (0/2)
Nikooie 2019 (55)	Second generation antipsychotics	Sedation	Moderate (probably for RCTs only)	ROBINS-I	Confounding (0/3), selection of participants into study (0/3), classification of interventions (0/3), deviations from intended interventions (0/3), missing data (0/3), measurement of outcomes (0/3), selection of reported results (0/3)/ overall: low risk of bias (0/3)
Nikooie 2019 (55)	Second generation antipsychotics	Neurologic outcomes	NR	ROBINS-I	Confounding (0/5), selection of participants into study (1/5), classification of interventions (0/5), deviations from intended interventions (0/5), missing data (1/5), measurement of outcomes (0/5), selection of reported results (1/5)/ overall: low risk of bias (0/5)
Ochen 2019 (91)	Surgery for achilles tendon rupture	Re-rupture	NR	MINORS	MINORS score: 11.3
Ochen 2019 (91)	Surgery for achilles tendon rupture	Complications	NR	MINORS	MINORS score: 11

Pittas 2010 (60)	Vitamin D	Hypertension	NR	AHRQ/ STROBE	Fair quality (3/3)
Raman 2013 (38)	Carotid endarterectomy	Ipsilateral stroke	NR	AHRQ	Were eligibility criteria clear? (2/2), was selection bias unlikely? (0/2), were interventions adequately described? (0/2), were the outcomes fully defined? (1/2), no baseline imbalance between groups? (1/2), appropriate statistical analysis? (1/2), were potential confounders properly accounted for? (0/2), low risk of bias (0/2), funding source clear and not industry related? (1/2)/ overall: quality C (2/2)
Raman 2013 (38)	Carotid endarterectomy	Stroke	NR	AHRQ	Were eligibility criteria clear? (3/3), was selection bias unlikely? (0/3), were interventions adequately described? (0/3), were the outcomes fully defined? (1/3), no baseline imbalance between groups? (1/3), appropriate statistical analysis? (1/3), were potential confounders properly accounted for? (0/3), low risk of bias (0/3), funding source clear and not industry related? (2/3)/ overall: quality C (3/3)
Raman 2013 (38)	Carotid artery stenting	Periprocedural stroke	NR	AHRQ	Were eligibility criteria clear? (5/5), was selection bias unlikely? (5/5), were interventions adequately described? (1/5), were the outcomes fully defined? (5/5), no baseline imbalance between groups (1/5), appropriate statistical analysis (4/5), if multicenter, was this accounted for in analysis? (0, or NA/5), were potential confounders properly accounted for? (0/5), low risk of bias (0/5)/ overall: quality C (5/5)
Schweizer 2013 (39)	Nasal deconolization	Surgical site infection	NR	Downs and Black	Mean ratings: reporting (6.5/of max. 11), external validity (3/of max. 3), internal validity (2/of max. 7), internal validity confounding (1/of max.6), number of studies sufficiently powered (4/6)
Schweizer 2013 (39)	Glycopeptide prophylaxis	Surgical site infection	NR	Downs and Black	Mean ratings: reporting (8.4/of max. 11), external validity (3/of max. 3), internal validity (3/of max. 7), internal validity confounding (1.7/of max.6), number of studies sufficiently powered (2/7)
Silvain 2012 (40)	Enoxaparin	All-cause mortality	NR	NOS	NOS 7.9
Silvain 2012 (40)	Enoxaparin	Major bleeding	NR	NOS	NOS 8.1
Silvain 2012 (40)	Enoxaparin	All-cause mortality or myocardial infarction	NR	NOS	NOS:7.7

Suthar 2012 (26)	Antiretroviral therapy	Tuberculosis infection	NR	NOS, modified	Modified NOS; average 53.2% of the maximum score
Te Morenga 2013 (61)	Sugar	Weight gain	NR	NA	NR
Te Morenga 2013 (61)	Sugar	Body Mass Index	NR	NA	NR
Thomas 2010 (92)	Influenza vaccines	Influenza-like illness	NR	NOS with verbal rating, Cochrane tool	NOS NA/ Cochrane: adequate sequence generation (0/1), allocation concealment (0/1), blinding (0/1), incomplete outcome data addressed (0/1), free of selective reporting (1/1), free of other bias (0/1)
Tickell-Painter 2017 (93)	Mefloquine	Discontinuation due to adverse effects	High (RCTs and cohort studies)	ROBINS-I	Confounding (0/9), selection of participants (0/9), measurement of interventions (5/9), departures from intended interventions (2/9), missing data (8/9), measurement of outcomes (0/9), selection of the reported results (4/9), other (5/9)
Tickell-Painter 2017 (93)	Mefloquine	Serious adverse events or effects	NR	ROBINS-I	Confounding (0/2), selection of participants (1/2), measurement of interventions (2/2), departures from intended interventions (0/2), missing data (1/2), measurement of outcomes (0/2), selection of the reported results (1/2), other (0/2)
Tickell-Painter 2017 (93)	Mefloquine	Nausea	NR	ROBINS-I	Confounding (1/3), selection of participants (1/3), measurement of interventions (2/3), departures from intended interventions (0/3), missing data (1/3), measurement of outcomes (0/3), selection of the reported results (1/3), other (0/3)
Tricco 2018 (45)	Live-attenuated zoster vaccines	Suspected Herpes Zoster	NR	NOS, not convertable	Representativeness of the exposed cohort (1/3), selection of the non-exposed cohort (3/3), ascertainment of exposure (3/3), demonstration that outcome of interest was not present at start of study (3/3), comparability of cohorts on the basis of the design or analysis (0/3), assessment of outcome (3/3), adequacy of follow up of cohorts (3/3)
Vinceti 2018 (59)	Selenium	Cancer	Very low	NOS	NOS: 8
Vinceti 2018 (59)	Selenium	Cancer mortality	Very low	NOS	NOS: 8.5; NR for (5/7)

Vinceti 2018 (59)	Selenium	Colorectal cancer	Very low	NOS	NOS: 8.5
Wilson 2011 (41)	Traditional birth attendants	Perinatal mortality	NR	NOS, modified, not convertable	NA
Wilson 2011 (41)	Traditional birth attendants	Neonatal mortality	NR	NOS, modified, not convertable	NA
Wilson 2019 (42)	Unicompartimental knee arthroplasty	Venous thromboembolism	NR	NOS, modified, not convertable	NA
Wilson 2019 (42)	Unicompartimental knee arthroplasty	Flexion-extension range	NR	NOS, modified, not convertable	NA
Wilson 2019 (42)	Unicompartimental knee arthroplasty	Operation duration	NR	NOS, modified, not convertable	NA
Yank 2011 (44)	Recombinant factor VII	All-cause mortality	Low (RCTs and cohort studies)	AHRQ/ based on various tools (STROBE and others)	Study quality rated as good, fair, or poor: good quality (2/2)
Yank 2011 (44)	Recombinant factor VII	Thromboembolism	Moderate (RCTs and cohort studies)	AHRQ/ based on various tools (STROBE and others)	Study quality rated as good, fair, or poor: good quality (2/2)
Zhang 2016 (94)	Everolimus-eluting bioresorbable vascular scaffold	Stent thrombosis	NR	NOS	NOS: 8
Zhang 2016 (94)	Everolimus-eluting bioresorbable vascular scaffold	All-cause mortality	NR	NOS	NOS: 7.5

Zhang 2016 (94)	Everolimus-eluting bioresorbable vascular scaffold	Coronary heart disease mortality	NR	NOS	NOS: 8
Zhang 2017 (95)	Percutaneous coronary intervention	All-cause mortality	NR	NOS	NOS: 8.4
Zhang 2017 (95)	Percutaneous coronary intervention	Cardiovascular mortality	NR	NOS	NOS: 8.2
Zhang 2017 (95)	Percutaneous coronary intervention	Myocardial infarction	NR	NOS	NOS: 8.4
Ziff 2015 (96)	Digoxin	All-cause mortality	NR	RoBANS	Selection of participants (1/8), confounding variables (1/8), measurement of exposure (1/8), blinding of outcome (1/8), incomplete outcome data (1/8), selective reporting (2/8)
Ziff 2015 (96)	Digoxin	Cardiovascular mortality	NR	RoBANS	Selection of participants (0/3), confounding variables (0/3), measurement of exposure (0/3), blinding of outcome (0/3), incomplete outcome data (0/3), selective reporting (0/3)
Ziff 2015 (96)	Digoxin	Hospital admission	NR	RoBANS	Selection of participants (0/4), confounding variables (2/4), measurement of exposure (3/4), blinding of outcome (2/4), incomplete outcome data (2/4), selective reporting (2/4)

*rated with GRADE/ AHRQ criteria; AHRQ= agency for healthcare research and quality; CLARITY= clinical advances through research and information translation; CONSORT= consolidated standards of reporting trials; DDP-4= dipeptidyl peptidase 4; DHA= docosahexaenoic acid; EPA= eicosapentaenoic acid; GRADE= grades of recommendation, assessment, development, and evaluation; MINORS= methodological index for non-randomized studies; MOOSE= meta-analyses of observational studies in epidemiology; NA= not applicable; NOS= newcastle-ottawa scale; NR= not reported; NSTE-ACS= non-ST elevation acute coronary syndrome; QUATSO= quality assessment tool for systematic reviews of observational studies; RoB= risk of bias; RoBANS= risk of bias assessment tool for non-randomized studies; ROBINS: risk of bias in non-randomized studies of interventions; SIGN= scottish intercollegiate guidelines network; SGLT-2= sodium glucose transporter 2; STROBE= strengthening the reporting of observational studies in epidemiology; UPSTF= united states preventive services task force.

Table S8. Overview of the instruments used for the assessment of risk of bias for bodies of evidence of RCTs and cohort studies, heat map

	Risk of bias instrument in BoE of RCTs									Risk of bias instrument in BoE of cohort studies													
Systematic review	Cochrane	JADAD	CONSORT, AHRQ	USPSTF, AHRQ	AHRQ	SIGN	MINORS	Individual tools	None	NOS	(Modified) Cochrane tool	STROBE, AHRQ	ROBINS-1	USPSTF, AHRQ	AHRQ	CLARITY	RoBANS	RoBANS, SIGN	MINORS	Downs and Black	Wells et al., 2013	Individual tools	None
Abou-Setta 2011 (74)																							
Abou-Setta 2011 (74)																							
Aburto 2013 (75)																							
Aburto 2013 (75)																							
Ahmad 2015 (27)																							
Alexander 2017 (76)																							
Alexander 2017 (76)																							
Alexander 2017 (76)																							
Alipanah 2018 (24)																							
Alipanah 2018 (24)																							

Systematic review	Cochrane	JADAD	CONSORT, AHRQ	USPSTF, AHRQ	AHRQ	SIGN	MINORS	Individual tools	None	NOS	(Modified) Cochrane tool	STROBE, AHRQ	ROBINS-1	USPSTF, AHRQ	AHRQ	CLARITY	RoBANS	RoBANS, SIGN	MINORS	Downs and Black	Wells et al., 2013	Individual tools	None
Alipanah 2018 (24)																							
Anglemyer 2013 (77)																							
Azad 2017 (21)																							
Barnard 2015 (28)																							
Barnard 2015 (28)																							
Barnard 2015 (28)																							
Bellemain-Appaix 2012 (48)																							
Bellemain-Appaix 2012 (48)																							
Bellemain-Appaix 2012 (48)																							
Bellemain-Appaix 2014 (47)																							
Bellemain-Appaix 2014 (47)																							
Bellemain-Appaix 2014 (47)																							

Systematic review	Cochrane	JADAD	CONSORT, AHRQ	USPSTF, AHRQ	AHRQ	SIGN	MINORS	Individual tools	None	NOS	(Modified) Cochrane tool	STROBE, AHRQ	ROBINS-1	USPSTF, AHRQ	AHRQ	CLARITY	RoBANS	RoBANS, SIGN	MINORS	Downs and Black	Wells et al., 2013	Individual tools	None
Bloomfield 2016 (22)																							
Bolland 2015 (49)																							
Bolland 2015 (49)																							
Bolland 2015 (49)																							
Brenner 2014 (29)																							
Brenner 2014 (29)																							
Chowdhury 2012 (78)																							
Chowdhury 2014a (79)																							
Chowdhury 2014a (79)																							
Chowdhury 2014a (79)																							
Chowdhury 2014b (80)																							
Chung 2011 (58)																							
Chung 2011 (58)																							
Chung 2016 (56)																							

Systematic review	Cochrane	JADAD	CONSORT, AHRQ	USPSTF, AHRQ	AHRQ	SIGN	MINORS	Individual tools	None	NOS	(Modified) Cochrane tool	STROBE, AHRQ	ROBINS-1	USPSTF, AHRQ	AHRQ	CLARITY	RoBANS	RoBANS, SIGN	MINORS	Downs and Black	Wells et al., 2013	Individual tools	None
Ding 2017 (81)																							
Fenton 2018 (30)																							
Fenton 2018 (30)																							
Fenton 2018 (30)																							
Filippini 2017 (43)																							
Fluri 2010 (31)																							
Fluri 2010 (31)																							
Fluri 2010 (31)																							
Gargiulo 2016 (32)																							
Gargiulo 2016 (32)																							
Gargiulo 2016 (32)																							
Hartling 2013 (50)																							
Hartling 2013 (50)																							
Hartling 2013 (50)																							

Systematic review	Cochrane	JADAD	CONSORT, AHRQ	USPSTF, AHRQ	AHRQ	SIGN	MINORS	Individual tools	None	NOS	(Modified) Cochrane tool	STROBE, AHRQ	ROBINS-1	USPSTF, AHRQ	AHRQ	CLARITY	RoBANS	RoBANS, SIGN	MINORS	Downs and Black	Wells et al., 2013	Individual tools	None
Henderson 2019 (51)																							
Higgins 2016 (25)																							
Higgins 2016 (25)																							
Hopley 2010 (33)																							
Hopley 2010 (33)																							
Hopley 2010 (33)																							
Hüpfl 2010 (67)																							
Jamal 2013 (82)																							
Jefferson 2010 (46)																							
Jefferson 2010 (46)																							
Jefferson 2012 (34)																							
Jefferson 2012 (34)																							
Jin 2012 (83)																							
Johnston 2019 (23)																							

Systematic review	Cochrane	JADAD	CONSORT, AHRQ	USPSTF, AHRQ	AHRQ	SIGN	MINORS	Individual tools	None	NOS	(Modified) Cochrane tool	STROBE, AHRQ	ROBINS-1	USPSTF, AHRQ	AHRQ	CLARITY	RoBANS	RoBANS, SIGN	MINORS	Downs and Black	Wells et al., 2013	Individual tools	None
Johnston 2019 (23)																							
Johnston 2019 (23)																							
Kansagara 2013 (52)																							
Keag 2018 (84)																							
Keag 2018 (84)																							
Kredo 2014 (85)																							
Kredo 2014 (85)																							
Kredo 2014 (85)																							
Li 2014 (54)																							
Li 2016 (53)																							
Li 2016 (53)																							
Matthews 2018 (86)																							
Menne 2019 (87)																							
Mesgarpour 2017 (88)																							
Mesgarpour 2017 (88)																							

Systematic review	Cochrane	JADAD	CONSORT, AHRQ	USPSTF, AHRQ	AHRQ	SIGN	MINORS	Individual tools	None	NOS	(Modified) Cochrane tool	STROBE, AHRQ	ROBINS-1	USPSTF, AHRQ	AHRQ	CLARITY	RoBANS	RoBANS, SIGN	MINORS	Downs and Black	Wells et al., 2013	Individual tools	None
Moberley 2013 (89)																							
Molnar 2015 (35)																							
Navarese 2013 (90)																							
Navarese 2013 (90)																							
Navarese 2013 (90)																							
Nelson 2010 (36)																							
Nelson 2010 (36)																							
Nieuwenhuijse 2014 (37)																							
Nieuwenhuijse 2014 (37)																							
Nieuwenhuijse 2014 (37)																							
Nikooie 2019 (55)																							
Nikooie 2019 (55)																							
Ochen 2019 (91)																							

Systematic review	Cochrane	JADAD	CONSORT, AHRQ	USPSTF, AHRQ	AHRQ	SIGN	MINORS	Individual tools	None	NOS	(Modified) Cochrane tool	STROBE, AHRQ	ROBINS-1	USPSTF, AHRQ	AHRQ	CLARITY	RoBANS	RoBANS, SIGN	MINORS	Downs and Black	Wells et al., 2013	Individual tools	None
Ochen 2019 (91)																							
Pittas 2010 (60)																							
Raman 2013 (38)																							
Raman 2013 (38)																							
Raman 2013 (38)																							
Schweizer 2013 (39)																							
Schweizer 2013 (39)																							
Silvain 2012 (40)																							
Silvain 2012 (40)																							
Silvain 2012 (40)																							
Suthar 2012 (26)																							
Te Morenga 2013 (61)																							
Te Morenga 2013 (61)																							
Thomas 2010 (92)																							

Systematic review	Cochrane	JADAD	CONSORT, AHRQ	USPSTF, AHRQ	AHRQ	SIGN	MINORS	Individual tools	None	NOS	(Modified) Cochrane tool	STROBE, AHRQ	ROBINS-1	USPSTF, AHRQ	AHRQ	CLARITY	RoBANS	RoBANS, SIGN	MINORS	Downs and Black	Wells et al., 2013	Individual tools	None
Tickell-Painter 2017 (93)																							
Tickell-Painter 2017 (93)																							
Tickell-Painter 2017 (93)																							
Tricco 2018 (45)																							
Vinceti 2018 (59)																							
Vinceti 2018 (59)																							
Vinceti 2018 (59)																							
Wilson 2011 (41)																							
Wilson 2011 (41)																							
Wilson 2019 (42)																							
Wilson 2019 (42)																							
Wilson 2019 (42)																							
Yank 2011 (44)																							
Yank 2011 (44)																							

Systematic review	Cochrane	JADAD	CONSORT, AHRQ	USPSTF, AHRQ	AHRQ	SIGN	MINORS	Individual tools	None	NOS	(Modified) Cochrane tool	STROBE, AHRQ	ROBINS-1	USPSTF, AHRQ	AHRQ	CLARITY	RoBANS	RoBANS, SIGN	MINORS	Downs and Black	Wells et al., 2013	Individual tools	None
Zhang 2016 (94)																							
Zhang 2016 (94)																							
Zhang 2016 (94)																							
Zhang 2017 (95)																							
Zhang 2017 (95)																							
Zhang 2017 (95)																							
Ziff 2015 (96)																							
Ziff 2015 (96)																							
Ziff 2015 (96)																							

AHRQ= agency for healthcare research and quality; CLARITY= clinical advances through research and information translation; CONSORT= consolidated standards of reporting trials; MINORS= methodological index for non-randomized studies; MOOSE= meta-analyses of observational studies in epidemiology; NOS= newcastle-ottawa scale; RoBANS= risk of bias assessment tool for non-randomized studies; ROBINS: risk of bias in non-randomized studies of interventions; SIGN= scottish intercollegiate guidelines network; STROBE= strengthening the reporting of observational studies in epidemiology; UPSTF= united states preventive services task force.

Table S9. Ratings of PI/ECO*-similarity degree for included body of evidence-pairs // *PI/ECO= population, intervention/ exposure, comparator, outcome

Reference/ year	Intervention/Exposure	Outcome	Population	Intervention/ exposure	Comparator	Outcome	Overall
Abou-Setta 2011 (74)	Nerve block	Delirium	2	2	2	1	2
Abou-Setta 2011 (74)	Spinal anesthesia	Mortality	2	1	1	1	2
Aburto 2013 (75)	Low sodium	Mortality	2	2	2	1	2
Aburto 2013 (75)	Low sodium	Cardiovascular disease	2	2	2	2	2
Ahmad 2015 (27)	Intra-aortic balloon pump	Mortality	1	1	1	1	1
Alexander 2017 (76)	Docosahexaenoic acid and eicosapentaenoic acid	Any coronary heart disease event	2	2	2	1	2
Alexander 2017 (76)	Docosahexaenoic acid and eicosapentaenoic acid	Fatal coronary heart disease events	2	2	2	1	2
Alexander 2017 (76)	Docosahexaenoic acid and eicosapentaenoic acid	Non-fatal coronary heart disease events	2	2	2	1	2
Alipanah 2018 (24)	Self-administered therapy	Low treatment success	3	2	1	1	3
Alipanah 2018 (24)	Self-administered therapy	Low treatment completion	3	2	1	1	3
Alipanah 2018 (24)	Self-administered therapy	Mortality	3	2	1	1	3
Anglemyer 2013 (77)	Antiretroviral therapy	HIV infection	2	2	3	1	3
Azad 2017 (21)	Nonnutritive sweeteners	BMI	2	2	1	1	2
Barnard 2015 (28)	Surgical abortion by mid-level providers	Failure or incomplete abortion	2	2	1	1	2
Barnard 2015 (28)	Surgical abortion by mid-level providers	Complications	2	2	1	1	2
Barnard 2015 (28)	Surgical abortion by mid-level providers	Abortion failure and complications	2	2	1	1	2
Bellemain-Appaix 2012 (48)	Clopidogrel	Mortality	2	2	2	1	2
Bellemain-Appaix 2012 (48)	Clopidogrel	Major bleeding	2	2	2	1	2
Bellemain-Appaix 2012 (48)	Clopidogrel	Major coronary event	2	2	2	1	2
Bellemain-Appaix 2014 (47)	P2Y12 Inhibitors	Mortality	2	2	2	1	2
Bellemain-Appaix 2014 (47)	P2Y12 Inhibitors	Major bleeding	2	2	2	1	2

Bellemain-Appaix 2014 (47)	P2Y12 Inhibitors	Main composite ischemic endpoint	2	2	2	1	2
Bloomfield 2016 (22)	Mediterranean diet	Breast cancer	2	2	2	1	2
Bolland 2015 (49)	Calcium supplements	All fractures	1	2	1	1	2
Bolland 2015 (49)	Calcium supplements	Vertebral fracture	1	2	1	1	2
Bolland 2015 (49)	Calcium supplements	Hip fracture	1	2	1	1	2
Brenner 2014 (29)	Sigmoidoscopy, screening for CRC	Colorectal cancer mortality	1	1	1	1	1
Brenner 2014 (29)	Sigmoidoscopy, screening for CRC	Colorectal cancer incidence	1	1	1	2	2
Chowdhury 2012 (78)	Omega-3-fatty acids	Cerebrovascular disease	2	2	1	1	2
Chowdhury 2014a (79)	α-linolenic acid	Coronary event	3	2	2	1	3
Chowdhury 2014a (79)	Omega-3-fatty acids	Coronary event	3	2	2	1	3
Chowdhury 2014a (79)	Omega-6-fatty acids	Coronary event	3	2	2	1	3
Chowdhury 2014b (80)	Vitamin D	Mortality	2	3	3	1	3
Chung 2011 (58)	Vitamin D	Colorectal cancer	2	3	3	1	3
Chung 2011 (58)	Vitamin D	Breast cancer	2	3	3	1	3
Chung 2016 (56)	Calcium	Cardiovascular mortality	2	2	1	1	2
Ding 2017 (81)	Dairy	Systolic blood pressure	2	1	1	1	2
Fenton 2018 (30)	Radiation therapy	Erectile dysfunction	1	1	2	1	2
Fenton 2018 (30)	Radical Prostatectomy	Urinary incontinence	1	1	2	1	2
Fenton 2018 (30)	Radical Prostatectomy	Erectile dysfunction	1	1	2	1	2
Filippini 2017 (43)	Disease-modifying drugs	Conversion to clinically definite multiple sclerosis	1	2	1	1	2
Fluri 2010 (31)	Extracranial-intracranial arterial bypass	Mortality	2	1	2	1	2
Fluri 2010 (31)	Extracranial-intracranial arterial bypass	Any stroke	2	1	2	1	2
Fluri 2010 (31)	Extracranial-intracranial arterial bypass	Death or dependency	1	1	2	1	2
Gargiulo 2016 (32)	Transcatheter aortic valve	Early mortality	2	2	1	1	2
Gargiulo 2016 (32)	Transcatheter aortic valve	Mid-term mortality	2	2	1	1	2
Gargiulo 2016 (32)	Transcatheter aortic valve	Long-term mortality	2	1	1	1	2

Hartling 2013 (50)	Treating gestational diabetes mellitus	Birth weight > 4000g	2	1	1	1	2
Hartling 2013 (50)	Treating gestational diabetes mellitus	Large-for-gestational age neonate	2	1	1	1	2
Hartling 2013 (50)	Treating gestational diabetes mellitus	Shoulder dystocia	2	1	1	1	2
Henderson 2019 (51)	Treating asymptomatic bacteriuria	Pyelonephritis	2	2	3	1	3
Higgins 2016 (25)	Bacillus Calmette-Guérin	Mortality	3	1	2	1	3
Higgins 2016 (25)	Measles containing vaccines	Mortality	3	2	2	1	3
Hopley 2010 (33)	Total hip arthroplasty	Reoperation	2	1	1	1	2
Hopley 2010 (33)	Total hip arthroplasty	Dislocation	2	1	1	1	2
Hopley 2010 (33)	Total hip arthroplasty	Deep infection	2	2	1	1	2
Hüpfl 2010 (67)	Chest-compression-only cardiopulmonary resuscitation	All-cause mortality	1	3	3	1	3
Jamal 2013 (82)	Non-calcium-based phosphat binders	Mortality	2	2	1	1	2
Jefferson 2010 (46)	Parenteral influenza vaccine	Influenza-like illness	2	2	3	1	3
Jefferson 2010 (46)	Parenteral influenza vaccine	Influenza	2	2	1	1	2
Jefferson 2012 (34)	Inactivated influenza vaccines	Influenza	1	2	1	1	2
Jefferson 2012 (34)	Inactivated influenza vaccines	Influenza-like illness	1	2	1	1	2
Jin 2012 (83)	Total flavonoids	Colorectal neoplasms	3	2	2	3	3
Johnston 2019 (23)	Low red meat	Mortality	2	2	2	1	2
Johnston 2019 (23)	Low red meat	Cardiovascular mortality	2	2	2	1	2
Johnston 2019 (23)	Low red meat	Cardiovascular disease	2	2	2	1	2
Kansagara 2013 (52)	Transfusion	Mortality	2	3	3	2	3
Keag 2018 (84)	Caesarean section	Urinary incontinence	3	2	2	1	3
Keag 2018 (84)	Caesarean section	Fecal incontinence	3	2	2	1	3
Kredo 2014 (85)	Nurse or clinical officer for initiation and maintenance of antiretroviral therapy	Mortality	2	3	1	1	3
Kredo 2014 (85)	Nurse or clinical officer for initiation and maintenance of antiretroviral therapy	Lost to follow-up	2	3	1	1	3
Kredo 2014 (85)	Nurse or clinical officer for maintenance of antiretroviral therapy	Mortality	2	3	2	1	3
Li 2014 (54)	Exenatide	Acute pancreatitis/ Admission for acute pancreatitis	2	1	2	2	2
Li 2016 (53)	DDP-4 Inhibitors	Heart failure	2	2	2	1	2

Li 2016 (53)	DDP-4 Inhibitors	Hospital admission for heart failure	2	2	2	1	2
Matthews 2018 (86)	Tamoxifen	Heart failure	2	3	1	1	3
Menne 2019 (87)	SGLT-2 inhibitors	Acute kidney injury	2	2	2	1	2
Mesgarpour 2017 (88)	Erythropoiesis stimulating agents	Venous thromboembolism	2	2	2	1	2
Mesgarpour 2017 (88)	Erythropoiesis stimulating agents	Mortality	2	2	2	1	2
Moberley 2013 (89)	Pneumococcal polysaccharide vaccines	Invasive pneumococcal disease	2	2	1	1	2
Molnar 2015 (35)	Neoral (Cyclosporin)	Acute rejection of kidney transplant	2	1	2	1	2
Navarese 2013 (90)	Early intervention for NSTE-ACS	Mortality	2	2	1	1	2
Navarese 2013 (90)	Early intervention for NSTE-ACS	Myocardial infarction	2	2	1	1	2
Navarese 2013 (90)	Early intervention for NSTE-ACS	Major bleeding	2	2	1	1	2
Nelson 2010 (36)	Caesarean section	Anal incontinence, feces	3	2	2	1	3
Nelson 2010 (36)	Caesarean section	Anal incontinence, flatus	3	2	2	1	3
Nieuwenhuijse 2014 (37)	Ceramic-on-ceramic bearings for total hip arthroplasty	Harris Hip Score	2	1	1	1	2
Nieuwenhuijse 2014 (37)	High-flexion total knee arthroplasty	Flexion (degrees)	2	1	1	1	2
Nieuwenhuijse 2014 (37)	Gender-specific total knee arthroplasty	Flexion-extension range (degrees)	2	1	1	1	2
Nikooie 2019 (55)	Second generation antipsychotics	Sedation	2	2	1	2	2
Nikooie 2019 (55)	Second generation antipsychotics	Neurologic outcomes	2	2	1	2	2
Ochen 2019 (91)	Surgery for achilles tendon rupture	Re-rupture	1	2	2	1	2
Ochen 2019 (91)	Surgery for achilles tendon rupture	Complications	1	2	2	1	2
Pittas 2010 (60)	Vitamin D	Hypertension	2	3	3	1	3
Raman 2013 (38)	Carotid endarterectomy	Ipsilateral stroke	2	1	2	1	2
Raman 2013 (38)	Carotid endarterectomy	Any stroke	2	1	2	1	2
Raman 2013 (38)	Carotid artery stenting	Periprocedural stroke	2	2	2	1	2
Schweizer 2013 (39)	Nasal deconolization	Surgical site infection	2	2	1	1	2
Schweizer 2013 (39)	Glycopeptide prophylaxis	Surgical site infection	2	2	2	1	2
Silvain 2012 (40)	Enoxaparin	Mortality	2	1	2	1	2
Silvain 2012 (40)	Enoxaparin	Major bleeding	2	1	2	1	2

Silvain 2012 (40)	Enoxaparin	Death or Myocardial infarction	2	1	2	1	2
Suthar 2012 (26)	Antiretroviral therapy	Tuberculosis infection	2	3	3	1	3
Te Morenga 2013 (61)	Sugar	Weight gain (kg)	2	1	2	1	2
Te Morenga 2013 (61)	Sugar	BMI (kg/m^2)	2	2	2	1	2
Thomas 2010 (92)	Influenza vaccines	Influenza-like illness	2	3	3	1	3
Tickell-Painter 2017 (93)	Mefloquine	Discontinuation due to adverse effects	2	1	1	1	2
Tickell-Painter 2017 (93)	Mefloquine	Serious adverse events or effects	3	1	1	2	3
Tickell-Painter 2017 (93)	Mefloquine	Nausea	3	1	1	1	3
Tricco 2018 (45)	Live-attenuated zoster vaccines	Suspected Herpes Zoster	2	2	2	1	2
Vinceti 2018 (59)	Selenium	Any cancer	2	3	3	2	3
Vinceti 2018 (59)	Selenium	Cancer mortality	2	3	3	1	3
Vinceti 2018 (59)	Selenium	Colorectal cancer	2	3	3	1	3
Wilson 2011 (41)	Traditional birth attendants	Perinatal mortality	1	2	3	1	3
Wilson 2011 (41)	Traditional birth attendants	Neonatal mortality	1	2	3	1	3
Wilson 2019 (42)	Unicompartimental knee arthroplasty	Venous thromboembolism	2	1	1	1	2
Wilson 2019 (42)	Unicompartimental knee arthroplasty	Range of movement (degrees)	2	1	1	1	2
Wilson 2019 (42)	Unicompartimental knee arthroplasty	Operation duration (minutes)	2	1	1	1	2
Yank 2011 (44)	Recombinant factor VII	Mortality	2	2	1	1	2
Yank 2011 (44)	Recombinant factor VII	Thromboembolic events	2	2	1	1	2
Zhang 2016 (94)	Everolimus-eluting bioresorbable vascular scaffold	Stent thrombosis	2	1	1	1	2
Zhang 2016 (94)	Everolimus-eluting bioresorbable vascular scaffold	Mortality	2	1	1	1	2
Zhang 2016 (94)	Everolimus-eluting bioresorbable vascular scaffold	Cardiac death	2	1	1	1	2
Zhang 2017 (95)	Percutaneous coronary intervention	Mortality	2	2	1	1	2
Zhang 2017 (95)	Percutaneous coronary intervention	Cardiovascular mortality	2	2	1	1	2
Zhang 2017 (95)	Percutaneous coronary intervention	Myocardial infarction	2	2	1	1	2
Ziff 2015 (96)	Digoxin	Mortality	3	1	1	1	3
Ziff 2015 (96)	Digoxin	Cardiovascular mortality	3	1	1	1	3

Ziff 2015 (96)	Digoxin	Hospital admission	2	1	1	1	2

DDP-4= dipeptidyl peptidase 4; NSTE-ACS= non-ST elevation acute coronary syndrome; SGLT-2= sodium glucose transporter 2.

Table S10. Effect estimates and statistical heterogeneity for meta-analyses of RCTs and cohort studies

Body of evidence-pair			Meta-analysis of RCTs			Meta-analysis of cohort studies		
**Systematic review**	**Intervention/ Exposure**	**Outcome**	**Number of studies**	**Summary measure; effect estimates (95% CI)**	$I^{2\,(\%)}$	**Number of studies**	**Summary measure; effect estimates (95% CI)**	$I^{2\,(\%)}$
Abou-Setta 2011 (74)	Nerve block	Delirium	4	OR: 0.33 (0.16, 0.66)	0	2	OR: 0.24 (0.08, 0.72)	60
Abou-Setta 2011 (74)	Spinal anesthesia	All-cause mortality	2	OR: 1.73 (0.53, 5.68)	0	5	OR: 0.87 (0.45, 1.67)	61
Aburto 2013 (75)	Low sodium	All-cause mortality	4	RR: 0.7 (0.44, 1.14)	0	7	RR: 0.94 (0.83, 1.06)	61
Aburto 2013 (75)	Low sodium	Cardiovascular disease	2	RR: 0.84 (0.57, 1.23)	0	9	RR: 0.89 (0.75, 1.08)	78
Ahmad 2015 (27)	Intra-aortic balloon pump	All-cause mortality	12	OR: 0.96 (0.74, 1.24)	0	14	OR: 1.02 (0.57, 1.82)	97
Alexander 2017 (76)	DHA and EPA	Coronary heart disease	18	RR: 0.94 (0.85, 1.05)	36	17	RR: 0.82 (0.74, 0.92)	66
Alexander 2017 (76)	DHA and EPA	Coronary heart disease mortality	14	RR: 1 (0.89, 1.11)	NR	14	RR: 0.77 (0.66, 0.9)	NR
Alexander 2017 (76)	DHA and EPA	Coronary heart disease incidence	9	RR: 0.92 (0.78, 1.09)	NR	4	RR: 0.81 (0.55, 1.19)	NR
Alipanah 2018 (24)	Self-administered therapy	Low treatment success	4	1.05 (0.96, 1.15)	29	16	RR: 1.23 (1.12, 1.37	93
Alipanah 2018 (24)	Self-administered therapy	Low treatment completion	5	RR: 1.27 (0.9, 1.79)	45	14	RR: 0.91 (0.74, 1.11)	88
Alipanah 2018 (24)	Self-administered therapy	All-cause mortality	4	RR: 0.73 (0.45, 1.19)	0	23	RR: 1.35 (1, 1.84)	90
Anglemyer 2013 (77)	Antiretroviral therapy	HIV infection	1	RR: 0.11 (0.04, 0.32)	NA	9	RR: 0.58 (0.35, 0.96)	64
Azad 2017 (21)	Nonnutritive sweeteners	Body Mass Index	3	MD: -0.37 (-1.1, 0.36)	9	1	MD: 0.77 (0.47, 1.07)	NA
Barnard 2015 (28)	Surgical abortion by mid-level providers	Failure or incomplete abortion	2	RR: 2.97 (0.21, 41.82)	70	2	RR: 2.47 (1.45, 4.22)	0

Barnard 2015 (28)	Surgical abortion by mid-level providers	Complications	2	RR: 0.99 (0.17, 5.7)	0	2	RR: 1.3 (0.57, 2.96)	70
Barnard 2015 (28)	Surgical abortion by mid-level providers	Abortion failure and complications	2	RR: 3.07 (0.16, 59.08)	76	3	RR: 1.33 (0.78, 2.27)	74
Bellemain-Appaix 2012 (48)	Clopidogrel	All-cause mortality	7	OR: 0.8 (0.57, 1.11)	0	8	OR: 0.79 (0.52, 1.2)	80
Bellemain-Appaix 2012 (48)	Clopidogrel	Major bleeding	7	OR: 1.18 (0.93, 1.5)	0	8	OR: 1.16 (0.83, 1.61)	49
Bellemain-Appaix 2012 (48)	Clopidogrel	Coronary heart disease	7	OR: 0.77 (0.66, 0.89)	4	8	OR: 0.76 (0.6, 0.95)	82
Bellemain-Appaix 2014 (47)	P2Y12 inhibitors	All-cause mortality	3	OR: 0.92 (0.43, 1.98)	13	4	OR: 0.69 (0.38, 1.25)	26
Bellemain-Appaix 2014 (47)	P2Y12 inhibitors	Major bleeding	3	OR: 1.45 (0.97, 2.15)	25	4	OR: 1.12 (0.87, 1.45)	0
Bellemain-Appaix 2014 (47)	P2Y12 inhibitors	Main composite ischemic endpoint	3	OR: 0.85 (0.67, 1.07)	44	4	OR: 0.79 (0.54, 1.15)	70
Bloomfield 2016 (22)	Mediterranean diet	Breast cancer	1	RR: 0.53 (0.28, 1.03)	NA	13	RR: 0.96 (0.9, 1.03)	52
Bolland 2015 (49)	Calcium	All fractures	22	RR: 0.9 (0.83, 0.96)	23	5	RR: 1.02 (0.93, 1.12)	68
Bolland 2015 (49)	Calcium	Vertebral fracture	12	RR: 0.86 (0.74, 1)	0	1	RR: 1.4 (1.1, 1.9)	NA
Bolland 2015 (49)	Calcium	Hip fracture	13	RR: 0.95 (0.76, 1.18)	36	6	RR: 1.09 (0.91, 1.3)	50
Brenner 2014 (29)	Sigmoidoscopy	Colorectal cancer mortality	4	RR: 0.72 (0.65, 0.8)	0	1	RR: 0.59 (0.45, 0.76)	NA
Brenner 2014 (29)	Sigmoidoscopy	Colorectal cancer incidence	4	RR: 0.82 (0.75, 0.89)	52	2	RR: 0.5 (0.37, 0.69)	0
Chowdhury 2012 (78)	Omega-3	Cerebrovascular disease	2	RR: 0.98 (0.89, 1.08)	12	10	RR: 0.9 (0.8, 1.01)	17
Chowdhury 2014a (79)	α-linolenic acid	Coronary heart disease	4	RR: 0.97 (0.69, 1.36)	52	7	RR: 0.99 (0.86, 1.14)	62
Chowdhury 2014a (79)	Omega-3	Coronary heart disease	17	RR: 0.94 (0.86, 1.03)	17	16	RR: 0.87 (0.78, 0.97)	76

Chowdhury 2014a (79)	Omega-6	Coronary heart disease	8	RR: 0.86 (0.69, 1.07)	59	8	RR: 0.98 (0.9, 1.06)	53
Chowdhury 2014b (80)	Vitamin D	All-cause mortality	22	RR: 0.98 (0.94, 1.02)	NR	68	RR: 0.69 (0.65, 0.75)	NR
Chung 2011 (58)	Vitamin D	Colorectal cancer	1	RR: 1.02 (0.6, 1.74)	NA	9	RR: 0.94 (0.91, 0.97)	NR
Chung 2011 (58)	Vitamin D	Breast cancer	1	RR: 0.99 (0.25, 4)	NA	4	RR: 0.99 (0.97, 1.01)	NR
Chung 2016 (56)	Calcium	Cardiovascular mortality	2	RR: 1.05 (0.82, 1.33)	0	6	RR: 0.99 (0.97, 1.01)	7
Ding 2017 (81)	Dairy	Systolic blood pressure	8	MD: -0.21 (-0.98, 0.57)	0	27	MD: -0.11 (-0.2, -0.02)	30
Fenton 2018 (30)	Radiation therapy	Erectile dysfunction	1	RR: 0.91 (0.77, 1.08)	NA	7	RR: 1.3 (1.19, 1.43)	33
Fenton 2018 (30)	Radical Prostatectomy	Urinary incontinence	3	RR: 2.27 (1.82, 2.84)	0	5	RR: 2.92 (1.8, 4.71)	67
Fenton 2018 (30)	Radical Prostatectomy	Erectile dysfunction	3	RR: 1.6 (1.23, 2.07)	87	6	RR: 1.49 (1.33, 1.66)	63
Filippini 2017 (43)	Disease-modifying drugs	Conversion to clinically definite multiple sclerosis	7	HR: 0.52 (0.46, 0.6)	0	2	HR: 0.48 (0.3, 0.78)	62
Fluri 2010 (31)	Extracranial-intracranial arterial bypass	All-cause mortality	2	OR: 0.81 (0.62, 1.05)	0	11	OR: 1 (0.62, 1.63)	0
Fluri 2010 (31)	Extracranial-intracranial arterial bypass	Stroke	2	OR: 0.99 (0.79, 1.23)	86	15	OR: 0.8 (0.54, 1.18)	3
Fluri 2010 (31)	Extracranial-intracranial arterial bypass	Stroke mortality or dependency	1	OR: 0.94 (0.74, 1.21)	NA	8	OR: 0.8 (0.5, 1.29)	0
Gargiulo 2016 (32)	Transcatheter aortic valve	Early all-cause mortality	5	OR: 0.8 (0.51, 1.25)	0	29	OR: 1.08 (0.84, 1.39)	41
Gargiulo 2016 (32)	Transcatheter aortic valve	Mid-term all-cause mortality	5	OR: 0.9 (0.64, 1.26)	22	18	OR: 1 (0.81, 1.24)	46
Gargiulo 2016 (32)	Transcatheter aortic valve	Long-term all-cause mortality	4	OR: 1.03 (0.65, 1.62)	65	6	OR: 1.7 (1.23, 2.35)	0
Hartling 2013 (50)	Treating gestational diabetes mellitus	High birth weight	5	RR: 0.5 (0.35, 0.71)	50	5	RR: 0.69 (0.31, 1.54)	88

Hartling 2013 (50)	Treating gestational diabetes mellitus	Large-for-gestational age neonate	3	RR: 0.56 (0.45, 0.69)	0	4	RR: 0.43 (0.27, 0.7)	58
Hartling 2013 (50)	Treating gestational diabetes mellitus	Shoulder dystocia	3	RR: 0.42 (0.23, 0.77)	0	4	RR: 0.38 (0.19, 0.78)	20
Henderson 2019 (51)	Treating asymptomatic bacteriuria	Pyelonephritis	12	RR: 0.24 (0.14, 0.4)	56	2	RR: 0.29 (0.15, 0.57)	0
Higgins 2016 (25)	Bacillus Calmette-Guérin	All-cause mortality	3	RR: 0.67 (0.4, 1.14)	58	8	RR: 0.46 (0.3, 0.69)	63
Higgins 2016 (25)	Measles containing vaccines	All-cause mortality	4	RR: 0.74 (0.51, 1.07)	0	13	RR: 0.53 (0.4, 0.7)	67
Hopley 2010 (33)	Total hip arthroplasty	Reoperation	4	RR: 1.09 (0.4, 2.99)	30	6	RR: 0.45 (0.18, 1.09)	26
Hopley 2010 (33)	Total hip arthroplasty	Dislocation	4	RR: 2.47 (0.69, 8.76)	0	5	RR: 0.8 (0.27, 2.39)	20
Hopley 2010 (33)	Total hip arthroplasty	Deep infection	4	RR: 1.71 (0.66, 4.45)	0	4	RR: 0.91 (0.25, 3.28)	0
Hüpfl 2010 (67)	Chest-compression-only cardiopulmonary resuscitation	All-cause mortality	3	RR: 0.82 (0.68, 0.99)	0	7	RR: 1.04 (0.9, 1.2)	0
Jamal 2013 (82)	Non-calcium-based phosphat binders	All-cause mortality	8	RR: 0.78 (0.61, 0.98)	43	3	RR: 0.89 (0.78, 1)	0
Jefferson 2010 (46)	Parenteral influenza vaccine	Influenza-like illness	4	RR: 0.59 (0.47, 0.73)	0	30	RR: 0.76 (0.66, 0.87)	57
Jefferson 2010 (46)	Parenteral influenza vaccine	Influenza	3	RR: 0.42 (0.27, 0.66)	0	10	RR: 0.5 (0.26, 0.97)	67
Jefferson 2012 (34)	Inactivated influenza vaccines	Influenza	5	RR: 0.41 (0.29, 0.59)	36	1	RR: 0.2 (0.1, 0.39)	NA
Jefferson 2012 (34)	Inactivated influenza vaccines	Influenza-like illness	5	RR: 0.64 (0.54, 0.76)	67	2	RR: 0.29 (0.07, 1.15)	95
Jin 2012 (83)	Total flavonoids	Colorectal neoplasms	1	RR: 1.09 (0.93, 1.28)	NA	3	RR: 1 (0.8, 1.25)	66
Johnston 2019 (23)	Low red meat	All-cause mortality	1	RR: 0.94 (0.89, 0.99)	NA	24	RR: 0.87 (0.82, 0.92)	NR
Johnston 2019 (23)	Low red meat	Cardiovascular mortality	1	RR: 1 (0.84, 1.19)	NA	25	RR: 0.86 (0.79, 0.94)	NR

Johnston 2019 (23)	Low red meat	Cardiovascular disease	1	RR: 0.97 (0.91, 1.04)	NA	12	RR: 0.87 (0.75, 1.01)	NR
Kansagara 2013 (52)	Transfusion	All-cause mortality	6	RR: 0.94 (0.61, 1.42)	17	11	RR: 2.49 (1.4, 4.43)	97
Keag 2018 (84)	Caesarean section	Urinary incontinence	1	OR: 0.78 (0.56, 1.08)	NA	8	OR: 0.56 (0.47, 0.66)	71
Keag 2018 (84)	Caesarean section	Fecal incontinence	1	OR: 3.07 (0.9, 10.49)	NA	5	OR: 1.04 (0.73, 1.48)	72
Kredo 2014 (85)	Starting and maintaining antiretroviral therapy	All-cause mortality	1	RR: 0.96 (0.82, 1.12)	NA	2	RR: 1.23 (1.14, 1.33)	0
Kredo 2014 (85)	Starting and maintaining antiretroviral therapy	Attrition	1	RR: 0.73 (0.55, 0.97)	NA	2	RR: 0.3 (0.05, 1.94)	98
Kredo 2014 (85)	Maintaining antiretroviral therapy	All-cause mortality	2	RR: 0.89 (0.59, 1.32)	0	1	RR: 0.19 (0.05, 0.78)	NA
Li 2014 (54)	Exenatide	Acute pancreatitis	5	RR: 0.86 (0.22, 3.37)	0	2	RR: 0.92 (0.69, 1.22)	0
Li 2016 (53)	DDP-4 inhibitors	Heart failure	34	RR: 0.9 (0.61, 1.35)	0	4	RR: 1.1 (1.04, 1.16)	0
Li 2016 (53)	DDP-4 inhibitors	Hospital admission for heart failure	5	OR: 1.13 (1, 1.27)	0	6	OR: 0.85 (0.74, 0.97)	33
Matthews 2018 (86)	Tamoxifen	Heart failure	1	RR: 0.52 (0.33, 0.71)	NA	2	RR: 0.84 (0.65, 1.07)	10
Menne 2019 (87)	SGLT-2 inhibitors	Acute kidney injury	41	OR: 0.75 (0.66, 0.84)	0	5	OR: 0.4 (0.33, 0.48)	39
Mesgarpour 2017 (88)	Erythropoiesis stimulating agents	Venous thromboembolism	12	RR: 1.12 (0.9, 1.4)	9	5	RR: 1.87 (0.59, 5.92)	78
Mesgarpour 2017 (88)	Erythropoiesis stimulating agents	All-cause mortality	17	RR: 0.81 (0.71, 0.93)	0	7	RR: 1.07 (0.65, 1.77)	91
Moberley 2013 (89)	Pneumococcal polysaccharide vaccines	Invasive pneumococcal disease	10	OR: 0.26 (0.14, 0.45)	0	2	OR: 0.57 (0.36, 0.89)	0
Molnar 2015 (35)	Neoral (Cyclosporin)	Acute rejection of kidney transplant	2	OR: 1.23 (0.64, 2.36)	11	2	OR: 0.47 (0.27, 0.83)	0
Navarese 2013 (90)	Early intervention for NSTE-ACS	All-cause mortality	7	OR: 0.83 (0.64, 1.09)	0	4	OR: 0.8 (0.63, 1.02)	78

Navarese 2013 (90)	Early intervention for NSTE-ACS	Myocardial infarction	7	OR: 1.15 (0.65, 2.01)	81	3	OR: 0.86 (0.69, 1.08)	86
Navarese 2013 (90)	Early intervention for NSTE-ACS	Major bleeding	7	OR: 0.76 (0.56, 1.04)	0	3	OR: 1.12 (0.69, 1.82)	92
Nelson 2010 (36)	Caesarean section	Anal incontinence, feces	1	OR: 1 (0.49, 2.05)	NA	11	OR: 0.91 (0.72, 1.16)	0
Nelson 2010 (36)	Caesarean section	Anal incontinence, flatus	1	OR: 0.83 (0.51, 1.36)	NA	4	OR: 1.02 (0.87, 1.2)	0
Nieuwenhuijse 2014 (37)	Ceramic-on-ceramic bearings for total hip arthroplasty	Harris Hip Score	7	MD: -0.23 (-1.09, 0.63)	24	3	MD: -0.5 (-2.09, 1.09)	62
Nieuwenhuijse 2014 (37)	High-flexion total knee arthroplasty	Flexion	20	MD: 1.68 (0.28, 3.08)	45	26	MD: 3.78 (1.64, 5.92)	78
Nieuwenhuijse 2014 (37)	Gender-specific total knee arthroplasty	Flexion-extension range	6	MD: 1.41 (-0.17, 2.99)	6	2	MD: 3.15 (-0.03, 6.34)	29
Nikooie 2019 (55)	Second generation antipsychotics	Sedation	6	RR: 1.26 (0.92, 1.72)	0	3	RR: 1.84 (0.4, 8.54)	34
Nikooie 2019 (55)	Second generation antipsychotics	Neurologic outcomes	6	RR: 0.45 (0.2, 1.01)	0	5	RR: 0.76 (0.59, 0.99)	0
Ochen 2019 (91)	Surgery for achilles tendon rupture	Re-rupture	10	RR: 0.4 (0.24, 0.69)	0	18	RR: 0.42 (0.28, 0.64)	31
Ochen 2019 (91)	Surgery for achilles tendon rupture	Complications	9	RR: 3.26 (1.26, 8.41)	74	15	RR: 2.93 (2.28, 3.75)	0
Pittas 2010 (60)	Vitamin D	Hypertension	1	RR: 1.01 (0.97, 1.05)	NA	3	RR: 0.57 (0.41, 0.79)	0
Raman 2013 (38)	Carotid endarterectomy	Ipsilateral stroke	3	RR: 0.72 (0.58, 0.9)	0	2	RR: 0.47 (0.05, 4.46)	83
Raman 2013 (38)	Carotid endarterectomy	Stroke	3	RR: 0.68 (0.56, 0.82)	18	3	RR: 0.73 (0.43, 1.22)	0
Raman 2013 (38)	Carotid artery stenting	Periprocedural stroke	2	RR: 1.75 (0.87, 3.52)	0	5	RR: 1.91 (1.72, 2.11)	7
Schweizer 2013 (39)	Nasal deconolization	Surgical site infection	5	RR: 0.63 (0.36, 1.13)	50	6	RR: 0.4 (0.28, 0.57)	0
Schweizer 2013 (39)	Glycopeptide prophylaxis	Surgical site infection	8	RR: 1.13 (0.9, 1.42)	0	7	RR: 0.34 (0.11, 1.1)	83

Silvain 2012 (40)	Enoxaparin	All-cause mortality	6	RR: 0.88 (0.7, 1.1)	0	7	RR: 0.49 (0.39, 0.62)	2
Silvain 2012 (40)	Enoxaparin	Major bleeding	9	RR: 0.88 (0.62, 1.24)	53	7	RR: 0.72 (0.56, 0.93)	0
Silvain 2012 (40)	Enoxaparin	All-cause mortality or myocardial infarction	13	RR: 0.86 (0.74, 0.99)	21	7	RR: 0.44 (0.35, 0.55)	0
Suthar 2012 (26)	Antiretroviral therapy	Tuberculosis infection	2	HR: 0.5 (0.34, 0.75)	0	9	HR: 0.32 (0.25, 0.41)	27
Te Morenga 2013 (61)	Sugar	Weight gain	10	MD: 0.75 (0.3, 1.19)	82	4	MD: 0.31 (-0.07, 0.68)	99
Te Morenga 2013 (61)	Sugar	Body Mass Index	3	MD: -0.06 (-0.15, 0.04)	0	4	MD: 0.02 (0.00, 0,05)	75
Thomas 2010 (92)	Influenza vaccines	Influenza-like illness	3	RR: 0.71 (0.55, 0.9)	45	1	RR: 0.31 (0.26, 0.36)	NA
Tickell-Painter 2017 (93)	Mefloquine	Discontinuation due to adverse effects	3	RR: 2.86 (1.53, 5.31)	0	9	RR: 2.73 (1.83, 4.08)	33
Tickell-Painter 2017 (93)	Mefloquine	Serious adverse events or effects	3	RR: 0.7 (0.14, 3.53)	0	2	RR: 3.08 (0.39, 24.11)	0
Tickell-Painter 2017 (93)	Mefloquine	Nausea	2	RR: 1.35 (1.05, 1.73)	0	3	RR: 1.85 (1.42, 2.43)	0
Tricco 2018 (45)	Live-attenuated zoster vaccines	Suspected Herpes Zoster	5	RR: 0.61 (0.48, 0.93)	0	3	RR: 0.48 (0.27, 0.84)	99
Vinceti 2018 (59)	Selenium	Cancer	5	RR: 0.99 (0.86, 1.14)	46	7	RR: 0.75 (0.59, 0.94)	46
Vinceti 2018 (59)	Selenium	Cancer mortality	2	RR: 0.81 (0.49, 1.32)	79	7	RR: 0.77 (0.6, 0.97)	66
Vinceti 2018 (59)	Selenium	Colorectal cancer	3	RR: 0.74 (0.41, 1.33)	48	6	RR: 0.82 (0.72, 0.94)	0
Wilson 2011 (41)	Traditional birth attendants	Perinatal mortality	5	RR: 0.76 (0.64, 0.88)	66	1	RR: 0.82 (0.38, 1.78)	NA
Wilson 2011 (41)	Traditional birth attendants	Neonatal mortality	6	RR: 0.79 (0.69, 0.88)	41	2	RR: 0.8 (0.47, 1.37)	0
Wilson 2019 (42)	Unicompartimental knee arthroplasty	Venous thromboembolism	2	RR: 0.24 (0.04, 1.37)	0	8	RR: 0.41 (0.29, 0.57)	30

Wilson 2019 (42)	Unicompartimental knee arthroplasty	Flexion-extension range	3	MD: -4.58 (-10.75, 1.59)	95	11	MD: -8.43 (-10.15, -6.71)	86
Wilson 2019 (42)	Unicompartimental knee arthroplasty	Operation duration	3	MD: -1.72 (-11.89, 8.45)	90	8	MD: -23.8 (-40.43, -7.17)	99
Yank 2011 (44)	Recombinant factor VII	All-cause mortality	2	RR: 1.4 (0.49, 4.02)	0	2	RR: 0.91 (0.39, 2.12)	0
Yank 2011 (44)	Recombinant factor VII	Thromboembolism	2	RR: 2.06 (0.48, 8.84)	16	2	RR: 1.81 (0.67, 4.87)	0
Zhang 2016 (94)	Everolimus-eluting bioresorbable vascular scaffold	Stent thrombosis	5	OR: 2.05 (0.95, 4.43)	0	3	OR: 2.32 (1.06, 5.07)	0
Zhang 2016 (94)	Everolimus-eluting bioresorbable vascular scaffold	All-cause mortality	5	OR: 0.96 (0.46, 2)	45	4	OR: 0.57 (0.23, 1.44)	0
Zhang 2016 (94)	Everolimus-eluting bioresorbable vascular scaffold	Coronary heart disease mortality	3	OR: 1.4 (0.45, 4.33)	44	4	OR: 0.81 (0.38, 1.7)	0
Zhang 2017 (95)	Percutaneous coronary intervention	All-cause mortality	5	HR: 1 (0.79, 1.26)	22	17	HR: 1.08 (0.92, 1.26)	37
Zhang 2017 (95)	Percutaneous coronary intervention	Cardiovascular mortality	4	HR: 1 (0.72, 1.39)	21	5	HR: 1.08 (0.51, 2.29)	78
Zhang 2017 (95)	Percutaneous coronary intervention	Myocardial infarction	5	HR: 1.39 (0.85, 2.27)	58	5	HR: 2.01 (1.64, 2.45)	0
Ziff 2015 (96)	Digoxin	All-cause mortality	7	RR: 0.99 (0.93, 1.05)	0	8	RR: 1.61 (1.31, 1.97)	65
Ziff 2015 (96)	Digoxin	Cardiovascular mortality	5	RR: 1.01 (0.94, 1.08)	0	3	RR: 2.53 (1.12, 5.71)	96
Ziff 2015 (96)	Digoxin	Hospital admission	2	RR: 0.94 (0.9, 0.99)	65	4	RR: 0.91 (0.87, 0.95)	64

DDP-4= dipeptidyl peptidase 4; DHA= docosahexaenoic acid; EPA= eicosapentaenoic acid; NA= not applicable; NR= not reported; NSTE-ACS= non-ST elevation acute coronary syndrome; SGLT-2= sodium glucose transporter 2.

Figures S2a to S7: Forest Plots

Systematic review, year	Intervention/Exposure	Outcome	RRR/ ROR/ RHR	95%-CI	Weight
**similar but not identical**					
Abou-Setta, 2011	Nerve block	Delirium	1.38	[0.37; 5.08]	0.2%
Abou-Setta, 2011	Spinal anesthesia	All-cause mortality	1.99	[0.51; 7.71]	0.2%
Aburto, 2013	Sodium	All-cause mortality	0.74	[0.46; 1.22]	0.9%
Aburto, 2013	Sodium	Cardiovascular disease	0.94	[0.62; 1.44]	1.0%
Alexander, 2017	DHA and EPA	Coronary heart disease	1.15	[0.98; 1.33]	1.6%
Alexander, 2017	DHA and EPA	Coronary heart disease mortality	1.30	[1.07; 1.57]	1.6%
Alexander, 2017	DHA and EPA	Coronary heart disease incidence	1.14	[0.75; 1.73]	1.0%
Anglemyer, 2013	Antiretroviral therapy	HIV infection	0.19	[0.06; 0.60]	0.3%
Barnard, 2015	Surgical abortion by mid-level providers	Failure or incomplete abortion	1.20	[0.08; 17.90]	0.1%
Barnard, 2015	Surgical abortion by mid-level providers	Complications	0.76	[0.11; 5.30]	0.1%
Barnard, 2015	Surgical abortion by mid-level providers	Abortion failure and complications	2.31	[0.11; 46.53]	0.0%
Bellemain-Appaix, 2012	Clopidogrel	All-cause mortality	1.01	[0.59; 1.73]	0.8%
Bellemain-Appaix, 2012	Clopidogrel	Major bleeding	1.02	[0.68; 1.53]	1.0%
Bellemain-Appaix, 2012	Clopidogrel	Coronary heart disease	1.01	[0.77; 1.33]	1.4%
Bellemain-Appaix, 2014	P2Y12 inhibitors	All-cause mortality	1.33	[0.51; 3.51]	0.4%
Bellemain-Appaix, 2014	P2Y12 inhibitors	Major bleeding	1.29	[0.81; 2.08]	0.9%
Bellemain-Appaix, 2014	P2Y12 inhibitors	Main composite ischemic endpoint	1.08	[0.69; 1.68]	1.0%
Bloomfield, 2016	Mediterranean diet	Breast cancer	0.55	[0.29; 1.06]	0.6%
Chowdhury, 2012	Omega-3	Cerebrovascular disease	1.09	[0.94; 1.27]	1.6%
Chowdhury, 2014b	Vitamin D	All-cause mortality	1.42	[1.31; 1.54]	1.7%
Chung, 2011	Vitamin D	Colorectal cancer	1.09	[0.64; 1.85]	0.8%
Chung, 2011	Vitamin D	Breast cancer	1.00	[0.25; 4.00]	0.2%
Chung, 2016	Calcium	Cardiovascular mortality	1.06	[0.83; 1.35]	1.4%
Fluri, 2010	Extracranial-intracranial arterial bypass	All-cause mortality	0.81	[0.47; 1.40]	0.8%
Fluri, 2010	Extracranial-intracranial arterial bypass	Stroke	1.24	[0.79; 1.94]	1.0%
Gargiulo, 2016	Transcatheter aortic valve	Early all-cause mortality	0.74	[0.44; 1.24]	0.8%
Gargiulo, 2016	Transcatheter aortic valve	Mid-term all-cause mortality	0.90	[0.60; 1.34]	1.1%
Gargiulo, 2016	Transcatheter aortic valve	Long-term all-cause mortality	0.61	[0.35; 1.06]	0.8%
Hartling, 2013	Treating gestational diabetes mellitus	High birth weight	0.72	[0.30; 1.74]	0.4%
Hartling, 2013	Treating gestational diabetes mellitus	Large-for-gestational age neonate	1.30	[0.77; 2.20]	0.8%
Hartling, 2013	Treating gestational diabetes mellitus	Shoulder dystocia	1.11	[0.44; 2.80]	0.4%
Henderson, 2019	Treating asymptomatic bacteriuria	Pyelonephritis	0.83	[0.35; 1.93]	0.4%
Hopley, 2010	Total hip arthroplasty	Reoperation	2.42	[0.63; 9.34]	0.2%
Hopley, 2010	Total hip arthroplasty	Dislocation	3.09	[0.58; 16.47]	0.1%
Hopley, 2010	Total hip arthroplasty	Deep infection	1.88	[0.38; 9.33]	0.2%
Jamal, 2013	Non-calcium-based phosphat binders	All-cause mortality	0.88	[0.67; 1.15]	1.4%
Jefferson, 2010	Parenteral influenza vaccine	Influenza-like illness	0.78	[0.60; 1.01]	1.4%
Jefferson, 2010	Parenteral influenza vaccine	Influenza	0.84	[0.38; 1.86]	0.5%
Johnston, 2019	Red meat	All-cause mortality	1.08	[1.00; 1.17]	1.7%
Johnston, 2019	Red meat	Cardiovascular mortality	1.16	[0.96; 1.41]	1.5%
Johnston, 2019	Red meat	Cardiovascular disease	1.11	[0.95; 1.31]	1.6%
Kansagara, 2013	Transfusion	All-cause mortality	0.38	[0.18; 0.77]	0.6%
Kredo, 2014	Starting and maintaining antiretroviral therapy	All-cause mortality	0.78	[0.66; 0.93]	1.6%
Kredo, 2014	Starting and maintaining antiretroviral therapy	Attrition	2.43	[0.38; 15.49]	0.1%
Kredo, 2014	Maintaining antiretroviral therapy	All-cause mortality	4.68	[1.12; 19.60]	0.2%
Li, 2014	Exenatide	Acute pancreatitis	0.93	[0.23; 3.77]	0.2%
Li, 2016	DDP-4 inhibitors	Heart failure	0.82	[0.55; 1.22]	1.1%
Li, 2016	DDP-4 inhibitors	Hospital admission for heart failure	1.33	[1.11; 1.59]	1.6%
Matthews, 2018	Tamoxifen	Heart failure	0.62	[0.39; 0.98]	1.0%
Menne, 2019	SGLT-2 inhibitors	Acute kidney injury	1.88	[1.50; 2.34]	1.5%
Mesgarpour, 2017	Erythropoiesis stimulating agents	Venous thromboembolism	0.60	[0.19; 1.94]	0.3%
Mesgarpour, 2017	Erythropoiesis stimulating agents	All-cause mortality	0.76	[0.45; 1.27]	0.8%
Moberley, 2013	Pneumococcal polysaccharide vaccines	Invasive pneumococcal disease	0.46	[0.22; 0.95]	0.5%
Molnar, 2015	Neoral (Cyclosporin)	Acute rejection of kidney transplant	2.62	[1.11; 6.19]	0.4%
Navarese, 2013	Early intervention for NSTE-ACS	All-cause mortality	1.04	[0.72; 1.49]	1.2%
Navarese, 2013	Early intervention for NSTE-ACS	Myocardial infarction	1.34	[0.73; 2.45]	0.7%
Navarese, 2013	Early intervention for NSTE-ACS	Major bleeding	0.68	[0.38; 1.21]	0.7%
Nikooie, 2019	Second generation antipsychotics	Sedation	0.68	[0.14; 3.27]	0.2%
Nikooie, 2019	Second generation antipsychotics	Neurologic outcomes	0.59	[0.25; 1.39]	0.4%
Pittas, 2010	Vitamin D	Hypertension	1.77	[1.27; 2.47]	1.2%
Raman, 2013	Carotid endarterectomy	Ipsilateral stroke	1.53	[0.16; 14.62]	0.1%
Raman, 2013	Carotid endarterectomy	Stroke	0.93	[0.53; 1.62]	0.8%
Raman, 2013	Carotid artery stenting	Periprocedural stroke	0.92	[0.45; 1.86]	0.6%
Schweizer, 2013	Nasal deconolization	Surgical site infection	1.57	[0.80; 3.09]	0.6%
Schweizer, 2013	Glycopeptide prophylaxis	Surgical site infection	3.32	[1.03; 10.75]	0.3%
Silvain, 2012	Enoxaparin	All-cause mortality	1.80	[1.30; 2.48]	1.2%
Silvain, 2012	Enoxaparin	Major bleeding	1.22	[0.80; 1.88]	1.0%
Silvain, 2012	Enoxaparin	All-cause mortality or myocardial infarction	1.95	[1.49; 2.56]	1.4%
Suthar, 2012	Antiretroviral therapy	Tuberculosis infection	1.56	[0.98; 2.49]	0.9%
Thomas, 2010	Influenza vaccines	Influenza-like illness	2.29	[1.70; 3.08]	1.3%
Tickell-Painter, 2017	Mefloquine	Discontinuation due to adverse effects	1.05	[0.50; 2.20]	0.5%
Tricco, 2018	Live-attenuated zoster vaccines	Suspected Herpes Zoster	1.27	[0.66; 2.45]	0.6%
Vinceti, 2018	Selenium	Cancer	1.32	[1.01; 1.73]	1.4%
Vinceti, 2018	Selenium	Cancer mortality	1.05	[0.61; 1.82]	0.8%
Vinceti, 2018	Selenium	Colorectal cancer	0.90	[0.49; 1.65]	0.7%
Wilson, 2019	Unicompartimental knee arthroplasty	Venous thromboembolism	0.59	[0.10; 3.54]	0.1%
Yank, 2011	Recombinant factor VII	All-cause mortality	1.54	[0.40; 5.94]	0.2%
Yank, 2011	Recombinant factor VII	Thromboembolism	1.14	[0.20; 6.63]	0.1%
Zhang, 2016	Everolimus-eluting bioresorbable vascular scaffold	Stent thrombosis	0.88	[0.29; 2.65]	0.3%
Zhang, 2016	Everolimus-eluting bioresorbable vascular scaffold	All-cause mortality	1.68	[0.52; 5.45]	0.3%
Zhang, 2016	Everolimus-eluting bioresorbable vascular scaffold	Coronary heart disease mortality	1.73	[0.44; 6.72]	0.2%
Zhang, 2017	Percutaneous coronary intervention	All-cause mortality	0.93	[0.70; 1.23]	1.3%
Zhang, 2017	Percutaneous coronary intervention	Cardiovascular mortality	0.93	[0.41; 2.10]	0.5%
Zhang, 2017	Percutaneous coronary intervention	Myocardial infarction	0.69	[0.41; 1.18]	0.8%
Ziff, 2015	Digoxin	Hospital admission	1.03	[0.97; 1.10]	1.8%
Random effects model			1.08	[1.00; 1.17]	66.1%
Prediction interval				[0.66; 1.79]	--
Heterogeneity: $I^2 = 65\%$, $\tau^2 = 0.0616$, $p < 0.01$					
**more or less identical**					
Ahmad, 2015	Intra-aortic balloon pump	All-cause mortality	0.94	[0.50; 1.78]	0.7%
Bolland, 2015	Calcium	All fractures	0.88	[0.78; 0.99]	1.7%
Bolland, 2015	Calcium	Vertebral fracture	0.61	[0.45; 0.84]	1.3%
Bolland, 2015	Calcium	Hip fracture	0.87	[0.66; 1.16]	1.3%
Brenner, 2014	Sigmoidoscopy	Colorectal cancer mortality	1.22	[0.92; 1.62]	1.3%
Brenner, 2014	Sigmoidoscopy	Colorectal cancer incidence	1.64	[1.19; 2.27]	1.2%
Fenton, 2018	Radiation therapy	Erectile dysfunction	0.70	[0.58; 0.85]	1.5%
Fenton, 2018	Radical Prostatectomy	Urinary incontinence	0.78	[0.46; 1.32]	0.8%
Fenton, 2018	Radical Prostatectomy	Erectile dysfunction	1.07	[0.81; 1.42]	1.3%
Filippini, 2017	Disease-modifying drugs	Conversion to clinically definite multiple sclerosis	1.08	[0.66; 1.78]	0.9%
Fluri, 2010	Extracranial-intracranial arterial bypass	Stroke mortality or dependency	1.17	[0.69; 2.00]	0.8%
Hüpfl, 2010	Chest-compression-only cardiopulmonary resuscitation	All-cause mortality	0.79	[0.62; 1.00]	1.4%
Jefferson, 2012	Inactivated influenza vaccines	Influenza	2.05	[0.95; 4.42]	0.5%
Jefferson, 2012	Inactivated influenza vaccines	Influenza-like illness	2.21	[0.54; 9.04]	0.2%
Ochen, 2019	Surgery for achilles tendon rupture	Re-rupture	0.95	[0.49; 1.86]	0.6%
Ochen, 2019	Surgery for achilles tendon rupture	Complications	1.11	[0.42; 2.97]	0.4%
Wilson, 2011	Traditional birth attendants	Perinatal mortality	0.93	[0.42; 2.04]	0.5%
Wilson, 2011	Traditional birth attendants	Neonatal mortality	0.99	[0.57; 1.71]	0.8%
Random effects model			0.96	[0.84; 1.10]	17.3%
Prediction interval				[0.62; 1.49]	--
Heterogeneity: $I^2 = 59\%$, $\tau^2 = 0.0387$, $p < 0.01$					
**broadly similar**					
Alipanah, 2018	Self-administered therapy	Low treatment success	0.85	[0.75; 0.98]	1.7%
Alipanah, 2018	Self-administered therapy	Low treatment completion	1.40	[0.94; 2.08]	1.1%
Alipanah, 2018	Self-administered therapy	All-cause mortality	0.54	[0.30; 0.96]	0.7%
Chowdhury, 2014a	α-linolenic acid	Coronary heart disease	0.98	[0.68; 1.41]	1.1%
Chowdhury, 2014a	Omega-3	Coronary heart disease	1.08	[0.94; 1.24]	1.7%
Chowdhury, 2014a	Omega-6	Coronary heart disease	0.88	[0.69; 1.11]	1.5%
Higgins, 2016	Bacillus Calmette-Guérin	All-cause mortality	1.46	[0.75; 2.84]	0.6%
Higgins, 2016	Measles containing vaccines	All-cause mortality	1.40	[0.88; 2.22]	0.9%
Jin, 2012	Total flavonoids	Colorectal neoplasms	1.09	[0.83; 1.43]	1.4%
Keag, 2018	Caesarean section	Urinary incontinence	1.39	[0.96; 2.02]	1.1%
Keag, 2018	Caesarean section	Fecal incontinence	2.95	[0.82; 10.59]	0.2%
Nelson, 2010	Caesarean section	Anal incontinence, feces	1.10	[0.52; 2.34]	0.5%
Nelson, 2010	Caesarean section	Anal incontinence, flatus	0.81	[0.49; 1.36]	0.8%
Tickell-Painter, 2017	Mefloquine	Serious adverse events or effects	0.23	[0.02; 3.12]	0.1%
Tickell-Painter, 2017	Mefloquine	Nausea	0.73	[0.51; 1.05]	1.1%
Ziff, 2015	Digoxin	All-cause mortality	0.61	[0.50; 0.76]	1.5%
Ziff, 2015	Digoxin	Cardiovascular mortality	0.40	[0.18; 0.90]	0.5%
Random effects model			0.95	[0.79; 1.13]	16.5%
Prediction interval				[0.49; 1.82]	--
Heterogeneity: $I^2 = 67\%$, $\tau^2 = 0.0857$, $p < 0.01$					
**Random effects model**			**1.04**	**[0.97; 1.11]**	**100.0%**
**Prediction interval**				**[0.63; 1.71]**	
Heterogeneity: $I^2 = 69\%$, $\tau^2 = 0.0614$, $p < 0.01$					

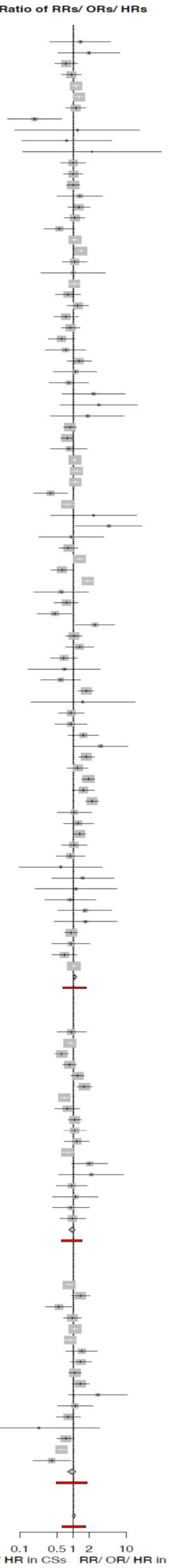

Figure S2a. Forest plot for binary outcomes, pooled ratio of ratios (RoR) for bodies of evidence from randomized controlled trials vs. cohort studies stratified by population similarity degree.

CSs= cohort studies; DDP-4= dipeptidyl peptidase 4; DHA= docosahexaenoic acid; EPA= eicosapentaenoic acid; HR= hazard ratio; NSTE-ACS= non-ST elevation acute coronary syndrome; OR= odds ratio; RCTs= randomized controlled trials; RHR= ratio of hazard ratios; ROR= ratio of odds ratios; RR= risk ratio; RRR= ratio of risk ratios; SGLT-2= sodium glucose transporter 2.

Systematic review, year	Intervention/Exposure	Outcome	RRR/ ROR/ RHR	95%-CI	Weight
**similar but not identical**					
Abou-Setta, 2011	Nerve block	Delirium	1.38	[0.37; 5.08]	0.2%
Aburto, 2013	Sodium	All-cause mortality	0.74	[0.46; 1.22]	0.9%
Aburto, 2013	Sodium	Cardiovascular disease	0.94	[0.62; 1.44]	1.0%
Alexander, 2017	DHA and EPA	Coronary heart disease	1.15	[0.98; 1.33]	1.6%
Alexander, 2017	DHA and EPA	Coronary heart disease mortality	1.30	[1.07; 1.57]	1.6%
Alexander, 2017	DHA and EPA	Coronary heart disease incidence	1.14	[0.75; 1.73]	1.0%
Alipanah, 2018	Self-administered therapy	Low treatment success	0.85	[0.75; 0.98]	1.7%
Alipanah, 2018	Self-administered therapy	Low treatment completion	1.40	[0.94; 2.08]	1.1%
Alipanah, 2018	Self-administered therapy	All-cause mortality	0.54	[0.30; 0.96]	0.7%
Anglemyer, 2013	Antiretroviral therapy	HIV infection	0.19	[0.06; 0.60]	0.3%
Barnard, 2015	Surgical abortion by mid-level providers	Failure or incomplete abortion	1.20	[0.08; 17.90]	0.1%
Barnard, 2015	Surgical abortion by mid-level providers	Complications	0.76	[0.11; 5.30]	0.1%
Barnard, 2015	Surgical abortion by mid-level providers	Abortion failure and complications	2.31	[0.11; 46.53]	0.0%
Bellemain-Appaix, 2012	Clopidogrel	All-cause mortality	1.01	[0.59; 1.73]	0.8%
Bellemain-Appaix, 2012	Clopidogrel	Major bleeding	1.02	[0.68; 1.53]	1.0%
Bellemain-Appaix, 2012	Clopidogrel	Coronary heart disease	1.01	[0.77; 1.33]	1.4%
Bellemain-Appaix, 2014	P2Y12 inhibitors	All-cause mortality	1.33	[0.51; 3.51]	0.4%
Bellemain-Appaix, 2014	P2Y12 inhibitors	Major bleeding	1.29	[0.81; 2.08]	0.9%
Bellemain-Appaix, 2014	P2Y12 inhibitors	Main composite ischemic endpoint	1.08	[0.69; 1.68]	1.0%
Bloomfield, 2016	Mediterranean diet	Breast cancer	0.55	[0.29; 1.06]	0.6%
Bolland, 2015	Calcium	All fractures	0.88	[0.78; 0.99]	1.7%
Bolland, 2015	Calcium	Vertebral fracture	0.61	[0.45; 0.84]	1.3%
Bolland, 2015	Calcium	Hip fracture	0.87	[0.66; 1.16]	1.3%
Chowdhury, 2012	Omega-3	Cerebrovascular disease	1.09	[0.94; 1.27]	1.6%
Chowdhury, 2014a	α-linolenic acid	Coronary heart disease	0.98	[0.68; 1.41]	1.1%
Chowdhury, 2014a	Omega-3	Coronary heart disease	1.08	[0.94; 1.24]	1.7%
Chowdhury, 2014a	Omega-6	Coronary heart disease	0.88	[0.69; 1.11]	1.5%
Chung, 2016	Calcium	Cardiovascular mortality	1.06	[0.83; 1.35]	1.4%
Filippini, 2017	Disease-modifying drugs	Conversion to clinically definite multiple sclerosis	1.08	[0.66; 1.78]	0.9%
Gargiulo, 2016	Transcatheter aortic valve	Early all-cause mortality	0.74	[0.44; 1.24]	0.8%
Gargiulo, 2016	Transcatheter aortic valve	Mid-term all-cause mortality	0.90	[0.60; 1.34]	1.1%
Henderson, 2019	Treating asymptomatic bacteriuria	Pyelonephritis	0.83	[0.35; 1.93]	0.4%
Higgins, 2016	Measles containing vaccines	All-cause mortality	1.40	[0.88; 2.22]	0.9%
Hopley, 2010	Total hip arthroplasty	Deep infection	1.88	[0.38; 9.33]	0.2%
Jamal, 2013	Non-calcium-based phosphat binders	All-cause mortality	0.88	[0.67; 1.15]	1.4%
Jefferson, 2010	Parenteral influenza vaccine	Influenza-like illness	0.78	[0.60; 1.01]	1.4%
Jefferson, 2010	Parenteral influenza vaccine	Influenza	0.84	[0.38; 1.86]	0.5%
Jefferson, 2012	Inactivated influenza vaccines	Influenza	2.05	[0.95; 4.42]	0.5%
Jefferson, 2012	Inactivated influenza vaccines	Influenza-like illness	2.21	[0.54; 9.04]	0.2%
Jin, 2012	Total flavonoids	Colorectal neoplasms	1.09	[0.83; 1.43]	1.4%
Johnston, 2019	Red meat	All-cause mortality	1.08	[1.00; 1.17]	1.7%
Johnston, 2019	Red meat	Cardiovascular mortality	1.16	[0.96; 1.41]	1.5%
Johnston, 2019	Red meat	Cardiovascular disease	1.11	[0.95; 1.31]	1.6%
Keag, 2018	Caesarean section	Urinary incontinence	1.39	[0.96; 2.02]	1.1%
Keag, 2018	Caesarean section	Fecal incontinence	2.95	[0.82; 10.59]	0.2%
Li, 2016	DDP-4 inhibitors	Heart failure	0.82	[0.55; 1.22]	1.1%
Li, 2016	DDP-4 inhibitors	Hospital admission for heart failure	1.33	[1.11; 1.59]	1.6%
Menne, 2019	SGLT-2 inhibitors	Acute kidney injury	1.88	[1.50; 2.34]	1.5%
Mesgarpour, 2017	Erythropoiesis stimulating agents	Venous thromboembolism	0.60	[0.19; 1.94]	0.3%
Mesgarpour, 2017	Erythropoiesis stimulating agents	All-cause mortality	0.76	[0.45; 1.27]	0.8%
Moberley, 2013	Pneumococcal polysaccharide vaccines	Invasive pneumococcal disease	0.46	[0.22; 0.95]	0.5%
Navarese, 2013	Early intervention for NSTE-ACS	All-cause mortality	1.04	[0.72; 1.49]	1.2%
Navarese, 2013	Early intervention for NSTE-ACS	Myocardial infarction	1.34	[0.73; 2.45]	0.7%
Navarese, 2013	Early intervention for NSTE-ACS	Major bleeding	0.68	[0.38; 1.21]	0.7%
Nelson, 2010	Caesarean section	Anal incontinence, feces	1.10	[0.52; 2.34]	0.5%
Nelson, 2010	Caesarean section	Anal incontinence, flatus	0.81	[0.49; 1.36]	0.8%
Nikooie, 2019	Second generation antipsychotics	Sedation	0.68	[0.14; 3.27]	0.2%
Nikooie, 2019	Second generation antipsychotics	Neurologic outcomes	0.59	[0.25; 1.39]	0.4%
Ochen, 2019	Surgery for achilles tendon rupture	Re-rupture	0.95	[0.49; 1.86]	0.6%
Ochen, 2019	Surgery for achilles tendon rupture	Complications	1.11	[0.42; 2.97]	0.4%
Raman, 2013	Carotid artery stenting	Periprocedural stroke	0.92	[0.45; 1.86]	0.6%
Schweizer, 2013	Nasal deconolization	Surgical site infection	1.57	[0.80; 3.09]	0.6%
Schweizer, 2013	Glycopeptide prophylaxis	Surgical site infection	3.32	[1.03; 10.75]	0.3%
Tricco, 2018	Live-attenuated zoster vaccines	Suspected Herpes Zoster	1.27	[0.66; 2.45]	0.6%
Wilson, 2011	Traditional birth attendants	Perinatal mortality	0.93	[0.42; 2.04]	0.5%
Wilson, 2011	Traditional birth attendants	Neonatal mortality	0.99	[0.57; 1.71]	0.8%
Yank, 2011	Recombinant factor VII	All-cause mortality	1.54	[0.40; 5.94]	0.2%
Yank, 2011	Recombinant factor VII	Thromboembolism	1.14	[0.20; 6.63]	0.1%
Zhang, 2017	Percutaneous coronary intervention	All-cause mortality	0.93	[0.70; 1.23]	1.3%
Zhang, 2017	Percutaneous coronary intervention	Cardiovascular mortality	0.93	[0.41; 2.10]	0.5%
Zhang, 2017	Percutaneous coronary intervention	Myocardial infarction	0.69	[0.41; 1.18]	0.8%
Random effects model			1.01	[0.95; 1.08]	61.5%
Prediction interval				[0.72; 1.43]	--
Heterogeneity: $I^2 = 50\%$, $\tau^2 = 0.0290$, $p < 0.01$					
**more or less identical**					
Abou-Setta, 2011	Spinal anesthesia	All-cause mortality	1.99	[0.51; 7.71]	0.2%
Ahmad, 2015	Intra-aortic balloon pump	All-cause mortality	0.94	[0.50; 1.78]	0.7%
Brenner, 2014	Sigmoidoscopy	Colorectal cancer mortality	1.22	[0.92; 1.62]	1.3%
Brenner, 2014	Sigmoidoscopy	Colorectal cancer incidence	1.64	[1.19; 2.27]	1.2%
Fenton, 2018	Radiation therapy	Erectile dysfunction	0.70	[0.58; 0.85]	1.5%
Fenton, 2018	Radical Prostatectomy	Urinary incontinence	0.78	[0.46; 1.32]	0.8%
Fenton, 2018	Radical Prostatectomy	Erectile dysfunction	1.07	[0.81; 1.42]	1.3%
Fluri, 2010	Extracranial-intracranial arterial bypass	All-cause mortality	0.81	[0.47; 1.40]	0.8%
Fluri, 2010	Extracranial-intracranial arterial bypass	Stroke	1.24	[0.79; 1.94]	1.0%
Fluri, 2010	Extracranial-intracranial arterial bypass	Stroke mortality or dependency	1.17	[0.69; 2.00]	0.8%
Gargiulo, 2016	Transcatheter aortic valve	Long-term all-cause mortality	0.61	[0.35; 1.06]	0.8%
Hartling, 2013	Treating gestational diabetes mellitus	High birth weight	0.72	[0.30; 1.74]	0.4%
Hartling, 2013	Treating gestational diabetes mellitus	Large-for-gestational age neonate	1.30	[0.77; 2.20]	0.8%
Hartling, 2013	Treating gestational diabetes mellitus	Shoulder dystocia	1.11	[0.44; 2.80]	0.4%
Higgins, 2016	Bacillus Calmette-Guérin	All-cause mortality	1.46	[0.75; 2.84]	0.6%
Hopley, 2010	Total hip arthroplasty	Reoperation	2.42	[0.63; 9.34]	0.2%
Hopley, 2010	Total hip arthroplasty	Dislocation	3.09	[0.58; 16.47]	0.1%
Li, 2014	Exenatide	Acute pancreatitis	0.93	[0.23; 3.77]	0.2%
Molnar, 2015	Neoral (Cyclosporin)	Acute rejection of kidney transplant	2.62	[1.11; 6.19]	0.4%
Raman, 2013	Carotid endarterectomy	Ipsilateral stroke	1.53	[0.16; 14.62]	0.1%
Raman, 2013	Carotid endarterectomy	Stroke	0.93	[0.53; 1.62]	0.8%
Silvain, 2012	Enoxaparin	All-cause mortality	1.80	[1.30; 2.48]	1.2%
Silvain, 2012	Enoxaparin	Major bleeding	1.22	[0.80; 1.88]	1.0%
Silvain, 2012	Enoxaparin	All-cause mortality or myocardial infarction	1.95	[1.49; 2.56]	1.4%
Tickell-Painter, 2017	Mefloquine	Discontinuation due to adverse effects	1.05	[0.50; 2.20]	0.5%
Tickell-Painter, 2017	Mefloquine	Serious adverse events or effects	0.23	[0.02; 3.12]	0.1%
Tickell-Painter, 2017	Mefloquine	Nausea	0.73	[0.51; 1.05]	1.1%
Wilson, 2019	Unicompartimental knee arthroplasty	Venous thromboembolism	0.59	[0.10; 3.54]	0.1%
Zhang, 2016	Everolimus-eluting bioresorbable vascular scaffold	Stent thrombosis	0.88	[0.29; 2.65]	0.3%
Zhang, 2016	Everolimus-eluting bioresorbable vascular scaffold	All-cause mortality	1.68	[0.52; 5.45]	0.3%
Zhang, 2016	Everolimus-eluting bioresorbable vascular scaffold	Coronary heart disease mortality	1.73	[0.44; 6.72]	0.2%
Ziff, 2015	Digoxin	All-cause mortality	0.61	[0.50; 0.76]	1.5%
Ziff, 2015	Digoxin	Cardiovascular mortality	0.40	[0.18; 0.90]	0.5%
Ziff, 2015	Digoxin	Hospital admission	1.03	[0.97; 1.10]	1.8%
Random effects model			1.07	[0.93; 1.23]	24.5%
Prediction interval				[0.60; 1.93]	--
Heterogeneity: $I^2 = 70\%$, $\tau^2 = 0.0777$, $p < 0.01$					
**broadly similar**					
Chowdhury, 2014b	Vitamin D	All-cause mortality	1.42	[1.31; 1.54]	1.7%
Chung, 2011	Vitamin D	Colorectal cancer	1.09	[0.64; 1.85]	0.8%
Chung, 2011	Vitamin D	Breast cancer	1.00	[0.25; 4.00]	0.2%
Hüpfl, 2010	Chest-compression-only cardiopulmonary resuscitation	All-cause mortality	0.79	[0.62; 1.00]	1.4%
Kansagara, 2013	Transfusion	All-cause mortality	0.38	[0.18; 0.77]	0.6%
Kredo, 2014	Starting and maintaining antiretroviral therapy	All-cause mortality	0.78	[0.66; 0.93]	1.6%
Kredo, 2014	Starting and maintaining antiretroviral therapy	Attrition	2.43	[0.38; 15.49]	0.1%
Kredo, 2014	Maintaining antiretroviral therapy	All-cause mortality	4.68	[1.12; 19.60]	0.2%
Matthews, 2018	Tamoxifen	Heart failure	0.62	[0.39; 0.98]	1.0%
Pittas, 2010	Vitamin D	Hypertension	1.77	[1.27; 2.47]	1.2%
Suthar, 2012	Antiretroviral therapy	Tuberculosis infection	1.56	[0.98; 2.49]	0.9%
Thomas, 2010	Influenza vaccines	Influenza-like illness	2.29	[1.70; 3.08]	1.3%
Vinceti, 2018	Selenium	Cancer	1.32	[1.01; 1.73]	1.4%
Vinceti, 2018	Selenium	Cancer mortality	1.05	[0.61; 1.82]	0.8%
Vinceti, 2018	Selenium	Colorectal cancer	0.90	[0.49; 1.65]	0.7%
Random effects model			1.14	[0.87; 1.49]	13.9%
Prediction interval				[0.42; 3.08]	--
Heterogeneity: $I^2 = 86\%$, $\tau^2 = 0.1937$, $p < 0.01$					
**Random effects model**			**1.04**	**[0.97; 1.11]**	**100.0%**
**Prediction interval**				**[0.63; 1.71]**	
Heterogeneity: $I^2 = 69\%$, $\tau^2 = 0.0614$, $p < 0.01$					

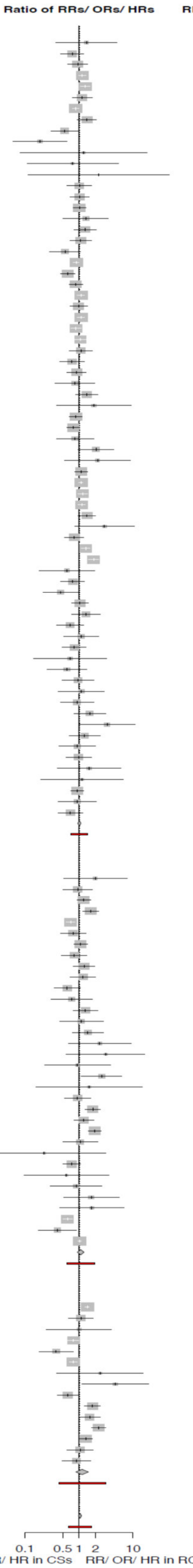

Figure S2b. Forest plot for binary outcomes, pooled ratio of ratios (RoR) for bodies of evidence from randomized controlled trials vs. cohort studies stratified by intervention/ exposure similarity degree.

CSs= cohort studies; DDP-4= dipeptidyl peptidase 4; DHA= docosahexaenoic acid; EPA= eicosapentaenoic acid; HR= hazard ratio; NSTE-ACS= non-ST elevation acute coronary syndrome; OR= odds ratio; RCTs= randomized controlled trials; RHR= ratio of hazard ratios; ROR= ratio of odds ratios; RR= risk ratio; RRR= ratio of risk ratios; SGLT-2= sodium glucose transporter 2.

Systematic review, year	Intervention/Exposure	Outcome	RRR/ ROR/ RHR	95%-CI	Weight
**similar but not identical**					
Abou-Setta, 2011	Nerve block	Delirium	1.38	[0.37; 5.08]	0.2%
Aburto, 2013	Sodium	All-cause mortality	0.74	[0.46; 1.22]	0.9%
Aburto, 2013	Sodium	Cardiovascular disease	0.94	[0.62; 1.44]	1.0%
Alexander, 2017	DHA and EPA	Coronary heart disease	1.15	[0.98; 1.33]	1.6%
Alexander, 2017	DHA and EPA	Coronary heart disease mortality	1.30	[1.07; 1.57]	1.6%
Alexander, 2017	DHA and EPA	Coronary heart disease incidence	1.14	[0.75; 1.73]	1.0%
Bellemain-Appaix, 2012	Clopidogrel	All-cause mortality	1.01	[0.59; 1.73]	0.8%
Bellemain-Appaix, 2012	Clopidogrel	Major bleeding	1.02	[0.68; 1.53]	1.0%
Bellemain-Appaix, 2012	Clopidogrel	Coronary heart disease	1.01	[0.77; 1.33]	1.4%
Bellemain-Appaix, 2014	P2Y12 inhibitors	All-cause mortality	1.33	[0.51; 3.51]	0.4%
Bellemain-Appaix, 2014	P2Y12 inhibitors	Major bleeding	1.29	[0.81; 2.08]	0.9%
Bellemain-Appaix, 2014	P2Y12 inhibitors	Main composite ischemic endpoint	1.08	[0.69; 1.68]	1.0%
Bloomfield, 2016	Mediterranean diet	Breast cancer	0.55	[0.29; 1.06]	0.6%
Chowdhury, 2014a	α-linolenic acid	Coronary heart disease	0.98	[0.68; 1.41]	1.1%
Chowdhury, 2014a	Omega-3	Coronary heart disease	1.08	[0.94; 1.24]	1.7%
Chowdhury, 2014a	Omega-6	Coronary heart disease	0.88	[0.69; 1.11]	1.5%
Fenton, 2018	Radiation therapy	Erectile dysfunction	0.70	[0.58; 0.85]	1.5%
Fenton, 2018	Radical Prostatectomy	Urinary incontinence	0.78	[0.46; 1.32]	0.8%
Fenton, 2018	Radical Prostatectomy	Erectile dysfunction	1.07	[0.81; 1.42]	1.3%
Fluri, 2010	Extracranial-intracranial arterial bypass	All-cause mortality	0.81	[0.47; 1.40]	0.8%
Fluri, 2010	Extracranial-intracranial arterial bypass	Stroke	1.24	[0.79; 1.94]	1.0%
Fluri, 2010	Extracranial-intracranial arterial bypass	Stroke mortality or dependency	1.17	[0.69; 2.00]	0.8%
Higgins, 2016	Bacillus Calmette-Guérin	All-cause mortality	1.46	[0.75; 2.84]	0.6%
Higgins, 2016	Measles containing vaccines	All-cause mortality	1.40	[0.88; 2.22]	0.9%
Jin, 2012	Total flavonoids	Colorectal neoplasms	1.09	[0.83; 1.43]	1.4%
Johnston, 2019	Red meat	All-cause mortality	1.08	[1.00; 1.17]	1.7%
Johnston, 2019	Red meat	Cardiovascular mortality	1.16	[0.96; 1.41]	1.5%
Johnston, 2019	Red meat	Cardiovascular disease	1.11	[0.95; 1.31]	1.6%
Keag, 2018	Caesarean section	Urinary incontinence	1.39	[0.96; 2.02]	1.1%
Keag, 2018	Caesarean section	Fecal incontinence	2.95	[0.82; 10.59]	0.2%
Kredo, 2014	Maintaining antiretroviral therapy	All-cause mortality	4.68	[1.12; 19.60]	0.2%
Li, 2014	Exenatide	Acute pancreatitis	0.93	[0.23; 3.77]	0.2%
Li, 2016	DDP-4 inhibitors	Heart failure	0.82	[0.55; 1.22]	1.1%
Li, 2016	DDP-4 inhibitors	Hospital admission for heart failure	1.33	[1.11; 1.59]	1.6%
Menne, 2019	SGLT-2 inhibitors	Acute kidney injury	1.88	[1.50; 2.34]	1.5%
Mesgarpour, 2017	Erythropoiesis stimulating agents	Venous thromboembolism	0.60	[0.19; 1.94]	0.3%
Mesgarpour, 2017	Erythropoiesis stimulating agents	All-cause mortality	0.76	[0.45; 1.27]	0.8%
Molnar, 2015	Neoral (Cyclosporin)	Acute rejection of kidney transplant	2.62	[1.11; 6.19]	0.4%
Nelson, 2010	Caesarean section	Anal incontinence, feces	1.10	[0.52; 2.34]	0.5%
Nelson, 2010	Caesarean section	Anal incontinence, flatus	0.81	[0.49; 1.36]	0.8%
Ochen, 2019	Surgery for achilles tendon rupture	Re-rupture	0.95	[0.49; 1.86]	0.6%
Ochen, 2019	Surgery for achilles tendon rupture	Complications	1.11	[0.42; 2.97]	0.4%
Raman, 2013	Carotid endarterectomy	Ipsilateral stroke	1.53	[0.16; 14.62]	0.1%
Raman, 2013	Carotid endarterectomy	Stroke	0.93	[0.53; 1.62]	0.8%
Raman, 2013	Carotid artery stenting	Periprocedural stroke	0.92	[0.45; 1.86]	0.6%
Schweizer, 2013	Glycopeptide prophylaxis	Surgical site infection	3.32	[1.03; 10.75]	0.3%
Silvain, 2012	Enoxaparin	All-cause mortality	1.80	[1.30; 2.48]	1.2%
Silvain, 2012	Enoxaparin	Major bleeding	1.22	[0.80; 1.88]	1.0%
Silvain, 2012	Enoxaparin	All-cause mortality or myocardial infarction	1.95	[1.49; 2.56]	1.4%
Tricco, 2018	Live-attenuated zoster vaccines	Suspected Herpes Zoster	1.27	[0.66; 2.45]	0.6%
Random effects model			1.13	[1.04; 1.22]	46.4%
Prediction interval				[0.76; 1.68]	--
Heterogeneity: $I^2 = 58\%$, $\tau^2 = 0.0374$, $p < 0.01$					
**more or less identical**					
Abou-Setta, 2011	Spinal anesthesia	All-cause mortality	1.99	[0.51; 7.71]	0.2%
Ahmad, 2015	Intra-aortic balloon pump	All-cause mortality	0.94	[0.50; 1.78]	0.7%
Alipanah, 2018	Self-administered therapy	Low treatment success	0.85	[0.75; 0.98]	1.7%
Alipanah, 2018	Self-administered therapy	Low treatment completion	1.40	[0.94; 2.08]	1.1%
Alipanah, 2018	Self-administered therapy	All-cause mortality	0.54	[0.30; 0.96]	0.7%
Barnard, 2015	Surgical abortion by mid-level providers	Failure or incomplete abortion	1.20	[0.08; 17.90]	0.1%
Barnard, 2015	Surgical abortion by mid-level providers	Complications	0.76	[0.11; 5.30]	0.1%
Barnard, 2015	Surgical abortion by mid-level providers	Abortion failure and complications	2.31	[0.11; 46.53]	0.0%
Bolland, 2015	Calcium	All fractures	0.88	[0.78; 0.99]	1.7%
Bolland, 2015	Calcium	Vertebral fracture	0.61	[0.45; 0.84]	1.3%
Bolland, 2015	Calcium	Hip fracture	0.87	[0.66; 1.16]	1.3%
Brenner, 2014	Sigmoidoscopy	Colorectal cancer mortality	1.22	[0.92; 1.62]	1.3%
Brenner, 2014	Sigmoidoscopy	Colorectal cancer incidence	1.64	[1.19; 2.27]	1.2%
Chowdhury, 2012	Omega-3	Cerebrovascular disease	1.09	[0.94; 1.27]	1.6%
Chung, 2016	Calcium	Cardiovascular mortality	1.06	[0.83; 1.35]	1.4%
Filippini, 2017	Disease-modifying drugs	Conversion to clinically definite multiple sclerosis	1.08	[0.66; 1.78]	0.9%
Gargiulo, 2016	Transcatheter aortic valve	Early all-cause mortality	0.74	[0.44; 1.24]	0.8%
Gargiulo, 2016	Transcatheter aortic valve	Mid-term all-cause mortality	0.90	[0.60; 1.34]	1.1%
Gargiulo, 2016	Transcatheter aortic valve	Long-term all-cause mortality	0.61	[0.35; 1.06]	0.8%
Hartling, 2013	Treating gestational diabetes mellitus	High birth weight	0.72	[0.30; 1.74]	0.4%
Hartling, 2013	Treating gestational diabetes mellitus	Large-for-gestational age neonate	1.30	[0.77; 2.20]	0.8%
Hartling, 2013	Treating gestational diabetes mellitus	Shoulder dystocia	1.11	[0.44; 2.80]	0.4%
Hopley, 2010	Total hip arthroplasty	Reoperation	2.42	[0.63; 9.34]	0.2%
Hopley, 2010	Total hip arthroplasty	Dislocation	3.09	[0.58; 16.47]	0.1%
Hopley, 2010	Total hip arthroplasty	Deep infection	1.88	[0.38; 9.33]	0.2%
Jamal, 2013	Non-calcium-based phosphat binders	All-cause mortality	0.88	[0.67; 1.15]	1.4%
Jefferson, 2010	Parenteral influenza vaccine	Influenza	0.84	[0.38; 1.86]	0.5%
Jefferson, 2012	Inactivated influenza vaccines	Influenza	2.05	[0.95; 4.42]	0.5%
Jefferson, 2012	Inactivated influenza vaccines	Influenza-like illness	2.21	[0.54; 9.04]	0.2%
Kredo, 2014	Starting and maintaining antiretroviral therapy	All-cause mortality	0.78	[0.66; 0.93]	1.6%
Kredo, 2014	Starting and maintaining antiretroviral therapy	Attrition	2.43	[0.38; 15.49]	0.1%
Matthews, 2018	Tamoxifen	Heart failure	0.62	[0.39; 0.98]	1.0%
Moberley, 2013	Pneumococcal polysaccharide vaccines	Invasive pneumococcal disease	0.46	[0.22; 0.95]	0.5%
Navarese, 2013	Early intervention for NSTE-ACS	All-cause mortality	1.04	[0.72; 1.49]	1.2%
Navarese, 2013	Early intervention for NSTE-ACS	Myocardial infarction	1.34	[0.73; 2.45]	0.7%
Navarese, 2013	Early intervention for NSTE-ACS	Major bleeding	0.68	[0.38; 1.21]	0.7%
Nikooie, 2019	Second generation antipsychotics	Sedation	0.68	[0.14; 3.27]	0.2%
Nikooie, 2019	Second generation antipsychotics	Neurologic outcomes	0.59	[0.25; 1.39]	0.4%
Schweizer, 2013	Nasal deconolization	Surgical site infection	1.57	[0.80; 3.09]	0.6%
Tickell-Painter, 2017	Mefloquine	Discontinuation due to adverse effects	1.05	[0.50; 2.20]	0.5%
Tickell-Painter, 2017	Mefloquine	Serious adverse events or effects	0.23	[0.02; 3.12]	0.1%
Tickell-Painter, 2017	Mefloquine	Nausea	0.73	[0.51; 1.05]	1.1%
Wilson, 2019	Unicompartimental knee arthroplasty	Venous thromboembolism	0.59	[0.10; 3.54]	0.1%
Yank, 2011	Recombinant factor VII	All-cause mortality	1.54	[0.40; 5.94]	0.2%
Yank, 2011	Recombinant factor VII	Thromboembolism	1.14	[0.20; 6.63]	0.1%
Zhang, 2016	Everolimus-eluting bioresorbable vascular scaffold	Stent thrombosis	0.88	[0.29; 2.65]	0.3%
Zhang, 2016	Everolimus-eluting bioresorbable vascular scaffold	All-cause mortality	1.68	[0.52; 5.45]	0.3%
Zhang, 2016	Everolimus-eluting bioresorbable vascular scaffold	Coronary heart disease mortality	1.73	[0.44; 6.72]	0.2%
Zhang, 2017	Percutaneous coronary intervention	All-cause mortality	0.93	[0.70; 1.23]	1.3%
Zhang, 2017	Percutaneous coronary intervention	Cardiovascular mortality	0.93	[0.41; 2.10]	0.5%
Zhang, 2017	Percutaneous coronary intervention	Myocardial infarction	0.69	[0.41; 1.18]	0.8%
Ziff, 2015	Digoxin	All-cause mortality	0.61	[0.50; 0.76]	1.5%
Ziff, 2015	Digoxin	Cardiovascular mortality	0.40	[0.18; 0.90]	0.5%
Ziff, 2015	Digoxin	Hospital admission	1.03	[0.97; 1.10]	1.8%
Random effects model			0.92	[0.84; 1.01]	39.1%
Prediction interval				[0.62; 1.37]	--
Heterogeneity: $I^2 = 51\%$, $\tau^2 = 0.0363$, $p < 0.01$					
**broadly similar**					
Anglemyer, 2013	Antiretroviral therapy	HIV infection	0.19	[0.06; 0.60]	0.3%
Chowdhury, 2014b	Vitamin D	All-cause mortality	1.42	[1.31; 1.54]	1.7%
Chung, 2011	Vitamin D	Colorectal cancer	1.09	[0.64; 1.85]	0.8%
Chung, 2011	Vitamin D	Breast cancer	1.00	[0.25; 4.00]	0.2%
Henderson, 2019	Treating asymptomatic bacteriuria	Pyelonephritis	0.83	[0.35; 1.93]	0.4%
Hüpfl, 2010	Chest-compression-only cardiopulmonary resuscitation	All-cause mortality	0.79	[0.62; 1.00]	1.4%
Jefferson, 2010	Parenteral influenza vaccine	Influenza-like illness	0.78	[0.60; 1.01]	1.4%
Kansagara, 2013	Transfusion	All-cause mortality	0.38	[0.18; 0.77]	0.6%
Pittas, 2010	Vitamin D	Hypertension	1.77	[1.27; 2.47]	1.2%
Suthar, 2012	Antiretroviral therapy	Tuberculosis infection	1.56	[0.98; 2.49]	0.9%
Thomas, 2010	Influenza vaccines	Influenza-like illness	2.29	[1.70; 3.08]	1.3%
Vinceti, 2018	Selenium	Cancer	1.32	[1.01; 1.73]	1.4%
Vinceti, 2018	Selenium	Cancer mortality	1.05	[0.61; 1.82]	0.8%
Vinceti, 2018	Selenium	Colorectal cancer	0.90	[0.49; 1.65]	0.7%
Wilson, 2011	Traditional birth attendants	Perinatal mortality	0.93	[0.42; 2.04]	0.5%
Wilson, 2011	Traditional birth attendants	Neonatal mortality	0.99	[0.57; 1.71]	0.8%
Random effects model			1.04	[0.80; 1.34]	14.5%
Prediction interval				[0.39; 2.80]	--
Heterogeneity: $I^2 = 82\%$, $\tau^2 = 0.1960$, $p < 0.01$					
**Random effects model**			**1.04**	**[0.97; 1.11]**	**100.0%**
**Prediction interval**				**[0.63; 1.71]**	
Heterogeneity: $I^2 = 69\%$, $\tau^2 = 0.0614$, $p < 0.01$					

Ratio of RRs/ ORs/ HRs (axis: 0.1, 0.5, 1, 2, 10)

RR/ OR/ HR in RCTs < RR/ OR/ HR in CSs — RR/ OR/ HR in RCTs > RR/ OR/ HR in CSs

Figure S2c. Forest plot for binary outcomes, pooled ratio of ratios (RoR) for bodies of evidence from randomized controlled trials vs. cohort studies stratified by comparator similarity degree.

CSs= cohort studies; DDP-4= dipeptidyl peptidase 4; DHA= docosahexaenoic acid; EPA= eicosapentaenoic acid; HR= hazard ratio; NSTE-ACS= non-ST elevation acute coronary syndrome; OR= odds ratio; RCTs= randomized controlled trials; RHR= ratio of hazard ratios; ROR= ratio of odds ratios; RR= risk ratio; RRR= ratio of risk ratios; SGLT-2= sodium glucose transporter 2.

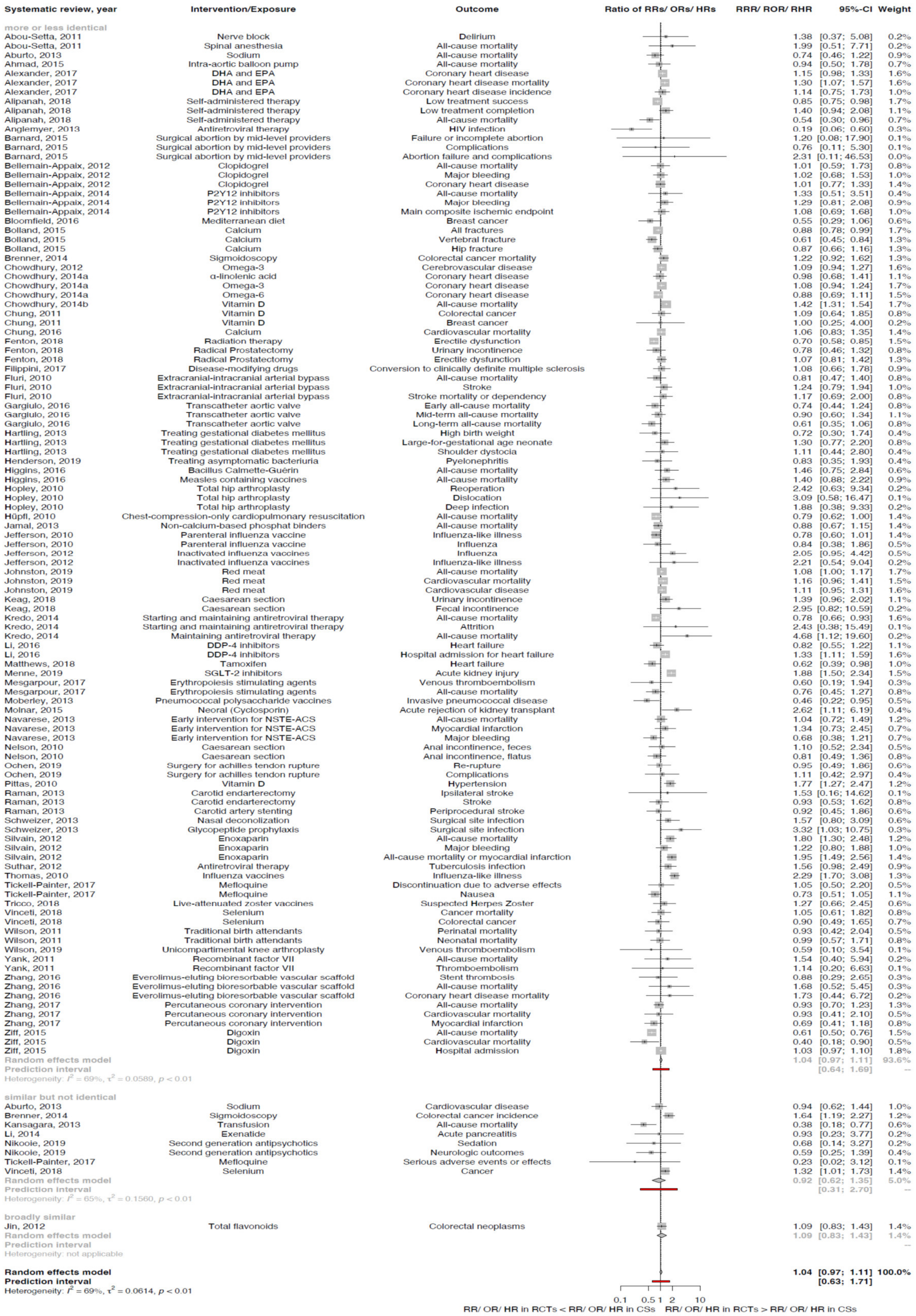

Figure S2d. Forest plot for binary outcomes, pooled ratio of ratios (RoR) for bodies of evidence from randomized controlled trials vs. cohort studies stratified by outcome similarity degree.

CSs= cohort studies; DDP-4= dipeptidyl peptidase 4; DHA= docosahexaenoic acid; EPA= eicosapentaenoic acid; HR= hazard ratio; NSTE-ACS= non-ST elevation acute coronary syndrome; OR= odds ratio; RCTs= randomized controlled trials; RHR= ratio of hazard ratios; ROR= ratio of odds ratios; RR= risk ratio; RRR= ratio of risk ratios; SGLT-2= sodium glucose transporter 2.

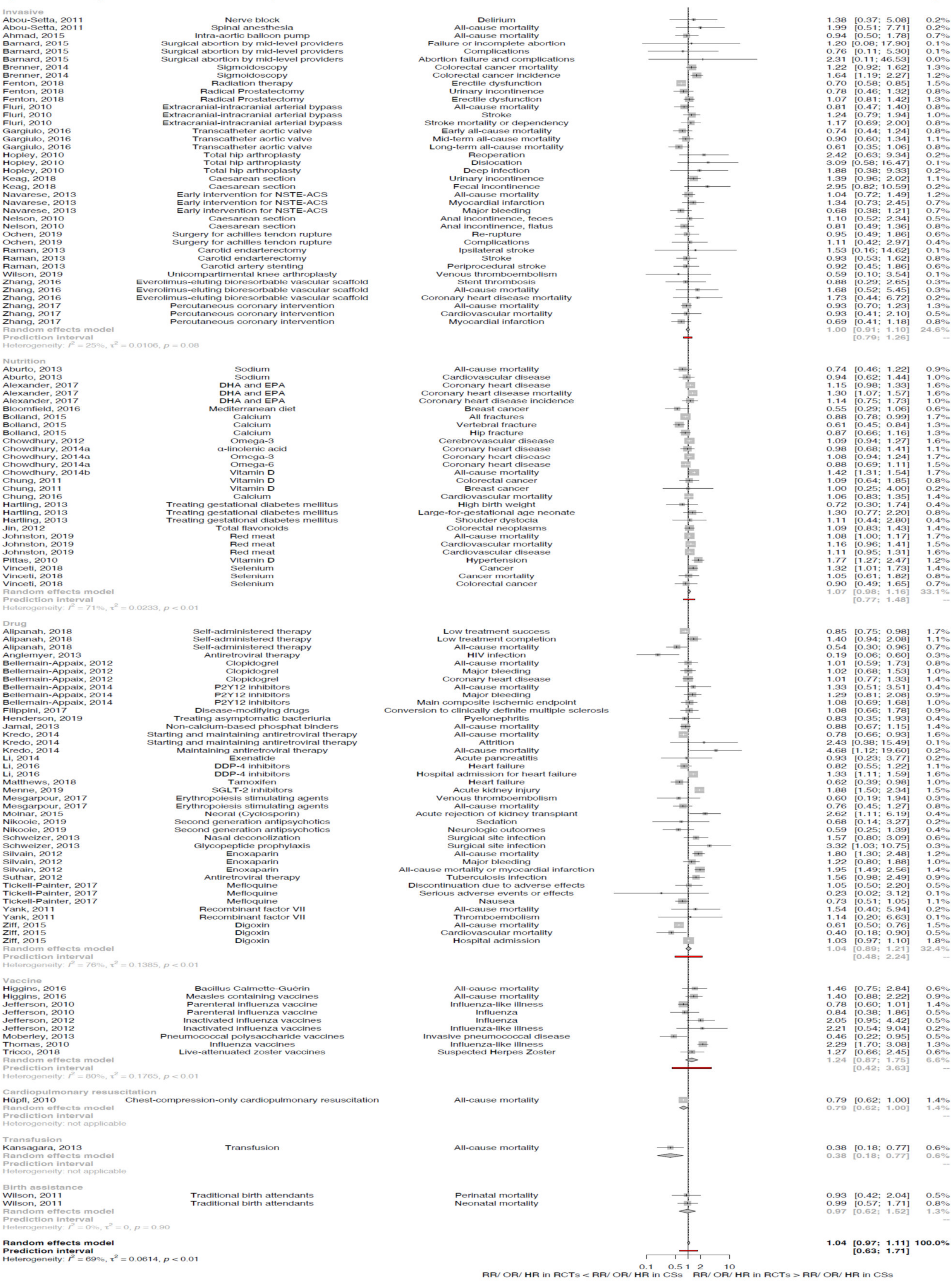

Systematic review, year	Intervention/Exposure	Outcome	RRR/ ROR/ RHR	95%-CI	Weight
Invasive					
Abou-Setta, 2011	Nerve block	Delirium	1.38	[0.37; 5.08]	0.2%
Abou-Setta, 2011	Spinal anesthesia	All-cause mortality	1.99	[0.51; 7.71]	0.2%
Ahmad, 2015	Intra-aortic balloon pump	All-cause mortality	0.94	[0.50; 1.78]	0.7%
Barnard, 2015	Surgical abortion by mid-level providers	Failure or incomplete abortion	1.20	[0.08; 17.90]	0.1%
Barnard, 2015	Surgical abortion by mid-level providers	Complications	0.76	[0.11; 5.30]	0.1%
Barnard, 2015	Surgical abortion by mid-level providers	Abortion failure and complications	2.31	[0.11; 46.53]	0.0%
Brenner, 2014	Sigmoidoscopy	Colorectal cancer mortality	1.22	[0.92; 1.62]	1.3%
Brenner, 2014	Sigmoidoscopy	Colorectal cancer incidence	1.64	[1.19; 2.27]	1.2%
Fenton, 2018	Radiation therapy	Erectile dysfunction	0.70	[0.58; 0.85]	1.5%
Fenton, 2018	Radical Prostatectomy	Urinary incontinence	0.78	[0.46; 1.32]	0.8%
Fenton, 2018	Radical Prostatectomy	Erectile dysfunction	1.07	[0.81; 1.42]	1.3%
Fluri, 2010	Extracranial-intracranial arterial bypass	All-cause mortality	0.81	[0.47; 1.40]	0.8%
Fluri, 2010	Extracranial-intracranial arterial bypass	Stroke	1.24	[0.79; 1.94]	1.0%
Fluri, 2010	Extracranial-intracranial arterial bypass	Stroke mortality or dependency	1.17	[0.69; 2.00]	0.8%
Gargiulo, 2016	Transcatheter aortic valve	Early all-cause mortality	0.74	[0.44; 1.24]	0.8%
Gargiulo, 2016	Transcatheter aortic valve	Mid-term all-cause mortality	0.90	[0.60; 1.34]	1.1%
Gargiulo, 2016	Transcatheter aortic valve	Long-term all-cause mortality	0.61	[0.35; 1.06]	0.8%
Hopley, 2010	Total hip arthroplasty	Reoperation	2.42	[0.63; 9.34]	0.2%
Hopley, 2010	Total hip arthroplasty	Dislocation	3.09	[0.58; 16.47]	0.1%
Hopley, 2010	Total hip arthroplasty	Deep infection	1.88	[0.38; 9.33]	0.2%
Keag, 2018	Caesarean section	Urinary incontinence	1.39	[0.96; 2.02]	1.1%
Keag, 2018	Caesarean section	Fecal incontinence	2.95	[0.82; 10.59]	0.2%
Navarese, 2013	Early intervention for NSTE-ACS	All-cause mortality	1.04	[0.72; 1.49]	1.2%
Navarese, 2013	Early intervention for NSTE-ACS	Myocardial infarction	1.34	[0.73; 2.45]	0.7%
Navarese, 2013	Early intervention for NSTE-ACS	Major bleeding	0.68	[0.38; 1.21]	0.7%
Nelson, 2010	Caesarean section	Anal incontinence, feces	1.10	[0.52; 2.34]	0.5%
Nelson, 2010	Caesarean section	Anal incontinence, flatus	0.81	[0.49; 1.36]	0.8%
Ochen, 2019	Surgery for achilles tendon rupture	Re-rupture	0.95	[0.49; 1.86]	0.6%
Ochen, 2019	Surgery for achilles tendon rupture	Complications	1.11	[0.42; 2.97]	0.4%
Raman, 2013	Carotid endarterectomy	Ipsilateral stroke	1.53	[0.16; 14.62]	0.1%
Raman, 2013	Carotid endarterectomy	Stroke	0.93	[0.53; 1.62]	0.8%
Raman, 2013	Carotid artery stenting	Periprocedural stroke	0.92	[0.45; 1.86]	0.6%
Wilson, 2019	Unicompartimental knee arthroplasty	Venous thromboembolism	0.59	[0.10; 3.54]	0.1%
Zhang, 2016	Everolimus-eluting bioresorbable vascular scaffold	Stent thrombosis	0.88	[0.29; 2.65]	0.3%
Zhang, 2016	Everolimus-eluting bioresorbable vascular scaffold	All-cause mortality	1.68	[0.52; 5.45]	0.3%
Zhang, 2016	Everolimus-eluting bioresorbable vascular scaffold	Coronary heart disease mortality	1.73	[0.44; 6.72]	0.2%
Zhang, 2017	Percutaneous coronary intervention	All-cause mortality	0.93	[0.70; 1.23]	1.3%
Zhang, 2017	Percutaneous coronary intervention	Cardiovascular mortality	0.93	[0.41; 2.10]	0.5%
Zhang, 2017	Percutaneous coronary intervention	Myocardial infarction	0.69	[0.41; 1.18]	0.8%
Random effects model			1.00	[0.91; 1.10]	24.6%
Prediction interval				[0.79; 1.26]	--
Heterogeneity: $I^2 = 25\%$, $\tau^2 = 0.0106$, $p = 0.08$					
Nutrition					
Aburto, 2013	Sodium	All-cause mortality	0.74	[0.46; 1.22]	0.9%
Aburto, 2013	Sodium	Cardiovascular disease	0.94	[0.62; 1.44]	1.0%
Alexander, 2017	DHA and EPA	Coronary heart disease	1.15	[0.98; 1.33]	1.6%
Alexander, 2017	DHA and EPA	Coronary heart disease mortality	1.30	[1.07; 1.57]	1.6%
Alexander, 2017	DHA and EPA	Coronary heart disease incidence	1.14	[0.75; 1.73]	1.0%
Bloomfield, 2016	Mediterranean diet	Breast cancer	0.55	[0.29; 1.06]	0.6%
Bolland, 2015	Calcium	All fractures	0.88	[0.78; 0.99]	1.7%
Bolland, 2015	Calcium	Vertebral fracture	0.61	[0.45; 0.84]	1.3%
Bolland, 2015	Calcium	Hip fracture	0.87	[0.66; 1.16]	1.3%
Chowdhury, 2012	Omega-3	Cerebrovascular disease	1.09	[0.94; 1.27]	1.6%
Chowdhury, 2014a	α-linolenic acid	Coronary heart disease	0.98	[0.68; 1.41]	1.1%
Chowdhury, 2014a	Omega-3	Coronary heart disease	1.08	[0.94; 1.24]	1.7%
Chowdhury, 2014a	Omega-6	Coronary heart disease	0.88	[0.69; 1.11]	1.5%
Chowdhury, 2014b	Vitamin D	All-cause mortality	1.42	[1.31; 1.54]	1.7%
Chung, 2011	Vitamin D	Colorectal cancer	1.09	[0.64; 1.85]	0.8%
Chung, 2011	Vitamin D	Breast cancer	1.00	[0.25; 4.00]	0.2%
Chung, 2016	Calcium	Cardiovascular mortality	1.06	[0.83; 1.35]	1.4%
Hartling, 2013	Treating gestational diabetes mellitus	High birth weight	0.72	[0.30; 1.74]	0.4%
Hartling, 2013	Treating gestational diabetes mellitus	Large-for-gestational age neonate	1.30	[0.77; 2.20]	0.8%
Hartling, 2013	Treating gestational diabetes mellitus	Shoulder dystocia	1.11	[0.44; 2.80]	0.4%
Jin, 2012	Total flavonoids	Colorectal neoplasms	1.09	[0.83; 1.43]	1.4%
Johnston, 2019	Red meat	All-cause mortality	1.08	[1.00; 1.17]	1.7%
Johnston, 2019	Red meat	Cardiovascular mortality	1.16	[0.96; 1.41]	1.5%
Johnston, 2019	Red meat	Cardiovascular disease	1.11	[0.95; 1.31]	1.6%
Pittas, 2010	Vitamin D	Hypertension	1.77	[1.27; 2.47]	1.2%
Vinceti, 2018	Selenium	Cancer	1.32	[1.01; 1.73]	1.4%
Vinceti, 2018	Selenium	Cancer mortality	1.05	[0.61; 1.82]	0.8%
Vinceti, 2018	Selenium	Colorectal cancer	0.90	[0.49; 1.65]	0.7%
Random effects model			1.07	[0.98; 1.16]	33.1%
Prediction interval				[0.77; 1.48]	--
Heterogeneity: $I^2 = 71\%$, $\tau^2 = 0.0233$, $p < 0.01$					
Drug					
Alipanah, 2018	Self-administered therapy	Low treatment success	0.85	[0.75; 0.98]	1.7%
Alipanah, 2018	Self-administered therapy	Low treatment completion	1.40	[0.94; 2.08]	1.1%
Alipanah, 2018	Self-administered therapy	All-cause mortality	0.54	[0.30; 0.96]	0.7%
Anglemyer, 2013	Antiretroviral therapy	HIV infection	0.19	[0.06; 0.60]	0.3%
Bellemain-Appaix, 2012	Clopidogrel	All-cause mortality	1.01	[0.59; 1.73]	0.8%
Bellemain-Appaix, 2012	Clopidogrel	Major bleeding	1.02	[0.68; 1.53]	1.0%
Bellemain-Appaix, 2012	Clopidogrel	Coronary heart disease	1.01	[0.77; 1.33]	1.4%
Bellemain-Appaix, 2014	P2Y12 inhibitors	All-cause mortality	1.33	[0.51; 3.51]	0.4%
Bellemain-Appaix, 2014	P2Y12 inhibitors	Major bleeding	1.29	[0.81; 2.08]	0.9%
Bellemain-Appaix, 2014	P2Y12 inhibitors	Main composite ischemic endpoint	1.08	[0.69; 1.68]	1.0%
Filippini, 2017	Disease-modifying drugs	Conversion to clinically definite multiple sclerosis	1.08	[0.66; 1.78]	0.9%
Henderson, 2019	Treating asymptomatic bacteriuria	Pyelonephritis	0.83	[0.35; 1.93]	0.4%
Jamal, 2013	Non-calcium-based phosphat binders	All-cause mortality	0.88	[0.67; 1.15]	1.4%
Kredo, 2014	Starting and maintaining antiretroviral therapy	All-cause mortality	0.78	[0.66; 0.93]	1.6%
Kredo, 2014	Starting and maintaining antiretroviral therapy	Attrition	2.43	[0.38; 15.49]	0.1%
Kredo, 2014	Maintaining antiretroviral therapy	All-cause mortality	4.68	[1.12; 19.60]	0.2%
Li, 2014	Exenatide	Acute pancreatitis	0.93	[0.23; 3.77]	0.2%
Li, 2016	DDP-4 inhibitors	Heart failure	0.82	[0.55; 1.22]	1.1%
Li, 2016	DDP-4 inhibitors	Hospital admission for heart failure	1.33	[1.11; 1.59]	1.6%
Matthews, 2018	Tamoxifen	Heart failure	0.62	[0.39; 0.98]	1.0%
Menne, 2019	SGLT-2 inhibitors	Acute kidney injury	1.88	[1.50; 2.34]	1.5%
Mesgarpour, 2017	Erythropoiesis stimulating agents	Venous thromboembolism	0.60	[0.19; 1.94]	0.3%
Mesgarpour, 2017	Erythropoiesis stimulating agents	All-cause mortality	0.76	[0.45; 1.27]	0.8%
Molnar, 2015	Neoral (Cyclosporin)	Acute rejection of kidney transplant	2.62	[1.11; 6.19]	0.4%
Nikooie, 2019	Second generation antipsychotics	Sedation	0.68	[0.14; 3.27]	0.2%
Nikooie, 2019	Second generation antipsychotics	Neurologic outcomes	0.59	[0.25; 1.39]	0.4%
Schweizer, 2013	Nasal deconolization	Surgical site infection	1.57	[0.80; 3.09]	0.6%
Schweizer, 2013	Glycopeptide prophylaxis	Surgical site infection	3.32	[1.03; 10.75]	0.3%
Silvain, 2012	Enoxaparin	All-cause mortality	1.80	[1.30; 2.48]	1.2%
Silvain, 2012	Enoxaparin	Major bleeding	1.22	[0.80; 1.88]	1.0%
Silvain, 2012	Enoxaparin	All-cause mortality or myocardial infarction	1.95	[1.49; 2.56]	1.4%
Suthar, 2012	Antiretroviral therapy	Tuberculosis infection	1.56	[0.98; 2.49]	0.9%
Tickell-Painter, 2017	Mefloquine	Discontinuation due to adverse effects	1.05	[0.50; 2.20]	0.5%
Tickell-Painter, 2017	Mefloquine	Serious adverse events or effects	0.23	[0.02; 3.12]	0.1%
Tickell-Painter, 2017	Mefloquine	Nausea	0.73	[0.51; 1.05]	1.1%
Yank, 2011	Recombinant factor VII	All-cause mortality	1.54	[0.40; 5.94]	0.2%
Yank, 2011	Recombinant factor VII	Thromboembolism	1.14	[0.20; 6.63]	0.1%
Ziff, 2015	Digoxin	All-cause mortality	0.61	[0.50; 0.76]	1.5%
Ziff, 2015	Digoxin	Cardiovascular mortality	0.40	[0.18; 0.90]	0.5%
Ziff, 2015	Digoxin	Hospital admission	1.03	[0.97; 1.10]	1.8%
Random effects model			1.04	[0.89; 1.21]	32.4%
Prediction interval				[0.48; 2.24]	--
Heterogeneity: $I^2 = 76\%$, $\tau^2 = 0.1385$, $p < 0.01$					
Vaccine					
Higgins, 2016	Bacillus Calmette-Guérin	All-cause mortality	1.46	[0.75; 2.84]	0.6%
Higgins, 2016	Measles containing vaccines	All-cause mortality	1.40	[0.88; 2.22]	0.9%
Jefferson, 2010	Parenteral influenza vaccine	Influenza-like illness	0.78	[0.60; 1.01]	1.4%
Jefferson, 2010	Parenteral influenza vaccine	Influenza	0.84	[0.38; 1.86]	0.5%
Jefferson, 2012	Inactivated influenza vaccines	Influenza	2.05	[0.95; 4.42]	0.5%
Jefferson, 2012	Inactivated influenza vaccines	Influenza-like illness	2.21	[0.54; 9.04]	0.2%
Moberley, 2013	Pneumococcal polysaccharide vaccines	Invasive pneumococcal disease	0.46	[0.22; 0.95]	0.5%
Thomas, 2010	Influenza vaccines	Influenza-like illness	2.29	[1.70; 3.08]	1.3%
Tricco, 2018	Live-attenuated zoster vaccines	Suspected Herpes Zoster	1.27	[0.66; 2.45]	0.6%
Random effects model			1.24	[0.87; 1.75]	6.6%
Prediction interval				[0.42; 3.63]	--
Heterogeneity: $I^2 = 80\%$, $\tau^2 = 0.1765$, $p < 0.01$					
Cardiopulmonary resuscitation					
Hüpfl, 2010	Chest-compression-only cardiopulmonary resuscitation	All-cause mortality	0.79	[0.62; 1.00]	1.4%
Random effects model			0.79	[0.62; 1.00]	1.4%
Prediction interval					--
Heterogeneity: not applicable					
Transfusion					
Kansagara, 2013	Transfusion	All-cause mortality	0.38	[0.18; 0.77]	0.6%
Random effects model			0.38	[0.18; 0.77]	0.6%
Prediction interval					--
Heterogeneity: not applicable					
Birth assistance					
Wilson, 2011	Traditional birth attendants	Perinatal mortality	0.93	[0.42; 2.04]	0.5%
Wilson, 2011	Traditional birth attendants	Neonatal mortality	0.99	[0.57; 1.71]	0.8%
Random effects model			0.97	[0.62; 1.52]	1.3%
Prediction interval					--
Heterogeneity: $I^2 = 0\%$, $\tau^2 = 0$, $p = 0.90$					
**Random effects model**			**1.04**	**[0.97; 1.11]**	**100.0%**
**Prediction interval**				**[0.63; 1.71]**	
Heterogeneity: $I^2 = 69\%$, $\tau^2 = 0.0614$, $p < 0.01$					

Figure S3. Forest plot for binary outcomes, pooled ratio of ratios (RoR) for bodies of evidence from randomized controlled trials vs. cohort studies stratified by intervention-type.

CSs= cohort studies; DDP-4= dipeptidyl peptidase 4; DHA= docosahexaenoic acid; EPA= eicosapentaenoic acid; HR= hazard ratio; NSTE-ACS= non-ST elevation acute coronary syndrome; OR= odds ratio; RCTs= randomized controlled trials; RHR= ratio of hazard ratios; ROR= ratio of odds ratios; RR= risk ratio; RRR= ratio of risk ratios; SGLT-2= sodium glucose transporter 2.

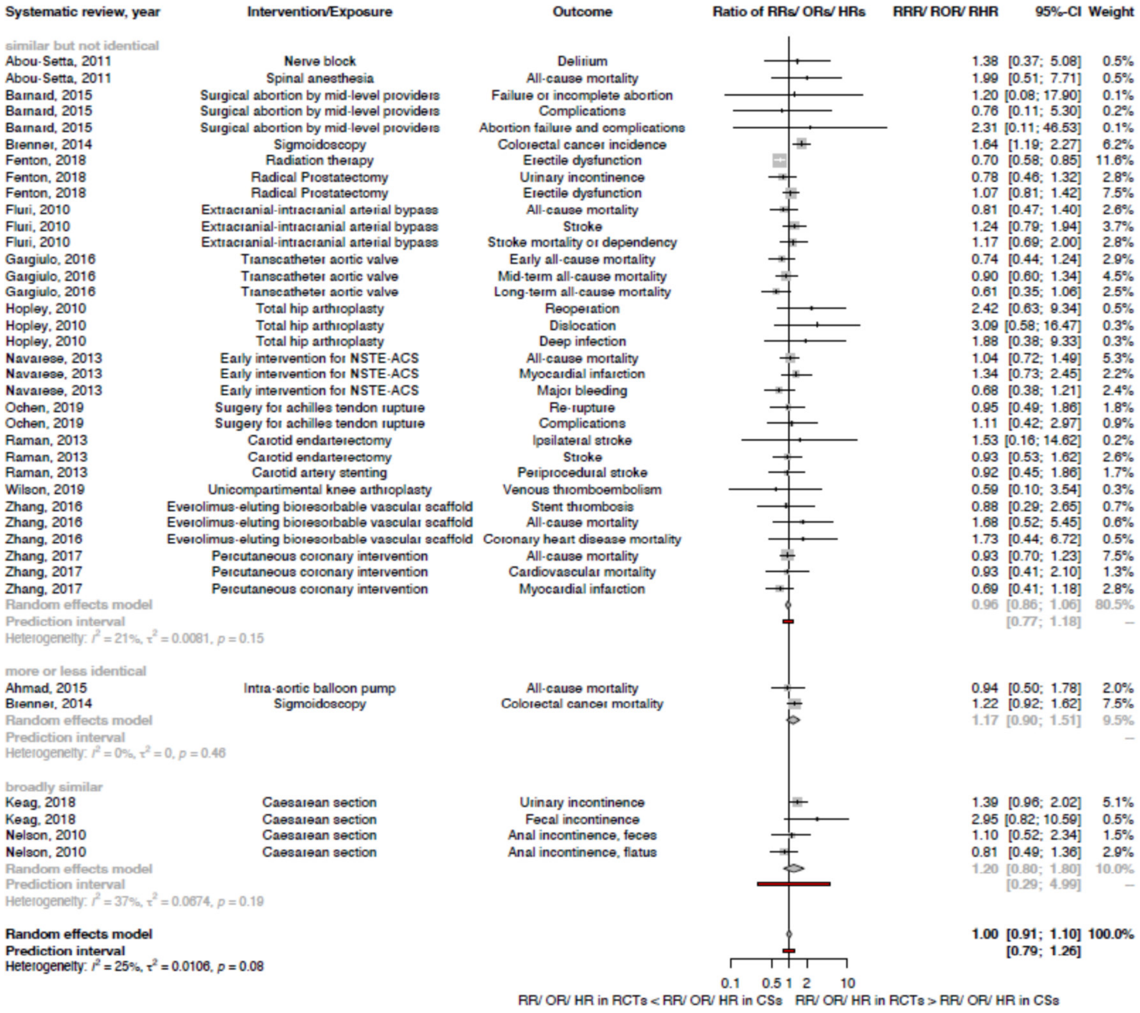

Figure S3a. Forest plot for binary outcomes, pooled ratio of ratios (RoR) for bodies of evidence (BoE) from randomized controlled trials vs. cohort studies, BoE with invasive procedures as intervention stratified by overall PI/ECO*-similarity degree.

*PI/ECO= population, intervention/ exposure, comparator, outcome; CSs= cohort studies; HR= hazard ratio; NSTE-ACS= non-ST elevation acute coronary syndrome; OR= odds ratio; RCTs= randomized controlled trials; RHR= ratio of hazard ratios; ROR= ratio of odds ratios; RR= risk ratio; RRR= ratio of risk ratios.

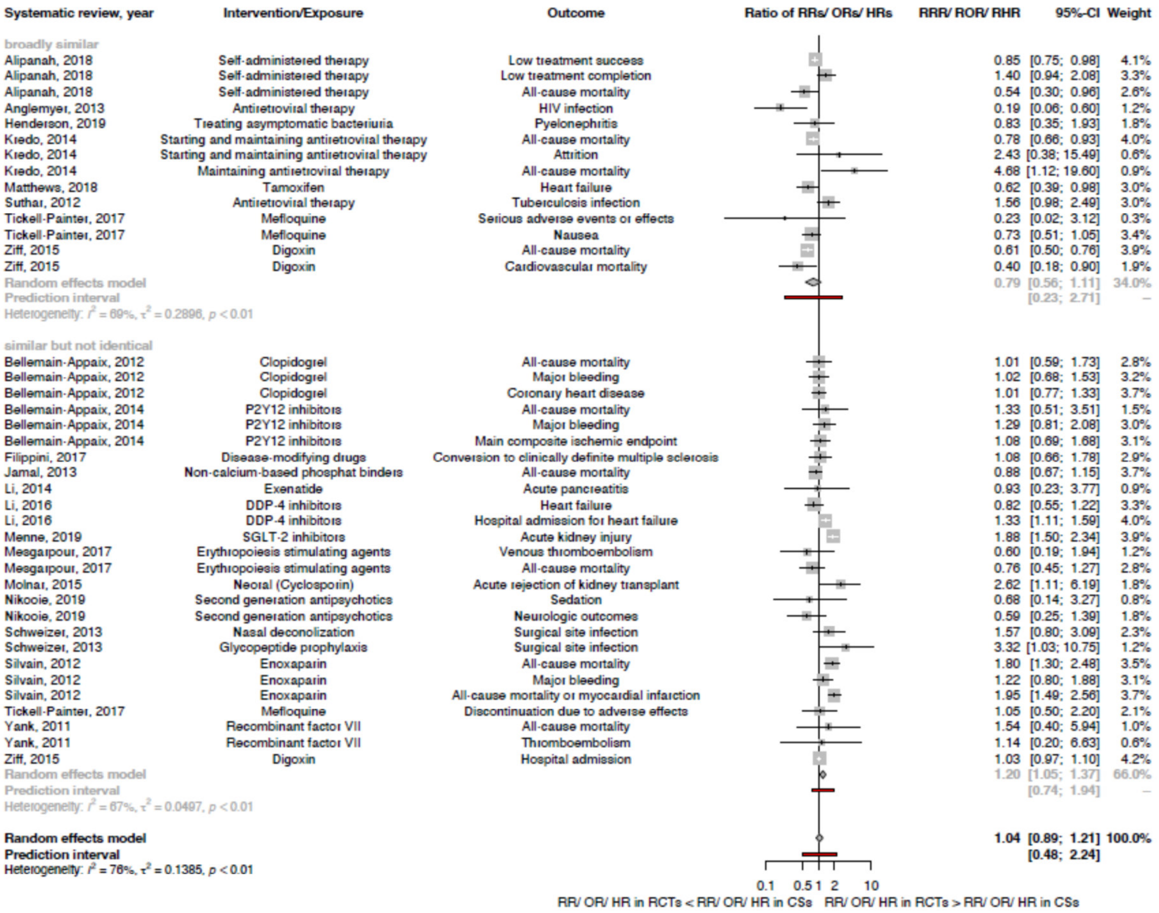

Figure S3b. Forest plot for binary outcomes, pooled ratio of ratios (RoR) for bodies of evidence (BoE) from randomized controlled trials vs. cohort studies, BoE with drugs as intervention stratified by overall PI/ECO-similarity degree.

*PI/ECO= population, intervention/ exposure, comparator, outcome; CSs= cohort studies; DDP-4= dipeptidyl peptidase 4; HR= hazard ratio; OR= odds ratio; RCTs= randomized controlled trials; RHR= ratio of hazard ratios; ROR= ratio of odds ratios; RR= risk ratio; RRR= ratio of risk ratios; SGLT-2= sodium glucose transporter 2.

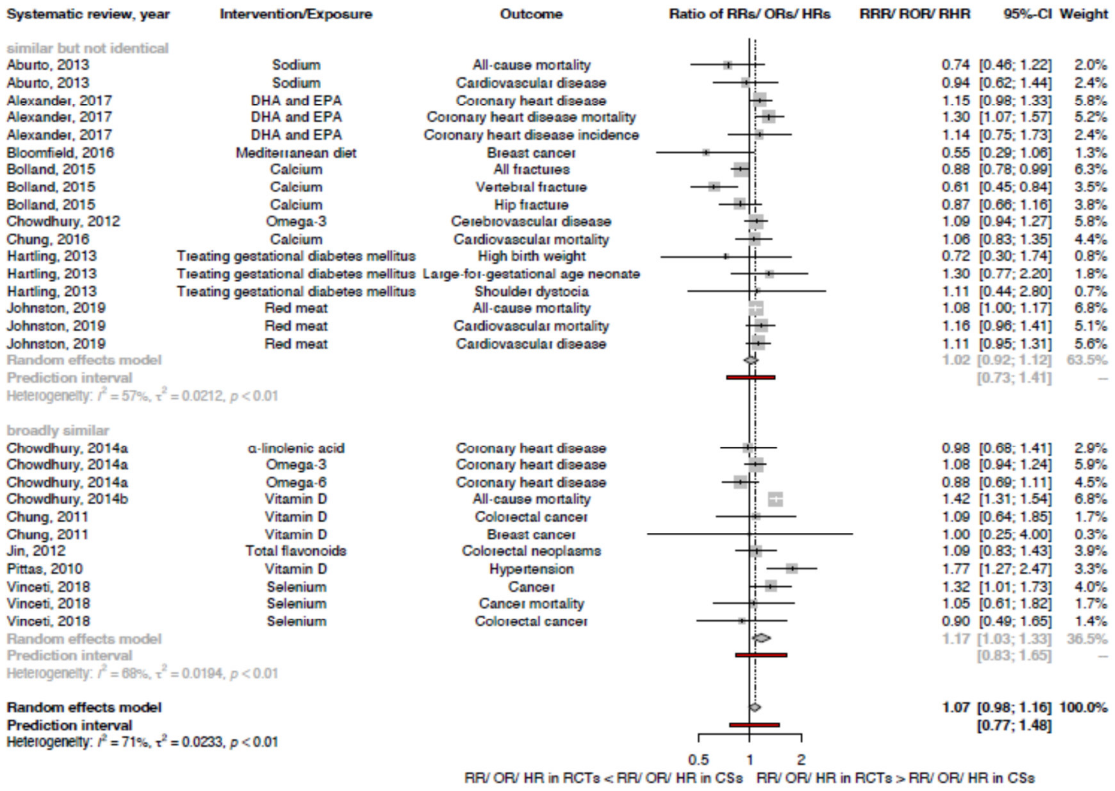

Figure S3c. Forest plot for binary outcomes, pooled ratio of ratios (RoR) for bodies of evidence (BoE) from randomized controlled trials vs. cohort studies, BoE with nutrition as intervention stratified by overall PI/ECO-similarity degree.

*PI/ECO= population, intervention/ exposure, comparator, outcome; CSs= cohort studies; DHA= docosahexaenoic acid; EPA= eicosapentaenoic acid; HR= hazard ratio; OR= odds ratio; RCTs= randomized controlled trials; RHR= ratio of hazard ratios; ROR= ratio of odds ratios; RR= risk ratio; RRR= ratio of risk ratios.

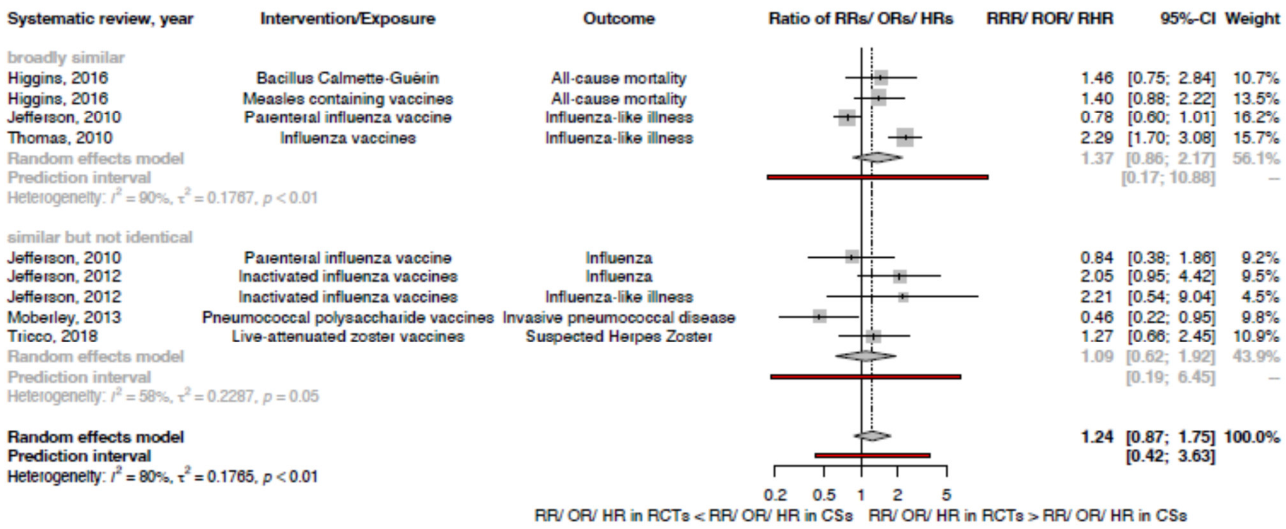

Figure S3d. Forest plot for binary outcomes, pooled ratio of ratios (RoR) for bodies of evidence (BoE) from randomized controlled trials vs. cohort studies, BoE with vaccines as intervention stratified by overall PI/ECO-similarity degree.

*PI/ECO= population, intervention/ exposure, comparator, outcome; CSs= cohort studies; HR= hazard ratio; OR= odds ratio; RCTs= randomized controlled trials; RHR= ratio of hazard ratios; ROR= ratio of odds ratios; RR= risk ratio; RRR= ratio of risk ratios.

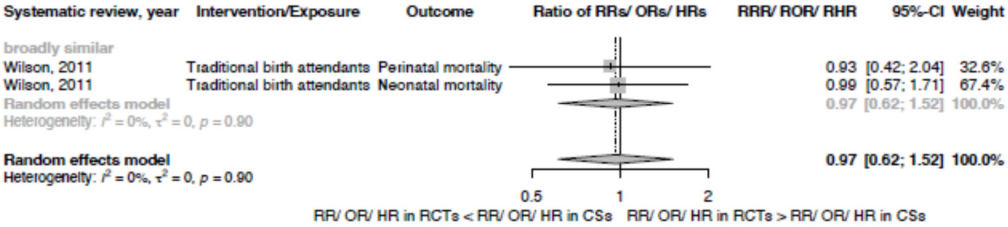

Figure S3e. Forest plot for binary outcomes, pooled ratio of ratios (RoR) for bodies of evidence (BoE) from randomized controlled trials vs. cohort studies, BoE with birth assistance as intervention stratified by overall PI/ECO-similarity degree.

*PI/ECO= population, intervention/ exposure, comparator, outcome; CSs= cohort studies; HR= hazard ratio; OR= odds ratio; RCTs= randomized controlled trials; RHR= ratio of hazard ratios; ROR= ratio of odds ratios; RR= risk ratio; RRR= ratio of risk ratios.

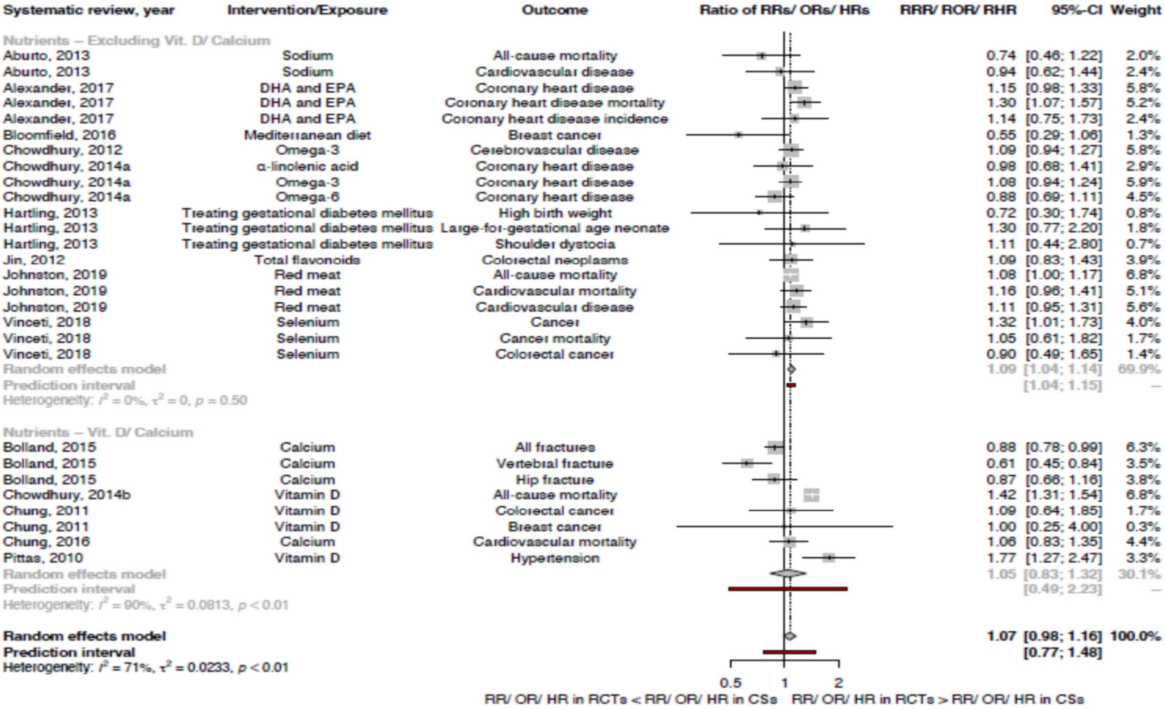

Figure S4. Forest plot for binary outcomes, pooled ratio of ratios (RoR) for bodies of evidence from randomized controlled trials vs. cohort studies, exploratory analysis for BoE with nutrition as intervention: Vitamin D/ Calcium as intervention vs. other nutrition-interventions.

CSs= cohort studies; DHA= docosahexaenoic acid; EPA= eicosapentaenoic acid; HR= hazard ratio; OR= odds ratio; RCTs= randomized controlled trials; RHR= ratio of hazard ratios; ROR= ratio of odds ratios; RR= risk ratio; RRR= ratio of risk ratios.

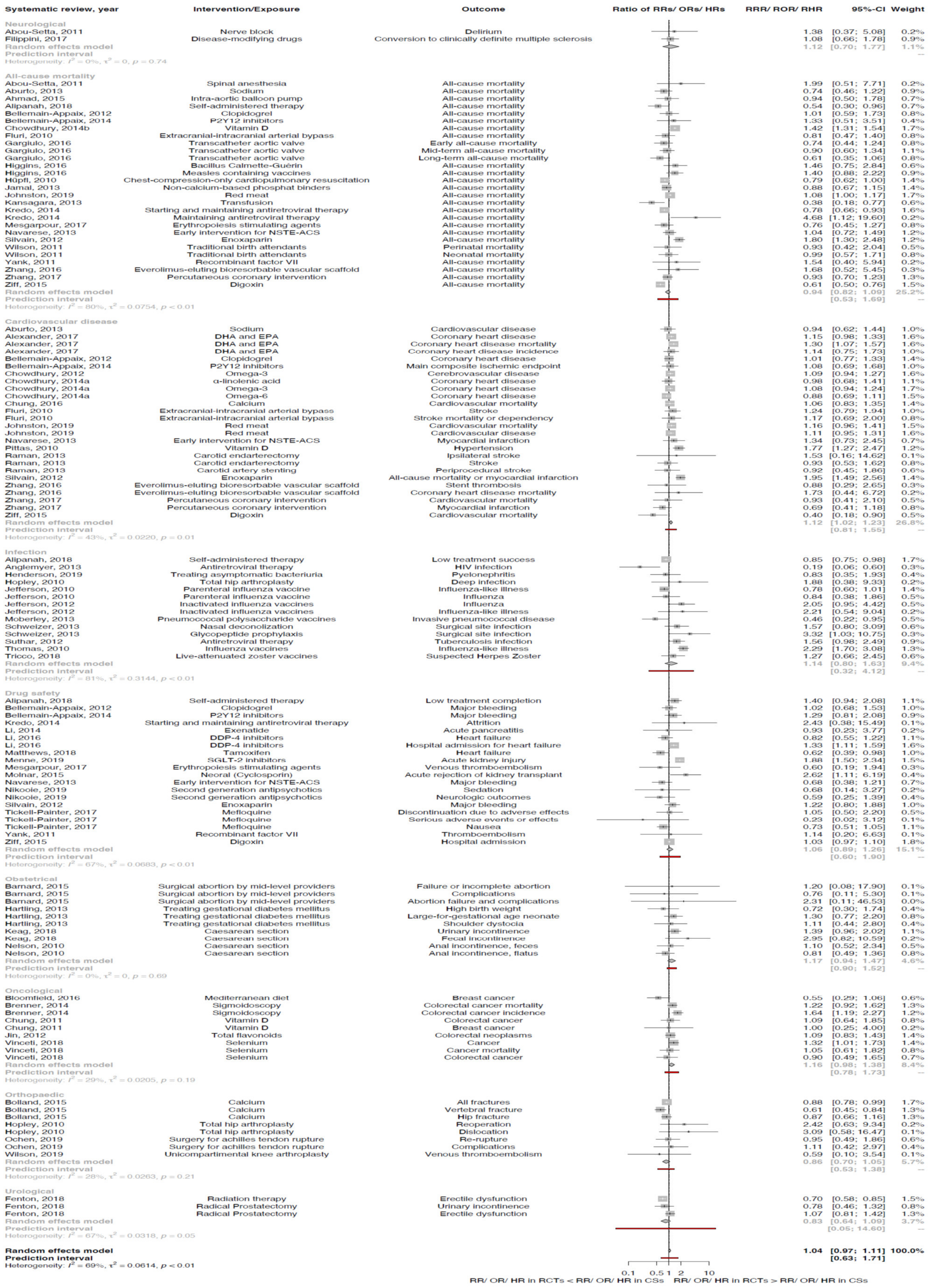

Figure S5. Forest plot for binary outcomes, pooled ratio of ratios (RoR) for bodies of evidence from randomized controlled trials vs. cohort studies stratified by outcome-category.

CSs= cohort studies; DDP-4= dipeptidyl peptidase 4; DHA= docosahexaenoic acid; EPA= eicosapentaenoic acid; HR= hazard ratio; NSTE-ACS= non-ST elevation acute coronary syndrome; OR= odds ratio; RCTs= randomized controlled trials; RHR= ratio of hazard ratios; ROR= ratio of odds ratios; RR= risk ratio; RRR= ratio of risk ratios; SGLT-2= sodium glucose transporter 2.

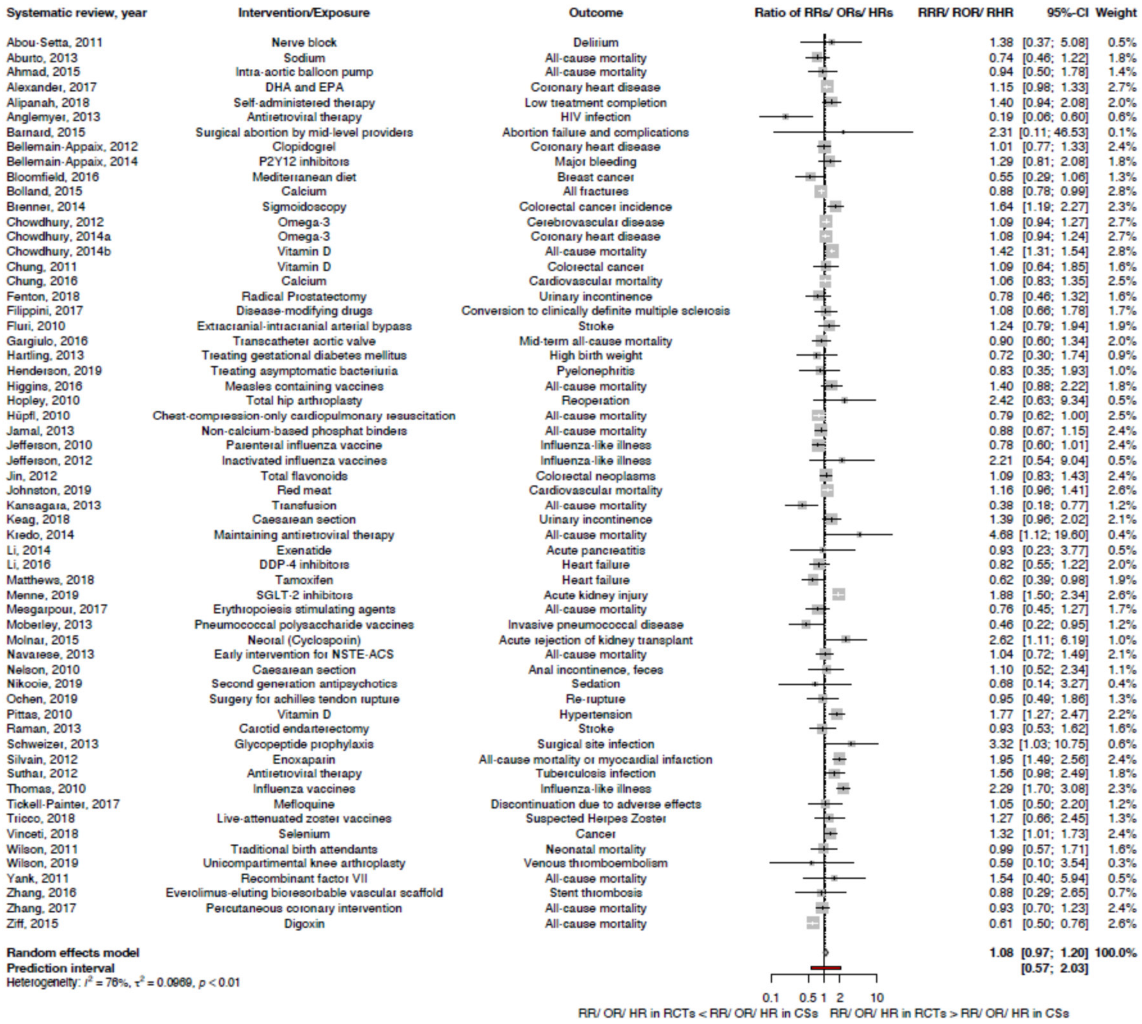

Figure S6. Forest plot for binary outcomes, pooled ratio of ratios (RoR) for bodies of evidence (BoE) from randomized controlled trials (RCTs) vs. cohort studies, sensitivity analysis including only the BoE-pair with the highest number of RCTs per systematic review.

CSs= cohort studies; DDP-4= dipeptidyl peptidase 4; DHA= docosahexaenoic acid; EPA= eicosapentaenoic acid; HR= hazard ratio; NSTE-ACS= non-ST elevation acute coronary syndrome; OR= odds ratio; RCTs= randomized controlled trials; RHR= ratio of hazard ratios; ROR= ratio of odds ratios; RR= risk ratio; RRR= ratio of risk ratios; SGLT-2= sodium glucose transporter 2.

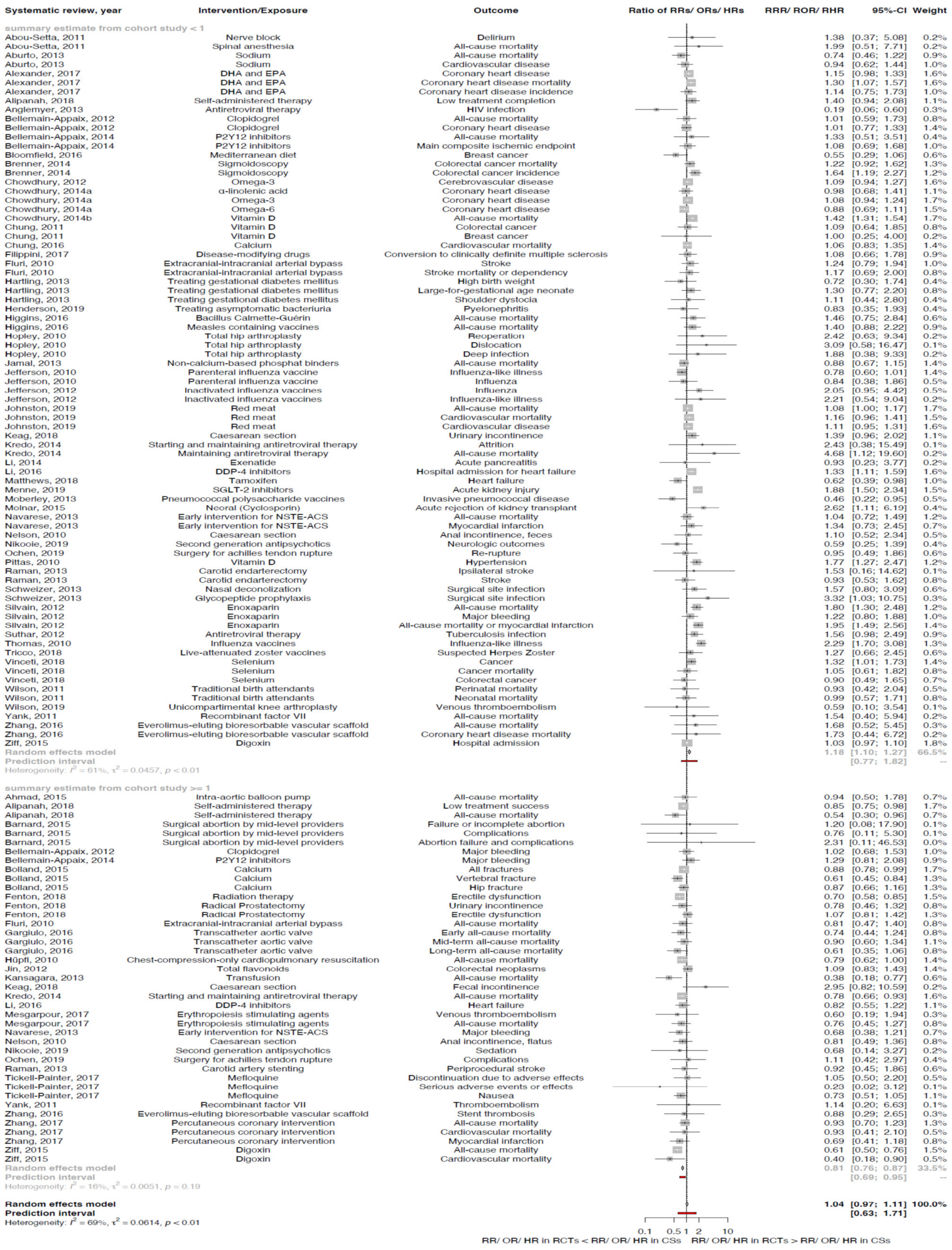

Figure S7. Forest plot for binary outcomes, pooled ratio of ratios (RoR) for bodies of evidence from randomized controlled trials vs. cohort studies, sensitivity analysis by direction of cohort study summary effect estimate (HR, OR, RR<1 vs. HR, OR, RR ≥ 1).

CSs= cohort studies; DDP-4= dipeptidyl peptidase 4; DHA= docosahexaenoic acid; EPA= eicosapentaenoic acid; HR= hazard ratio; NSTE-ACS= non-ST elevation acute coronary syndrome; OR= odds ratio; RCTs= randomized controlled trials; RHR= ratio of hazard ratios; ROR= ratio of odds ratios; RR= risk ratio; RRR= ratio of risk ratios; SGLT-2= sodium glucose transporter 2.
